# Supplementary material for: Prevalence of Alpha(α)-Thalassemia in Southeast Asia (2010–2020): A Meta-Analysis Involving 83,674 Subjects
Source: Int J Environ Res Public Health. 2020 Oct 9;17(20):7354. doi: 10.3390/ijerph17207354 (PMC7600098; doi:10.3390/ijerph17207354)
Supplement: Supplementary file 1 [file ijerph-17-07354-s001.zip › ijerph-909078-supplementary/Table S2. Initial literature search from Pubmed..pdf]

Table S2: Initial literature search from Pubmed.

Title	Authors	Details
Management and Outcomes of Fetal Hydrops in a Tertiary Care Centre in Singapore.	Thong XY, Lee LY, Chia DA, Wong YC, Biswas A.	Ann Acad Med Singapore. 2017 Jan;46(1):4-10.
Diagnosis of common hemoglobinopathies among South East Asian population using capillary isoelectric focusing system.	Srivorakun H, Fucharoen G, Sanchaisuriya K, Fucharoen S.	Int J Lab Hematol. 2017 Feb;39(1):101-111. doi: 10.1111/ijlh.12585.
Health-Related Quality of Life and Health Utility Values in Beta Thalassemia Major Patients Receiving Different Types of Iron Chelators in Iran.	Seyedifar M, Dorkoosh FA, Hamidieh AA, Naderi M, Karami H, Karimi M, Fadaiyrayeny M, Musavi M, Safaei S, Ahmadian-Attari MM, Hadjibabaie M, Cheraghali AM, Akbari Sari A.	Int J Hematol Oncol Stem Cell Res. 2016 Oct 1;10(4):224-231.
Iron dysregulation in beta-thalassemia.	Leecharoenkiat K, Lithanatudom P, Sornjai W, Smith DR.	Asian Pac J Trop Med. 2016 Nov;9(11):1035-1043. doi: 10.1016/j.apjtm.2016.07.035. Review.
New trend in the epidemiology of thalassaemia.	Li CK.	Best Pract Res Clin Obstet Gynaecol. 2017 Feb;39:16-26. doi: 10.1016/j.bpobgyn.2016.10.013. Review.
Diagnosis of imported Ugandan typhoid fever based on local outbreak information: A case report.	Ota S, Maki Y, Mori K, Hamamoto T, Kurokawa A, Ishihara M, Yamamoto T, Imai K, Misawa K, Yuki A, Fujikura Y, Maeda T, Kawana A.	J Infect Chemother. 2016 Nov;22(11):770-773. doi: 10.1016/j.jiac.2016.05.004.
Molecular Understanding of Non-Transfusion-Dependent Thalassemia Associated with Hemoglobin E-Î²-Thalassemia in Northeast Thailand.	Yamsri S, Pakdee N, Fucharoen G, Sanchaisuriya K, Fucharoen S.	Acta Haematol. 2016;136(4):233-239.
HbE/Î²-Thalassemia and Oxidative Stress: The Key to Pathophysiological Mechanisms and Novel Therapeutics.	Hirsch RE, Sibmooh N, Fucharoen S, Friedman JM.	Antioxid Redox Signal. 2016 Nov 28. [Epub ahead of print]
A Number of Cases in Iran Presenting with Coinheritance of Hemoglobin-H Disease and Beta-Thalassemia Minor.	Zarei T, Dehbozorgian J, Imanifard J, Setoodegan F, Karimi M.	Hemoglobin. 2016 Sep;40(5):316-318.
[Gene Diagnosis and Analysis of Clinical Hematological Phenotype of Thailand Deleted Î±-Thalassemia 1].	Lin N, Huang HL, Wang Y, Zheng L, Fang XQ, Cai MY, Wang LS, Liu HK, Xu LP, Lin Y.	Zhongguo Shi Yan Xue Ye Xue Za Zhi. 2016 Aug;24(4):1116-20. doi: 10.7534/j.issn.1009-2137.2016.04.029. Chinese.
Occurrence of the -â€‰-(SEA), -â€‰-(THAI) and -â€‰-(FIL) Î±-Thalassemia-1 Carriers from a 7-Year Study at Ramathibodi Hospital, Bangkok, Thailand.	Pongjantharasatien K, Banyatsuppasin W, Pounsawat S, Jindadamrongwech S.	Hemoglobin. 2016 Aug;40(4):283-4. doi: 10.1080/03630269.2016.1189932.
Ultrafast Magnetic Resonance Imaging for Iron Quantification in Thalassemia Participants in the	Abdel-Gadir A, Vorasettakarnkij Y, Ngamkasem H, Nordin S, Ako EA, Tumkosit M, Sucharitchan P, Uaprasert N,	Circulation. 2016 Aug 2;134(5):432-4. doi: 10.1161/CIRCULATIONAHA.1

Developing World: The TIC-TOC Study (Thailand and UK International Collaboration in Thalassaemia Optimising Ultrafast CMR).	Kellman P, Piechnik SK, Fontana M, Fernandes JL, Manisty C, Westwood M, Porter JB, Walker JM, Moon JC.	16.022803. No abstract available.
Detection of the common South-East Asian Î²0-thalassemia mutations in samples with borderline HbA2 levels.	Rungsee P, Kongthai K, Pornprasert S.	Clin Chem Lab Med. 2017 Jan 1;55(1):e17-e20. doi: 10.1515/cclm-2016-0470. No abstract available.
Hemoglobin E Hemoglobinopathy in an Adult from Assam with Unusual Presentation: A Diagnostic Dilemma.	Kiran SS, Aithal S, Belagavi CS.	J Lab Physicians. 2016 Jul-Dec;8(2):116-9. doi: 10.4103/0974-2727.180793.
Rapid detection of Î±-thalassaemia variants using droplet digital PCR.	Lee TY, Lai MI, Ramachandran V, Tan JA, Teh LK, Othman R, Hussein NH, George E.	Int J Lab Hematol. 2016 Aug;38(4):435-43. doi: 10.1111/ijlh.12520.
Screening of (-SEA) Î±-thalassaemia using an immunochromatographic strip assay for the Î¶-globin chain in a population with a high prevalence and heterogeneity of haemoglobinopathies.	Jomoui W, Fucharoen G, Sanchaisuriya K, Fucharoen S.	J Clin Pathol. 2017 Jan;70(1):63-68. doi: 10.1136/jclinpath-2016-203765.
Analysis of Î±1 and Î±2 globin genes among patients with hemoglobin Adana in Malaysia.	Lee TY, Lai MI, Ismail P, Ramachandran V, Tan JA, Teh LK, Othman R, Hussein NH, George E.	Genet Mol Res. 2016 Apr 7;15(2). doi: 10.4238/gmr.15027400.
Spectrum of Common Î±-Globin Deletion Mutations in the Southern Region of Vietnam.	Bui Thi Kim L, Phu Chi D, Hoang Thanh C.	Hemoglobin. 2016 Jun;40(3):206-7. doi: 10.3109/03630269.2016.1166126.
Molecular Epidemiology of Hemoglobinopathies in Cambodia.	Munkongdee T, Tanakulmas J, Butthep P, Winichagoon P, Main B, Yiannakis M, George J, Devenish R, Fucharoen S, Svasti S.	Hemoglobin. 2016 Jun;40(3):163-7. doi: 10.3109/03630269.2016.1158723.
The prevalence of alpha-thalassemia amongst Tai and Mon-Khmer ethnic groups residing in northern Thailand: A population-based study.	Lithanatudom P, Khampan P, Smith DR, Svasti S, Fucharoen S, Kangwanpong D, Kampuansai J.	Hematology. 2016 Sep;21(8):480-5. doi: 10.1080/10245332.2016.1148374.
Modifying effect of XmnI, BCL11A, and HBS1L-MYB on clinical appearances: A study on Î²-thalassemia and hemoglobin E/Î²-thalassemia patients in Indonesia.	Rujito L, Basalamah M, Siswandari W, Setyono J, Wulandari G, Mulatsih S, Sofro AS, Sadewa AH, Sutaryo S.	Hematol Oncol Stem Cell Ther. 2016 Jun;9(2):55-63. doi: 10.1016/j.hemonc.2016.02.003.
Efficacy and safety of iron-chelation therapy with deferoxamine, deferiprone, and deferasirox for the treatment of iron-loaded patients with non-transfusion-dependent thalassemia syndromes.	Kontoghiorghe CN, Kontoghiorghes GJ.	Drug Des Devel Ther. 2016 Jan 29;10:465-81. doi: 10.2147/DDDT.S79458. Review.
EVALUATION OF RED BLOOD CELL INDICES RELATED DISORDERS AMONG ELIGIBLE BLOOD DONORS AT THE UNIVERSITI PUTRA MALAYSIA (UPM).	Riahi S, Mei IL, Idris FB, George E, Noor SM.	Southeast Asian J Trop Med Public Health. 2015 Sep;46(5):911-7.

Hemoglobin E Prevalence among Ethnic Groups Residing in Malaria-Endemic Areas of Northern Thailand and Its Lack of Association with Plasmodium falciparum Invasion In Vitro.	Lithanatudom P, Wipasa J, Inti P, Chawansuntati K, Svasti S, Fucharoen S, Kangwanpong D, Kampuansai J.	PLoS One. 2016 Jan 25;11(1):e0148079. doi: 10.1371/journal.pone.0148079. Erratum in: PLoS One. 2016;11(9):e0163430.
Hb lepore/Î²0-thalassaemia with Î±+-thalassaemia interactions, a potential diagnostic pitfall.	Alauddin H, Mohamad Nasir S, Ahadon M, Raja Sabudin RZ, Ithnin A, Hussin NH, Alias H, Loh CK, Abdul Latiff Z, Abdul Murad NA, Othman A.	Malays J Pathol. 2015 Dec;37(3):287-92.
Î²-Thalassemia Haplotypes in Romania in the Context of Genetic Mixing in the Mediterranean Area.	Cherry L, Calo C, Talmaci R, Perrin P, Gavrila L.	Hemoglobin. 2016;40(2):85-96. doi: 10.3109/03630269.2015.1124113. Review.
Hemoglobin Constant Spring among Southeast Asian Populations: Haplotypic Heterogeneities and Phylogenetic Analysis.	Jomoui W, Fucharoen G, Sanchaisuriya K, Nguyen VH, Fucharoen S.	PLoS One. 2015 Dec 18;10(12):e0145230. doi: 10.1371/journal.pone.0145230.
Hemoglobin Variants in Northern Thailand: Prevalence, Heterogeneity and Molecular Characteristics.	Panyasai S, Fucharoen G, Fucharoen S.	Genet Test Mol Biomarkers. 2016 Jan;20(1):37-43. doi: 10.1089/gtmb.2015.0182.
DETECTION OF DELETION Î±(+)-THALASSEMIA MUTATION [-Î± (3.7), -Î± (4.2)] BY QUANTITATIVE PCR ASSAY.	Seeratanachot T, Shimbhu D, Charoenkwan P, Sanguansermsri T.	Southeast Asian J Trop Med Public Health. 2015 Jan;46(1):110-5.
The Homozygous Hemoglobin EE Genotype and Chronic Inflammation Are Associated with High Serum Ferritin and Soluble Transferrin Receptor Concentrations among Women in Rural Cambodia.	Karakochuk CD, Whitfield KC, Rappaport AI, Barr SI, Vercauteren SM, McLean J, Prak S, Hou K, Talukder A, Devenish R, Green TJ.	J Nutr. 2015 Dec;145(12):2765-73. doi: 10.3945/jn.115.218636.
Pregnancy outcomes among women with beta-thalassemia trait.	Charoenboon C, Jatavan P, Traisrisilp K, Tongsong T.	Arch Gynecol Obstet. 2016 Apr;293(4):771-4. doi: 10.1007/s00404-015-3908-6.
Molecular Heterogeneity of Thalassemia among Pregnant Laotian Women.	Wongprachum K, Sanchaisuriya K, Dethvongphanh M, Norcharoen B, Htalongsengchan B, Vidamaly V, Sanchaisuriya P, Fucharoen S, Fucharoen G, Schelp FP.	Acta Haematol. 2016;135(2):65-9. doi: 10.1159/000438739.
Mutation Spectrum of Î²-Thalassemia and Other Hemoglobinopathies in Chittagong, Southeast Bangladesh.	Chatterjee T, Chakravarty A, Chakravarty S, Chowdhury MA, Sultana R.	Hemoglobin. 2015;39(6):389-92. doi: 10.3109/03630269.2015.1078810.
Correlation of BACH1 and Hemoglobin E/Beta-Thalassemia Globin Expression.	Lee TY, Muniandy L, Teh LK, Abdullah M, George E, Sathar J, Lai MI.	Turk J Haematol. 2016 Mar 5;33(1):15-20. doi: 10.4274/tjh.2014.0197.
A novel gap-PCR with high resolution melting analysis for the detection of Î±-thalassaemia Southeast Asian and Filipino Î²Â°-thalassaemia deletion.	Kho SL, Chua KH, George E, Tan JA.	Sci Rep. 2015 Sep 14;5:13937. doi: 10.1038/srep13937.

Severe bacterial infections in patients with non-transfusion-dependent thalassemia: prevalence and clinical risk factors.	Teawtrakul N, Jetsrisuparb A, Sirijerachai C, Chansung K, Wanitpongpun C.	Int J Infect Dis. 2015 Oct;39:53-6. doi: 10.1016/j.ijid.2015.09.001.
Phenotype and Genotype in a Cohort of 312 Adult Patients with Nontransfusion-Dependent Thalassemia in Northeast Thailand.	Prayalaw P, Teawtrakul N, Jetsrisuparb A, Pongudom S, Fucharoen G, Fucharoen S.	Acta Haematol. 2016;135(1):15-20. doi: 10.1159/000435802.
Molecular Scanning of Î²-Thalassemia in the Southern Region of Central Java, Indonesia; a Step Towards a Local Prevention Program.	Rujito L, Basalamah M, Mulatsih S, Sofro AS.	Hemoglobin. 2015;39(5):330-3. doi: 10.3109/03630269.2015.1065420.
The Spectrum of Î±-Thalassemia Mutations in Kermanshah Province, West Iran.	Alibakhshi R, Mehrabi M, Omidniakan L, Shafieenia S.	Hemoglobin. 2015;39(6):403-6. doi: 10.3109/03630269.2015.1070732.
Psychological Aspects in Children and Adolescents With Major Thalassemia: A Case-Control Study.	Behdani F, Badiee Z, Hebrani P, Moharreri F, Badiee AH, Hajivosugh N, Rostami Z, Akhavanrezayat A.	Iran J Pediatr. 2015 Jun;25(3):e322. doi: 10.5812/ijp.25(3)2015.322.
Coexistence of Malaria and Thalassemia in Malaria Endemic Areas of Thailand.	Kuesap J, Chaijaroenkul W, Rungsihirunrat K, Pongjantharasatien K, Na-Bangchang K.	Korean J Parasitol. 2015 Jun;53(3):265-70. doi: 10.3347/kjp.2015.53.3.265.
The Prevalence of Hemoglobinopathies in Young Adolescents in the Province of MuÄŸla in Turkey: Results of a Screening Program.	Topal Y, Topal H, Ceyhan MN, Azik F, Ã‡apanoÄŸlu M, KocabaÅŸ CN.	Hemoglobin. 2015;39(4):247-50. doi: 10.3109/03630269.2015.1046185.
Interaction of Hb Grey Lynn (Vientiane) [Î±91(FG3)Leu>Phe (Î±1)] with Hb E [Î²26(B8) Glu>Lys] and Î±(+)-thalassemia: Molecular and Hematological Analysis.	Singha K, Fucharoen G, Fucharoen S.	Clin Lab. 2015;61(5-6):631-5.
Health-Related Quality of Life in Adolescents with Thalassemia.	Boonchooduang N, Louthrenoo O, Choeyprasert W, Charoenkwan P.	Pediatr Hematol Oncol. 2015;32(5):341-8. doi: 10.3109/08880018.2015.1033795.
Treatment of Î²-Thalassemia/Hemoglobin E with Antioxidant Cocktails Results in Decreased Oxidative Stress, Increased Hemoglobin Concentration, and Improvement of the Hypercoagulable State.	Yanpanitch OU, Hatairaktham S, Charoensakdi R, Panichkul N, Fucharoen S, Srichairatanakool S, Siritanaratkul N, Kalpravidh RW.	Oxid Med Cell Longev. 2015;2015:537954. doi: 10.1155/2015/537954.
Red Cell Indices and Formulas Used in Differentiation of Î²-Thalassemia Trait from Iron Deficiency in Thai Adults.	Plengsuree S, Punyamung M, Yanola J, Nanta S, Jaiping K, Maneewong K, Wongwiwatthananukit S, Pornprasert S.	Hemoglobin. 2015;39(4):235-9. doi: 10.3109/03630269.2015.1048352.
A family empowerment program for families having children with thalassemia, Thailand.	Wacharasin C, Phaktoop M, Sananreangsak S.	Nurs Health Sci. 2015 Sep;17(3):387-94. doi: 10.1111/nhs.12201.

Molecular spectrum of Î±-globin gene mutations in the Aegean region of Turkey: first observation of three Î±-globin gene mutations in the Turkish population.	Onay H, Aykut A, Karaca E, Durmaz A, Solmaz AE, Ã‡oÄŸulu Ã–, AydÄ±nok Y, Vergin C, Ã–zkÄ±nay F.	Int J Hematol. 2015 Jul;102(1):1-6. doi: 10.1007/s12185-015-1796-y.
Examining the usefulness of a Family Empowerment Program guided by the Illness Beliefs Model for families caring for a child with thalassemia.	Wacharasin C, Phaktoop M, Sananreangsak S.	J Fam Nurs. 2015 May;21(2):295-321. doi: 10.1177/1074840715585000.
Implementation of newborn screening for hemoglobin h disease in mainland china.	Xie XM, Zhou JY, Li J, Li R, Liao C, Li DZ.	Indian J Hematol Blood Transfus. 2015 Jun;31(2):242-6. doi: 10.1007/s12288-014-0432-y.
Thalassemia major is a major risk factor for pediatric melioidosis in Kota Kinabalu, Sabah, Malaysia.	Fong SM, Wong KJ, Fukushima M, Yeo TW.	Clin Infect Dis. 2015 Jun 15;60(12):1802-7. doi: 10.1093/cid/civ189.
Wandering spleen with chronic torsion in a patient with thalassaemia.	Ho CL.	Singapore Med J. 2014 Dec;55(12):e198-200.
Sonographic markers of fetal Î±-thalassemia major.	Li X, Zhou Q, Zhang M, Tian X, Zhao Y.	J Ultrasound Med. 2015 Feb;34(2):197-206. doi: 10.7863/ultra.34.2.197. Review.
Genetic hemoglobin disorders rather than iron deficiency are a major predictor of hemoglobin concentration in women of reproductive age in rural prey Veng, Cambodia.	Karakochuk CD, Whitfield KC, Barr SI, Lamers Y, Devlin AM, Vercauteren SM, Kroeun H, Talukder A, McLean J, Green TJ.	J Nutr. 2015 Jan;145(1):134-42. doi: 10.3945/jn.114.198945.
High resolution melting analytical platform for rapid prenatal and postnatal diagnosis of Î²-thalassemia common among Southeast Asian population.	Prajantasen T, Fucharoen S, Fucharoen G.	Clin Chim Acta. 2015 Feb 20;441:56-62. doi: 10.1016/j.cca.2014.12.015.
Î±-thalassemia-associated hydrops fetalis: A rare cause of thyrotoxic cardiomyopathy.	Tumian NR, Wong M, Wong CL.	J Obstet Gynaecol Res. 2015 Jun;41(6):967-70. doi: 10.1111/jog.12648.
A large cohort of Î²(+)-thalassemia in Thailand: molecular, hematological and diagnostic considerations.	Yamsri S, Singha K, Prajantasen T, Taweenan W, Fucharoen G, Sanchaisuriya K, Fucharoen S.	Blood Cells Mol Dis. 2015 Feb;54(2):164-9. doi: 10.1016/j.bcmd.2014.11.008.
Craniofacial manifestations of Î²-thalassemia major.	Javid B, Said-Al-Naief N.	Oral Surg Oral Med Oral Pathol Oral Radiol. 2015 Jan;119(1):e33-40. doi: 10.1016/j.oooo.2014.08.020.
Prevalence of anemia, iron deficiency, thalassemia and glucose-6-phosphate dehydrogenase deficiency among hill-tribe school children in Omkoi District, Chiang Mai Province, Thailand.	Yanola J, Kongpan C, Pornprasert S.	Southeast Asian J Trop Med Public Health. 2014 Jul;45(4):920-5.
Transfusion-dependent thalassemia in Northern Sarawak: a molecular study to identify different genotypes	Tan JA, Chin SS, Ong GB, Mohamed Unni MN, Soosay AE, Gudum HR, Kho SL, Chua KH, Chen JJ, George E.	Public Health Genomics. 2015;18(1):60-4. doi: 10.1159/000368342.

in the multi-ethnic groups and the importance of genomic sequencing in unstudied populations.		
Immune response of thalassemia major patients in Indonesia with and without splenectomy.	Sari TT, Gatot D, Akib AA, Bardosono S, Hadinegoro SR, Harahap AR, Idjradinata PS.	Acta Med Indones. 2014 Jul;46(3):217-25.
Pakistani children's experiences of growing up with beta-thalassemia major.	Mufti GE, Towell T, Cartwright T.	Qual Health Res. 2015 Mar;25(3):386-96. doi: 10.1177/1049732314552663.
The correlation of Î±-globin gene mutations and the XmnI polymorphism with clinical severity of Hb E/Î²-thalassemia.	Charoenkwan P, Teerachaimahit P, Sanguansermsri T.	Hemoglobin. 2014;38(5):335-8. doi: 10.3109/03630269.2014.952744.
Known and new hemoglobin A2 variants in Thailand and implication for Î²-thalassemia screening.	Panyasai S, Fucharoen G, Fucharoen S.	Clin Chim Acta. 2015 Jan 1;438:226-30. doi: 10.1016/j.cca.2014.09.003.
ARKRAY ADAMS A1c HA-8180T Analyzer for Diagnosis of Thalassemia and Hemoglobinopathies Common in Southeast Asia.	Kunwandee J, Srivorakun H, Fucharoen G, Sanchaisuriya K, Fucharoen S.	Lab Med. 2014 Summer;45(3):e112-21. doi: 10.1309/LMMH649POETQREXL.
Hb Cibeles [Î±2 CD25(B6) (Gly â†’ Asp)]: a novel alpha chain variant causing alpha-thalassemia.	de la Fuente-Gonzalo F, Nieto JM, Vinuesa L, Sevilla J, DÃaz-Mediavilla J, Villegas A, GonzÃ¡lez FA, Ropero P.	Int J Hematol. 2014 Dec;100(6):599-601. doi: 10.1007/s12185-014-1663-2.
A single centre study on birth of children with transfusion-dependent thalassaemia in Malaysia and reasons for ineffective prevention.	Ngim CF, Ibrahim H, Lai NM, Ng CS.	Prenat Diagn. 2015 Jan;35(1):51-9. doi: 10.1002/pd.4484.
Hemoglobin Q-Thailand and its combinations with other forms of thalassemia or hemoglobinopathies in northern Thailand.	Panyasai S, Pornprasert S.	Clin Lab. 2014;60(7):1099-103.
The associations of SEA-alpha thalassemia 1, XmnI-Ggamma polymorphism and beta-globin gene mutations with the clinical severity of beta-thalassemia syndrome in northern Thailand.	Tatu T, Sritong W, Sa-Nguansermsri T.	J Med Assoc Thai. 2014 Mar;97(3):300-7.
Copy number variation in Thai population.	Suktitipat B, Naktang C, Mhuantong W, Tularak T, Artiwet P, Pasomsap E, Jongjaroenprasert W, Fuchareon S, Mahasirimongkol S, Chantratita W, Yimwadsana B, Charoensawan V, Jinawath N.	PLoS One. 2014 Aug 13;9(8):e104355. doi: 10.1371/journal.pone.0104355.
Incidence of ototoxicity in pediatric patients with transfusion-dependent thalassemia who are less well-chelated by mono- and combined therapy of iron chelating agents.	Tanphaichitr A, Kusuwan T, Limviriyakul S, Atipas S, Pooliam J, Sangpraypan T, Tanphaichitr VS, Viprakasit V.	Hemoglobin. 2014;38(5):345-50. doi: 10.3109/03630269.2014.940462.

Haemoglobin E-beta Thalassaemia in Singapore.	Tan ES, Koh C, Law HY, Tan GP, Lai AH, Ng IS.	Ann Acad Med Singapore. 2014 Jun;43(6):331-3. No abstract available.
Application of HTA research on policy decision-making.	Youngkong S.	J Med Assoc Thai. 2014 May;97 Suppl 5:S119-26.
A 65 bp deletion in band 3 gene of beta-thalassemia patients in Indonesia.	Dewajanthi AM, Lubis VK, Wanandi SI, Gatot D, Soegianto RR, Freisleben SK, Wahidiyat I, Freisleben HJ.	Southeast Asian J Trop Med Public Health. 2014 Jan;45(1):183-6.
New mathematical formula for differentiating thalassemia trait and iron deficiency anemia in thalassemia prevalent area: a study in healthy school-age children.	Sirachainan N, Iamsirirak P, Charoenkwan P, Kadegasem P, Wongwerawattanakoon P, Sasanakul W, Chansatitporn N, Chuansumrit A.	Southeast Asian J Trop Med Public Health. 2014 Jan;45(1):174-82.
Molecular characterization of Î±- and Î²-thalassaemia among Malay patients.	Yatim NF, Rahim MA, Menon K, Al-Hassan FM, Ahmad R, Manocha AB, Saleem M, Yahaya BH.	Int J Mol Sci. 2014 May 19;15(5):8835-45. doi: 10.3390/ijms15058835.
A case series of Î±-thalassemia intermedia due to compound heterozygosity for Hb Adana [HBA2: c179G>A (or HBA1); p.Gly60Asp] with other Î±-thalassemias in Malay families.	Alauddin H, Jaapar NA, Azma RZ, Ithnin A, Razak NF, Loh CK, Alias H, Abdul-Latiff Z, Othman A.	Hemoglobin. 2014;38(4):277-81. doi: 10.3109/03630269.2014.916720.
Molecular characteristic of alpha thalassaemia among patients diagnosed in UKM Medical Centre.	Azma RZ, Ainoon O, Hafiza A, Azlin I, Noor Farisah AR, Nor Hidayati S, Noor Hamidah H.	Malays J Pathol. 2014 Apr;36(1):27-32.
The use of Taqman genotyping assays for rapid confirmation of Î²-thalassaemia mutations in the Malays: accurate diagnosis with low DNA concentrations.	Teh LK, Lee TY, Tan JA, Lai MI, George E.	Int J Lab Hematol. 2015 Feb;37(1):79-89. doi: 10.1111/ijlh.12240.
Interaction of hemoglobin Grey Lynn (Vientiane) with a non-deletional Î±(+)-thalassemia in an adult Thai proband.	Singha K, Fucharoen G, Fucharoen S.	Biochem Med (Zagreb). 2014 Feb 15;24(1):167-74. doi: 10.11613/BM.2014.019.
Genotyping of beta thalassemia trait by high-resolution DNA melting analysis.	Saetung R, Ongchai S, Charoenkwan P, Sanguansermsri T.	Southeast Asian J Trop Med Public Health. 2013 Nov;44(6):1055-64.
Correlation between T2* cardiovascular magnetic resonance with left ventricular function and mass in adolescent and adult major thalassemia patients with iron overload.	Djer MM, Anggriawan SL, Gatot D, Amalia P, Sastroasmoro S, Widjaja P.	Acta Med Indones. 2013 Oct;45(4):295-301.
Criteria for detection of alpha-thalassemia-1 Thai type deletion in routine laboratory.	Pornprasert S, Punyamung M, Treesuwan K.	Clin Lab. 2013;59(11-12):1423-7.
Effect of genotype on pulmonary hypertension risk in patients with thalassemia.	Teawtrakul N, Ungprasert P, Pussadhamma B, Prayalaw P, Fucharoen S, Jetsrisuparb A, Pongudom S, Sirijerachai C,	Eur J Haematol. 2014;92(5):429-34. doi: 10.1111/ejh.12261.

	Chansung K, Wanitpongpun C, Chuncharunee S.	
Molecular basis of transfusion dependent beta-thalassemia major patients in Sabah.	Teh LK, George E, Lai MI, Tan JA, Wong L, Ismail P.	J Hum Genet. 2014 Mar;59(3):119-23. doi: 10.1038/jhg.2013.131.
Hemoglobin Constant Spring is markedly high in women of an ethnic minority group in Vietnam: a community-based survey and hematologic features.	Nguyen VH, Sanchaisuriya K, Wongprachum K, Nguyen MD, Phan TT, Vo VT, Sanchaisuriya P, Fucharoen S, Schelp FP.	Blood Cells Mol Dis. 2014 Apr;52(4):161-5. doi: 10.1016/j.bcmd.2013.12.002.
A newly modified hemoglobin H inclusion test as a secondary screening for Î±(0)-thalassemia in Southeast Asian populations.	Fucharoen G, Yooyen K, Chaibunruang A, Fucharoen S.	Acta Haematol. 2014;132(1):10-4. doi: 10.1159/000355187.
Severe Î±-thalassemia intermedia due to a compound heterozygosity for the highly unstable Hb Adana (HBA2: c.179G>A) and a novel codon 24 (HBA2: c.75T>A) mutation.	Megawati D, Nainggolan IM, Swastika M, Susanah S, Mose JC, Harahap AR, Setianingsih I.	Hemoglobin. 2014;38(2):149-51. doi: 10.3109/03630269.2013.863206.
Massive hemothorax due to intrathoracic extramedullary hematopoiesis in a patient with beta thalassemia hemoglobin E disease.	Tantraworasin A, Saeteng S.	J Med Assoc Thai. 2013 Jul;96(7):866-9.
Genetic heterogeneity of hemoglobin AEBart's disease: a large cohort data from a single referral center in northeast Thailand.	Chaibunruang A, Karnpean R, Fucharoen G, Fucharoen S.	Blood Cells Mol Dis. 2014 Apr;52(4):176-80. doi: 10.1016/j.bcmd.2013.11.006.
A simple and highly sensitive ELISA for screening of the Î±-thalassemia-1 Southeast Asian-type deletion.	Pata S, Khummuang S, Pornprasert S, Tatu T, Kasinrerk W.	J Immunoassay Immunochem. 2014;35(2):194-206. doi: 10.1080/15321819.2013.838963.
Applying molecular immunohaematology to regularly transfused thalassaemic patients in Thailand.	Rujirojindakul P, Flegel WA.	Blood Transfus. 2014 Jan;12(1):28-35. doi: 10.2450/2013.0058-13.
Phenotypic expression of Hb F in common high Hb F determinants in Thailand: roles of Î±-thalassemia, 5' Î´-globin BCL11A binding region and 3' Î²-globin enhancer.	Prakobkaew N, Fucharoen S, Fuchareon G, Siriratmanawong N.	Eur J Haematol. 2014 Jan;92(1):73-9. doi: 10.1111/ejh.12201.
Diagnostic utility of isoelectric focusing and high performance liquid chromatography in neonatal cord blood screening for thalassemia and non-sickling hemoglobinopathies.	Uaprasert N, Settapiboon R, Amornsiriwat S, Sarnthammakul P, Thanapat T, Rojnuckarin P, Sutcharitchan P.	Clin Chim Acta. 2014 Jan 1;427:23-6. doi: 10.1016/j.cca.2013.09.041.
Five hemoglobin variants in a double heterozygote for Î±- and Î²-globin chain defects.	Singha K, Fucharoen G, Fucharoen S.	Acta Haematol. 2014;131(2):71-5. doi: 10.1159/000353123.
Nonimmune hydrops fetalis in a children's hospital: a six-year series.	Ng ZM, Seet MJ, Erng MN, Buendia F, Chang AS, Sriram B.	Singapore Med J. 2013 Sep;54(9):487-90.

Distribution of alpha thalassaemia gene variants in diverse ethnic populations in malaysia: data from the institute for medical research.	Ahmad R, Saleem M, Aloysious NS, Yelumalai P, Mohamed N, Hassan S.	Int J Mol Sci. 2013 Sep 10;14(9):18599-614. doi: 10.3390/ijms140918599.
Counseling for prenatal diagnosis and termination of pregnancy due to thalassemia major: a survey of health care workers' practices in Malaysia.	Ngim CF, Lai NM, Ibrahim H.	Prenat Diagn. 2013 Dec;33(13):1226-32. doi: 10.1002/pd.4233.
Low fetal hemoglobin rates in patients carrying Thai (Î´Î²)0-deletion and Turkish (Î´Î²)0-deletion/inversion strengthen the hypothesis that the 5'Î´ BCL11A binding site plays a major role in its fetal hemoglobin inhibitory regulation. Response to "The 12.6 kb-deletion in the Î²-globin gene cluster is the ...	Ghedira ES, Pissard S.	Haematologica. 2013 Sep;98(9):e119-20. doi: 10.3324/haematol.2013.093716. No abstract available.
The 12.6 kb-deletion in the Î²-globin gene cluster is the known Thai/Vietnamese (Î´Î²)0-thalassemia commonly found in Southeast Asia.	Chalaow N, Thein SL, Viprakasit V.	Haematologica. 2013 Sep;98(9):e117-8. doi: 10.3324/haematol.2013.090613. No abstract available.
Burden of anemia in relation to thalassemia and iron deficiency among Vietnamese pregnant women.	Siridamrongvattana S, Van Hoa N, Sanchaisuriya K, Dung N, Hoa PT, Sanchaisuriya P, Fucharoen G, Fucharoen S, Schelp FP.	Acta Haematol. 2013;130(4):281-7. doi: 10.1159/000351168.
Fetal cardiac circumference derived by spatiotemporal image correlation as a predictor of fetal hemoglobin Bart disease at midpregnancy.	Siwawong W, Tongprasert F, Srisupundit K, Luewan S, Tongsong T.	J Ultrasound Med. 2013 Aug;32(8):1483-8. doi: 10.7863/ultra.32.8.1483.
Rapid prenatal diagnosis of common beta-thalassemia mutations in Southeast Asia using pyrosequencing.	Ho SS, Huan PT, Leow GH, Ching LK, Chiu L, Law HY, Koay ES.	Prenat Diagn. 2013 Nov;33(11):1017-22. doi: 10.1002/pd.4183.
Distribution of alpha thalassaemia in 16 year old Malaysian Students in Penang, Melaka and Sabah.	Rahimah AN, Nisha S, Safiah B, Roshida H, Punithawathy Y, Nurul H, Syahzuwan H, Zubaidah Z.	Med J Malaysia. 2012 Dec;67(6):565-70.
Children with hemoglobin E/Î²-thalassemia have a high risk of being vitamin D deficient even if they get abundant sun exposure: a study from Thailand.	Nakavachara P, Viprakasit V.	Pediatr Blood Cancer. 2013 Oct;60(10):1683-8. doi: 10.1002/pbc.24614.
Gonadal function of beta-thalassemics following stem cell transplantation conditioned with myeloablative and reduced intensity regimens.	Poomthavorn P, Chawalitdamrong P, Hongeng S, Mahachoklertwattana P, Pakakasama S, Khlairit P, Chailurkit LO.	J Pediatr Endocrinol Metab. 2013;26(9-10):925-32. doi: 10.1515/jpem-2012-0399.
Pregnancy outcomes amongst thalassemia traits.	Hanprasertpong T, Kor-anantakul O, Leetanaporn R, Suntharasaj T, Suwanrath C, Pruksanusak N, Pranpanus S.	Arch Gynecol Obstet. 2013 Nov;288(5):1051-4. doi: 10.1007/s00404-013-2886-9.

Molecular and hematological studies in a large cohort of Î±(0)-thalassemia in northeast Thailand: data from a single referral center.	Chaibunruang A, Prommetta S, Yamsri S, Fucharoen G, Sae-Ung N, Sanchaisuriya K, Fucharoen S.	Blood Cells Mol Dis. 2013 Aug;51(2):89-93. doi: 10.1016/j.bcmd.2013.04.003.
Adrenal insufficiency is prevalent in HbE/Î²-thalassaemia paediatric patients irrespective of their clinical severity and transfusion requirement.	Nakavachara P, Viprakasit V.	Clin Endocrinol (Oxf). 2013 Dec;79(6):776-83. doi: 10.1111/cen.12235.
Thalassemia and hemoglobinopathies in Thua Thien Hue Province, Central Vietnam.	Nguyen HV, Sanchaisuriya K, Nguyen D, Phan HT, Siridamrongvattana S, Sanchaisuriya P, Fucharoen S, Fucharoen G, Schelp FP.	Hemoglobin. 2013;37(4):333-42. doi: 10.3109/03630269.2013.790829.
Genetic compound heterozygosity for Southeast Asian ovalocytosis and thalassemia in Thailand: prevalence and phenotypic analysis.	Ngouprommin L, Sae-Ung N, Fucharoen S, Fucharoen G, Sanchaisuriya K, Jetsrisuparb A.	Clin Genet. 2014 Feb;85(2):198-200. doi: 10.1111/cge.12128. No abstract available.
Problems in determining thalassemia carrier status in a program for prevention and control of severe thalassemia syndromes: a lesson from Thailand.	Viprakasit V, Limwongse C, Sukpanichnant S, Ruangvutilert P, Kanjanakorn C, Glomglao W, Sirikong M, Utto W, Tanphaichitr VS.	Clin Chem Lab Med. 2013 Aug;51(8):1605-14. doi: 10.1515/cclm-2013-0098.
Unrecognized vertebral fractures in adolescents and young adults with thalassemia syndromes.	Engkakul P, Mahachoklertwattana P, Jaovisidha S, Chuansumrit A, Poomthavorn P, Chitrapazt N, Chuncharunee S.	J Pediatr Hematol Oncol. 2013 Apr;35(3):212-7. doi: 10.1097/MPH.0b013e3182841574.
Evidence of recent natural selection on the Southeast Asian deletion (--(SEA)) causing Î±-thalassemia in South China.	Qiu QW, Wu DD, Yu LH, Yan TZ, Zhang W, Li ZT, Liu YH, Zhang YP, Xu XM.	BMC Evol Biol. 2013 Mar 11;13:63. doi: 10.1186/1471-2148-13-63.
Clinical course of dengue in patients with thalassaemia.	Natesirinilkul R, Tantiworawit A, Charoenkwan P.	Paediatr Int Child Health. 2013 Feb;33(1):32-6. doi: 10.1179/2046905512Y.0000000020.
Specific and straightforward molecular investigation of Î²-thalassemia mutations in the Malaysian Malays and Chinese using direct TaqMan genotyping assays.	Kho SL, Chua KH, George E, Tan JA.	Genet Mol Res. 2013 Jul 15;12(3):2409-15. doi: 10.4238/2013.February.28.4.
Deferiprone (GPO-L-ONE(Â®)) monotherapy reduces iron overload in transfusion-dependent thalassemias: 1-year results from a multicenter prospective, single arm, open label, dose escalating phase III pediatric study (GPO-L-ONE; A001) from Thailand.	Viprakasit V, Nuchprayoon I, Chuansumrit A, Torcharus K, Pongtanakul B, Laothamatas J, Srichairatanakool S, Pooliam J, Supajitkasem S, Suriyaphol P, Tanphaichitr VS, Tuchinda S.	Am J Hematol. 2013 Apr;88(4):251-60. doi: 10.1002/ajh.23386.
Hemoglobin Pyrgos with hemoglobin H disease: new triple heterozygosity.	Insiripong S, Jitpakdeebodin V, Jopang Y, Fucharoen S.	Southeast Asian J Trop Med Public Health. 2012 Sep;43(5):1258-61.

HbA2 levels in normal, beta-thalassaemia and haemoglobin E carriers by capillary electrophoresis.	Hafiza A, Malisa MY, Khirotdin RD, Azlin I, Azma Z, Thong MC, Ali IM, Yeoh ZN, Mohd Ishak L, Mohd Radzi NR, Hussin NH.	Malays J Pathol. 2012 Dec;34(2):161-4.
Low cost biosensor-based molecular differential diagnosis of Î±-thalassemia (Southeast Asia deletion).	Wangmaung N, Promptmas C, Chomean S, Sanchomphu C, Ittarat W.	Clin Chem Lab Med. 2013 Jun;51(6):1199-205. doi: 10.1515/cclm-2012-0732.
Cost utility analysis of reduced intensity hematopoietic stem cell transplantation in adolescence and young adult with severe thalassemia compared to hypertransfusion and iron chelation program.	Sruamsiri R, Chaiyakunapruk N, Pakakasama S, Sirireung S, Sripaiboonkij N, Bunworasate U, Hongeng S.	BMC Health Serv Res. 2013 Feb 5;13:45. doi: 10.1186/1472-6963-13-45.
The prevention of thalassemia.	Cao A, Kan YW.	Cold Spring Harb Perspect Med. 2013 Feb 1;3(2):a011775. doi: 10.1101/cshperspect.a011775. Review.
Evidence of differential selection for the -Î±(3.7) and -Î±(4.2) single-Î±-globin gene deletions within the same population.	Yap ZM, Sun KM, Teo CR, Tan AS, Chong SS.	Eur J Haematol. 2013 Mar;90(3):210-3. doi: 10.1111/ejh.12058.
Prenatal and post-natal screening of Î²-thalassemia and hemoglobin E genes in Thailand using denaturing high performance liquid chromatography.	Prajantasen T, Fucharoen S, Fucharoen G, Siriratmanawong N, Pinmuang-Ngam C.	Mol Biol Rep. 2013 Apr;40(4):3173-9. doi: 10.1007/s11033-012-2391-4.
A proficiency testing program of hemoglobin analysis in prevention and control of severe hemoglobinopathies in Thailand.	Karnpean R, Fucharoen G, Pansuwan A, Changtrakul D, Fucharoen S.	Clin Chem Lab Med. 2013 Jun;51(6):1265-71. doi: 10.1515/cclm-2012-0588.
Detection of coinherited Hb H-Constant Spring/PaksÃ© disease and Hb E by capillary electrophoresis and high performance liquid chromatography.	Pornprasert S, Waneesorn J.	Hemoglobin. 2013;37(2):176-82. doi: 10.3109/03630269.2012.752744.
HbA2 levels in Î²-thalassaemia carriers with the Filipino Î²0-deletion: are the levels higher than what is found with non-deletional forms of Î²0-thalassaemia?	George E, Teh LK, Tan J, Lai MI, Wong L.	Pathology. 2013 Jan;45(1):62-5. doi: 10.1097/PAT.0b013e32835af7c1.
Association of Hb Thailand [Î±56(E5)Lysâ†'Thr] and Hb Phnom Penh [Î±117(GH5)-Ile-Î±118(H1)] with Î±(0)-thalassemia: molecular and hematological features and differential diagnosis.	Singha K, Srivorakun H, Fucharoen G, Changtrakul Y, Komwilaisak P, Jetsrisuparb A, Puangplruk R, Fucharoen S.	Hemoglobin. 2013;37(1):37-47. doi: 10.3109/03630269.2012.747964.
A single-tube multiplex gap-polymerase chain reaction for the detection of eight Î²-globin gene	Tritipsombut J, Phylipsen M, Viprakasit V, Chalaow N, Sanchaisuriya K, Giordano PC, Fucharoen S, Harteveld CL.	Hemoglobin. 2012;36(6):571-80. doi: 10.3109/03630269.2012.747441.

cluster deletions common in Southeast Asia.		
The impact and disease burden of thalassemia in Thailand: a population-based study in 2010.	Teawtrakul N, Chansung K, Sirijerachai C, Wanitpongpun C, Thepsuthammarat K.	J Med Assoc Thai. 2012 Jul;95 Suppl 7:S211-6.
Identification of hemoglobin variants in samples received for glycated hemoglobin testing.	Saw S, Loh TP, Yin C, Sethi SK.	Clin Chim Acta. 2013 Jan 16;415:173-5. doi: 10.1016/j.cca.2012.10.043.
Hematopoietic cell transplantation for thalassemia: a global perspective BMT tandem meeting 2013.	Mehta PA, Faulkner LB.	Biol Blood Marrow Transplant. 2013 Jan;19(1 Suppl):S70-3. doi: 10.1016/j.bbmt.2012.10.025. Review.
Dengue infection in hematologic-oncologic pediatric patients: aggravation of anemia and bleeding risk.	Chuansumrit A, Tangnararatchakit K, Sirachainan N, Pakakasamal S, Hongeng S, Chaiyaratana W, Kitpoka P, Yoksan S.	Southeast Asian J Trop Med Public Health. 2012 Mar;43(2):311-22.
Predictors of family management behavior for children with thalassemia.	Sananreangsak S, Lapvongwatanal P, Virutsetazin K, Vatanasomboon P, Gaylord N.	Southeast Asian J Trop Med Public Health. 2012 Jan;43(1):160-71.
Obstructive sleep apnea among children with severe beta-thalassemia.	Sritippayawan S, Norasetthekul S, Nuchprayoon I, Deerojanawong J, Desudchit T, Prapphal N.	Southeast Asian J Trop Med Public Health. 2012 Jan;43(1):152-9.
Prevalence of iron deficiency anaemia and thalassaemia trait among undergraduate medical students.	Azma RZ, Ainoon O, Azlin I, Hamenuddin H, Hadi NA, Tatt WK, Syazana IN, Asmaliza AM, Das S, Hamidah NH.	Clin Ter. 2012 Jul;163(4):287-91.
Artemisinin-resistant Plasmodium falciparum in Pursat province, western Cambodia: a parasite clearance rate study.	Amaratunga C, Sreng S, Suon S, Phelps ES, Stepniewska K, Lim P, Zhou C, Mao S, Anderson JM, Lindegardh N, Jiang H, Song J, Su XZ, White NJ, Dondorp AM, Anderson TJ, Fay MP, Mu J, Duong S, Fairhurst RM.	Lancet Infect Dis. 2012 Nov;12(11):851-8. doi: 10.1016/S1473-3099(12)70181-0.
Causes and attitude of husbands toward thalassemic carrier screening test when their partners have abnormal screening tests.	Vuthiwong C, Panichkul P, Punpuckdeekoon P.	J Med Assoc Thai. 2012 May;95 Suppl 5:S162-7.
Prevalence and hematological parameters of thalassemia in Tha Kradarn subdistrict Chachoengsao Province, Thailand.	Nillakupt K, Nathalang O, Arnutti P, Jindadamrongwech S, Boonsiri T, Panichkul S, Areekul W.	J Med Assoc Thai. 2012 May;95 Suppl 5:S124-32.
Unmasking Hb PaksÃ© (codon 142, TAA>TAT, Î±2) and its combinations in patients also carrying Hb Constant Spring (codon 142, TAA>CAA, Î±2) in northern Thailand.	Pornprasert S, Panyasai S, Treesuwan K.	Hemoglobin. 2012;36(5):491-6.
Prevalence of anemia and underlying iron status in naive antiretroviral therapy HIV-infected children with moderate immune suppression.	Kosalaraksa P, Bunupuradah T, Vonthanak S, Wiangnon S, Hansudewechakul R, Vibol U, Kanjanavanit S, Ngampiyaskul C, Wongsawat J, Luesomboon W, Lumbiganon P, Sopa B, Apornpong T,	AIDS Res Hum Retroviruses. 2012 Dec;28(12):1679-86. doi: 10.1089/AID.2011.0373.

	Chuenyam T, Cooper DA, Ruxrungtham K, Ananworanich J, Puthanakit T.	
Health related quality of life in Middle Eastern children with beta-thalassemia.	Caocci G, Efficace F, Ciotti F, Roncarolo MG, Vacca A, Piras E, Littera R, Markous RS, Collins GS, Ciceri F, Mandelli F, Marktel S, La Nasa G.	BMC Blood Disord. 2012 Jun 22;12:6. doi: 10.1186/1471-2326-12-6.
Development of a fluorescence immunochromatographic assay for the detection of zeta globin in the blood of (--(SEA)) Î±-thalassemia carriers.	Wen L, Zhu P, Liu Y, Pan Q, Qu Y, Xu X, Li X, Fu N.	Blood Cells Mol Dis. 2012 Oct 15-Dec 15;49(3-4):128-32. doi: 10.1016/j.bcmd.2012.05.011.
Genetic origin and interaction of the Filipino Î²â◎°-thalassemia with Hb E and Î±-thalassemia in a Thai family.	Yamsri S, Sanchaisuriya K, Fucharoen G, Fucharoen S.	Transl Res. 2012 Jun;159(6):473-6. doi: 10.1016/j.trsl.2011.10.008.
The definition and epidemiology of non-transfusion-dependent thalassemia.	Weatherall DJ.	Blood Rev. 2012 Apr;26 Suppl 1:S3-6. doi: 10.1016/S0268-960X(12)70003-6. Review.
Inter-site validations of the Pixel-Wise method for cardiac T2* analysis in transfusion-dependent Thai thalassemia patients.	Saiviroonporn P, Viprakasit V, Maneesai A, Siritanaratkul N, Pongtanakul B, Wood JC, Krittayaphong R.	J Med Assoc Thai. 2012 Feb;95 Suppl 2:S165-72.
Contribution of examiner subjectivity and patient heterogeneity on long case examination.	Chierakul N, Danchaivijitr S, Kontee P, Naruman C.	J Med Assoc Thai. 2012 Feb;95 Suppl 2:S83-6.
Proxy indicators for identifying iron deficiency among anemic vegetarians in an area prevalent for thalassemia and hemoglobinopathies.	Wongprachum K, Sanchaisuriya K, Sanchaisuriya P, Siridamrongvattana S, Manpeun S, Schlep FP.	Acta Haematol. 2012;127(4):250-5. doi: 10.1159/000337032.
[Medical support on human resources and clinical laboratory in Myanmar].	Koide N.	Rinsho Byori. 2012 Mar;60(3):233-42. Japanese.
Hb A2/E levels found in co-inheritance with the Î±-thalassemia-1 - -(SEA)/type deletion and either Hb E or Î²-thalassemia.	Pornprasert S, Treesuwan K, Punyamung M, Kongthai K.	Hemoglobin. 2012;36(4):381-7. doi: 10.3109/03630269.2012.679375.
Coinheritance of Hb S [Î²6(A3)Gluâ†'Val, GAG>GTG] with Î²0-thalassemia codon 17 (A>T) in a Thai patient.	Pornprasert S, Panyasai S, Kongthai K, Treesuwan K.	Hemoglobin. 2012;36(3):265-9. doi: 10.3109/03630269.2012.669358.
Thalassaemia screening among students in a secondary school in Ampang, Malaysia.	Jameela S, Sabirah SO, Babam J, Phan CL, Visalachy P, Chang KM, Salwana MA, Zuraidah A, Subramanian Y, Rahimah A.	Med J Malaysia. 2011 Dec;66(5):522-4.
Clinical indicators for pulmonary arterial hypertension in thalassemia.	Chueamuangphan N, Wongtheptien W, Nawarawong W, Sukornthasarn A, Chuncharunee S, Tawichasri C, Patumanond J.	J Med Assoc Thai. 2012 Jan;95(1):16-21.
A rare Hb H disease due to the - -(SEA) and 16.6 kb Î±-thalassemia-2 deletions.	Sroymora S, Jindadamrongwech S, Butthep P, Chuncharunee S.	Hemoglobin. 2012;36(2):200-4. doi: 10.3109/03630269.2012.655355.

Molecular study and genotype/phenotype correlation of Î² Thalassemia in Malaysia.	Sivalingam M, Looi ML, Zakaria SZ, Hamidah NH, Alias H, Latiff ZA, Ibrahim H, Jamal R.	Int J Lab Hematol. 2012 Aug;34(4):377-82. doi: 10.1111/j.1751-553X.2012.01405.x.
Community participation for thalassemia prevention initiated by village health volunteers in northeastern Thailand.	Jopang Y, Petchmark S, Jetsrisuparb A, Sanchaisuriya K, Sanchaisuriya P, Schelp FP.	Asia Pac J Public Health. 2015 Mar;27(2):NP2144-56. doi: 10.1177/1010539511430520.
Health-related quality of life in Thai thalassemic children treated with iron chelation.	Torcharus K, Pankaew T.	Southeast Asian J Trop Med Public Health. 2011 Jul;42(4):951-9.
Craniofacial deformities in transfusion-dependent thalassemia patients in Malaysia: prevalence and effect of treatment.	Toman HA, Hassan R, Hassan R, Nasir A.	Southeast Asian J Trop Med Public Health. 2011 Sep;42(5):1233-40.
Multicenter validation of fully automated capillary electrophoresis method for diagnosis of thalassemias and hemoglobinopathies in Thailand.	Sangkitporn S, Sangkitporn SK, Tanjatham S, Suwannakan B, Rithapirom S, Yodtup C, Yowang A, Duangruang S.	Southeast Asian J Trop Med Public Health. 2011 Sep;42(5):1224-32.
Towards a prevention program for Î²-thalassemia. The molecular spectrum in East Java, Indonesia.	Hernanda PY, Tursilowati L, Arkesteijn SG, Ugrasena ID, Larasati MC, Soeatmadji SM, Giordano PC, Harteveld CL.	Hemoglobin. 2012;36(1):1-6. doi: 10.3109/03630269.2011.642914.
Australian Paediatric Surveillance Unit study of haemoglobinopathies in Australian children.	Argent E, Emder P, Monagle P, Mowat D, Petterson T, Russell S, Sachdev R, Stone C, Ziegler DS.	J Paediatr Child Health. 2012 Apr;48(4):356-60. doi: 10.1111/j.1440-1754.2011.02236.x.
Hb H disease with various Î² hemoglobinopathies: molecular, hematological and diagnostic aspects.	Fucharoen S, Fucharoen G.	Hemoglobin. 2012;36(1):18-24. doi: 10.3109/03630269.2011.638406.
Micromapping of thalassemia and hemoglobinopathies in diferent regions of northeast Thailand and Vientiane, Laos People's Democratic Republic.	Tritipsombut J, Sanchaisuriya K, Phollarp P, Bouakhasith D, Sanchaisuriya P, Fucharoen G, Fucharoen S, Schelp FP.	Hemoglobin. 2012;36(1):47-56. doi: 10.3109/03630269.2011.637149.
Haemoglobinopathies in southeast Asia.	Fucharoen S, Winichagoon P.	Indian J Med Res. 2011 Oct;134:498-506. Review.
Berberine-induced haemolysis revisited: safety of Rhizoma coptidis and Cortex phellodendri in chronic haematological diseases.	Linn YC, Lu J, Lim LC, Sun H, Sun J, Zhou Y, Ng HS.	Phytother Res. 2012 May;26(5):682-6. doi: 10.1002/ptr.3617.
First description of a Hb A2 variant in Thailand. Identification of Hb A2-Melbourne [Î´43(CD2)Gluâ†’Lys] in Thai individuals.	Chaibunruang A, Fucharoen G, Fucharoen S.	Hemoglobin. 2012;36(1):80-4. doi: 10.3109/03630269.2011.599465.
Î±-Hemoglobin stabilizing protein: a modulating factor in thalassemias?	Wajcman H, Vasseur C, Pissard S, Baudin-Creuza V.	Hemoglobin. 2011;35(5-6):463-8. doi: 10.3109/03630269.2011.576354.
The impact of thalassemia on Southeast Asian and Asian Indian	Liem RI, Gilgour B, Pelligra SA, Mason M, Thompson AA.	Ethn Dis. 2011 Summer;21(3):361-9.

families in the United States: a qualitative study.		
Cytokine-induced apoptosis of beta-thalassemia/hemoglobin E erythroid progenitor cells via nitric oxide-mediated process in vitro.	Kheansaard W, Panichob P, Fucharoen S, Tanyong DI.	Acta Haematol. 2011;126(4):224-30. doi: 10.1159/000329903.
Use of deferiprone for iron chelation in patients with transfusion-dependent thalassaemia.	Jamuar SS, Lai AH, Tan AM, Chan MY, Tan ES, Ng IS.	J Paediatr Child Health. 2011 Nov;47(11):812-7. doi: 10.1111/j.1440-1754.2011.02031.x.
Genotype-phenotype diversity of beta-thalassemia in Malaysia: treatment options and emerging therapies.	George E, Ann TJ.	Med J Malaysia. 2010 Dec;65(4):256-60. Review.
Alpha thalassemia major--a hematological prelude to an oncological problem.	Pant S.	Med Hypotheses. 2011 Nov;77(5):786-7. doi: 10.1016/j.mehy.2011.07.037.
Chelation therapy with desferrioxamine does not normalize ferritin level but attenuates oxidative damage and improves total antioxidant level in Malaysian Chinese beta-thalassaemia major patients.	Kuppusamy UR, Tan JA.	West Indian Med J. 2011 Jan;60(1):3-8.
A rapid detection for Î±-thalassemia by PCR combined with dissociation curve analysis.	Jia X, Liu J, Wang L, Yao L, Tang N, Cai R, Xiao B.	Exp Mol Pathol. 2011 Oct;91(2):626-30. doi: 10.1016/j.yexmp.2011.06.013.
Feasibility of preconception screening for thalassaemia in Indonesia: exploring the opinion of Javanese mothers.	Widayanti CG, Ediati A, Tamam M, Faradz SM, Sistermans EA, Plass AM.	Ethn Health. 2011 Aug-Oct;16(4-5):483-99. doi: 10.1080/13557858.2011.564607.
Increased oxidative metabolism is associated with erythroid precursor expansion in Î²0-thalassaemia/Hb E disease.	Leecharoenkiat A, Wannatung T, Lithanatudom P, Svasti S, Fucharoen S, Chokchaichamnankit D, Srisomsap C, Smith DR.	Blood Cells Mol Dis. 2011 Oct 15;47(3):143-57. doi: 10.1016/j.bcmd.2011.06.005.
Detection of Î±-thalassemia-1 Southeast Asian and Thai type deletions and Î²-thalassemia 3.5-kb deletion by single-tube multiplex real-time PCR with SYBR Green1 and high-resolution melting analysis.	Pornprasert S, Wiengkum T, Srithep S, Chainoi I, Singboottra P, Wongwiwatthananukit S.	Korean J Lab Med. 2011 Jul;31(3):138-42. doi: 10.3343/kjlm.2011.31.3.138.
Quantitative ultrasound measurement of the calcaneus in Southeast Asian children with thalassemia: comparison with dual-energy X-ray absorptiometry.	Sani FM, Sarji SA, Bilgen M.	J Ultrasound Med. 2011 Jul;30(7):883-94.
Phosphoproteomic analysis of apoptotic hematopoietic stem cells from hemoglobin E/Î²-thalassemia.	Ponnikorn S, Panichakul T, Sresanga K, Wongborisuth C, Roytrakul S, Hongeng S, Tungpradabkul S.	J Transl Med. 2011 Jun 25;9:96. doi: 10.1186/1479-5876-9-96.

Interference of hemoglobin Hope on beta-thalassemia diagnosis by the capillary electrophoresis Method.	Panyasai S, Sukunthamala K, Jaiping K, Wongwiwatthananukit S, Singboottra P, Pornprasert S.	Am J Clin Pathol. 2011 Jul;136(1):14-8. doi: 10.1309/AJCP1BT2MGATKFHL.
Genotype and phenotype characterizations in a large cohort of Î²-thalassemia heterozygote with different forms of Î±-thalassemia in northeast Thailand.	Yamsri S, Sanchaisuriya K, Fucharoen G, Sae-Ung N, Fucharoen S.	Blood Cells Mol Dis. 2011 Aug 15;47(2):120-4. doi: 10.1016/j.bcmd.2011.05.003.
Iron-chelating therapies in a transfusion-dependent thalassaemia population in Thailand: a cost-effectiveness study.	Luangasanatip N, Chaiyakunapruk N, Upakdee N, Wong P.	Clin Drug Investig. 2011;31(7):493-505. doi: 10.2165/11587120-000000000-00000.
Systematic review: epidemiology of hepatitis C genotype 6 and its management.	Chao DT, Abe K, Nguyen MH.	Aliment Pharmacol Ther. 2011 Aug;34(3):286-96. doi: 10.1111/j.1365-2036.2011.04714.x. Review.
Comparison of the region-based and pixel-wise methods for cardiac T2* analysis in 50 transfusion-dependent Thai thalassemia patients.	Saiviroonporn P, Viprakasit V, Boonyasirinant T, Khuhapinant A, Wood JC, Krittayaphong R.	J Comput Assist Tomogr. 2011 May-Jun;35(3):375-81. doi: 10.1097/RCT.0b013e31820eaaf2.
Evaluation of the URIT-2900 automated hematology analyzer for screening of thalassemia and hemoglobinopathies in Southeast Asian populations.	Karnpean R, Pansuwan A, Fucharoen G, Fucharoen S.	Clin Biochem. 2011 Jul;44(10-11):889-93. doi: 10.1016/j.clinbiochem.2011.04.009.
Hematological characteristics and effective screening for compound heterozygosity for Hb constant spring and deletional Î±+ -thalassemia.	Uaprasert N, Rojnuckarin P, Settapiboon R, Amornsiriwat S, Sutcharitchan P.	Am J Hematol. 2011 Jul;86(7):615-7. doi: 10.1002/ajh.22033. No abstract available.
[Molecular characterization of two new mutations of Î±Â° thalassemia in two Spanish families (mutation --(ED) and --(GP))].	Villegas A, Ropero P, Anguita E, HernÃ¡ndez A, Polo M, AtaÃºlfo GonzÃ¡lez F.	Med Clin (Barc). 2011 May 28;136(15):674-7. doi: 10.1016/j.medcli.2011.01.015. Spanish.
Public perceptions and attitudes toward thalassaemia: Influencing factors in a multi-racial population.	Wong LP, George E, Tan JA.	BMC Public Health. 2011 Mar 30;11:193. doi: 10.1186/1471-2458-11-193.
Self-reported level of and factors influencing the compliance to desferrioxamine therapy in multitransfused thalassaemias.	Lee WS, Toh TH, Chai PF, Soo TL.	J Paediatr Child Health. 2011 Aug;47(8):535-40. doi: 10.1111/j.1440-1754.2011.02017.x.
Clinical efficacy and safety evaluation of tailoring iron chelation practice in thalassaemia patients from Asia-Pacific: a subanalysis of the EPIC study of deferasirox.	Viprakasit V, Ibrahim H, Ha SY, Ho PJ, Li CK, Chan LL, Chiu CF, Sutcharitchan P, Habr D, Domokos G, Roubert B, Xue HL, Bowden DK, Lin KH.	Int J Hematol. 2011 Mar;93(3):319-28. doi: 10.1007/s12185-011-0789-8.
Presumptive diagnosis of common haemoglobinopathies in Southeast Asia using a capillary electrophoresis system.	Fucharoen G, Srivorakun H, Singsanan S, Fucharoen S.	Int J Lab Hematol. 2011 Aug;33(4):424-33. doi: 10.1111/j.1751-553X.2011.01301.x.

Hemoglobin Lepore EF Bart's disease: a molecular, hematological, and diagnostic aspects.	Chaibunruang A, Fucharoen G, Jetsrisuparb A, Fucharoen S.	Ann Hematol. 2011 Nov;90(11):1337-40. doi: 10.1007/s00277-011-1173-8.
Biopsychosocial predictors of health-related quality of life in children with thalassemia in Thammasat University Hospital.	Surapolchai P, Satayasai W, Sinlapamongkolkul P, Udomsubpayakul U.	J Med Assoc Thai. 2010 Dec;93 Suppl 7:S65-75.
Skeletal, dentoalveolar, and soft tissue cephalometric measurements of Malay transfusion-dependent thalassaemia patients.	Toman HA, Nasir A, Hassan R, Hassan R.	Eur J Orthod. 2011 Dec;33(6):700-4. doi: 10.1093/ejo/cjq147.
Thalassemia and hemoglobinopathies in Southeast Asian newborns: diagnostic assessment using capillary electrophoresis system.	Srivorakun H, Fucharoen G, Changtrakul Y, Komwilaisak P, Fucharoen S.	Clin Biochem. 2011 Apr;44(5-6):406-11. doi: 10.1016/j.clinbiochem.2011.01.006.
Anemia, iron deficiency and thalassemia among adolescents in Northeast Thailand: results from two independent surveys.	Pansuwan A, Fucharoen G, Fucharoen S, Himakhun B, Dangwiboon S.	Acta Haematol. 2011;125(4):186-92. doi: 10.1159/000322666.
Prevalence of fractures among Thais with thalassaemia syndromes.	Sutipornpalangkul W, Janechetsadatham Y, Siritanaratkul N, Harnroongroj T.	Singapore Med J. 2010 Oct;51(10):817-21.
Molecular screening of the Hbs Constant Spring (codon 142, TAA>CAA, Î±2) and PaksÃ© (codon 142, TAA>TAT, Î±2) mutations in Thailand.	Pichanun D, Munkongdee T, Klamchuen S, Butthep P, Winichagoon P, Fucharoen S, Svasti S.	Hemoglobin. 2010;34(6):582-6. doi: 10.3109/03630269.2010.526914.
High prevalence of alpha- and beta-thalassemia in the Kadazandusuns in East Malaysia: challenges in providing effective health care for an indigenous group.	Tan JA, Lee PC, Wee YC, Tan KL, Mahali NF, George E, Chua KH.	J Biomed Biotechnol. 2010;2010. pii: 706872. doi: 10.1155/2010/706872.
The prevalence of iron deficiency anemia in pregnant women in Nakhonsawan, Thailand.	Sukrat B, Suwathanapisate P, Siritawee S, Poungthong T, Phupongpankul K.	J Med Assoc Thai. 2010 Jul;93(7):765-70.
Title	Description	Details
Hb Koya Dora [alpha142, Term-->Ser (TAA>TCA in alpha2)]: a rare mutation of the alpha2 gene stop codon associated with alpha-thalassemia.	Brennan SO, Ryken S, Chan T.	Hemoglobin. 2010;34(4):402-5. doi: 10.3109/03630269.2010.486344.
A cost-utility and budget impact analysis of allogeneic hematopoietic stem cell transplantation for severe thalassemic patients in Thailand.	Leelahavarong P, Chaikledkaew U, Hongeng S, Kasemsup V, Lubell Y, Teerawattananon Y.	BMC Health Serv Res. 2010 Jul 16;10:209. doi: 10.1186/1472-6963-10-209.
[Detection of deletional alpha-thalassemia from free fetal DNA in maternal plasma].	Chen Y, Ou QS, Zhou HR.	Zhongguo Shi Yan Xue Ye Xue Za Zhi. 2010 Jun;18(3):679-82. Chinese.
Epidemiology of stillbirth and strategies for its prevention in Vietnam.	Hirst JE, Arbuckle SM, Do TM, Ha LT, Jeffery HE.	Int J Gynaecol Obstet. 2010 Aug;110(2):109-13. doi: 10.1016/j.ijgo.2010.03.028.

Prevention of severe thalassemia in northeast Thailand: 16 years of experience at a single university center.	Yamsri S, Sanchaisuriya K, Fucharoen G, Sae-Ung N, Ratanasiri T, Fucharoen S.	Prenat Diagn. 2010 Jun;30(6):540-6. doi: 10.1002/pd.2514.
Large scale screening for haemoglobin disorders in southern Vietnam: implications for avoidance and management.	O'Riordan S, Hien TT, Miles K, Allen A, Quyen NN, Hung NQ, Anh DQ, Tuyen LN, Khoa DB, Thai CQ, Triet DM, Phu NH, Dunstan S, Peto T, Clegg J, Farrar J, Weatherall D.	Br J Haematol. 2010 Aug;150(3):359-64. doi: 10.1111/j.1365-2141.2010.08237.x.
[Case of deletion type beta(0)-thalassemia found in an Asian (Malaysian) family living in Japan].	Harano K, Harano T.	Rinsho Byori. 2010 Apr;58(4):325-31. Japanese.
Thalassemia and iron deficiency in a group of northeast Thai school children: relationship to the occurrence of anemia.	Panomai N, Sanchaisuriya K, Yamsri S, Sanchaisuriya P, Fucharoen G, Fucharoen S, Schelp FP.	Eur J Pediatr. 2010 Nov;169(11):1317-22. doi: 10.1007/s00431-010-1218-3.
Beta-thalassemia cardiomyopathy: history, present considerations, and future perspectives.	Kremastinos DT, Farmakis D, Aessopos A, Hahalis G, Hamodraka E, Tsiapras D, Keren A.	Circ Heart Fail. 2010 May;3(3):451-8. doi: 10.1161/CIRCHEARTFAILURE.109.913863. Review.
Improvement of alpha(0)-thalassemia screening using combined osmotic fragility, dichlorophenolindophenol and Hb H inclusion tests.	Chaibunruang A, Pornphannukool S, Sae-Ung N, Fucharoen G, Sanchaisuriya K, Fucharoen S.	Clin Lab. 2010;56(3-4):111-7.
High-throughput beta-thalassemia carrier screening by allele-specific Q-primer real-time polymerase chain reaction.	Liu X, Law HY, Tan YM, Hong Y.	Anal Biochem. 2010 Sep 1;404(1):97-9. doi: 10.1016/j.ab.2010.04.025.
A comprehensive, simple molecular assay of common deletions and mutations causing alpha-thalassemia in Southeast Asia and southern China.	Liu J, Jia X, Tang N, Wang L, Han H, Cai R, Wang Q, Xiao B.	Am J Hematol. 2010 May;85(5):370-2. doi: 10.1002/ajh.21671. No abstract available.
Two new alpha1-globin gene point mutations: Hb Nedlands (HBA1:c.86C>T) [alpha28(B9)Ala->Val] and Hb Queens Park (HBA1:c.98T>A) [alpha32(B13)Met->Lys].	Phylipsen M, Prior JF, Lim E, Lingam N, Finlayson J, Arkesteijn SG, Harteveld CL, Giordano PC.	Hemoglobin. 2010 Jan;34(2):123-6. doi: 10.3109/03630261003679854.
Complex interaction of Hb Q-Thailand and Hb E with alpha(0)-thalassemia and hereditary persistence of fetal hemoglobin in a Chinese family.	Zheng W, Liu Y, Chen D, Rong K, Ge Y, Gong C, Chen H.	Ann Hematol. 2010 Sep;89(9):883-8. doi: 10.1007/s00277-010-0935-z.
Cord blood screening for alpha-thalassemia and hemoglobin variants by isoelectric focusing in northern Thai neonates: correlation with genotypes and hematologic parameters.	Charoenkwan P, Taweephol R, Sirichotiyakul S, Tantiprabha W, Sae-Tung R, Suanta S, Sakdasirisathaporn P, Sanguansermsri T.	Blood Cells Mol Dis. 2010 Jun 15;45(1):53-7. doi: 10.1016/j.bcmd.2010.02.015.

Non-O1, non-O139 Vibrio cholerae bacteraemia in splenectomised thalassaemic patient from Malaysia.	Deris ZZ, Leow VM, Wan Hassan WM, Nik Lah NA, Lee SY, Siti Hawa H, Siti Asma H, Ravichandran M.	Trop Biomed. 2009 Dec;26(3):320-5.
Hydrops fetalis in the stillborn: a series from the central region of Thailand.	Taweevisit M, Thorner PS.	Pediatr Dev Pathol. 2010 Sep-Oct;13(5):369-74. doi: 10.2350/09-12-0771-OA.1.
High resolution DNA melting analysis: an application for prenatal control of alpha-thalassemia.	Sirichotiyakul S, Wanapirak C, Saetung R, Sanguansermsri T.	Prenat Diagn. 2010 Apr;30(4):348-51. doi: 10.1002/pd.2480.
Infections and illnesses in children with Bb E beta-thalassemia: a prospective controlled study.	Jetsrisuparb A, Jetsrisuparb C.	J Med Assoc Thai. 2010 Jan;93(1):48-55.
Novel technique for rapid detection of alpha-globin gene mutations and deletions.	Liu J, Jia X, Tang N, Zhang X, Wu X, Cai R, Wang L, Liu Q, Xiao B, Zhu J, Wang Q.	Transl Res. 2010 Mar;155(3):148-55. doi: 10.1016/j.trsl.2009.10.003.
Amelioration of oxidative stress in red blood cells from patients with beta-thalassemia major and intermedia and E-beta-thalassemia following administration of a fermented papaya preparation.	Fibach E, Tan ES, Jamuar S, Ng I, Amer J, Rachmilewitz EA.	Phytother Res. 2010 Sep;24(9):1334-8. doi: 10.1002/ptr.3116.
Non-haem iron-mediated oxidative stress in haemoglobin E beta-thalassaemia.	Chakraborty I, Mitra S, Gachhui R, Kar M.	Ann Acad Med Singapore. 2010 Jan;39(1):13-6.
Whole blood and component use in resource poor settings.	Marwaha N.	Biologicals. 2010 Jan;38(1):68-71. doi: 10.1016/j.biologicals.2009.10.020. Review.
Sensitivity and specificity of simple erythrocyte osmotic fragility test for screening of alpha-thalassemia-1 and Beta-thalassemia trait in pregnant women.	Tongprasert F, Sirichotiyakul S, Piyamongkol W, Tongsong T.	Gynecol Obstet Invest. 2010;69(4):217-20. doi: 10.1159/000271779.
Drug metabolizing enzyme CYP1A2 status in pediatric patients with hemoglobin E-beta thalassemia.	Senggunprai L, Kukongviriyapan U, Jetsrisuparb A, Kukongviriyapan V.	J Med Assoc Thai. 2009 Dec;92(12):1675-80.
Interactions of hemoglobin Lepore (deltabeta hybrid hemoglobin) with various hemoglobinopathies: A molecular and hematological characteristics and differential diagnosis.	Chaibunruang A, Srivorakun H, Fucharoen S, Fucharoen G, Sae-ung N, Sanchaisuriya K.	Blood Cells Mol Dis. 2010 Mar 15;44(3):140-5. doi: 10.1016/j.bcmd.2009.11.008.
Fetal hemoglobin in sickle cell anemia: genome-wide association studies suggest a regulatory region in the 5' olfactory receptor gene cluster.	Solovieff N, Milton JN, Hartley SW, Sherva R, Sebastiani P, Dworkis DA, Klings ES, Farrer LA, Garrett ME, Ashley-Koch A, Telen MJ, Fucharoen S, Ha SY, Li CK, Chui DH, Baldwin CT, Steinberg MH.	Blood. 2010 Mar 4;115(9):1815-22. doi: 10.1182/blood-2009-08-239517.
Rapid diagnosis of the alpha-thalassemia-1 Southeast Asian type deletion using a single tube real-time	Liu JZ, Xiao B, Wang QT, Wang LR.	Hemoglobin. 2009;33(6):546-9. doi: 10.3109/03630260903337055.

SYBR-polymerase chain reaction combined with dissociation curve analysis.		
SYTO9 and SYBR GREEN1 with high resolution melting analysis for molecular confirmatory testing of the common Southeast Asian beta(0)-thalassemia mutations.	Chamras U, Sukunthamala K, Pornprasert S.	Hemoglobin. 2009;33(6):539-45. doi: 10.3109/03630260903336560.
Complex interaction of Hb E [beta26(B8)Glu-->Lys], Hb Korle-Bu [beta73(E17)Asp-->Asn] and a deletional alpha-thalassemia-1 in pregnancy.	Siriratmanawong N, Chansri W, Singsanan S, Fucharoen G, Fucharoen S.	Hemoglobin. 2009;33(6):507-14. doi: 10.3109/03630260903343780.
Successful preimplantation genetic diagnosis of Hb Bart's hydrops fetalis in Singapore after fresh and frozen embryo replacement cycles.	Yap C, Wang W, Tan AS, Tan WC, Lim MN, Chong SS.	Ann Acad Med Singapore. 2009 Oct;38(10):910-3.
False positive rates of thalassemia screening in rural clinical setting: 10-year experience in Thailand.	Jopang Y, Thinkhamrop B, Puangpruk R, Netnee P.	Southeast Asian J Trop Med Public Health. 2009 May;40(3):576-80.
First successful preimplantation genetic diagnosis in Singapore--avoidance of beta-thalassaemia major.	Yap C, Tan AS, Wang W, Lim MN, Chong SS.	Ann Acad Med Singapore. 2009 Aug;38(8):720-3.
Quality of life in transfusion-dependent thalassaemia patients on desferrioxamine treatment.	Dahlui M, Hishamshah MI, Rahman AJ, Aljunid SM.	Singapore Med J. 2009 Aug;50(8):794-9.
Improvement in the detection of alpha0- and deletional alpha-thalassemia by real-time PCR combined with dissociation curve analysis.	Liu J, Tang N, Liu Q, Wang L, Han H, Cai R, Wu X, Xiao B.	Acta Haematol. 2009;122(1):17-22. doi: 10.1159/000232578.
Variable clinical phenotypes of alpha-thalassemia syndromes.	Singer ST.	ScientificWorldJournal. 2009 Jul 13;9:615-25. doi: 10.1100/tsw.2009.69. Review.
A comparison of the accuracy of the corpuscular fragility and mean corpuscular volume tests for the alpha-thalassemia 1 and beta-thalassemia traits.	Sirichotiyakul S, Wanapirak C, Srisupundit K, Luewan S, Tongsong T.	Int J Gynaecol Obstet. 2009 Oct;107(1):26-9. doi: 10.1016/j.ijgo.2009.05.012.
Rheumatic valvular heart disease in thalassaemic patients: a summary of reported Thai cases.	Wiwanitkit V.	Cardiovasc J Afr. 2009 May-Jun;20(3):200. Review.
Incidence of haemoglobinopathies in various populations - the impact of immigration.	Henderson S, Timbs A, McCarthy J, Gallienne A, Van Mourik M, Masters G, May A, Khalil MS, Schuh A, Old J.	Clin Biochem. 2009 Dec;42(18):1745-56. doi: 10.1016/j.clinbiochem.2009.05.012.
Hemagglutination test for rapid serodiagnosis of human pythiosis.	Jindayok T, Piromsontikorn S, Srimuang S, Khupulsup K, Krajaejun T.	Clin Vaccine Immunol. 2009 Jul;16(7):1047-51. doi: 10.1128/CVI.00113-09.

Evolution of hematological parameters in HIV-1-infected patients with and without thalassemia carriages during highly active antiretroviral therapy.	Pornprasert S, Sonboon P, Kiatwattanacharoen S, Klinbuayaem V, Leenasirimakul P, Promping C, Inta P, Ajhan S, Leechanachai P.	HIV Clin Trials. 2009 Mar-Apr;10(2):88-93. doi: 10.1310/hct1002-88.
Comparison of the accuracy of dichlorophenolindophenol (DCIP), modified DCIP, and hemoglobin E tests to screen for the HbE trait in pregnant women.	Wanapirak C, Sirichotiyakul S, Luewan S, Srisupundit K, Tongsong T.	Int J Gynaecol Obstet. 2009 Oct;107(1):59-60. doi: 10.1016/j.ijgo.2009.04.020. No abstract available.
Incidence, causes and pregnancy outcomes of hydrops fetalis at Srinagarind Hospital, 1996-2005: a 10-year review.	Ratanasiri T, Komwilaisak R, Sittivech A, Kleebkeaw P, Seejorn K.	J Med Assoc Thai. 2009 May;92(5):594-9.
Clinical and hematological characteristics of uncommon beta-globin variants in Thailand.	Uaprasert N, Rojnuckarin P, Settapiboon R, Amornsiriwat S, Sutcharitchan P.	Int J Hematol. 2009 Jun;89(5):568-71. doi: 10.1007/s12185-009-0334-1.
Thalassemia intermedia in HbH-CS disease with compound heterozygosity for beta-thalassemia: challenges in hemoglobin analysis and clinical diagnosis.	Tan JA, Kok JL, Tan KL, Wee YC, George E.	Genes Genet Syst. 2009 Feb;84(1):67-71.
Risk factors for gallstone disease in a Thai population.	Panpimanmas S, Manmee C.	J Epidemiol. 2009;19(3):116-21.
Clinical features and molecular analysis in Thai patients with HbH disease.	Laosombat V, Viprakasit V, Chotsampancharoen T, Wongchanchailert M, Khodchawan S, Chinchang W, Sattayasevana B.	Ann Hematol. 2009 Dec;88(12):1185-92. doi: 10.1007/s00277-009-0743-5.
Modification of platelet shape change parameter by oxidized lipoprotein from beta-thalassemia/Hemoglobin E.	Sutipornpalangkul W, Unchern S, Sanvarinda Y, Chantharaksri U, Fucharoen S.	J Med Assoc Thai. 2009 Apr;92(4):504-9.
Comparison of vascular complications between conventional treatment and bone marrow transplantation for children with beta-thalassemia disease.	Limsuwan A, Tubtom D, Pakakasama S, Chaunsumrit A.	Pediatr Cardiol. 2009 Aug;30(6):777-80. doi: 10.1007/s00246-009-9436-z.
Elevated serum transferrin receptor levels in common types of thalassemia heterozygotes in Southeast Asia: a correlation with genotypes and red cell indices.	Uaprasert N, Rojnuckarin P, Bhokaisawan N, Settapiboon R, Wacharaprechanont T, Amornsiriwat S, Sutcharitchan P.	Clin Chim Acta. 2009 May;403(1-2):110-3. doi: 10.1016/j.cca.2009.01.031.
Title	Description	Details
Development of a comprehensive red blood cell enzymopathy laboratory in Thailand: the study of normal activity in eight erythroenzymes in Thais.	Tachavanich K, Viprakasit V, Pung-Amritt P, Veerakul G, Chansing K, Tanphaichitr VS.	Southeast Asian J Trop Med Public Health. 2009 Mar;40(2):317-26.
Clinical and hematological phenotype of homozygous hemoglobin E: revisit of a benign condition with hidden reproductive risk.	Tachavanich K, Viprakasit V, Chinchang W, Glomglao W, Pung-Amritt P, Tanphaichitr VS.	Southeast Asian J Trop Med Public Health. 2009 Mar;40(2):306-16.

Thalassemic mothers and their babies.	Insiripong S, Prabriputaloong S, Wisanuyothin N.	Southeast Asian J Trop Med Public Health. 2009 Mar;40(2):302-5.
Perinatal zidovudine prophylaxis in HIV type-1-infected pregnant women with thalassaemia carriage in Thailand.	Briand N, Pornprasert S, Ngo-Giang-Huong N, GalactÃ©ros F, Pissard S, Tatu T, Sanguansermsri T, Jourdain G, Lallemant M, Le Coeur S.	Antivir Ther. 2009;14(1):117-22.
Detection of Hb E mutation (beta(26), GAG-AAG, Glu-Lys) using allelic discrimination analysis.	Sangkitporn S, Sangkitporn SK, Sangnoi A, Duangruang S.	Int J Lab Hematol. 2009 Feb;31(1):74-80.
Analysis of fetal blood using capillary electrophoresis system: a simple method for prenatal diagnosis of severe thalassemia diseases.	Srivorakun H, Fucharoen G, Sae-Ung N, Sanchaisuriya K, Ratanasiri T, Fucharoen S.	Eur J Haematol. 2009 Jul;83(1):57-65. doi: 10.1111/j.1600-0609.2009.01245.x.
Hemoglobin E detection using PCR with confronting two-pair primers.	Intorasoot S, Thongpung R, Tragoolpua K, Chottayaporn M.	J Med Assoc Thai. 2008 Nov;91(11):1677-80.
Detection of beta-globin gene mutations among Kelantan Malay thalassaemia patients by polymerase chain reaction restriction fragment length polymorphism.	Rozitah R, Nizam MZ, Nur Shafawati AR, Nor Atifah MA, Dewi M, Kannan TP, Ariffin N, Norsarwany M, Setianingsih I, Harahap A, Zilfalil BA.	Singapore Med J. 2008 Dec;49(12):1046-9.
Outcomes of pregnancies complicated by beta-thalassemia/hemoglobin E disease.	Luewan S, Srisupundit K, Tongsong T.	Int J Gynaecol Obstet. 2009 Mar;104(3):203-5. doi: 10.1016/j.ijgo.2008.10.012.
Newborn screening for hemoglobinopathies in California.	Michlitsch J, Azimi M, Hoppe C, Walters MC, Lubin B, Lorey F, Vichinsky E.	Pediatr Blood Cancer. 2009 Apr;52(4):486-90. doi: 10.1002/pbc.21883.
Rheumatological manifestations in patients with melioidosis.	Teparrakkul P, Tsai JJ, Chierakul W, Gerstenmaier JF, Wacharaprechasgu T, Piyaphanee W, Limmathurotsakul D, Chaowagul W, Day NP, Peacock SJ.	Southeast Asian J Trop Med Public Health. 2008 Jul;39(4):649-55.
Growth velocity in transfusion dependent prepubertal thalassemia patients: results from a thalassemia center in Malaysia.	Hamidah A, Arini MI, Zarina AL, Zulkifli SZ, Jamal R.	Southeast Asian J Trop Med Public Health. 2008 Sep;39(5):900-5.
Outcomes of pregnancies affected by hemoglobin H disease.	Tongsong T, Srisupundit K, Luewan S.	Int J Gynaecol Obstet. 2009 Mar;104(3):206-8. doi: 10.1016/j.ijgo.2008.10.010.
Hypochromic microcytic anemia with a variant hemoglobin.	Bain BJ.	Am J Hematol. 2009 Jan;84(1):59. doi: 10.1002/ajh.21300. No abstract available.
Hemoglobin profiles and hematologic features of thalassemic newborns: application to screening of alpha-thalassemia 1 and hemoglobin E.	Tritipsombut J, Sanchaisuriya K, Fucharoen S, Fucharoen G, Siriratmanawong N, Pinmuang-ngam C, Sanchaisuriya P.	Arch Pathol Lab Med. 2008 Nov;132(11):1739-45. doi: 10.1043/1543-2165-132.11.1739.
Molecular, hematological and clinical aspects of thalassemia major and thalassemia intermedia associated	Nuntakarn L, Fucharoen S, Fucharoen G, Sanchaisuriya K, Jetsrisuparb A, Wiangnon S.	Blood Cells Mol Dis. 2009 Jan-Feb;42(1):32-5. doi: 10.1016/j.bcmd.2008.09.002.

with Hb E-beta-thalassemia in Northeast Thailand.		
Hb Constant Spring [alpha 142, Term->Gln (TAA>CAA in alpha2)] in the alpha-thalassemia of anemic patients in Myanmar.	Ne-Win, Harano K, Harano T, Kyaw-Shwe, Aye-Aye-Myint, Khin-Thander-Aye, Okada S.	Hemoglobin. 2008;32(5):454-61. doi: 10.1080/03630260802341588.
Pregnancy outcomes in women complicated by thalassemia syndrome at Maharaj Nakorn Chiang Mai Hospital.	Traisrisilp K, Luewan S, Tongsong T.	Arch Gynecol Obstet. 2009 May;279(5):685-9. doi: 10.1007/s00404-008-0804-3.
Current status of hematopoietic stem cell transplantations in Vietnam.	van Be T, van Binh T, Binh N, Tuan T, Nghia H, Hien B.	Bone Marrow Transplant. 2008 Aug;42 Suppl 1:S146-S148. doi: 10.1038/bmt.2008.145.
Clinical silent cerebral infarct (SCI) in patients with thalassemia diseases assessed by magnetic resonance imaging (MRI).	Metarugcheep P, Chanyawattiwongs S, Srisubat K, Pootrakul P.	J Med Assoc Thai. 2008 Jun;91(6):889-94.
Transabdominal chorionic villus sampling: experience at Maharaj Nakorn Chiang Mai Hospital.	Sirichotiyakul S, Piyamongkol W, Tongprasert F, Srisupandit K, Luewan S.	J Med Assoc Thai. 2008 Jun;91(6):813-7.
BCL11A is a major HbF quantitative trait locus in three different populations with beta-hemoglobinopathies.	Sedgewick AE, Timofeev N, Sebastiani P, So JC, Ma ES, Chan LC, Fucharoen G, Fucharoen S, Barbosa CG, Vardarajan BN, Farrer LA, Baldwin CT, Steinberg MH, Chui DH.	Blood Cells Mol Dis. 2008 Nov-Dec;41(3):255-8. doi: 10.1016/j.bcmd.2008.06.007.
Detection of beta-thalassemia mutations using a multiplex amplification refractory mutation system assay.	Mirasena S, Shimbhu D, Sanguansermsri M, Sanguansermsri T.	Hemoglobin. 2008;32(4):403-9. doi: 10.1080/03630260701798391.
Hb Q-H disease: two cases in a Cantonese family.	Lin M, Wu JR, Yang LY, Zou HY, Wang Q, Zheng L.	Blood Cells Mol Dis. 2008 Nov-Dec;41(3):259-60. doi: 10.1016/j.bcmd.2008.06.001. No abstract available.
Genetic disorders and malaria in Indo-China region.	Wiwanitkit V.	J Vector Borne Dis. 2008 Jun;45(2):98-104. Review.
Co-inheritance of alpha-and beta-thalassemia in Khuzestan Province, Iran.	Rahim F, Kaikhaei B, Zandian K, Hoseini A.	Hematology. 2008 Feb;13(1):59-64. doi: 10.1179/102453308X315843.
Identification of beta-globin gene mutations in Thailand using an automated fluorescence-based DNA sequencer.	Sangkitporn SK, Eksiri L, Sangnoi A, Duangruang S, Dumbua A, Rattanakittisophon K, Sangkitporn S.	Int J Lab Hematol. 2009 Oct;31(5):521-7. doi: 10.1111/j.1751-553X.2008.01072.x.
Detection and haplotype differentiation of Southeast Asian alpha-thalassemia using polymerase chain reaction and a piezoelectric biosensor immobilized with a single oligonucleotide probe.	Vattanaviboon P, Sangseekhiow K, Winichagoon P, Promptmas C.	Transl Res. 2008 May;151(5):246-54. doi: 10.1016/j.trsl.2007.12.009.

Evidence of gene conversion in the evolutionary process of the codon 41/42 (-CTTT) mutation causing beta-thalassemia in southern China.	Zhang W, Cai WW, Zhou WP, Li HP, Li L, Yan W, Deng QK, Zhang YP, Fu YX, Xu XM.	J Mol Evol. 2008 May;66(5):436-45. doi: 10.1007/s00239-008-9096-2.
Thalassemia and hemoglobinopathies in pregnant Lao women: carrier screening, prevalence and molecular basis.	Savongsy O, Fucharoen S, Fucharoen G, Sanchaisuriya K, Sae-Ung N.	Ann Hematol. 2008 Aug;87(8):647-54. doi: 10.1007/s00277-008-0490-z.
Alpha-thalassaemia.	Leung WC, Leung KY, Lau ET, Tang MH, Chan V.	Semin Fetal Neonatal Med. 2008 Aug;13(4):215-22. doi: 10.1016/j.siny.2008.02.006. Review.
Chorionic villus sampling for early prenatal diagnosis at Bhumibol Adulyadej Hospital.	Rueangchainikhom W, Sarapak S, Orungrote N.	J Med Assoc Thai. 2008 Jan;91(1):1-6.
Alpha-thalassaemia in association with beta-thalassaemia patients in Malaysia: a study on the co-inheritance of both disorders.	Wee YC, Tan KL, Kuldip K, Tai KS, George E, Tan PC, Chia P, Subramaniam R, Yap SF, Tan JA.	Community Genet. 2008;11(3):129-34. doi: 10.1159/000113874.
Increased urinary 1,N6-ethenodeoxyadenosine and 3,N4-ethenodeoxycytidine excretion in thalassemia patients: markers for lipid peroxidation-induced DNA damage.	Meerang M, Nair J, Sirankapracha P, Thephinlap C, Srichairatanakool S, Fucharoen S, Bartsch H.	Free Radic Biol Med. 2008 May 15;44(10):1863-8. doi: 10.1016/j.freeradbiomed.2008.02.009.
Multi-center transferability of a breath-hold T2 technique for myocardial iron assessment.	He T, Kirk P, Firmin DN, Lam WM, Chu WC, Au WY, Chan GC, Tan RS, Ng I, Biceroglu S, Aydinok Y, Fogel MA, Cohen AR, Pennell DJ.	J Cardiovasc Magn Reson. 2008 Feb 21;10:11. doi: 10.1186/1532-429X-10-11.
Hematological abnormalities in patients with distal renal tubular acidosis and hemoglobinopathies.	Khositseth S, Sirikanaerat A, Khoprasert S, Opastirakul S, Kingwatanakul P, Thongnoppakhun W, Yenchitsomanus PT.	Am J Hematol. 2008 Jun;83(6):465-71. doi: 10.1002/ajh.21151.
Platelet aggregation and activation in thalassemia major patients in Indonesia.	Setiabudy R, Wahidiyat PA, Setiawan L.	Clin Appl Thromb Hemost. 2008 Jul;14(3):346-51.
The prevalence and persistence of human parvovirus B19 infection in thalassemic patients.	Siritantikorn S, Kaewrawang S, Siritanaratkul N, Theamboonlers A, Poovorawan Y, Kantakamalakul W, Wasi C.	Asian Pac J Allergy Immunol. 2007 Jun-Sep;25(2-3):169-74.
Application of maternal plasma DNA analysis for noninvasive prenatal diagnosis of Hb E-beta-thalassemia.	Tungwiwat W, Fucharoen G, Fucharoen S, Ratanasiri T, Sanchaisuriya K, Sae-Ung N.	Transl Res. 2007 Nov;150(5):319-25.
Red cell autoantibodies among thalassaemia patients in Hospital Universiti Sains Malaysia.	Noor Haslina MN, Ariffin N, Illuni Hayati I, Rosline H.	Singapore Med J. 2007 Oct;48(10):922-5.
Beta-globin gene cluster polymorphisms are strongly associated with severity of HbE/beta(0)-thalassemia.	Ma Q, Abel K, Sripichai O, Whitacre J, Angkachatchai V, Makarasara W, Winichagoon P, Fucharoen S, Braun A, Farrer LA.	Clin Genet. 2007 Dec;72(6):497-505.

Identification of hemoglobin AC heterozygote status in a Malay family: a decision between hemoglobin electrophoresis and high performance liquid chromotography.	Rosline H, Roshan TM, Ahmed SA, Ilunihayati I.	Southeast Asian J Trop Med Public Health. 2007 May;38(3):543-5.
[Prenatal gene diagnosis of paternally inherited alpha-thalassemia by detecting fetal DNA in maternal plasma].	Chen P, Li MJ, Li MQ, Li SQ, Zhou LY, Lin WX.	Zhonghua Yi Xue Za Zhi. 2007 Jun 12;87(22):1540-4. Chinese.
The prevalence and causes of anemia during pregnancy in Maharaj Nakorn Chiang Mai Hospital.	Sukrat B, Sirichotiyakul S.	J Med Assoc Thai. 2006 Oct;89 Suppl 4:S142-6.
Prenatal prevention for severe thalassemia disease at Srinagarind Hospital.	Ratanasiri T, Charoenthong C, Komwilaisak R, Changtrakul Y, Fucharoen S, Wongkham J, Kleebkaow P, Seejorn K.	J Med Assoc Thai. 2006 Oct;89 Suppl 4:S87-93.
Molecular characterisation and frequency of Ggamma Xmn I polymorphism in Chinese and Malay beta-thalassaemia patients in Malaysia.	Wong YC, George E, Tan KL, Yap SF, Chan LL, Tan JA.	Malays J Pathol. 2006 Jun;28(1):17-21.
Haemoglobin Hope in a northern Thai family: first identification of homozygous haemoglobin Hope associated with haemoglobin H disease.	Sura T, Busabaratana M, Youngcharoen S, Wisedpanichkij R, Viprakasit V, Trachoo O.	Eur J Haematol. 2007 Sep;79(3):251-4.
Hemoglobin E disease in North Indian population: a report of 11 cases.	Kishore B, Khare P, Gupta RJ, Bisht S, Majumdar K.	Hematology. 2007 Aug;12(4):343-7.
H63D mutation of the hemochromatosis gene and serum ferritin levels in Thai thalassemia carriers.	Yamsri S, Sanchaisuriya K, Fucharoen S, Fucharoen G, Jetsrisuparb A, Wiangnon S, Changtrakul Y, Sanchaisuriya P.	Acta Haematol. 2007;118(2):99-105.
Is routine molecular screening for common alpha-thalassaemia deletions necessary as part of an antenatal screening programme?	Sorour Y, Heppinstall S, Porter N, Wilson GA, Goodeve AC, Rees D, Wright J.	J Med Screen. 2007;14(2):60-1.
Molecular characterization and origins of Hb Constant Spring and Hb PaksÃ© in Southeast Asian populations.	Singsanan S, Fucharoen G, Savongsy O, Sanchaisuriya K, Fucharoen S.	Ann Hematol. 2007 Sep;86(9):665-9.
Thalassemia intermedia associated with the Hb Constant Spring EE Bart's disease in pregnancy: a molecular and hematological analysis.	Fucharoen S, Fucharoen G, Sae-ung N, Sanchaisuriya K.	Blood Cells Mol Dis. 2007 Sep-Oct;39(2):195-8.
Distal renal tubular acidosis associated with anion exchanger 1 mutations in children in Thailand.	Khositseth S, Sirikanerat A, Wongbenjarat K, Opastirakul S, Khoprasert S, Peuksungnern R, Wattanasirichaigoon D, Thongnoppakhun W, Viprakasit V, Yenchitsomanus PT.	Am J Kidney Dis. 2007 Jun;49(6):841-850.e1.

Hemoglobin H disease induced by the common SEA deletion and the rare hemoglobin Quong Sze in a Thai female: longitudinal clinical course, molecular characterization, and development of a PCR/RFLP-based detection method.	Sura T, Trachoo O, Viprakasit V, Vathesatogkit P, Tunteeratum A, Busabaratana M, Wisedpanichkij R, Isarangkura P.	Ann Hematol. 2007 Sep;86(9):659-63.
Expression of betaE and gamma-globin genes in infants heterozygous for hemoglobin E and double heterozygous for hemoglobin E and alpha-thalassemia.	Winichagoon P, Svasti S, Winichagoon P, Chitchumroonchokchai C, Fucharoen S.	Haematologica. 2007 May;92(5):702-3.
The effect of iron fortification and de-worming on anaemia and iron status of Vietnamese schoolchildren.	Le Huong T, Brouwer ID, Nguyen KC, Burema J, Kok FJ.	Br J Nutr. 2007 May;97(5):955-62.
Prenatal diagnosis of Hb Bart's hydrops fetalis caused by a genetic compound heterozygosity for two different alpha-thalassemia determinants.	Siriratmanawong N, Pinmuang-Ngam C, Fucharoen G, Fucharoen S.	Fetal Diagn Ther. 2007;22(4):264-8.
Further identification of Hb G-Coushatta [beta22(B4)Glu-->Ala (GAA-->GCA)] in Thailand by the polymerase chain reaction-single-strand conformation polymorphism technique and by amplification refractory mutation system-polymerase chain reaction.	Chinchang W, Viprakasit V.	Hemoglobin. 2007;31(1):93-9.
Doppler waveform indices of the Middle Cerebral Artery of normal fetuses in the first half of pregnancy in the Thai population.	Rujiwetpongstorn J, Phupong V.	Arch Gynecol Obstet. 2007 Oct;276(4):351-4.
Red cell immunization in multiply transfused Malay thalassemic patients.	Noor Haslina MN, Ariffin N, Illuni Hayati I, Rosline H.	Southeast Asian J Trop Med Public Health. 2006 Sep;37(5):1015-20.
Effect of the maternal betaE-globin gene on hematologic responses to iron supplementation during pregnancy.	Sanchaisuriya K, Fucharoen S, Ratanasiri T, Sanchaisuriya P, Fucharoen G, Dietz E, Schelp FP.	Am J Clin Nutr. 2007 Feb;85(2):474-9.
Lived experiences of mothers caring for children with thalassemia major in Thailand.	Prasomsuk S, Jetsrisuparp A, Ratanasiri T, Ratanasiri A.	J Spec Pediatr Nurs. 2007 Jan;12(1):13-23.
Severe beta(0) thalassemia/hemoglobin E disease caused by de novo 22-base pair duplication in the paternal allele of beta globin gene.	Rojnuckarin P, Settapiboon R, Vanichsetakul P, Sueblinvong T, Sutcharitchan P.	Am J Hematol. 2007 Jul;82(7):663-5.
Microsatellite markers within --SEA breakpoints for prenatal diagnosis of HbBarts hydrops fetalis.	Ho SS, Chong SS, Koay ES, Chan YH, Sukumar P, Chiu LL, Wang W, Roy A, Rauff M, Su LL, Biswas A, Choolani M.	Clin Chem. 2007 Feb;53(2):173-9.

Risk of a couple having a child with severe thalassemia syndrome, prevalence in lower northern Thailand.	Wong P, Thanormrat P, Srithipayawan S, Jermnim N, Niyomthom S, Nimnuch N, Sanguansermsri T.	Southeast Asian J Trop Med Public Health. 2006 Mar;37(2):366-9.
Thalassemia among blood donors at the Hospital Universiti Sains Malaysia.	Rosline H, Ahmed SA, Al-Joudi FS, Rapiaah M, Naing NN, Adam NA.	Southeast Asian J Trop Med Public Health. 2006 May;37(3):549-52.
Modification of CYP2E1 and CYP3A4 activities in haemoglobin E-beta thalassemia patients.	Somparn N, Kukongviriyapan U, Tassaneeyakul W, Jetsrisuparb A, Kukongviriyapan V.	Eur J Clin Pharmacol. 2007 Jan;63(1):43-50.
Development and application of a real-time quantitative PCR for prenatal detection of fetal alpha(0)-thalassemia from maternal plasma.	Tungwiwat W, Fucharoen S, Fucharoen G, Ratanasiri T, Sanchaisuriya K.	Ann N Y Acad Sci. 2006 Sep;1075:103-7.
Alpha(0)-thalassemia and related disorders in northeast Thailand: a molecular and hematological characterization.	Sae-ung N, Fucharoen G, Sanchaisuriya K, Fucharoen S.	Acta Haematol. 2007;117(2):78-82.
Coexistence of Southeast Asian ovalocytosis and beta-thalassemia: a molecular and hematological analysis.	Fucharoen G, Fucharoen S, Singsanan S, Sanchaisuriya K.	Am J Hematol. 2007 May;82(5):381-5.
Characterization of a novel deletion causing (deltabeta)0-thalassemia in a Thai family.	Svasti S, Paksua S, Nuchprayoon I, Winichagoon P, Fucharoen S.	Am J Hematol. 2007 Feb;82(2):155-61.
Characterisation and confirmation of rare beta-thalassaemia mutations in the Malay, Chinese and Indian ethnic groups in Malaysia.	Tan JA, Chin PS, Wong YC, Tan KL, Chan LL, George E.	Pathology. 2006 Oct;38(5):437-41.
Two independent origins of Hb Dhonburi (Neapolis) [beta 126 (H4) Val-->Gly]: an electrophoretically silent hemoglobin variant.	Viprakasit V, Chinchang W.	Clin Chim Acta. 2007 Feb;376(1-2):179-83.
The prevalence and molecular basis of hemoglobinopathies in Cambodia.	Carnley BP, Prior JF, Gilbert A, Lim E, Devenish R, Sing H, Sarin E, Guhadasan R, Sullivan SG, Wise CA, Bittles AH, Chan K, Wong MS, Chan V, Erber WN.	Hemoglobin. 2006;30(4):463-70.
Successful preimplantation genetic diagnosis for alpha- and beta-thalassemia in China.	Deng J, Peng WL, Li J, Fang C, Liang XY, Zeng YH, Sun HY, Zhou CQ, Zhuang GL.	Prenat Diagn. 2006 Nov;26(11):1021-8.
Common alpha-thalassemia deletions in transfusion-dependent thalassemia patients in the Southeast Asia region of Myanmar.	Ne-Win, Harano K, Harano T, Thein-Thein-Myint, Rai-Mra, Aye-Aye-Myint, Shimono K, Okada S.	Lab Hematol. 2006;12(3):139-42.
Screening of concurrent alpha-thalassaemia 1 in beta-thalassaemia carriers.	Chong YM, Tan JA, Zubaidah Z, Rahimah A, Kuldip K, George E.	Med J Malaysia. 2006 Jun;61(2):217-20.
[Acute anaemia in a Vietnamese patient with alpha-thalassaemia and a parvovirus infection].	Slomp J, Bosschaart A, Dousma M, van Zwieten R, Giordano PC, van den Bergh FA.	Ned Tijdschr Geneeskd. 2006 Jul 15;150(28):1577-82. Dutch.

Clinical and epidemiological analyses of human pythiosis in Thailand.	Krajaejun T, Sathapatayavongs B, Pracharktam R, Nitiyanant P, Leelachaikul P, Wanachiwanawin W, Chaiprasert A, Assanasen P, Saipetch M, Mootsikapun P, Chetchotisakd P, Lekhakula A, Mitarnun W, Kalnauwakul S, Supparatpinyo K, Chaiwarith R, Chiewchanvit S, Tananuvat N, Srisiri S, Suankratay C, Kulwichit W, Wongsaisuwan M, et al.	Clin Infect Dis. 2006 Sep 1;43(5):569-76.
Regarding Luecke T, Pfister S, Duerken M. Neurodevelopmental outcome and haematological course of a long-time survivor with homozygous alpha-thalassaemia: case report and review of the literature. Acta Paediatr 2005;94:1330-3.	Dickerhoff R.	Acta Paediatr. 2006 Aug;95(8):1017-8; author reply 1018. No abstract available.
Enhanced oxidative cross-linking of hemoglobin E with spectrin and loss of erythrocyte membrane asymmetry in hemoglobin Ebeta-thalassemia.	Datta P, Basu S, Chakravarty SB, Chakravarty A, Banerjee D, Chandra S, Chakrabarti A.	Blood Cells Mol Dis. 2006 Sep-Oct;37(2):77-81.
Effect of coenzyme Q10 as an antioxidant in beta-thalassemia/Hb E patients.	Kalpravidh RW, Wichit A, Siritanaratkul N, Fucharoen S.	Biofactors. 2005;25(1-4):225-34.
Screening for thalassemia and hemoglobinopathy in a rural area of Thailand: a preliminary study.	Nathalang O, Nillakupt K, Arnutti P, Boonsiri T, Panichkul S, Areekul W.	J Med Assoc Thai. 2005 Nov;88 Suppl 3:S35-42.
Hematological parameters and red blood cell indices in healthy Thai children: a revision for 2005.	Viprakasit V, Suwanthol L, Sangpraypan T, Glomglao W, Utto W, Veerakul G.	J Med Assoc Thai. 2005 Nov;88 Suppl 8:S188-96.
Dengue hemorrhagic fever in patients with thalassemia.	Pongtanakul B, Narkbunnam N, Veerakul G, Sanpakit K, Viprakasit V, Tanphaichitr VT, Suvatte V.	J Med Assoc Thai. 2005 Nov;88 Suppl 8:S80-5.
Anaemia and iron deficiency anaemia among young adolescent girls from the peri urban coastal area of Indonesia.	Kurniawan YA, Muslimatun S, Achadi EL, Sastroamidjojo S.	Asia Pac J Clin Nutr. 2006;15(3):350-6.
Health Related Quality of Life in Malaysian children with thalassaemia.	Ismail A, Campbell MJ, Ibrahim HM, Jones GL.	Health Qual Life Outcomes. 2006 Jul 2;4:39.
Thalassemia and hemoglobinopathies rather than iron deficiency are major causes of pregnancy-related anemia in northeast Thailand.	Sanchaisuriya K, Fucharoen S, Ratanasiri T, Sanchaisuriya P, Fucharoen G, Dietz E, Schelp FP.	Blood Cells Mol Dis. 2006 Jul-Aug;37(1):8-11.
Molecular mechanism of high hemoglobin F production in Southeast Asian-type hereditary persistence of fetal hemoglobin.	Changsri K, Akkarapathumwong V, Jamsai D, Winichagoon P, Fucharoen S.	Int J Hematol. 2006 Apr;83(3):229-37.
An increase of the cardiothoracic ratio leads to a diagnosis of Bart's hydrops.	Phupong V.	J Med Assoc Thai. 2006 Apr;89(4):509-12.

Haemoglobin Lepore in a Malay family: a case report.	Pasangna J, George E, Nagaratnam M.	Malays J Pathol. 2005 Jun;27(1):33-7.
Identification of a novel 74-kiloDalton immunodominant antigen of Pythium insidiosum recognized by sera from human patients with pythiosis.	Krajaejun T, Kunakorn M, Pracharktam R, Chongtrakool P, Sathapatayavongs B, Chaiprasert A, Vanittanakom N, Chindamporn A, Mootsikapun P.	J Clin Microbiol. 2006 May;44(5):1674-80.
Audit of birth defects in 34,109 deliveries in a tertiary referral center.	Noraihan MN, See MH, Raja R, Baskaran TP, Symonds EM.	Med J Malaysia. 2005 Oct;60(4):460-8.
Molecular and hematological profiles of hemoglobin EE disease with different forms of alpha-thalassemia.	Fucharoen G, Trithipsombat J, Sirithawee S, Yamsri S, Changtrakul Y, Sanchaisuriya K, Fucharoen S.	Ann Hematol. 2006 Jul;85(7):450-4.
Acute glomerulonephritis in beta-thalassemia/haemoglobin E patients: a summary on Thai cases.	Wiwanitkit V.	Nephrology (Carlton). 2006 Feb;11(1):79. No abstract available.
Association between bone mineral density and erythropoiesis in Thai children and adolescents with thalassemia syndromes.	Mahachoklertwattana P, Pootrakul P, Chuansumrit A, Choubtum L, Sriphrapradang A, Sirisriro R, Rajatanavin R.	J Bone Miner Metab. 2006;24(2):146-52.
Brain pathology in a syndrome of hypertension, convulsion, and cerebral haemorrhage in thalassaemic patients after multiple blood-transfusions: a summary in reported Thai autopsy cases.	Wiwanitkit V.	J Hypertens. 2006 Mar;24(3):601. No abstract available.
Antioxidant in plasma of hemoglobin-E trait.	Palasuwan A, Kittisakulrat T, Amornrit W, Soogarun S, Wiwanitkit V, Pradniwat P.	Southeast Asian J Trop Med Public Health. 2005;36 Suppl 4:271-3.
Enhanced maturation and proliferation of beta-thalassemia/Hb E erythroid precursor cells in culture.	Kittikalayawong Y, Sila-asna M, Bunyaratvej A.	Southeast Asian J Trop Med Public Health. 2005 Sep;36(5):1298-303.
Predicting fetal anemia by using reference centile charts for liver length, spleen perimeter and umbilical vein maximum flow velocity in Thai fetuses throughout gestation.	Chawanpaiboon S, Titapant V, Sutantawibul A, Kanokpongsakdi S, Kangkagate C.	J Obstet Gynaecol Res. 2005 Dec;31(6):547-51.
Title	Description	Details
Heterogeneity in alpha-thalassemia interactions in Malays, Chinese and Indians in Malaysia.	Wee YC, Tan KL, Chow TW, Yap SF, Tan JA.	J Obstet Gynaecol Res. 2005 Dec;31(6):540-6.
Diagnosis and management of alpha thalassemia disorders.	Lorey F.	Ann N Y Acad Sci. 2005;1054:514-5. No abstract available.
Genetic analysis of candidate modifier polymorphisms in Hb E-beta 0-thalassemia patients.	Sripichai O, Whitacre J, Munkongdee T, Kumkhaek C, Makarasara W, Winichagoon P, Abel K, Braun A, Fucharoen S.	Ann N Y Acad Sci. 2005;1054:433-8.
Alpha-thalassemia: Hb H disease and Hb Barts hydrops fetalis.	Chui DH.	Ann N Y Acad Sci. 2005;1054:25-32. Review.

Changing patterns of thalassemia worldwide.	Vichinsky EP.	Ann N Y Acad Sci. 2005;1054:18-24. Review.
[Successful preimplantation genetic diagnosis for alpha-thalassemia using fluorescent polymerase chain reaction].	Deng J, Peng WL, Liu Y, Zhou CQ, Li J, Fang C, Lin WQ, Zhuang GL, Zeng YH, Tong DY.	Zhonghua Yi Xue Za Zhi. 2005 Oct 12;85(38):2682-5. Chinese.
Common origin of a rare beta-globin initiation codon mutation (ATG-->AGG) in Asians.	Viprakasit V, Chinchang W, Suwanthol L, Tanphaichitr VS.	Clin Lab Haematol. 2005 Dec;27(6):409-15.
Restrictive lung disease and serum TGF-beta1 in thalassemia major children.	Sritippayawan S, Lekhanont P, Harnruthakorn C, Samransamruajkit R, Deerojanawong J, Seksarn P, Poovorawan Y, Prapphal N.	Asian Pac J Allergy Immunol. 2005 Jun-Sep;23(2-3):121-6.
High frequency of alpha-thalassemia in the So ethnic group of South Laos.	Sengchanh S, Sanguansermsri T, Horst D, Horst J, Flatz G.	Acta Haematol. 2005;114(3):164-6. No abstract available.
Neonatal anemia associated with Southeast Asian ovalocytosis.	Laosombat V, Dissaneevate S, Wongchanchailert M, Satayasevanaa B.	Int J Hematol. 2005 Oct;82(3):201-5.
Compound heterozygote state for GgammaAgamma(deltabeta) degrees -thalassemia and hereditary persistence of fetal hemoglobin.	Fucharoen S, Panyasai S, Surapot S, Fucharoen G, Sanchaisuriya K.	Am J Hematol. 2005 Oct;80(2):119-23.
High incidence of 3-thalassemia, hemoglobin E, and glucose-6-phosphate dehydrogenase deficiency in populations of malaria-endemic southern Shan State, Myanmar.	Than AM, Harano T, Harano K, Myint AA, Ogino T, Okadaa S.	Int J Hematol. 2005 Aug;82(2):119-23.
Molecular analysis of unknown beta-globin gene mutations using polymerase chain reaction-single strand conformation polymorphism (PCR-SSCP) technique and its application in Thai families with beta-thalassemias and beta-globin variants.	Chinchang W, Viprakasit V, Pung-Amritt P, Tanphaichitr VS, Yenchitsomanus PT.	Clin Biochem. 2005 Nov;38(11):987-96.
A rare association of alphaO-thalassemia (--SEA) and an initiation codon mutation (ATG-->A-G) of the alpha2 gene causes Hb H disease in Thailand.	Viprakasit V, Chinchang W, Glomglao W, Tanphaichitr VS.	Hemoglobin. 2005;29(3):235-40.
Genetic disorders in a paediatric hospital in Cambodia.	Guhadasan R, Pises N.	Ann Hum Biol. 2005 Mar-Apr;32(2):188-94.
Alpha-thalassaemia and population health in Southeast Asia.	Chui DH.	Ann Hum Biol. 2005 Mar-Apr;32(2):123-30. Review.
Hemoglobin E prevalence in malaria-endemic villages in Myanmar.	Win N, Lwin AA, Oo MM, Aye KS, Soe S, Okada S.	Acta Med Okayama. 2005 Apr;59(2):63-6.
Modeling for tertiary structure of globin chain in Hemoglobin Suan-Dok disorder.	Wiwanitkit V.	Hematology. 2005 Apr;10(2):163-5.

Pregnancy outcome of hemoglobin H disease.	Tantiweerawong N, Jaovisidha A, Israngura Na Ayudhya N.	Int J Gynaecol Obstet. 2005 Sep;90(3):236-7. No abstract available.
Compound heterozygote states for Hb C/Hb Malay and Hb C/Hb E in pregnancy: a molecular and hematological analysis.	Fucharoen S, Fucharoen G, Sanchaisuriya K, Surapot S.	Blood Cells Mol Dis. 2005 Sep-Oct;35(2):196-200.
The spectrum of beta-globin gene mutations in children with beta-thalassaemia major from Kota Kinabalu, Sabah, Malaysia.	Thong MK, Soo TL.	Singapore Med J. 2005 Jul;46(7):340-3.
Characterisation of beta-globin gene mutations in Malaysian children: a strategy for the control of beta-thalassaemia in a developing country.	Thong MK, Tan JA, Tan KL, Yap SF.	J Trop Pediatr. 2005 Dec;51(6):328-33.
Risks of seroconversion of hepatitis B, hepatitis C and human immunodeficiency viruses in children with multitransfused thalassaemia major.	Lee WS, Teh CM, Chan LL.	J Paediatr Child Health. 2005 May-Jun;41(5-6):265-8.
Molecular and clinical features of Hb H disease in northern Thailand.	Charoenkwan P, Taweephon R, Sae-Tung R, Thanarattanakorn P, Sanguansermsri T.	Hemoglobin. 2005;29(2):133-40.
Case report post-splenectomy sepsis in thalassemic patients.	Zarina AL, Hamidah A, Zulkifli SZ, Jamal R.	Southeast Asian J Trop Med Public Health. 2005 Mar;36(2):471-3.
Hemoglobin E levels in double heterozygotes of hemoglobin E and SEA-type alpha-thalassemia.	Charoenkwan P, Wanapirak C, Thanarattanakorn P, Sekararithi R, Sae-Tung R, Sittipreechacharn S, Sanguansermsri T.	Southeast Asian J Trop Med Public Health. 2005 Mar;36(2):467-70.
Abnormalities in lung function among multiply-transfused thalassemia patients: results from a thalassemia center in Malaysia.	Jamal R, Baizura J, Hamidah A, Idris N, Jeffrey AH, Roslan H.	Southeast Asian J Trop Med Public Health. 2005 Jan;36(1):265-9.
Prevalence of thalassemia in pregnant women at Maharaj Nakorn Chiang Mai Hospital.	Wanapirak C, Muninthorn W, Sanguansermsri T, Dhananjayanonda P, Tongsong T.	J Med Assoc Thai. 2004 Dec;87(12):1415-8.
Thalassemia intermedia associated with complex interaction of Hb Beijing [alpha16(A14)Lys-->Asn] and Hb E [beta26(B8)Glu-->Lys] with a deletional alpha-thalassemia-1 in a Thai family.	Fucharoen S, Chunpanich S, Sanchaisuriya K, Fucharoen G, Kunyanone N.	Hemoglobin. 2005;29(1):77-83.
The Southeast Asian 12.5 KB (delta-beta) degrees-deletion: a common beta-thalassemia in Mon-Khmer groups (Lao Theung) of South Laos.	Sanguansermsri T, Sengchanh S, Horst D, Eigel A, Flatz G, Horst J.	Haematologica. 2004 Aug;89(8):ECR29.
Molecular and hematological characterization of hemoglobin Hope/hemoglobin E and hemoglobin	Chunpanich S, Fucharoen S, Sanchaisuriya K, Fucharoen G, Kam-itsara K.	Lab Hematol. 2004;10(4):215-20.

Hope/alpha-thalassemia 2 in Thai patients.		
Association of Hb Q-Thailand with homozygous Hb E and heterozygous Hb Constant Spring in pregnancy.	Sanchaisuriya K, Chunpanich S, Fucharoen S, Fucharoen G, Sanchaisuriya P, Changtrakun Y.	Eur J Haematol. 2005 Mar;74(3):221-7.
Laboratory investigations of Hb Constant Spring.	Tangvarasittichai O, Jeenapongsa R, Sitthiworanan C, Sanguansermsri T.	Clin Lab Haematol. 2005 Feb;27(1):47-9.
Study on molecular epidemiology of the alpha-thalassemias in Liuzhou City, Guangxi Autonomous Region, China.	Cai R, Liu J, Wang L, Liang X, Xiao B, Su L, Zhou Y, Pan L.	Hemoglobin. 2004;28(4):325-33.
Hemoglobin H disease classification by isoelectric focusing: molecular verification of 110 cases from Thailand.	Sutcharitchan P, Wang W, Settapiboon R, Amornsiriwat S, Tan AS, Chong SS.	Clin Chem. 2005 Mar;51(3):641-4. No abstract available.
Etiology and outcome of non-immune hydrops fetalis in southern Thailand.	Suwanrath-Kengpol C, Kor-anantakul O, Suntharasaj T, Leetanaporn R.	Gynecol Obstet Invest. 2005;59(3):134-7.
Comparison of cost effectiveness between measuring the serum erythropoietin level and reticulocyte count for monitoring thalassemic patients: a note in Thai beta thalassemia/Hb E subjects.	Wiwanitkit V.	Hematology. 2004 Aug;9(4):311-3.
Molecular defects in the beta-globin gene identified in different ethnic groups/populations during prenatal diagnosis for beta-thalassemia: a Malaysian experience.	Tan JA, George E, Tan KL, Chow T, Tan PC, Hassan J, Chia P, Subramanium R, Chandran R, Yap SF.	Clin Exp Med. 2004 Dec;4(3):142-7.
A molecular epidemiologic study of thalassemia using newborns' cord blood in a multiracial Asian population in Singapore: results and recommendations for a population screening program.	Kham SK, Quah TC, Loong AM, Tan PL, Fraser A, Chong SS, Yeoh AE.	J Pediatr Hematol Oncol. 2004 Dec;26(12):817-9. Erratum in: J Pediatr Hematol Oncol. 2005 Jun;27(6):345. Yin, Shirley Kham Kow [corrected to Kham, SK]; Chuan, Samuel Chong Siong [corrected to Chong, SS]; Eng-Juh, Allen Yeoh [corrected to Yeoh, AE].
Generation of transgenic mice expressing human hemoglobin E.	Chen Q, Bouhassira EE, Besse A, Suzuka SM, Fabry ME, Nagel RL, Hirsch RE.	Blood Cells Mol Dis. 2004 Nov-Dec;33(3):303-7.
Hematologic problems in immigrants from Southeast Asia.	Jeng MR, Vichinsky E.	Hematol Oncol Clin North Am. 2004 Dec;18(6):1405-22, x. Review.
Laboratory diagnosis of a compound heterozygosity for Hb Hekinan [alpha27(B8) Glu-Asp] and a deletional alpha-thalassaemia 2 in Thailand.	Chunpanich S, Ayukarn K, Sanchaisuriya K, Fucharoen G, Fucharoen S.	Clin Lab Haematol. 2004 Oct;26(5):355-8.
The 'hot-spot' of Hb E [beta26(B8)Glu->Lys] in Southeast Asia: beta-globin	Flatz G, Sanguansermsri T, Sengchanh S, Horst D, Horst J.	Hemoglobin. 2004 Aug;28(3):197-204.

anomalies in the Lao Theung population of southern Laos.		
Hemoglobin Pakse: presence on red blood cell membrane and detection by polymerase chain reaction-single-strand conformational polymorphism.	Turbpaiboon C, Siritantikorn A, Thongnoppakhun W, Bunditworapoom D, Limwongse C, Yenchitsomanus PT, Siritanaratkul N, Wilairat P.	Int J Hematol. 2004 Aug;80(2):136-9.
Homogeneity of beta(0)-thalassemia codon 17 (A-->T) alleles in Northern Thailand using a direct DNA sequencing method.	Sanguansermsri P, Shimbhu D, Wongvilairat R, Saetung R, Sanguansermsri T.	J Med Assoc Thai. 2004 Aug;87(8):883-6.
Rapid detection of common southeast Asian beta-thalassemia mutations by nonisotopic multiplex PCR-SSCP analysis.	Yip SP, Fung LF, Lo ST.	Genet Test. 2004 Summer;8(2):104-8.
Erythrocyte osmotic fragility test for screening of alpha-thalassemia-1 and beta-thalassemia trait in pregnancy.	Sirichotiyakul S, Tantipalakorn C, Sanguansermsri T, Wanapirak C, Tongsong T.	Int J Gynaecol Obstet. 2004 Sep;86(3):347-50.
A simplified screening strategy for thalassaemia and haemoglobin E in rural communities in south-east Asia.	Fucharoen G, Sanchaisuriya K, Sae-ung N, Dangwibul S, Fucharoen S.	Bull World Health Organ. 2004 May;82(5):364-72.
Haemoglobin E in pregnancy. A case report. Diagnosis, familiar study and counselling, follow up until delivery and new-born observation.	Gessoni G, Bergamini L, Giacomini A, Valverde S, Armani M, Antico F, Trevisan S, Vettorato F, Sartori R.	Minerva Ginecol. 2004 Apr;56(2):155-9.
Molecular basis and hematologic characterization of deltabeta-thalassemia and hereditary persistence of fetal hemoglobin in Thailand.	Panyasai S, Fucharoen S, Surapot S, Fucharoen G, Sanchaisuriya K.	Haematologica. 2004 Jul;89(7):777-81.
Overview of molecular deletion patterns of alpha0-thalassemia in Thailand.	Wiwanitkit V.	Lab Hematol. 2004;10(2):122-3. No abstract available.
Identification of Hb Q-India (alpha64 Asp-->His) in Thailand.	Viprakasit V, Chinchang W, Pung-Amritt P, Tanphaichitr VS.	Hematology. 2004 Apr;9(2):151-5.
Prevalence of HFE mutations among the Thai population and correlation with iron loading in haemoglobin E disorder.	Viprakasit V, Vathesathokit P, Chinchang W, Tachavanich K, Pung-Amritt P, Wimhurst VL, Yenchitsomanus PT, Merryweather-Clarke AT, Tanphaichitr VS.	Eur J Haematol. 2004 Jul;73(1):43-9.
Two cases of compound heterozygosity for Hb Hekinan [alpha27(B8)Glu-->Asp (alpha1)] and alpha-thalassemia in Thailand.	Ngiwsara L, Srisomsap C, Winichagoon P, Fucharoen S, Svasti J.	Hemoglobin. 2004 May;28(2):145-50.
Thalassemia screening among Royal Thai Army medical cadets.	Nathalang O, Arnutti P, Nillakupt K.	Mil Med. 2004 Apr;169(4):274-6.
Prevention of thalassemia: experiences from Samui Island.	Sangkitporn S, Chongkitivitya N, Pathompanichratana S, Sangkitporn SK, Songkharm B, Watanapocha U, Pathtong W.	J Med Assoc Thai. 2004 Feb;87(2):204-12. Review.

The diverse molecular basis and hematological features of Hb H and AEBart's diseases in Northeast Thailand.	Boonsa S, Sanchaisuriya K, Fucharoen G, Wiangnon S, Jetsrisuparb A, Fucharoen S.	Acta Haematol. 2004;111(3):149-54.
Unusual phenotype of hemoglobin EE with hemoglobin H disease: a pitfall in clinical diagnosis and genetic counseling.	Viprakasit V, Tanphaichitr VS.	J Pediatr. 2004 Mar;144(3):391-3.
Co-inheritance of Hb Pak Num Po, a novel alpha1 gene mutation, and alpha0 thalassemia associated with transfusion-dependent Hb H disease.	Viprakasit V, Tanphaichitr VS, Veerakul G, Chinchang W, Petrarat S, Pung-Amritt P, Higgs DR.	Am J Hematol. 2004 Mar;75(3):157-63.
Prevention and control of thalassemia at Saraburi Regional Hospital.	Chareonkul P, Kraisin J.	J Med Assoc Thai. 2004 Jan;87(1):8-15.
Isolated erythrocyte membranes of transfusion-dependent and non-transfused thalassemia patients in Jakarta, investigated by electron paramagnetic resonance spectroscopy.	Udyaningsih-Freisleben SK, Kurniati V, Prasetyo PB, Handayani S, Adhiyanto C, Soegianto RR, Pudiantari R, Munthe BG, Ramelan W, Freisleben HJ.	Biofactors. 2003;19(1-2):87-100.
Iron status and oxidative stress in beta-thalassemia patients in Jakarta.	Laksmitawati DR, Handayani S, Udyaningsih-Freisleben SK, Kurniati V, Adhiyanto C, Hidayat J, Kusnandar S, Dillon HS, Munthe BG, Wirawan R, Soegianto RR, Ramelan W, Freisleben HJ.	Biofactors. 2003;19(1-2):53-62.
A community-based thalassemia prevention and control model in northern Thailand.	Pansatiankul B, Saisorn S.	J Med Assoc Thai. 2003 Aug;86 Suppl 3:S576-82.
Spontaneous mutation of the hemoglobin Leiden (beta 6 or 7 Glu->0) in a Thai girl.	Sanguansermsri P, Shimbhu D, Wongvilairat R, Pimsorn C, Sanguansermsri T.	Haematologica. 2003 Dec;88(12):ECR35.
A thalassaemia array for Southeast Asia.	Chan K, Wong MS, Chan TK, Chan V.	Br J Haematol. 2004 Jan;124(2):232-9.
Prevalence and clinical significance of hepatitis C virus infection in Thai patients with thalassemia.	Wanachiwanawin W, Luengrojanakul P, Sirangkapracha P, Leowattana W, Fucharoen S.	Int J Hematol. 2003 Nov;78(4):374-8.
Human pythiosis, a rare cause of arteritis: case report and literature review.	Prasertwitayakij N, Louthrenoo W, Kasitanon N, Thamprasert K, Vanittanakom N.	Semin Arthritis Rheum. 2003 Dec;33(3):204-14. Review.
Testing the "malaria hypothesis" for the case of Thailand: a genetic appraisal.	Poolsuwan S.	Hum Biol. 2003 Aug;75(4):585-605.
Rapid diagnosis of alpha(o)-thalassemia using the relative quantitative PCR and the dissociation curve analysis.	Sangkitporn SK, Wangkahat K, Sangnoi A, Songkharm B, Charoenporn P, Sangkitporn S.	Clin Lab Haematol. 2003 Dec;25(6):359-65.
Fetal cells in maternal blood: state of the art for non-invasive prenatal diagnosis.	Ho SS, O'Donoghue K, Choolani M.	Ann Acad Med Singapore. 2003 Sep;32(5):597-603; quiz 604. Review.

Interaction of hemoglobin E and several forms of alpha-thalassemia in Cambodian families.	Fucharoen S, Sanchaisuriya K, Fucharoen G, Panyasai S, Devenish R, Luy L.	Haematologica. 2003 Oct;88(10):1092-8.
Rapid, simultaneous genotyping of five common Southeast Asian beta-thalassemia mutations by multiplex minisequencing and denaturing HPLC.	Yip SP, Pun SF, Leung KH, Lee SY.	Clin Chem. 2003 Oct;49(10):1656-9. No abstract available.
Hematological and molecular characterization of beta-thalassemia/Hb Tak compound heterozygote.	Charoenkwan P, Thanarattanakorn P, Chaovaluksakul S, Sittipreechacharn S, Sae-Tang R, Sanguansermsri T.	Southeast Asian J Trop Med Public Health. 2003 Jun;34(2):415-9.
Using reticulocyte indices to identify alpha-thalassemia--a preliminary report.	Soogarun S, Suwansaksri J, Wiwanitkit V.	Southeast Asian J Trop Med Public Health. 2002;33 Suppl 3:159-63.
Molecular and hematologic features of hemoglobin E heterozygotes with different forms of alpha-thalassemia in Thailand.	Sanchaisuriya K, Fucharoen G, Sae-ung N, Jetsrisuparb A, Fucharoen S.	Ann Hematol. 2003 Oct;82(10):612-6.
Red cell indices and therapeutic trial of iron in diagnostic work-up for anemic Thai females.	Nuchprayoon I, Sukthawee B, Nuchprayoon T.	J Med Assoc Thai. 2003 Jun;86 Suppl 2:S160-9.
Alpha thalassaemia in Indonesia: phenotypes and molecular defects.	Setianingsih I, Harahap A, Nainggolan IM.	Adv Exp Med Biol. 2003;531:47-56. Review. No abstract available.
Homozygous alpha-thalassemia treated with intrauterine transfusions and postnatal hematopoietic stem cell transplantation.	Thornley I, Lehmann L, Ferguson WS, Davis I, Forman EN, Guinan EC.	Bone Marrow Transplant. 2003 Aug;32(3):341-2. Review. No abstract available.
Mild beta-thalassemia intermedia caused by compound heterozygosity for (G)gamma((A)gammadeltabeta)(o)/beta-thalassemia and molecular characterization of the defect in four Chinese families.	Tan Jin Ai MA, Yap SF, Tan KL, Wong YC, Wee YC, Kok JL.	Acta Haematol. 2003;109(4):169-75.
The global distribution of length polymorphisms of the promoters of the glucuronosyltransferase 1 gene (UGT1A1): hematologic and evolutionary implications.	Premawardhena A, Fisher CA, Liu YT, Verma IC, de Silva S, Arambepola M, Clegg JB, Weatherall DJ.	Blood Cells Mol Dis. 2003 Jul-Aug;31(1):98-101.
Clinical trial of deferiprone iron chelation therapy in beta-thalassaemia/haemoglobin E patients in Thailand.	Pootrakul P, Sirankapracha P, Sankote J, Kachintorn U, Maungsub W, Sriphen K, Thakernpol K, Atisuk K, Fucharoen S, Chantraluksri U, Shalev O, Hoffbrand AV.	Br J Haematol. 2003 Jul;122(2):305-10.
Molecular characterization of hereditary persistence of fetal hemoglobin in the Karen people of Thailand.	Trachoo O, Sura T, Sakuntabhai A, Singhasivanon P, Krudsood S, Phimpraphi W, Krasaesub S, Chanjarunee S, Looareesuwan S.	Hemoglobin. 2003 May;27(2):97-104.

Analysis of beta-thalassemia mutations in northern Thailand using an automated fluorescence DNA sequencing technique.	Sirichotiyakul S, Saetung R, Sanguansermsri T.	Hemoglobin. 2003 May;27(2):89-95.
The validation of an automated liquid chromatography system for the diagnosis of thalassemias and hemoglobinopathies.	Sangkitporn S, Pung-amritt P, Sangkitporn SK, Sangnoi A, Tanphaichitr VS.	Southeast Asian J Trop Med Public Health. 2002 Dec;33(4):862-8.
Screening for the carriers of thalassemias and abnormal hemoglobins at the community level.	Winichagoon P, Thitivichianlert A, Lebnak T, Piankijagum A, Fucharoen S.	Southeast Asian J Trop Med Public Health. 2002;33 Suppl 2:145-50.
Effect of nutrition support on immunity in paediatric patients with beta-thalassaemia major.	Tienboon P.	Asia Pac J Clin Nutr. 2003;12(1):61-5.
Multiple minisequencing screen for seven southeast Asian nondeletional alpha-thalassemia mutations.	Wang W, Ma ES, Chan AY, Chui DH, Chong SS.	Clin Chem. 2003 May;49(5):800-3. No abstract available.
Complex interaction of Hb Hekinan [alpha27(B8) Glu-Asp] and Hb E [beta26(B8) Glu-Lys] with a deletional alpha-thalassemia 1 in a Thai family.	Fucharoen S, Changtrakun Y, Ratanasiri T, Fucharoen G, Sanchaisuriya K.	Eur J Haematol. 2003 May;70(5):304-9.
Family functioning in children with thalassemia.	Thanarattanakorn P, Louthrenoo O, Sittipreechacharn S, Sanguansermsri T.	Clin Pediatr (Phila). 2003 Jan-Feb;42(1):79-82. No abstract available.
Multiplex minisequencing screen for common Southeast Asian and Indian beta-thalassemia mutations.	Wang W, Kham SK, Yeo GH, Quah TC, Chong SS.	Clin Chem. 2003 Feb;49(2):209-18.
Hemoglobin electrophoresis in Thai non-anemic pregnant subjects, a need for additional screening for hemoglobin E.	Paritpokee N, Wiwanitkit V, Suwansaksri J.	Clin Chem Lab Med. 2002 Nov;40(11):1176-7. No abstract available.
Genetic variants of beta-globin gene in Thai malaria patients.	Naka I, Ohashi J, Patarapotikul J, Hananantachai H, Looareesuwan S, Tokunaga K.	Southeast Asian J Trop Med Public Health. 2003;34 Suppl 2:29-31.
Serum erythropoietin levels in pediatric patients with beta-thalassemia/hemoglobin E.	Paritpokee N, Wiwanitkit V, Bhokaisawan N, Boonchalermvichian C, Preechakas P.	Clin Lab. 2002;48(11-12):631-4.
Thalassemia mutations and their clinical aspects in Japan.	Hattori Y.	Int J Hematol. 2002 Aug;76 Suppl 2:90-2. Review.
Stem cell transplantation for thalassemia.	Issaragrisil S.	Int J Hematol. 2002 Aug;76 Suppl 1:307-9. Review.
Some hematological problems in Indonesia.	Timan IS, Aulia D, Atmakusma D, Sudoyo A, Windiastuti E, Kosasih A.	Int J Hematol. 2002 Aug;76 Suppl 1:286-90.
Marked increase in serum transferrin receptor among Thai children with Hb-E-beta-thalassaemia.	Boonchalermvichian C, Paritpokee N, Bhokaisawan N, Nuchprayoon I, Wiwanitkit V.	J Paediatr Child Health. 2002 Dec;38(6):601-3.
Molecular characterization of Hb D-Punjab [beta121(GH4)Glu-->Gln] in Thailand.	Fucharoen S, Changtrakun Y, Surapot S, Fucharoen G, Sanchaisuriya K.	Hemoglobin. 2002 Aug;26(3):261-9.

Hb G-Makassar [beta6(A3)Glu-->Ala; codon 6 (GAG-->GCG)]: molecular characterization, clinical, and hematological effects.	Viprakasit V, Wiriyasateinkul A, Sattayasevana B, Miles KL, Laosombat V.	Hemoglobin. 2002 Aug;26(3):245-53.
Hb PaksÃ© [(alpha2) codon 142 (TAA-->TAT or Term-->Tyr)J in Thai patients with EAbart's disease and Hb H Disease.	Sanchaisuriya K, Fucharoen G, Fucharoen S.	Hemoglobin. 2002 Aug;26(3):227-35.
Early detection of cardiac involvement in beta-thalassemia children.	Durongpisitkul K, Kruasukon S, Kangkagate C, Tanphaichitr VS.	J Med Assoc Thai. 2002 Aug;85 Suppl 2:S667-73.
Psychosocial problems in children with thalassemia and their siblings.	Louthrenoo O, Sittipreechacharn S, Thanarattanakorn P, Sanguansermsri T.	J Med Assoc Thai. 2002 Aug;85(8):881-5.
Combined one-tube osmotic fragility (OF) test and dichlorophenol-indolphenol (DCIP) test screening for hemoglobin disorders, an experience in 213 Thai pregnant women.	Wiwanitkit V, Suwansaksri J, Paritpokee N.	Clin Lab. 2002;48(9-10):525-8.
Molecular and hematological characterization of HPFH-6/Indian deletion-inversion Ggamma(Agammadeltabeta)0-thalassemia and Ggamma(Agammadeltabeta)0-thalassemia/HbE in Thai patients.	Fucharoen S, Pengjam Y, Surapot S, Fucharoen G, Sanchaisuriya K.	Am J Hematol. 2002 Oct;71(2):109-13.
Molecular analysis of beta-thalassemia in South Vietnam.	Svasti S, Hieu TM, Munkongdee T, Winichagoon P, Van Be T, Van Binh T, Fucharoen S.	Am J Hematol. 2002 Oct;71(2):85-8.
Rapid confirmation of Southeast Asian and Filipino alpha-thalassemia genotypes from newborn screening specimens.	Bhardwaj U, Zhang YH, Blackburn W, McCabe LL, McCabe ER.	Am J Hematol. 2002 Sep;71(1):56-8.
A simplified screening for alpha-thalassemia 1 (SEA type) using a combination of a modified osmotic fragility test and a direct PCR on whole blood cell lysates.	Panyasai S, Sringam P, Fucharoen G, Sanchaisuriya K, Fucharoen S.	Acta Haematol. 2002;108(2):74-8.
Compound heterozygosity for Hb Korle-Bu (beta(73); Asp-Asn) and Hb E (beta(26); Glu-Lys) with a 3.7-kb deletional alpha-thalassemia in Thai patients.	Changtrakun Y, Fucharoen S, Ayukarn K, Siriratmanawong N, Fucharoen G, Sanchaisuriya K.	Ann Hematol. 2002 Jul;81(7):389-93.
Hemoglobin E: a balanced polymorphism protective against high parasitemias and thus severe P falciparum malaria.	Chotivanich K, Udomsangpetch R, Pattanapanyasat K, Chierakul W, Simpson J, Looareesuwan S, White N.	Blood. 2002 Aug 15;100(4):1172-6.
Compound heterozygosity for alpha0-thalassemia (- -THAI) and Hb constant spring causes severe Hb H disease.	Viprakasit V, Tanphaichitr VS.	Hemoglobin. 2002 May;26(2):155-62.

Thalassemia major.	Peh WC.	Am J Orthop (Belle Mead NJ). 2002 Jun;31(6):320. No abstract available.
A wider molecular spectrum of beta-thalassaemia in Myanmar.	Win N, Harano T, Harano K, Myint TT, Mra R, Okada S, Shimono K, Myint AA.	Br J Haematol. 2002 Jun;117(4):988-92.
The use of the amplification refractory mutation system (arms) in the detection of rare beta-thalassemia mutations in the Malays and Chinese in Malaysia.	Chan Yoke Fan, Tan Kim Lian, Wong Yean Ching, Wee Yong Chui, Yap Sook Fan, Tan Jin Ai Mary Anne.	Southeast Asian J Trop Med Public Health. 2001 Dec;32(4):872-9.
Complex interactions of deltabeta hybrid haemoglobin (Hb Lepore-Hollandia) Hb E (beta(26G-->A)) and alpha+ thalassaemia in a Thai family.	Viprakasit V, Pung-Amritt P, Suwanthon L, Clark K, Tanphaichtr VS.	Eur J Haematol. 2002 Feb;68(2):107-11.
Beta-thalassemia major in Malaysia, an ongoing public health problem.	George E.	Med J Malaysia. 2001 Dec;56(4):397-400. No abstract available.
PCR-based analysis of alpha-thalassemia in Southern Taiwan.	Chen TP, Liu TC, Chang CS, Chang JG, Tsai HJ, Lin SF.	Int J Hematol. 2002 Apr;75(3):277-80.
Short stature and truncal shortening in transfusion dependent thalassemia patients: results from a thalassemia center in Malaysia.	Hamidah A, Rahmah R, Azmi T, Aziz J, Jamal R.	Southeast Asian J Trop Med Public Health. 2001 Sep;32(3):625-30.
Hb Siam [alpha15(A13)Gly-->Arg (alpha1) (GGT-->CGT)] is a typical alpha chain hemoglobinopathy without an alpha-thalassemic effect.	Turbpaiboon C, Svasti S, Sawangareetakul P, Winichagoon P, Srisomsap C, Siritanaratkul N, Fucharoen S, Wilairat P, Svasti J.	Hemoglobin. 2002 Feb;26(1):77-81. No abstract available.
[Rapid detection of alpha-thalassemia of Southeast Asian deletion by polymerase chain reaction and its application to prenatal diagnosis].	Xiao W, Xu X, Liu Z.	Zhonghua Xue Ye Xue Za Zhi. 2000 Apr;21(4):192-4. Chinese.
Molecular analysis of a thai beta-thalassaemia heterozygote with normal haemoglobin A2 level: implication for population screening.	Fucharoen S, Fucharoen G, Sanchaisuriya K, Pengjam Y.	Ann Clin Biochem. 2002 Jan;39(Pt 1):44-9.
Association of homozygous alpha-thalassaemia of the Southeast Asian type with hypospadias: still an intriguing enigma.	Utsch B, Hansmann M, Albers N, Lentze MJ, Bidlingmaier F, Ludwig M.	Fetal Diagn Ther. 2002 Mar-Apr;17(2):127-8. No abstract available.
Clinical phenotypes and molecular characterization of Hb H-PaksÃ© disease.	Viprakasit V, Tanphaichitr VS, Pung-Amritt P, Petrarat S, Suwantol L, Fisher C, Higgs DR.	Haematologica. 2002 Feb;87(2):117-25.
Association of Hb Hope [beta136(H14)Gly-->Asp] and Hb H disease.	Svasti S, Yodsowon B, Sriphanich R, Winichagoon P, Boonkhan P, Suwanban T, Sawangareetrakul P, Srisomsap C, Ketudat-Cairns JR, Svasti J, Fucharoen S.	Hemoglobin. 2001 Nov;25(4):429-35. No abstract available.
Interaction of the alpha2 polyadenylation signal mutation (AATAAA-->AATA--) and alpha0-	Laosombat V, Fucharoen S, Wiriyasateinkul A.	Hemoglobin. 2001 Nov;25(4):383-9.

thalassemia (--SEA), resulting in Hb H disease in a Thai patient.		
Hb Dartmouth [alpha66(E15)Leu-->Pro (alpha2) (CTG-->CCG)]: a novel alpha2-globin gene mutation associated with severe neonatal anemia when inherited in trans with Southeast Asian alpha-thalassemia-1.	McBride KL, Snow K, Kubik KS, Fairbanks VF, Hoyer JD, Fairweather RB, Chaffee S, Edwards WH.	Hemoglobin. 2001 Nov;25(4):375-82.
Major hematologic diseases in the developing world- new aspects of diagnosis and management of thalassemia, malarial anemia, and acute leukemia.	Greenberg PL, Gordeuk V, Issaragrisil S, Siritanaratkul N, Fucharoen S, Ribeiro RC.	Hematology Am Soc Hematol Educ Program. 2001:479-98. Review.
Clinical and hematological features of beta(+)-thalassemia (IVS-1 nt 5, G-C mutation) in Thai patients.	Laosombat V, Wongchanchailert M, Sattayasevana B, Wiriyasateinkul A, Fucharoen S.	Eur J Haematol. 2001 Aug;67(2):100-4.
Real-time quantitative PCR analysis for alpha-thalassemia-1 of Southeast Asian type deletion in Taiwan.	Sun CF, Lee CH, Cheng SW, Lin MH, Wu TL, Tsao KC, Chiu DT, Liou JD, Chu DC.	Clin Genet. 2001 Oct;60(4):305-9.
Molecular analysis of beta-thalassemia in South Vietnam.	Le TH, Pissard S, Pham HV, Lacombe C, Truong DH, Goossens M, Truong DK.	Hemoglobin. 2001 Aug;25(3):305-9.
Genetic counseling for thalassemia in Thailand: problems and solutions.	Dhamcharee V, Romyanan O, Ninlagarn T.	Southeast Asian J Trop Med Public Health. 2001 Jun;32(2):413-8.
Universal newborn screening for Hb H disease in California.	Lorey F, Cunningham G, Vichinsky EP, Lubin BH, Witkowska HE, Matsunaga A, Azimi M, Sherwin J, Eastman J, Farina F, Waye JS, Chui DH.	Genet Test. 2001 Summer;5(2):93-100.
Thalassemia syndromes in Saudi Arabia. Meta-analysis of local studies.	Al-Awamy BH.	Saudi Med J. 2000 Jan;21(1):8-17.
Living a 'normal' life: young people coping with thalassaemia major or sickle cell disorder.	Atkin K, Ahmad WI.	Soc Sci Med. 2001 Sep;53(5):615-26.
Atypical hemoglobin H disease in a Thai patient resulting from a combination of alpha-thalassemia 1 and hemoglobin Constant Spring with hemoglobin J Bangkok heterozygosity.	Fucharoen S, Ayukarn K, Sanchaisuriya K, Fucharoen G.	Eur J Haematol. 2001 May;66(5):312-6.
Molecular characterization of thalassemia intermedia with homozygous Hb Malay and Hb Malay/HbE in Thai patients.	Fucharoen S, Sanchaisuriya K, Fucharoen G, Surapot S.	Haematologica. 2001 Jun;86(6):657-8. No abstract available.
Application of ribosomal RNA gene restriction patterns analysis and pulsed-field gel electrophoresis in distinguishing Salmonella weltevreden isolates in Malaysia.	Goh YL, Puthucheary SD, Thong KL.	Southeast Asian J Trop Med Public Health. 2000 Dec;31(4):697-701.

Detection and structural analysis of abnormal hemoglobins found in Thailand.	Svasti J, Srisomsap C, Winichagoon P, Fucharoen S.	Southeast Asian J Trop Med Public Health. 1999;30 Suppl 2:88-93.
A reliable screening test to identify adult carriers of the (--SEA) alpha zero-thalassemia deletion. Detection of embryonic zeta-globin chains by enzyme-linked immunosorbent assay.	Lafferty JD, Crowther MA, Waye JS, Chui DH.	Am J Clin Pathol. 2000 Dec;114(6):927-31.
Combine-ARMS: a rapid and cost-effective protocol for molecular characterization of beta-thalassemia in Malaysia.	Tan KL, Tan JA, Wong YC, Wee YC, Thong MK, Yap SF.	Genet Test. 2001 Spring;5(1):17-22.
Should we screen for globin gene mutations in blood samples with mean corpuscular volume (MCV) greater than 80 fL in areas with a high prevalence of thalassaemia?	Chan LC, Ma SK, Chan AY, Ha SY, Waye JS, Lau YL, Chui DH.	J Clin Pathol. 2001 Apr;54(4):317-20.
Prenatal diagnosis of beta-thalassemia major by high-performance liquid chromatography analysis of hemoglobins in fetal blood samples.	Sanguansermsri T, Thanarattanakorn P, Steger HF, Tongsong T, Chanprapaph P, Wanpirak C, Siriwatanapa P, Sirichotiyakul S, Flatz G.	Hemoglobin. 2001 Feb;25(1):19-27.
Prevention and control of thalassemia in Ramathibodi Hospital, Thailand.	Jaovisidha A, Ajjimarkorn S, Panburana P, Somboonsub O, Herabutya Y, Rungsiprakarn R.	Southeast Asian J Trop Med Public Health. 2000 Sep;31(3):561-5.
Intrahepatic stones: the UKM experience.	Din J, Qureshi A, Daud A, Ahmad H.	Med J Malaysia. 2000 Dec;55(4):473-7.
Clinical and hematologic features of beta0-thalassemia (frameshift 41/42 mutation) in Thai patients.	Laosombat V, Wongchanchailert M, Sattayasevana B, Wiriyasateinkul A, Fucharoen S.	Haematologica. 2001 Feb;86(2):138-41.
Prenatal eradication of Hb Bart's hydrops fetalis.	Tongsong T, Wanapirak C, Sirivatanapa P, Sa-nguansermsri T, Sirichotiyakul S, Piyamongkol W, Chanprapaph P, Steger HF, Sekararithi R, Tuggapichitti A.	J Reprod Med. 2001 Jan;46(1):18-22.
[Genetic diagnosis of alpha and beta thalassemia dual heterozygote].	Zeng R, Yu S, Hu B.	Zhonghua Xue Ye Xue Za Zhi. 1998 Oct;19(10):525-7. Chinese.
Non-radioactive Southern hybridization for early diagnosis of alpha-thalassemia with southeast Asian-type deletion in Taiwan.	Chu DC, Lee CH, Lo MD, Cheng SW, Chen DP, Wu TL, Tsao KC, Chiu DT, Sun CF.	Am J Med Genet. 2000 Dec 11;95(4):332-5.
Clinical and hematological features of codon 17, A-T mutation of beta-thalassemia in Thai patients.	Laosombat V, Wongchanchailert M, Sattayasevana B, Wiriyasateinkul A, Fucharoen S.	Eur J Haematol. 2001 Feb;66(2):126-9.
Severe terminal transverse limb reduction defects in homozygous Southeast-Asian alpha-thalassaemia-1.	Chen CP.	Clin Dysmorphol. 2001 Jan;10(1):71-3.
Two thalassemia intermedia patients with delta beta/beta-thalassemia and a deletional type alpha-thalassemia.	Li Z, Liu L, Zhao Y, Zhong X, Xu X.	Haematologica. 2001 Jan;86(1):108. No abstract available.

Title	Description	Details
Outreach strategies for Southeast Asian communities: experience, practice, and suggestions for approaching Southeast Asian immigrant and refugee communities to provide thalassemia education and trait testing.	Choy J, Foote D, Bojanowski J, Yamashita R, Vichinsky E.	J Pediatr Hematol Oncol. 2000 Nov-Dec;22(6):588-92.
Asian immigration and public health in California: thalassemia in newborns in California.	Lorey F.	J Pediatr Hematol Oncol. 2000 Nov-Dec;22(6):564-6.
Clinical manifestation of beta-thalassemia/hemoglobin E disease.	Fucharoen S, Ketvichit P, Pootrakul P, Siritanaratkul N, Piankijagum A, Wasi P.	J Pediatr Hematol Oncol. 2000 Nov-Dec;22(6):552-7.
Introduction to the problem of hemoglobin E-beta thalassemia.	Weatherall DJ.	J Pediatr Hematol Oncol. 2000 Nov-Dec;22(6):551. No abstract available.
Simplified multiplex-PCR diagnosis of common southeast asian deletional determinants of alpha-thalassemia.	Chong SS, Boehm CD, Cutting GR, Higgs DR.	Clin Chem. 2000 Oct;46(10):1692-5. No abstract available.
A correlation of erythrokinetics, ineffective erythropoiesis, and erythroid precursor apoptosis in thai patients with thalassemia.	Pootrakul P, Sirankapracha P, Hemsorach S, Moungsub W, Kumbunlue R, Piangitjagum A, Wasi P, Ma L, Schrier SL.	Blood. 2000 Oct 1;96(7):2606-12.
Screening for (--SEA) alpha-globin gene deletion in beta-thalassemia carriers and prevention of hydrops fetalis.	Kwan Ma ES, Yin Chan AY, Yin Ha S, Fung Chan GC, Lung Lau Y, Chan LC.	Haematologica. 2000 Sep;85(9):991-3. No abstract available.
Novel germline BRCA1 mutations detected in women in singapore who developed breast carcinoma before the age of 36 years.	Ho GH, Phang BH, Ng IS, Law HY, Soo KC, Ng EH.	Cancer. 2000 Aug 15;89(4):811-6.
Serum erythropoietin levels in thalassemia major and intermedia.	Chaisiripoomkere W, Jootar S, Chanjarunee S, Ungkanont A.	Southeast Asian J Trop Med Public Health. 1999 Dec;30(4):786-8.
Plan and process for hematology laboratory standard in Thailand.	Bunyaratvej A.	Southeast Asian J Trop Med Public Health. 1999;30 Suppl 3:173-6.
Standardization on laboratory diagnosis of thalassemia and abnormal hemoglobin.	Fucharoen S, Winichagoon P, Piankijagum A.	Southeast Asian J Trop Med Public Health. 1999;30 Suppl 3:90-8. Review.
High prevalence of hepatitis C in patients with thalassemia and patients with liver diseases in Myanmar (Burma).	Okada S, Taketa K, Ishikawa T, Koji T, Swe T, Win N, Win KM, Mra R, Myint TT.	Acta Med Okayama. 2000 Jun;54(3):137-8.
[Fetus with hydrops in a woman from Vietnam].	EggebÃ¸ TM, Smedvig E, Zanussi GF, MÃ¥seide PH, Blaas HG, Sandberg S.	Tidsskr Nor Laegeforen. 2000 Jun 20;120(16):1878-81. Norwegian. No abstract available.
Molecular analysis of beta-thalassemia in Vietnam.	Filon D, Oppenheim A, Rachmilewitz EA, Kot R, Truc DB.	Hemoglobin. 2000 May;24(2):99-104.

Spinal cord compression: a rareness in pregnant thalassemic woman.	Phupong V, Uerpairojkij B, Limpongsanurak S.	J Obstet Gynaecol Res. 2000 Apr;26(2):117-20.
Reticulocyte analysis in iron deficiency anemia and hemolytic anemia.	Butthep P, Wisedpanichkij R, Jindadamrongwech S, Kaewkethong P, Pattamakom S, Sila-Asna M, Bunyaratvej A.	J Med Assoc Thai. 2000 Mar;83 Suppl 1:S114-22.
Etiology and incidence of thrombotic and hemorrhagic disorders in Thai patients with extreme thrombocytosis.	Chuncharunee S, Archararit N, Ungkanont A, Jootar S, Angchaisuksiri P, Bunyaratavej A, Rojanasthein S, Atichartakarn V.	J Med Assoc Thai. 2000 Mar;83 Suppl 1:S95-100.
Extramedullary haematopoietic tumor producing small intestinal intussusception in a beta-thalassemia/hemoglobin E Thai boy: a case report.	Wongwaisayawan S, Pornkul R, Teeraratkul S, Pakakasama S, Treepongkaruna S, Nithiyanant P.	J Med Assoc Thai. 2000 Mar;83 Suppl 1:S17-22.
Parvovirus B19 caused fetal death: a case report in Thailand.	Booncharoen P, Boonpasat Y, Sriurairatana S.	J Med Assoc Thai. 2000 Mar;83 Suppl 1:S12-6.
Clearance of hepatitis TT virus infection among thalassemia children and IVDU.	Vimolket T, Theamboonlers A, Jantaradsamee P, Seksarn P, Hirsch P, Poovorawan Y.	Southeast Asian J Trop Med Public Health. 1999 Jun;30(2):269-72.
HLA class I antibodies in thalassemic patients.	Nathalang O, Torcharus K, Chuansumrit A, Punyaprasiddhi P, Sriphaisal T, Tatsumi N.	Southeast Asian J Trop Med Public Health. 1998 Dec;29(4):801-4.
Prenatal diagnosis of thalassemia in Songklanagarind Hospital in southern Thailand.	Kor-anantakul O, Suwanrath CT, Leetanaporn R, Suntharasaj T, Liabsuetrakul T, Rattanaprueksachart R.	Southeast Asian J Trop Med Public Health. 1998 Dec;29(4):795-800.
Seroprevalence of hepatitis B, hepatitis C, CMV and HIV in multiply transfused thalassemia patients: results from a thalassemia day care center in Malaysia.	Jamal R, Fadzillah G, Zulkifli SZ, Yasmin M.	Southeast Asian J Trop Med Public Health. 1998 Dec;29(4):792-4.
Clinical and hematologic aspects of hemoglobin E beta-thalassemia.	Fucharoen S, Winichagoon P.	Curr Opin Hematol. 2000 Mar;7(2):106-12. Review.
Single-tube multiplex-PCR screen for common deletional determinants of alpha-thalassemia.	Chong SS, Boehm CD, Higgs DR, Cutting GR.	Blood. 2000 Jan 1;95(1):360-2.
PCR-based diagnosis of the Filipino (--(FIL)) and Thai (--(THAI)) alpha-thalassemia-1 deletions.	Eng B, Patterson M, Borys S, Chui DH, Waye JS.	Am J Hematol. 2000 Jan;63(1):54-6.
Hydroxyurea and sodium phenylbutyrate therapy in thalassemia intermedia.	Hoppe C, Vichinsky E, Lewis B, Foote D, Styles L.	Am J Hematol. 1999 Dec;62(4):221-7.
Tubulointerstitial renal failure in childhood leptospirosis.	Vachvanichsanong P, Dissaneewate P, Mitarnun W.	Pediatr Emerg Care. 1999 Oct;15(5):332-4.
[Implication of health checkups of students from developing countries in Japan].	Fujimoto K, Momosaka Y, Uchida K, Ide H, Narisada H, Fujishiro K, Oda S, Okubo T.	Nihon Koshu Eisei Zasshi. 1999 Jun;46(6):476-86. Japanese.
Type 1 diabetes in Thai children aged 0-14 years.	Likitmaskul S, Angsusingha K, Morris S, Kiattisakthavee P, Chaichanwatanakul K, Tuchinda C.	J Med Assoc Thai. 1999 Aug;82(8):826-32.

The future of medicine from the standpoint of the practising paediatrician.	Wong HB.	Ann Acad Med Singapore. 1999 Mar;28(2):299-310. No abstract available.
The precise breakpoints of a Filipino-type alpha-thalassemia-1 deletion found in two Japanese.	Hattori Y, Okayama N, Ohba Y, Yamashiro Y, Yamamoto K, Tsukimoto I, Kohakura M.	Hemoglobin. 1999 Aug;23(3):239-48.
Cure of beta-thalassaemia major by umbilical cord blood transplantation--a case report of Malaysia's first cord blood transplantation.	Chan LL, Lin HP.	J Trop Pediatr. 1999 Aug;45(4):243-5.
Phenotypic variability of Filipino beta(o)-thalassemia/HbE patients in Indonesia.	Setianingsih I, Williamson R, Daud D, Harahap A, Marzuki S, Forrest S.	Am J Hematol. 1999 Sep;62(1):7-12.
Prevalence study and molecular characterization of alpha-thalassemia in Filipinos.	Ko TM, Hwa HL, Liu CW, Li SF, Chu JY, Cheung YP.	Ann Hematol. 1999 Aug;78(8):355-7.
Anemia in Sakon Nakhon Province.	Channgam P, Thumpenjit S, Koowatanasiri P.	J Med Assoc Thai. 1999 Jun;82(6):545-51.
Thalassemia and malaria: new insights into an old problem.	Clegg JB, Weatherall DJ.	Proc Assoc Am Physicians. 1999 Jul-Aug;111(4):278-82. Review.
A single, large deletion accounts for all the beta-globin gene mutations in twenty families from Sabah (North Borneo), Malaysia. Mutation in brief no. 240. Online.	Thong MK, Rudzki Z, Hall J, Tan JA, Chan LL, Yap SF.	Hum Mutat. 1999;13(5):413.
A novel 105 basepair deletion causing beta(0)-thalassemia in members of a Thai family.	Nopparatana C, Saechan V, Nopparatana C, Pornpatkul M, Panich V, Fukumaki Y.	Am J Hematol. 1999 May;61(1):1-4.
Homozygous alpha-thalassaemia and hypospadias--common aetiology or incidental association? Long-term survival of Hb Bart's hydrops syndrome leads to new aspects for counselling of alpha-thalassaemic traits.	Dame C, Albers N, Hasan C, Bode U, Eigel A, Hansmann M, Brenner R, Bartmann P.	Eur J Pediatr. 1999 Mar;158(3):217-20.
Prevalence and molecular characterization of beta-thalassemia in Filipinos.	Ko TM, Caviles AP Jr, Hwa HL, Liu CW, Hsu PM, Chung YP.	Ann Hematol. 1998 Dec;77(6):257-60.
Hepatitis TT virus infection in high-risk groups.	Poovorawan Y, Theamboonlers A, Jantaradsamee P, Kaew-in N, Hirsch P, Tangkitvanich P.	Infection. 1998 Nov-Dec;26(6):355-8.
Influence of hemoglobin E trait on the severity of Falciparum malaria.	Hutagalung R, Wilairatana P, Looareesuwan S, Brittenham GM, Aikawa M, Gordeuk VR.	J Infect Dis. 1999 Jan;179(1):283-6.
Effects of alpha-thalassemia on pharmacokinetics of the antimalarial agent artesunate.	Ittarat W, Looareesuwan S, Pootrakul P, Sumpunsirikul P, Vattanavibool P, Meshnick SR.	Antimicrob Agents Chemother. 1998 Sep;42(9):2332-5.
Antenatal screening and prenatal diagnosis of thalassaemia: an update.	Small KA, Chan FY.	Med J Aust. 1998 Aug 17;169(4):215-9. No abstract available.

Outreach strategies for Asian Pacific Island (API) communities.	Choy J, Yamashita RC, Foote D, Heer N, Vichinsky EP.	Ann N Y Acad Sci. 1998 Jun 30;850:514-5. No abstract available.
Alpha- and beta-thalassemia in Thailand.	Fucharoen S, Winichagoon P, Siritanaratkul N, Chowthaworn J, Pootrakul P.	Ann N Y Acad Sci. 1998 Jun 30;850:412-4. No abstract available.
An alpha-2 globin gene initiation codon mutation in a Vietnamese patient with Hb H disease.	Kutlar F, Adamkiewicz TV, Markowitz RB, Holley L, Kutlar A.	Ann N Y Acad Sci. 1998 Jun 30;850:398-400. No abstract available.
The hemoglobin E syndromes.	Rees DC, Styles L, Vichinsky EP, Clegg JB, Weatherall DJ.	Ann N Y Acad Sci. 1998 Jun 30;850:334-43. Review.
Prenatal diagnosis for thalassaemia in a multicultural society.	Trent RJ, Le H, Yu B.	Prenat Diagn. 1998 Jun;18(6):591-8.
Prevalence of infection with hepatitis G virus among various groups in Thailand.	Poovorawan Y, Theamboonlers A, Chongsrisawat V, Jantaradsamee P.	Ann Trop Med Parasitol. 1998 Jan;92(1):89-95.
Coinheritance of alpha-thalassemia-1 and hemoglobin E/beta zero-thalassemia: practical implications for neonatal screening and genetic counseling.	Krishnamurti L, Chui DH, Dallaire M, LeRoy B, Waye JS, Perentesis JP.	J Pediatr. 1998 May;132(5):863-5.
Molecular basis of beta-thalassemia in the Maldives.	Furuumi H, Firdous N, Inoue T, Ohta H, Winichagoon P, Fucharoen S, Fukumaki Y.	Hemoglobin. 1998 Mar;22(2):141-51.
High prevalence of hepatitis G virus infection in multiply transfused children with thalassaemia.	Poovorawan Y, Theamboonlers A, Chongsrisawat V, Seksarn P, Jarvis L, Simmonds P.	J Gastroenterol Hepatol. 1998 Mar;13(3):253-6.
Prenatal and postnatal diagnoses of thalassemias and hemoglobinopathies by HPLC.	Fucharoen S, Winichagoon P, Wisedpanichkij R, Sae-Ngow B, Sriphanich R, Oncoung W, Muangsapaya W, Chowthaworn J, Kanokpongsakdi S, Bunyaratvej A, Piankijagum A, Dewaele C.	Clin Chem. 1998 Apr;44(4):740-8.
Detection of zeta-globin chains in the cord blood by ELISA (enzyme-linked immunosorbent assay): rapid screening for alpha-thalassemia 1 (Southeast Asian type).	Ausavarungnirun R, Winichagoon P, Fucharoen S, Epstein N, Simkins R.	Am J Hematol. 1998 Apr;57(4):283-6.
Renal function in adult beta-thalassemia/Hb E disease.	Ong-ajyooth L, Malasit P, Ong-ajyooth S, Fucharoen S, Pootrakul P, Vasuvattakul S, Siritanaratkul N, Nilwarangkur S.	Nephron. 1998;78(2):156-61.
Hb Tak confirmed by DNA analysis: not expressed as thalassemia in a Hb Tak/Hb E compound heterozygote.	Hoyer JD, Wick MJ, Thibodeau SN, Viker KA, Conner R, Fairbanks VF.	Hemoglobin. 1998 Jan;22(1):45-52.
Molecular characterization and PCR diagnosis of Thailand deletion of alpha-globin gene cluster.	Ko TM, Tseng LH, Kao CH, Lin YW, Hwa HL, Hsu PM, Li SF, Chuang SM.	Am J Hematol. 1998 Feb;57(2):124-30. Erratum in: Am J Hematol 1999 Feb;60(2):173.
Novel mutation of the alpha 2-globin gene initiation codon (ATG-->A-G) in a Vietnamese girl with Hb H disease.	Waye JS, Eng B, Patterson M, Chui DH, Nisbet-Brown E, Olivieri NF.	Hemoglobin. 1997 Sep;21(5):469-72. No abstract available.

Hemoglobinopathies in Southeast Asia: molecular biology and clinical medicine.	Fucharoen S, Winichagoon P.	Hemoglobin. 1997 Jul;21(4):299-319. Review. No abstract available.
Hemoglobin typing in cholangiocarcinoma.	Insiripong S, Thaisamakr S, Amatachaya C.	Southeast Asian J Trop Med Public Health. 1997 Jun;28(2):424-7.
Prenatal diagnosis of thalassemia in the Chinese.	Liu TC, Lin SF, Yang TY, Lee JP, Chen TP, Chang JG.	Am J Hematol. 1997 Jun;55(2):65-8.
Malaria infection after allogeneic bone marrow transplantation in a child with thalassemia.	Tran VB, Tran VB, Lin KH.	Bone Marrow Transplant. 1997 Jun;19(12):1259-60.
[A rapid method for diagnosing alpha-thalassemia-1 of Southeast Asia type].	Jin Q, Jiang N, Tang X.	Zhonghua Xue Ye Xue Za Zhi. 1997 May;18(5):257-60. Chinese.
The 1996 Runme Shaw Memorial Lecture: malaria--past, present and future.	Warrell DA.	Ann Acad Med Singapore. 1997 May;26(3):380-7. Review.
Beta-thalassaemia in the immigrant and non-immigrant German populations.	Vetter B, Schwarz C, Kohne E, Kulozik AE.	Br J Haematol. 1997 May;97(2):266-72.
Frequency of deletional alpha-thalassemia genotypes in a predominantly Asian-American population.	HofgÃ¤rtner WT, West Keefe SF, Tait JF.	Am J Clin Pathol. 1997 May;107(5):576-81.
The prevalence of hepatitis C virus antibodies in thalassemic patients in the south of Thailand.	Laosombat V, Pornpatkul M, Wongchanchailert M, Worachat K, Wiriyasatienku A.	Southeast Asian J Trop Med Public Health. 1997 Mar;28(1):149-53.
Laboratory and field comparisons of adenosine influx in Plasmodium falciparum and Plasmodium vivax infected erythrocytes with genetic abnormalities from patients in Myanmar.	Myint-Oo, O'Sullivan WJ, Gero AM.	Southeast Asian J Trop Med Public Health. 1997 Mar;28(1):22-31.
The unusual pathobiology of hemoglobin constant spring red blood cells.	Schrier SL, Bunyaratvej A, Khuhapinant A, Fucharoen S, Aljurf M, Snyder LM, Keifer CR, Ma L, Mohandas N.	Blood. 1997 Mar 1;89(5):1762-9.
Fetal erythropoiesis and the diagnosis and treatment of hemoglobin disorders in the fetus and child.	Olivieri NF.	Semin Perinatol. 1997 Feb;21(1):63-9. Review.
Mutation of a highly conserved residue of betaI spectrin associated with fatal and near-fatal neonatal hemolytic anemia.	Gallagher PG, Petruzzi MJ, Weed SA, Zhang Z, Marchesi SL, Mohandas N, Morrow JS, Forget BG.	J Clin Invest. 1997 Jan 15;99(2):267-77.
Difference in pattern of erythropoietin response between beta-thalassemia/hemoglobin E children and adults.	Sukpanichnant S, Opartkiattikul N, Fucharoen S, Tanphaichitr VS, Hasuike T, Tatsumi N.	Southeast Asian J Trop Med Public Health. 1997;28 Suppl 3:134-7.
Analysis of beta-thalassemia mutations and beta-locus control	Sriroongrueng W, Schleiemacher E, Panich V, Nopparatana C, Saechan V, Laosombat V, Pornpatkul M, Fukumaki Y.	Southeast Asian J Trop Med Public Health. 1997;28 Suppl 3:120-7.

region hypersensitive sites 2, 3 and 4 in southern Thailand.		
Clinical, hematological and molecular features in Thais with beta-Malay/beta-thalassemia and beta-Malay/HbE.	Laosombat V, Wongchanchailert M, Sattayesevana B, Nopparatana C.	Southeast Asian J Trop Med Public Health. 1997;28 Suppl 3:106-9.
Molecular basis of beta-thalassemia in Thai Muslim patients in the the south of Thailand.	Laosombat V, Nopparatana C, Wongchanchailert M, Wiriyasateinkul A.	Southeast Asian J Trop Med Public Health. 1997;28 Suppl 3:104-5.
Molecular and hematological characterization of HbE heterozygote with alpha-thalassemia determinant.	Sanchaisuriya K, Fucharoen G, Sae-ung N, Sae-ue N, Baisungneon R, Jetsrisuparb A, Fucharoen S.	Southeast Asian J Trop Med Public Health. 1997;28 Suppl 3:100-3.
Red cell parameters in alpha-thalassemia with and without beta-thalassemia trait or hemoglobin E trait.	Jindadamrongwech S, Wisedpanichkij R, Bunyaratvej A, Hathirat P.	Southeast Asian J Trop Med Public Health. 1997;28 Suppl 3:97-9.
Alpha-thalassemia incidence in southern Thailand by restriction endonuclease analysis of globin DNA from placental blood at Songklanagarind Hospital.	Sriroongrueng W, Pornpatkul M, Panich V, Fucharoen S.	Southeast Asian J Trop Med Public Health. 1997;28 Suppl 3:93-6.
Screening for thalassemia: an economics viewpoint.	Indaratna K.	Southeast Asian J Trop Med Public Health. 1997;28 Suppl 3:75-81.
Pyruvate kinase deficiency in an alpha-thalassemia family: first case report in Thailand.	Tanphaichitr VS, Suvatte V, Mahasandana C, Veerakul G, Pung-amritt P, Tachavanich K, Ideguchi H.	Southeast Asian J Trop Med Public Health. 1997;28 Suppl 3:64-8.
Human genomics: implications for health.	Wasi P.	Southeast Asian J Trop Med Public Health. 1997;28 Suppl 2:19-24.
Human T-lymphotropic virus type II infection in Vietnamese thalassemic patients.	Lin MT, Nguyen BT, Binh TV, Be TV, Chiang TY, Tseng LH, Yang YC, Lin KH, Chen YC.	Arch Virol. 1997;142(7):1429-40.
Early prenatal diagnosis of beta-thalassaemia in Singapore.	Ng IS, Ong JB, Tan CL, Law HY.	Ann Acad Med Singapore. 1996 Nov;25(6):779-82.
A large beta-thalassemia deletion in a family of Indonesian-Malay descent.	Dimovski AJ, Baysal E, Efremov DG, Prior JF, Raven JL, Efremov GD, Huisman TH.	Hemoglobin. 1996 Nov;20(4):377-92.
Prenatal diagnosis of Hb Bart's hydrops fetalis in West Malaysia: the identification of the alpha thal 1 defect by PCR based strategies.	George E, Mokhtar AB, Azman ZA, Hasnida K, Saripah S, Hwang CM.	Singapore Med J. 1996 Oct;37(5):501-4.
Anemia, genetic diseases, and malaria in prehistoric mainland Southeast Asia.	Tayles N.	Am J Phys Anthropol. 1996 Sep;101(1):11-27.
Prevalence of alpha-thalassemias in northern Thailand.	Lemmens-Zygulska M, Eigel A, Helbig B, Sanguansermsri T, Horst J, Flatz G.	Hum Genet. 1996 Sep;98(3):345-7.
Frequency of alpha-thalassemia-1 of the Southeast Asian-type among pregnant women in northern	Kitsirisakul B, Steger HF, Sanguansermsri T.	Southeast Asian J Trop Med Public Health. 1996 Jun;27(2):362-3.

Thailand determined by PCR technique.		
Hematologic disorders in children from southeast Asia.	Glader BE, Look KA.	Pediatr Clin North Am. 1996 Jun;43(3):665-81. Review.
The spectrum of beta-thalassemia mutations in Malays in Singapore and Kelantan.	Abdullah WA, Jamaluddin NB, Kham SK, Tan JA.	Southeast Asian J Trop Med Public Health. 1996 Mar;27(1):164-8.
New frameshift mutation, insertion of A, at codon 95 of the beta-globin gene causes beta-thalassemia in two Vietnamese families.	Cai S, Chehab FF.	Hum Mutat. 1996;8(3):293-4. No abstract available.
Molecular heterogeneity of beta-thalassaemia in Malaysia: a practical approach to diagnosis.	Thong MK, Law HY, Ng IS.	Ann Acad Med Singapore. 1996 Jan;25(1):79-83.
Production of monoclonal and polyclonal antibodies against various haemoglobins for the detection of thalassaemias.	Gan YY, Lai CM, Lee SH, Gan LH.	Ann Acad Med Singapore. 1996 Jan;25(1):75-8. Review.
Clinical problem-solving. If at first you don't succeed.	Ross JM, Sox HC.	N Engl J Med. 1995 Dec 7;333(23):1557-60. No abstract available.
Alpha-thalassaemia and globin gene rearrangements in French Polynesia.	Philippon G, Martinson JJ, Rugless MJ, Moulia-Pelat JP, Plichart R, Roux JF, Martin PM, Clegg JB.	Eur J Haematol. 1995 Sep;55(3):171-7.
C to T transition at the first nucleotide of codon 63 of the beta-globin gene corresponding to hemoglobin M-Saskatoon in an Indonesian boy.	Suryantoro P, Takeshima Y, Haryanto A, Matsuo M.	Jpn J Hum Genet. 1995 Jun;40(2):195-201.
Detection of alpha-thalassemia-1 (Southeast Asian type) and its application for prenatal diagnosis.	Winichagoon P, Fucharoen S, Kanokpongsakdi S, Fukumaki Y.	Clin Genet. 1995 Jun;47(6):318-20.
[Hemoglobinopathies. Current therapeutic possibilities].	Birgens HS, Karle H.	Ugeskr Laeger. 1995 May 29;157(22):3179-85. Danish.
Intrauterine therapy for homozygous alpha-thalassemia.	Carr S, Rubin L, Dixon D, Star J, Dailey J.	Obstet Gynecol. 1995 May;85(5 Pt 2):876-9.
Symmetrical terminal transverse limb defects: report of a twenty-week fetus.	Harmon JV Jr, Osathanondh R, Holmes LB.	Teratology. 1995 Apr;51(4):237-42. Review.
Recurrent fatal hydrops fetalis associated with a nucleotide substitution in the erythrocyte beta-spectrin gene.	Gallagher PG, Weed SA, Tse WT, Benoit L, Morrow JS, Marchesi SL, Mohandas N, Forget BG.	J Clin Invest. 1995 Mar;95(3):1174-82.
[Thalassemic alleles in Mexican mestizos].	Ibarra B, Perea FJ, Villalobos-ArÃ¡mbula AR.	Rev Invest Clin. 1995 Mar-Apr;47(2):127-31. Spanish.
Genetic red cell disorders and severity of falciparum malaria in Myanmar.	Oo M, Tin-Shwe, Marlar-Than, O'Sullivan WJ.	Bull World Health Organ. 1995;73(5):659-65.
Problems of genetic diseases and their services in Indonesia: a country report.	Sofro AS.	Southeast Asian J Trop Med Public Health. 1995;26 Suppl 1:5-8. No abstract available.

Prenatal diagnosis of Hb Bart's hydrops fetalis by PCR technique: Pramongkutklao experience.	Torcharus K, Sriphaisal T, Krutvecho T, Ketupanya A, Vuthiwong C, Suwanasophon C, Noonai A.	Southeast Asian J Trop Med Public Health. 1995;26 Suppl 1:287-90.
Prenatal diagnosis for beta-thalassemia syndromes using HRP-labeled oligonucleotide probes at Siriraj Hospital.	Winichagoon P, Fucharoen S, Siritanaratkul N, Tassana P, Thonglairoam V, Siriboon W, Kanokpongsakdi S.	Southeast Asian J Trop Med Public Health. 1995;26 Suppl 1:282-6.
Clinical phenotypes and genotypes diagnosis of thalassemia in children.	Torcharus K, Sriphaisal T, Krutvecho T, Suwanasophon C, Intarapakawong J.	Southeast Asian J Trop Med Public Health. 1995;26 Suppl 1:275-7.
Prevalence of hemoglobin E, alpha-thalassemia and glucose-6-phosphate dehydrogenase deficiency in 1,000 cord bloods studied in Bangkok.	Tanphaichitr VS, Mahasandana C, Suvatte V, Yodthong S, Pung-amritt P, Seeloem J.	Southeast Asian J Trop Med Public Health. 1995;26 Suppl 1:271-4.
Phitsanulok population: the highest incidence of hemoglobin E in the northern provinces of Thailand and PND counseling.	Pravatmuang P, Tiloklurs M, Suannum M, Chaipat C.	Southeast Asian J Trop Med Public Health. 1995;26 Suppl 1:266-70.
Medical genetics in Thailand.	Sirinavin C.	Southeast Asian J Trop Med Public Health. 1995;26 Suppl 1:26-33. Review.
Molecular analysis of Hb Q-H disease and Hb Q-Hb E in a Singaporean family.	Tan J, Tay JS, Wong YC, Kham SK, Bte Abd Aziz N, Teo SH, Wong HB.	Southeast Asian J Trop Med Public Health. 1995;26 Suppl 1:252-6.
Molecular basis of alpha (0)-thalassemia in northeast of Thailand.	Fucharoen G, Fucharoen S, Wanhakit C, Srithong W.	Southeast Asian J Trop Med Public Health. 1995;26 Suppl 1:249-51.
Molecular mechanisms of thalassemia in southeast Asia.	Winichagoon P, Fucharoen S, Wilairat P, Fukumaki Y.	Southeast Asian J Trop Med Public Health. 1995;26 Suppl 1:235-40.
The spectrum of beta-thalassemia mutations in southern Thailand.	Nopparatana C, Panich V, Saechan V, Sriroongrueng V, Nopparatana C, Rungjeadpha J, Pornpatkul M, Laosombat V, Fukumaki Y.	Southeast Asian J Trop Med Public Health. 1995;26 Suppl 1:229-34.
The clinical severity of beta-thalassemia mutations in West Malaysia.	George E.	Southeast Asian J Trop Med Public Health. 1995;26 Suppl 1:225-8.
Molecular pathology of beta-thalassemia in Indonesia.	Sofro AS.	Southeast Asian J Trop Med Public Health. 1995;26 Suppl 1:221-4. No abstract available.
Genetic problems and genetic services in Malaysia: a country report.	Hassan K.	Southeast Asian J Trop Med Public Health. 1995;26 Suppl 1:11-5. No abstract available.
Filipino beta-thalassemia due to a large deletion: identification of the deletion endpoints and polymerase chain reaction (PCR)-based diagnosis.	Waye JS, Eng B, Hunt JA, Chui DH.	Hum Genet. 1994 Nov;94(5):530-2.
The amplification refractory mutation system (ARMS): a rapid and direct	Tan JA, Tay JS, Lin LI, Kham SK, Chia JN, Chin TM, Aziz NB, Wong HB.	Prenat Diagn. 1994 Nov;14(11):1077-82.

prenatal diagnostic technique for beta-thalassaemia in Singapore.		
Rapid detection of -alpha 4.2 deletion of alpha-thalassemia-2 by polymerase chain reaction.	Chang JG, Liu TC, Chiou SS, Chen JT, Chen TP, Lin CP.	Ann Hematol. 1994 Oct;69(4):205-9.
Beta-thalassemia mutations in Singapore--a strategy for prenatal diagnosis.	Ng IS, Ong JB, Tan CL, Law HY.	Hum Genet. 1994 Oct;94(4):385-8.
A simple non-radioactive assay for hemoglobin E gene in prenatal diagnosis.	Fucharoen S, Fucharoen G, Ratanasiri T, Jetsrisuparb A, Fukumaki Y.	Clin Chim Acta. 1994 Sep;229(1-2):197-203. No abstract available.
Rapid and simultaneous non-radioactive method for detecting alpha-thalassemia 1 (SEA type) and Hb Constant Spring genes.	Fucharoen G, Fucharoen S.	Eur J Haematol. 1994 Sep;53(3):186-7. No abstract available.
Carrier screening and prenatal diagnosis for alpha-thalassemia with biphasic polymerase chain reaction.	Kuo PL, Lin TM, Huang KF, Yeh NG, Wu DB, Wu RC, Tzeng CC, Wu HL, Huang KE.	J Formos Med Assoc. 1994 Sep;93(9):765-9.
Bone marrow transplantation in Thailand.	Issaragrisil S, Visuthisakchai S, Suvatte V, Chandanayingyong D, Piankijagum A.	Transplant Proc. 1994 Aug;26(4):2348-9. No abstract available.
Human pythiosis in Thailand.	Imwidthaya P.	Postgrad Med J. 1994 Aug;70(826):558-60. Review.
Nondeletional type of hereditary persistence of fetal haemoglobin: molecular characterization of three unrelated Thai HPFH.	Winichagoon P, Fucharoen S, Wilairat P, Chihara K, Fukumaki Y.	Br J Haematol. 1994 Aug;87(4):797-804.
Thalassaemia in Malaysia: a strategy for prevention.	Ainoon O, Cheong SK.	Malays J Pathol. 1994 Jun;16(1):23-7.
Molecular basis of beta-thalassemia minor in Taiwan.	Chang JG, Lin CP, Liu TC, Chiou SS, Chen PH, Lee LS, Chen TP.	Int J Hematol. 1994 Jun;59(4):267-72.
Molecular basis and hematological characterization of Hb H disease in southeast Asia.	Liu TC, Chiou SS, Lin SF, Chen TP, Tseng WP, Chen PH, Chang JG.	Am J Hematol. 1994 Apr;45(4):293-7.
Rapid detection of deletions causing delta beta thalassemia and hereditary persistence of fetal hemoglobin by enzymatic amplification.	Craig JE, Barnetson RA, Prior J, Raven JL, Thein SL.	Blood. 1994 Mar 15;83(6):1673-82.
Allelic stability of VNTR locus 3' alpha HVR: linkage disequilibrium with the common alpha-thalassaemia-1 deletion of South-East Asia (--SEA/).	Waye JS, Eng B.	Hum Hered. 1994 Mar-Apr;44(2):61-7.
A novel deletion of approximately 27 kb including the beta-globin gene and the locus control region 3'HS-1 regulatory sequence: beta zero-thalassemia or hereditary persistence of fetal hemoglobin?	Dimovski AJ, Divoky V, Adekile AD, Baysal E, Wilson JB, Prior JF, Raven JL, Huisman TH.	Blood. 1994 Feb 1;83(3):822-7.

Alpha 2-globin gene mutation Hb G-Waimanalo: occurrence in combination with alpha-thalassemia-1.	Landin B, Berg P.	Hemoglobin. 1994 Jan;18(1):71-2. No abstract available.
Rapid molecular characterization of Hb H disease in Chinese by polymerase chain reaction.	Chang JG, Liu TC, Perng LI, Chiou SS, Chen TP, Chen PH, Lin CP.	Ann Hematol. 1994 Jan;68(1):33-7.
Etiologies, consequences, and treatment of iron overload.	Gordeuk VR, McLaren GD, Samowitz W.	Crit Rev Clin Lab Sci. 1994;31(2):89-133. Review.
Cord blood transplantation in thalassemia.	Issaragrisil S.	Blood Cells. 1994;20(2-3):259-62; discussion 263. Review.
Beta-thalassaemia mutations in west Malaysia: a new thalassaemia clinical score system.	George E.	Ann Acad Med Singapore. 1994 Jan;23(1):89-93.
Molecular characterization of beta-thalassaemia in Singaporean Chinese: application to prenatal diagnosis.	Tan JA, Tay JS, Kham S, Chin TM, Wong HB, Lai PS.	J Paediatr Child Health. 1993 Dec;29(6):461-3.
Hb E beta +-thalassaemia in west Malaysia: clinical features in the most common beta-thalassaemia mutation of the Malays [IVS 1-5 (G-->C)].	George E, Wong HB.	Singapore Med J. 1993 Dec;34(6):500-3.
Immunocytological test to detect adult carriers of (--SEA/) deletional alpha-thalassaemia.	Tang W, Luo HY, Eng B, Waye JS, Chui DH.	Lancet. 1993 Nov 6;342(8880):1145-7.
Perinatal case report of unexpected thalassemia Hb Bart.	Hofstaetter C, Gonser M, Goelz R.	Fetal Diagn Ther. 1993 Nov-Dec;8(6):418-22.
Prevalences of thalassemia/hemoglobinopathies and G-6-PD deficiency in malaria patients.	Insiripong S, Tulayalak P, Amatachaya C.	J Med Assoc Thai. 1993 Oct;76(10):554-8.
HTLV-I antibody screening in donated blood and thalassemic patients.	Chiewsilp P, Iamsilp W, Hathirat P.	J Med Assoc Thai. 1993 Oct;76 Suppl 2:103-5.
[Diagnosis of hemoglobinopathies and thalassemias].	Brosstad F, Stavem P, Kofstad J, Gran T.	Tidsskr Nor Laegeforen. 1993 Sep 30;113(23):2925-8. Norwegian.
Hemoglobin E and codon 17 nonsense: two beta-globin gene mutations common in Southeast Asia detected by the use of ARMS.	Steger H, Eigel A, Flatz G, Horst J.	Ann Hematol. 1993 Sep;67(3):119-20.
Detection of the (--SEA) double alpha-globin gene deletion by a simple immunologic assay for embryonic zeta-globin chains.	Ireland JH, Luo HY, Chui DH, Chu B, Yuen J, Hsia YE.	Am J Hematol. 1993 Sep;44(1):22-8.
BamH1 polymorphism in the Chinese, Malays, and Indians in Singapore and its application in the prenatal diagnosis of beta-thalassemia.	Tan JA, Tay SH, Kham KY, Wong HB.	Jpn J Hum Genet. 1993 Sep;38(3):315-8.
Donor for BMT with haemoglobin H disease.	Peltier JY, Girault D, Debre M, Galacteros F, Fischer A, Girot R.	Bone Marrow Transplant. 1993 Jul;12(1):81-4.

Cultural and psychosocial considerations in screening for thalassemia in the Southeast Asian refugee population.	Nidorf JF, Ngo KY.	Am J Med Genet. 1993 Jun 1;46(4):398-402.
Beta 0-thalassemia due to a cytosine deletion in codon 41 associated with hemoglobin E.	Petmitr S, Fucharoen S, Rajkulchai P, Bifani JP, Wilairat P.	Southeast Asian J Trop Med Public Health. 1993 Jun;24(2):384-5. No abstract available.
Filipino beta zero thalassaemia: a high Hb A2 beta zero thalassaemia resulting from a large deletion of the 5' beta globin gene region.	Motum PI, Kearney A, Hamilton TJ, Trent RJ.	J Med Genet. 1993 Mar;30(3):240-4.
[Reversible spinal cord compression caused by extramedullary hematopoietic foci in thalassemia].	Massenkeil G, Wichmann W, Krummenacher F, Rhyner K.	Dtsch Med Wochenschr. 1993 Jan 29;118(4):100-6. German.
Bone marrow transplantation for thalassemia in Thailand.	Issaragrisil S, Visudhisakchai S, Suvatte V, Chandanayingyong D, Piankijagum A, Mahasandana C, Tanphaichitr VS.	Bone Marrow Transplant. 1993;12 Suppl 1:42-4.
Identification of two novel beta zero-thalassemia mutations in a Filipino family: frameshift codon 67 (-TG) and a beta-globin gene deletion.	Eng B, Chui DH, Saunderson J, Olivieri NF, Waye JS.	Hum Mutat. 1993;2(5):375-9.
Molecular characterization of Hb H disease by polymerase chain reaction.	Chen TP, Lin SF, Chang JG, Tsao CJ, Liu TC, Chiou SS, Liu HW.	Acta Haematol. 1993;90(4):177-81.
HIV seroprevalence in hematologic patients other than hemophiliacs at Ramathibodi Hospital.	Mahaphan W, Hathirat P, Isarangkura P, Chuansumrit A, Atnaseo P, Panthangkura W, Chiewsilp P.	Southeast Asian J Trop Med Public Health. 1993;24 Suppl 1:187-90.
Alpha thalassaemia hydrops fetalis in the UK: the importance of screening pregnant women of Chinese, other South East Asian and Mediterranean extraction for alpha thalassaemia trait.	Petrou M, Brugiatelli M, Old J, Hurley P, Ward RH, Wong KP, Rodeck C, Modell B.	Br J Obstet Gynaecol. 1992 Dec;99(12):985-9.
Role of lipid peroxidation and antioxidants in aging process and thalassemia.	Chanarat N.	Kitasato Arch Exp Med. 1992 Dec;65(4):245-9. Review.
Thalassemia in SouthEast Asia: problems and strategy for prevention and control.	Fucharoen S, Winichagoon P.	Southeast Asian J Trop Med Public Health. 1992 Dec;23(4):647-55. Review.
Molecular basis of beta thalassemia in the south of Thailand.	Laosombat V, Fucharoen SP, Panich V, Fucharoen G, Wongchanchailert M, Sriroongrueng W, Nopparatana C, Kenpitak K, Maipang M, Fukumaki Y.	Am J Hematol. 1992 Nov;41(3):194-8.
Hemoglobin H disease in children.	Wongchanchailert M, Laosombat V, Maipang M.	J Med Assoc Thai. 1992 Nov;75(11):611-8.
Rapid prenatal diagnosis of Hb Bart's hydrops fetalis in southeast Asia area by polymerase chain reaction.	Chang JG, Chen CP, Ho HJ, Lin CP, Lee LS, Chen PH.	Int J Hematol. 1992 Oct;56(2):155-9.
Identification of five rare mutations including a novel frameshift mutation	Winichagoon P, Fucharoen S, Wilairat P, Chihara K, Fukumaki Y, Wasi P.	Biochim Biophys Acta. 1992 Aug 25;1139(4):280-6.

causing beta zero-thalassemia in Thai patients with beta zero-thalassemia/hemoglobin E disease.		
The spectrum of beta thalassaemia in Burma.	Brown JM, Thein SL, Weatherall DJ, Mar KM.	Br J Haematol. 1992 Aug;81(4):574-8.
Thalassaemia and haemoglobinopathies in Brunei Darussalam.	Ismail JB.	Med J Malaysia. 1992 Jun;47(2):98-102.
Thalassemia in the outpatient department of the Yangon Children's Hospital in Myanmar: cost analysis of the day-care-room services for thalassemia.	Aung Myo Han, Khin Ei Han, Thein Thein Myint.	Southeast Asian J Trop Med Public Health. 1992 Jun;23(2):273-7.
Thalassemia in the outpatient department of the Yangon Children's Hospital in Myanmar: knowledge, attitudes and practice in relation to thalassemia.	Khin Ei Han, Aung Myo Han, Thein Thein Myint.	Southeast Asian J Trop Med Public Health. 1992 Jun;23(2):269-72.
Thalassemia in the outpatient department of the Yangon Children's Hospital in Myanmar: basic hematological values of thalassemia traits.	Khin Ei Han, Aung Myo Han, Kyaw Win, Thein Thein Myint.	Southeast Asian J Trop Med Public Health. 1992 Jun;23(2):264-8.
From molecular medicine to public health.	Kidson C.	Southeast Asian J Trop Med Public Health. 1992 Jun;23(2):175-6. No abstract available.
Hydrops fetalis caused by severe alpha-thalassemia.	Hsieh FJ, Ko TM, Chen HY.	Early Hum Dev. 1992 Jun-Jul;29(1-3):233-6.
A PCR-based strategy to detect the common severe determinants of alpha thalassaemia.	Bowden DK, Vickers MA, Higgs DR.	Br J Haematol. 1992 May;81(1):104-8.
Hereditary anemias in Hawaii: an update.	Hsia YE.	Hawaii Med J. 1992 Jan;51(1):17, 19. No abstract available.
Carrier detection and prenatal diagnosis of alpha-thalassemia of Southeast Asian deletion by polymerase chain reaction.	Ko TM, Tseng LH, Hsieh FJ, Hsu PM, Lee TY.	Hum Genet. 1992 Jan;88(3):245-8.
Types of thalassemia among patients attending a large university clinic in Kuala Lumpur, Malaysia.	George E, Li HJ, Fei YJ, Reese AL, Baysal E, Cepreganova B, Wilson JB, Gu LH, Nechtman JF, Stoming TA, et al.	Hemoglobin. 1992;16(1-2):51-66.
Deletional types of alpha-thalassaemia in central Java.	Tan JA, Tay JS, Soemantri A, Kham SK, Wong HB, Lai PS, Saha N.	Hum Hered. 1992;42(5):289-92.
Different cell volume with high target cell population between liver disease and homozygous hemoglobin E.	Kaewketthong P, Bunyaratvej A, Barusrux S.	J Med Assoc Thai. 1992 Jan;75 Suppl 1:228-32.
Thalassemic red cells determined by different technology of blood cell analysers.	Bunyaratvej A, Buthachat S, Ratanasritong S, Chitagatgone S.	J Med Assoc Thai. 1992 Jan;75 Suppl 1:223-7.

Molecular heterogeneity of beta-thalassemia in Thailand.	Fukumaki Y, Fucharoen S, Fucharoen G, Okamoto N, Ichinose M, Jetsrisuparb A, Sriroongrueng W, Nopparatana C, Laosombat V, Panich V, et al.	Southeast Asian J Trop Med Public Health. 1992;23 Suppl 2:14-21.
Biophysical changes of red cells with thalassemia-like abnormal hemoglobin.	Bunyaratvej A, Fucharoen S, Tatsumi N.	Southeast Asian J Trop Med Public Health. 1992;23 Suppl 2:86-90.
Size distribution curves of blood cells in thalassemias and hemoglobin H diseases.	Tatsumi N, Tsuda I, Funahara Y, Matsumoto H, Bunyaratvej A, Siritanakul N, Fucharoen S.	Southeast Asian J Trop Med Public Health. 1992;23 Suppl 2:79-85.
The molecular basis of alpha-thalassemia in Thailand.	Winichagoon P, Fucharoen S, Wasi P.	Southeast Asian J Trop Med Public Health. 1992;23 Suppl 2:7-13.
Protein C and protein S deficiency in thalassemic patients.	Shirahata A, Funahara Y, Opartkiattikul N, Fucharoen S, Laosombat V, Yamada K.	Southeast Asian J Trop Med Public Health. 1992;23 Suppl 2:65-73.
Detection of PF3 availability in whole blood from volunteers and beta-thalassemia/HbE patients: a promising method for prediction of thrombotic tendency.	Opartkiattikul N, Funahara Y, Hijikata-Okunomiya A, Yamaguchi N, Fucharoen S, Talalak P.	Southeast Asian J Trop Med Public Health. 1992;23 Suppl 2:52-9.
Spontaneous platelet aggregation in thalassemic children and adolescents.	Laosombat V, Wongchanchailert M, Kenpitak K, Wisitpongpon C.	Southeast Asian J Trop Med Public Health. 1992;23 Suppl 2:42-6.
Increase in spontaneous platelet aggregation in beta-thalassemia/hemoglobin E disease: a consequence of splenectomy.	Opartkiattikul N, Funahara Y, Fucharoen S, Talalak P.	Southeast Asian J Trop Med Public Health. 1992;23 Suppl 2:36-41.
Pathogenesis of hypoxemia.	Chantharaksri U, Tonsuwonnont W, Fucharoen S, Wasi P.	Southeast Asian J Trop Med Public Health. 1992;23 Suppl 2:32-5.
Pulmonary thromboembolism in thalassemic patients.	Sonakul D, Fucharoen S.	Southeast Asian J Trop Med Public Health. 1992;23 Suppl 2:25-8. Review.
Liver tissue injury secondary to iron overload in beta-thalassemia/hemoglobin E disease.	Thakerngpol K, Fucharoen S, Sumiyoshi A, Stitnimankarn T.	Southeast Asian J Trop Med Public Health. 1992;23 Suppl 2:110-5.
Interaction between endothelial cells and thalassemic red cells in vitro.	Butthep P, Bunyaratvej A, Kitaguchi H, Funahara Y, Fucharoen S.	Southeast Asian J Trop Med Public Health. 1992;23 Suppl 2:101-4.
The problem of thalassemia in Thailand.	Panich V, Pornpatkul M, Sriroongrueng W.	Southeast Asian J Trop Med Public Health. 1992;23 Suppl 2:1-6.
Automatic measurement of hemoglobin F in blood obtained from patients with hemoglobin E/E and beta-thalassemia/hemoglobin/E.	Tatsumi N, Tsuda I, Funahara Y, Bunyaratvej A, Fucharoen S.	Southeast Asian J Trop Med Public Health. 1992;23 Suppl 2:91-4.
Hemoglobin constant spring in Bangkok: molecular screening by	Thonglairoam V, Winichagoon P, Fucharoen S, Tanphaichitr VS, Pung-amritt P, Embury SH, Wasi P.	Am J Hematol. 1991 Dec;38(4):277-80.

selective enzymatic amplification of the alpha 2-globin gene.		
Double heterozygosity of the beta-Malay and a novel beta-thalassemia gene in a Thai patient.	Fucharoen S, Fucharoen G, Laosombat V, Fukumaki Y.	Am J Hematol. 1991 Oct;38(2):142-4.
The pattern of thalassemia in children at the Department of Child Health, School of Medicine University of North Sumatera/Dr. Pirngadi Hospital, Medan.	Sinulingga S, Loebis IF, Sutjipto A.	Paediatr Indones. 1991 Sep-Oct;31(9-10):261-7.
Rapid diagnosis of alpha-thalassemia-1 of southeast Asia type and hydrops fetalis by polymerase chain reaction.	Chang JG, Lee LS, Lin CP, Chen PH, Chen CP.	Blood. 1991 Aug 1;78(3):853-4. No abstract available.
Quantitation of beta-thalassemia genes in Quebec immigrants of Mediterranean, southeast Asian, and Asian Indian origins.	Kaplan F, Kokotsis G, Capua A, Scriver CR.	Clin Invest Med. 1991 Aug;14(4):325-30.
Nutritional status in childhood thalassaemia at the Department of Child Health University of North Sumatera Dr. Pirngadi Hospital Medan.	Loebis IF, Sinulingga S, Sutjipto A.	Paediatr Indones. 1991 May-Jun;31(5-6):141-4.
Hb Fukuyama or alpha 2 beta(2)77(EF1)His----Tyr observed in an Indonesian female.	Smit JW, Deggeller K, Tamminga RY, Wilson JB, Webber BB, Huisman TH.	Hemoglobin. 1991;15(4):331-3. No abstract available.
Detection of the most frequent beta-thalassemic defect in Southeast Asia by PCR.	Sebillon P, Bienvenu T, Girot R, Labie D, Kaplan JC, Beldjord C.	Hemoglobin. 1991;15(4):313-5. No abstract available.
Hb Westmead [alpha 122(H5)His----Gln], Hb E [beta 26(B8)Glu----Lys], and alpha-thalassemia-2 (3.7 Kb deletion) in a Laotian family.	Gu YC, Gu LH, Wilson JB, Cepreganova B, Ramachandran M, Walker EL 3rd, Huisman TH, Potitong P.	Hemoglobin. 1991;15(4):297-302. No abstract available.
Fetal red cell in Thai thalassemia trait patients.	Chiewsilp P, Fucharoen S, Apibal S, Soponsuksatit A.	Acta Haematol. 1991;86(2):116. No abstract available.
Prenatal diagnosis of alpha- and beta-thalassaemias in Singapore--current status.	Tan JA, Tay SH, Siang OK, Lai PS, Boon WH, Kham S.	Ann Trop Paediatr. 1991;11(1):67-74.
Alpha-thalassemia: prenatal diagnosis and neonatal implications.	Maberry MC, Klein VR, Boehm C, Warren TC, Gilstrap LC 3rd.	Am J Perinatol. 1990 Oct;7(4):356-8.
The molecular basis of beta-thalassemia in Thailand: application to prenatal diagnosis.	Thein SL, Winichagoon P, Hesketh C, Best S, Fucharoen S, Wasi P, Weatherall DJ.	Am J Hum Genet. 1990 Sep;47(3):369-75.
Chinese in west Malaysia: the geography of beta thalassaemia mutations.	George-Kodiseri E, Yang KG, Kutlar F, Wilson JB, Kutlar A, Stoming TA, Gonzales-Redondo JM, Huisman TH.	Singapore Med J. 1990 Aug;31(4):374-7.
Prenatal diagnosis of alpha-thalassemia by polymerase chain reaction and dual restriction enzyme analysis.	Lebo RV, Saiki RK, Swanson K, Montano MA, Erlich HA, Golbus MS.	Hum Genet. 1990 Aug;85(3):293-9.

Molecular basis of HbE-beta-thalassemia and the origin of HbE in northeast Thailand: identification of one novel mutation using amplified DNA from buffy coat specimens.	Fucharoen G, Fucharoen S, Jetsrisuparb A, Fukumaki Y.	Biochem Biophys Res Commun. 1990 Jul 31;170(2):698-704.
Southeast Asian immigrants: the new thalassemias in Americans.	Anderson HM, Ranney HM.	Semin Hematol. 1990 Jul;27(3):239-46. Review. No abstract available.
Childhood onset of diabetes mellitus. Report on hospital cases.	Sutan-Assin M, Rukman Y, Batubara JR.	Paediatr Indones. 1990 Jul-Aug;30(7-8):209-12.
Subcutaneous mucormycosis caused by Saksenaea vasiformis in a thalassaemic child: first case report in Thailand.	Tanphaichitr VS, Chaiprasert A, Suvatte V, Thasnakorn P.	Mycoses. 1990 Jun;33(6):303-9.
Cardiac involvement in beta-thalassemia/hemoglobin E disease: clinical and hemodynamic findings.	Jootar P, Fucharoen S.	Southeast Asian J Trop Med Public Health. 1990 Jun;21(2):269-73.
Anaemia in National Service Registrants in Singapore.	Mak KH, Wong HB.	Ann Acad Med Singapore. 1990 May;19(3):359-63.
Abnormality of von Willebrand factor in patients with hemoglobin E-beta (0) thalassemia.	Benson PJ, Peterson LC, Hasegawa DK, Smith CM 2nd.	Am J Clin Pathol. 1990 Mar;93(3):395-9.
Hydrops fetalis due to Bart hemoglobinopathy at Ramathibodi Hospital (1978-1987): a 10-year review.	O-Prasertsawat P, Suthutvoravut S, Chaturachinda K.	J Med Assoc Thai. 1990 Feb;73 Suppl 1:65-8.
Beta-thalassemia/haemoglobin E disease in Vietnam.	Nguyen CK, Le TT, Duong BT, Dinh PH, Ta TH, Tran HH.	J Trop Pediatr. 1990 Feb;36(1):43-5.
Prenatal diagnosis of alpha and beta thalassemias and hemophilia A: experience in Hong Kong.	Chan V.	Clin Biochem. 1990 Feb;23(1):79-84.
Study of alpha-thalassemia in northeastern Thailand at the DNA level.	Hundrieser J, Laig M, Yongvanit P, Sriboonlue P, Sanguansermsri T, KÃ¼hnau W, Pape M, Flatz G.	Hum Hered. 1990;40(2):85-8.
Beta-thalassemia in Thailand.	Winichagoon P, Fucharoen S, Thonglairoam V, Tanapotiwirut V, Wasi P.	Ann N Y Acad Sci. 1990;612:31-42. Review. No abstract available.
Thai G gamma (A gamma delta beta)zero-thalassemia and its interaction with a single gamma-globin gene on a chromosome carrying beta zero-thalassemia.	Winichagoon P, Fucharoen S, Thonglairoam V, Wasi P.	Hemoglobin. 1990;14(2):185-97.
Beta zero-thalassemia in a Thai family is caused by a 3.4 kb deletion including the entire beta-globin gene.	Sanguansermsri T, Pape M, Laig M, Hundrieser J, Flatz G.	Hemoglobin. 1990;14(2):157-68.
Thalassaemia in Singapore.	Wong HB.	J Singapore Paediatr Soc. 1990;32(3-4):53-60. No abstract available.

Molecular characterization and nonradioactive detection of beta-thalassemia in Malaysia.	Fucharoen S, Fucharoen G, Ata K, Aziz S, Hashim S, Hassan K, Fukumaki Y.	Acta Haematol. 1990;84(2):82-8.
Impact of the detection of Î¶-globin chains and hemoglobin Bart's using immunochromatographic strip tests for Î±0-thalassemia (--SEA) differential diagnosis	Pata S, Laopajon W, Pongpaiboon M, Thongkum W, Polpong N, Munkongdee T, Paiboonsukwong K, Fucharoen S, Tayapiwatana C, Kasinrerk W.	PLoS One. 2019 Oct 29;14(10):e0223996. doi: 10.1371/journal.pone.0223996. eCollection 2019.
Molecular characteristics of thalassemia and hemoglobin variants in prenatal diagnosis program in northern Thailand	Mankhemthong K, Phusua A, Suanta S, Srisittipoj P, Charoenkwan P, Sanguansermsri T.	Int J Hematol. 2019 Oct;110(4):474-481. doi: 10.1007/s12185-019-02694-y. Epub 2019 Jun 25.
Molecular characteristics of Î±(+)-thalassemia (3.7â€¯kb deletion) in Southeast Asia: Molecular subtypes, haplotypic heterogeneity, multiple founder effects and laboratory diagnostics	Charoenwijitkul T, Singha K, Fucharoen G, Sanchaisuriya K, Thepphitak P, Wintachai P, Karnpean R, Fucharoen S.	Clin Biochem. 2019 Sep;71:31-37. doi: 10.1016/j.clinbiochem.2019.06.005. Epub 2019 Jun 12.
Molecular analysis of a large novel deletion causing Î±(+)-thalassemia	Zhuang J, Tian J, Wei J, Zheng Y, Zhuang Q, Wang Y, Xie Q, Zeng S, Wang G, Pan Y, Jiang Y.	BMC Med Genet. 2019 May 6;20(1):74. doi: 10.1186/s12881-019-0797-8.
Hb H Disease Results from Compound Heterozygosity of -â€‰-(SEA) and -Î±(MAL3.5) in a Chinese Family	Zhao Y, Lou J, Sun M, Fu Y, Ye W, Li Y, Dai Y, Liu Y.	Hemoglobin. 2019 Jan;43(1):69-72. doi: 10.1080/03630269.2019.1575849. Epub 2019 Feb 27.
Validation of Microarray for the Simultaneous Detection of Common Î±- and Î²-Thalassemia Gene Mutations	Pornprasert S, Anurak R, Ruengdit C, Pienthai N, Tookjai M, Punyamung M, Pongpunyayuen P.	Lab Med. 2019 Jul 16;50(3):306-312. doi: 10.1093/labmed/lmy087.
First Report of a Case with Nondeletional Hb H Disease Caused by IVS-I-116 (A>G) of the Î±2-Globin Gene	He XH, Zhang R, Mai GX, Ren LR, Li DZ.	Hemoglobin. 2018 Sep-Nov;42(5-6):344-346. doi: 10.1080/03630269.2018.1556684. Epub 2019 Jan 24.
Potential new approaches to the management of the Hb Bart's hydrops fetalis syndrome: the most severe form of Î±-thalassemia	King AJ, Higgs DR.	Hematology Am Soc Hematol Educ Program. 2018 Nov 30;2018(1):353-360. doi: 10.1182/asheducation-2018.1.353.
A Rare Hb H Hydrops Fetalis Syndrome Caused by the - - (SEA) Deletion in Combination with the Rare Hb Hirosaki Mutation in a Chinese Patient	Li Q, Li Y, Zhong M, Zhang VW, Jin W, Li S, Li L.	Hemoglobin. 2018 Jul;42(4):278-280. doi: 10.1080/03630269.2018.1536665. Epub 2018 Nov 29.
Increased Prevalence of Thalassemia in Young People in Korea: Impact of Increasing Immigration	Lee HJ, Shin KH, Kim HH, Yang EJ, Park KH, Kim MJ, Kwon JR, Choi YS, Kim JN, Shin MG, Cho YG, Kim	Ann Lab Med. 2019 Mar;39(2):133-140. doi: 10.3343/alm.2019.39.2.133.

	SJ, Kim KH, Jeong S, Kong SG, Jung YJ, Lee N, Kim MJ, Seong MW.	
Analysis of Gene Mutation Types of Î±- and Î²-Thalassemia in Fuzhou, Fujian Province in China	Xu C, Liao B, Qi Y, Huangfu Z, Chen J, Chen Y.	Hemoglobin. 2018 May;42(3):143-147. doi: 10.1080/03630269.2018.1496096. Epub 2018 Oct 22.
A Simple Whole-Blood Polymerase Chain Reaction without DNA Extraction for Thalassemia Diagnosis	Srisuwan W, Tatu T.	Hemoglobin. 2018 May;42(3):178-183. doi: 10.1080/03630269.2018.1496929. Epub 2018 Oct 4.
Development of Visual Detection of Î±-Thalassemia-1 (the - -(SEA) Deletion) Using pH-Sensitive Loop-Mediated Isothermal Amplification	Chomean S, Pholyiam K, Thamwarokun A, Kaset C.	Hemoglobin. 2018 May;42(3):171-177. doi: 10.1080/03630269.2018.1488723. Epub 2018 Sep 7.
The phenomena of balanced effect between Î±-globin gene and of Î²-globin gene	Zhong L, Gan X, Xu L, Liang C, Xie Y, Lin W, Chen P, Liu M.	BMC Med Genet. 2018 Aug 17;19(1):145. doi: 10.1186/s12881-018-0659-9.
Molecular Spectrum of Î±- and Î²-Thalassemia Mutations in a Large Ethnic Hakka Population in Southern China	Zhao P, Weng R, Wu H.	Hemoglobin. 2018 Mar;42(2):117-121. doi: 10.1080/03630269.2018.1470094. Epub 2018 Jul 23.
Whole Blood PCR for Rapid Screening of Î±(0)-Thalassemia	Wichian P, Yamsri S, Sanchaisuriya K, Fucharoen G, Fucharoen S.	Ann Clin Lab Sci. 2018 Mar;48(2):231-235.
Outcomes and morbidities of patients who survive haemoglobin Bart's hydrops fetalis syndrome: 20-year retrospective review	Chan WY, Leung AW, Luk CW, Li RC, Ling AS, Ha SY.	Hong Kong Med J. 2018 Apr;24(2):107-118. doi: 10.12809/hkmj176336. Epub 2018 Apr 6.
Characterization of Hb Bart's Hydrops Fetalis Caused by -â€‰-(SEA) and a Large Novel Î±(0)-Thalassemia Deletion	He S, Li J, Huang P, Zhang S, Lin L, Zuo Y, Tian X, Zheng C, Qiu X, Chen B.	Hemoglobin. 2018 Jan;42(1):61-64. doi: 10.1080/03630269.2018.1434198. Epub 2018 Mar 1.
A prospective analysis for prevalence of complications in Thai nontransfusion-dependent Hb E/Î²-thalassemia and Î±-thalassemia (Hb H disease)	Ekwattanakit S, Siritanaratkul N, Viprakasit V.	Am J Hematol. 2018 May;93(5):623-629. doi: 10.1002/ajh.25046. Epub 2018 Feb 14.
Coinheritance of Î±- and Î²-Thalassemia with a Novel Mutation (HBB: c.268_281delAGTGAGCTGCACTG) in a Chinese Family	Cheng C, Peng Q, Li S, Yang L, Li W, Rao C, Lu X.	Hemoglobin. 2017 Jul-Nov;41(4-6):288-290. doi: 10.1080/03630269.2017.1405018. Epub 2017 Dec 18.
Two Î±1-Globin Gene Point Mutations Causing Severe Hb H Disease	Jiang H, Huang LY, Zhen L, Jiang F, Li DZ.	Hemoglobin. 2017 Jul-Nov;41(4-6):293-296. doi: 10.1080/03630269.2017.1390478. Epub 2017 Nov 8.
First Report of Hb Kent [Î²37(C3)Trpâ†’Cys	Chan NCN, Chow KH, Leung RFY, Tang SSH, Chiu MFW, Lie R, Cheng	Hemoglobin. 2017 Jul-Nov;41(4-6):283-285. doi:

(TGG>TGC) HBB: c.114G>C] in a Chinese Family	KCK, Lau KM, Chan NPH, Ng MHL.	10.1080/03630269.2017.1378673. Epub 2017 Oct 18.
Novel interactions of two Î±-Hb variants with SEA deletion Î±(0)-thalassemia: hematological and molecular analyses	Srivorakun H, Singha K, Fucharoen G, Fucharoen S.	Hematology. 2018 Apr;23(3):187-191. doi: 10.1080/10245332.2017.1380930. Epub 2017 Sep 25.
Î²â€‘thalassemia caused by compound heterozygous mutations and cured by bone marrow transplantation: A case report	Wu L, Peng Z, Lu S, Tan M, Rong Y, Tian R, Yang Y, Chen Y, Chen J.	Mol Med Rep. 2017 Nov;16(5):6552-6557. doi: 10.3892/mmr.2017.7476. Epub 2017 Sep 12.
A comprehensive ethnic-based analysis of alpha thalassaemia allelle frequency in northern Thailand	Kulaphisit M, Kampuansai J, Leecharoenkiat K, Wathikthinnakon M, Kangwanpong D, Munkongdee T, Svasti S, Fucharoen S, Smith DR, Lithanatudom P.	Sci Rep. 2017 Jul 5;7(1):4690. doi: 10.1038/s41598-017-04957-2.
Design, Validation, and Clinical Implementation of a Gap-Polymerase Chain Reaction Method for Î±-Thalassemia Genotyping Using Capillary Electrophoresis	Basha B, Mularo F, Cook JR.	Hemoglobin. 2017 Mar;41(2):124-130. doi: 10.1080/03630269.2017.1327868. Epub 2017 Jun 9.
A Pilot Study of Noninvasive Prenatal Diagnosis of Alpha- and Beta-Thalassemia with Target Capture Sequencing of Cell-Free Fetal DNA in Maternal Blood	Wang W, Yuan Y, Zheng H, Wang Y, Zeng D, Yang Y, Yi X, Xia Y, Zhu C.	Genet Test Mol Biomarkers. 2017 Jul;21(7):433-439. doi: 10.1089/gtmb.2016.0411. Epub 2017 May 24.
Complex Interaction of Hb Q-Thailand with Î±(0)- and Î²(0)-Thalassemia in a Chinese Family	He S, Qin Q, Lin L, Chen Q, Yi S, Wei H, Du J, Zheng C, Qiu X, Chen B.	Hemoglobin. 2017 Jan;41(1):68-72. doi: 10.1080/03630269.2017.1295985. Epub 2017 May 5.
The Hb E (HBB: c.79G>A), Mean Corpuscular Volume, Mean Corpuscular Hemoglobin Cutoff Points in Double Heterozygous Hb E/- -(SEA) Î±-Thalassemia-1 Carriers are Dependent on Hemoglobin Levels	Leckngam P, Limweeraprajak E, Kiewkarnkha T, Tatu T.	Hemoglobin. 2017 Jan;41(1):38-43. doi: 10.1080/03630269.2017.1295984. Epub 2017 Apr 16.
Genetic origin of Î±(0)-thalassemia (SEA deletion) in Southeast Asian populations and application to accurate prenatal diagnosis of Hb Bart's hydrops fetalis syndrome	Jomoui W, Fucharoen G, Sanchaisuriya K, Charoenwijitkul P, Maneesarn J, Xu X, Fucharoen S.	J Hum Genet. 2017 Aug;62(8):747-754. doi: 10.1038/jhg.2017.41. Epub 2017 Apr 6.
