# Supplementary material for: Prevalence of Alpha(α)-Thalassemia in Southeast Asia (2010–2020): A Meta-Analysis Involving 83,674 Subjects
Source: Int J Environ Res Public Health. 2020 Oct 9;17(20):7354. doi: 10.3390/ijerph17207354 (PMC7600098; doi:10.3390/ijerph17207354)
Supplement: Supplementary file 1 [file ijerph-17-07354-s001.zip › ijerph-909078-supplementary/Table S3. Initial literature search from SCOPUS..pdf]

**Table S3**: Initial literature search from SCOPUS.

| Title | Authors | DOI |
|---|---|---|
| Preconception carrier screening and prenatal diagnosis in thalassemia and hemoglobinopathies: challenges and future perspectives | Traeger-Synodinos J., Harteveld C.L. | 10.1080/14737159.2017.1285701 |
| Low oxygen saturation and severe anemia in compound heterozygous Hb Louisville [Î²42(CD1)Pheâ†’Leu] and Hb La Desirade [Î²129(H7)Alaâ†’Val] | Kamseng P., Trakulsrichai S., Trachoo O., Yimniam W., Panthan B., Jittorntam P., Niparuck P., Sanguanwit P., Wananukul W., Jindadamrongwech S. | 10.1080/10245332.2016.1231989 |
| The epidemiologic transition of thalassemia and associated hemoglobinopathies in southern Taiwan | Wang H.-C., Hsieh L.-L., Liu Y.-C., Hsiao H.-H., Lin S.-K., Tsai W.-C., Liu T.-C. | 10.1007/s00277-016-2868-7 |
| Diagnosis of common hemoglobinopathies among South East Asian population using capillary isoelectric focusing system | Srivorakun H., Fucharoen G., Sanchaisuriya K., Fucharoen S. | 10.1111/ijlh.12585 |
| Detection of the common South-East Asian Î²0-thalassemia mutations in samples with borderline HbA2 levels | Rungsee P., Kongthai K., Pornprasert S. | 10.1515/cclm-2016-0470 |
| Practice of iron chelation therapy for transfusion-dependent thalassemia in Southeast Asia | Azman N.F., Abdullah W.-Z., Mohamad N., Bahar R., Johan M.F., Diana R., Sarifah B.H., Yusoff S., Nasir A., Othman A., Sukeri S., Ibrahim M.I., Hussein A., Hassan R., Yahya P., Hassan R., Zilfalil B.A. | 10.5372/1905-7415.1006.524 |
| Molecular Epidemiology of Hemoglobinopathies in Cambodia | Munkongdee T., Tanakulmas J., Butthep P., Winichagoon P., Main B., Yiannakis M., George J., Devenish R., Fucharoen S., Svasti S. | 10.3109/03630269.2016.1158723 |
| Progress Toward the Control and Management of the Thalassemias | Fucharoen S., Weatherall D.J. | 10.1016/j.hoc.2015.12.001 |
| Hematological Practice in Hong Kong and China | Kwong Y.-L., Ha S.-Y., Chan V. | 10.1016/j.hoc.2015.11.010 |
| Î² -Thalassemia Haplotypes in Romania in the Context of Genetic Mixing in the Mediterranean Area | Cherry L., Calo C., Talmaci R., Perrin P., Gavrila L. | 10.3109/03630269.2015.1124113 |
| Prevalence of Î±-thalassaemia genotypes in pregnant women in northern Thailand | Pharephan S., Sirivatanapa P., Makonkawkeyoon S., Tuntiwechapikul W., Makonkawkeyoon L. | 10.4103/0971-5916.182622 |
| Molecular Heterogeneity of Thalassemia among Pregnant Laotian Women | Wongprachum K., Sanchaisuriya K., Dethvongphanh M., Norcharoen B., Htalongsengchan B., Vidamaly V., Sanchaisuriya P., Fucharoen S., Fucharoen G., Schelp F.P. | 10.1159/000438739 |
| When to consider transfusion therapy for patients with non-transfusion-dependent thalassaemia | Taher A.T., Radwan A., Viprakasit V. | 10.1111/vox.12201 |
| Development of hemoglobin typing control materials for laboratory investigation of thalassemia and hemoglobinopathies | Pornprasert S., Tookjai M., Punyamung M., Pongpunyayuen P., Jaiping K. | 10.1515/cclm-2015-0113 |

| | | |
|---|---|---|
| Thalassemia screening using different automated blood cell counters: Consideration of appropriate cutoff values | Chaitraiphop C., Sanchaisuriya K., Inthavong S., Fucharoen G., Sanchaisuriya P., Changtrakun Y., Fucharoen S. | 10.7754/Clin.Lab.2015.150720 |
| Characterization of Deletions of the HBA and HBB Loci by Array Comparative Genomic Hybridization | Sabath D.E., Bender M.A., Sankaran V.G., Vamos E., Kentsis A., Yi H.-S., Greisman H.A. | 10.1016/j.jmoldx.2015.07.011 |
| Prevalence of thalassemia and hemoglobinopathy in eastern India: A 10-year high-performance liquid chromatography study of 119,336 cases | Mondal S.K., Mandal S. | 10.4103/0973-6247.175424 |
| Health-related quality of life and health utility values in beta thalassemia major patients receiving different types of iron chelators in Iran | Seyedifar M., Dorkoosh F.A., Hamidieh A.A., Naderi M., Karami H., Karimi M., Fadaiyrayeny M., Musavi M., Safaei S., Ahmadian-Attari M.M., Hadjibabaie M., Cheraghali A.M., Sari A.A. | |
| Effect of combined versus monotherapy with deferoxamine and deferiprone in Iron overloaded thalassemia patients: A randomized clinical trial | Hejazi S., Safari O., Arjmand R., Qorbani M., Pourrostami K., Safari A., Hemmati A. | 10.22038/ijp.2016.6871 |
| Risk assessment of gene variants for neonatal hyperbilirubinemia in Taiwan | Weng Y.-H., Chiu Y.-W., Cheng S.-W., Yang C.-Y. | 10.1186/s12887-016-0685-8 |
| Two independent genetic origins of Î²+-thalassemia Due to -31 A to G mutation in thai and japanese populations | Lerttham W., Fucharoen G., Yamsri S., Fucharoen S. | |
| Hemoglobin constant spring among southeast asian populations: Haplotypic heterogeneities and phylogenetic analysis | Jomoui W., Fucharoen G., Sanchaisuriya K., Hoa Nguyen V., Fucharoen S. | 10.1371/journal.pone.0145230 |
| Understanding the contrasting spatial haplotype patterns of malaria-protective Î²-globin polymorphisms | Hockham C., Piel F.B., Gupta S., Penman B.S. | 10.1016/j.meegid.2015.09.018 |
| Acute non-atherosclerotic ST-segment elevation myocardial infarction in an adolescent with concurrent hemoglobin H-Constant Spring disease and polycythemia vera | Rattarittamrong E., Norasetthada L., Tantiworawit A., Chai-Adisaksopha C., Hantrakool S., Rattanathammethee T., Charoenkwan P. | 10.4081/hr.2015.5941 |
| Molecular Scanning of Î²-Thalassemia in the Southern Region of Central Java, Indonesia; a Step Towards a Local Prevention Program | Rujito L., Basalamah M., Mulatsih S., Sofro A.S.M. | 10.3109/03630269.2015.1065420 |
| The Effect of Nonsense Mediated Decay on Transcriptional Activity Within the Novel Î²-Thalassemia Mutation HBB: c.129delT | Forster L., Ardakani R.M., Qadah T., Finlayson J., Ghassemifar R. | 10.3109/03630269.2015.1065270 |
| Prevalence of thalassemia carriers among the Lahu hill tribe population, Chiang Rai, Thailand | Apidechkul T. | 10.5372/1905-7415.0904.423 |
| A novel AÎ³Î´Î²0-thalassemia caused by DNA deletion-inversion-insertion of the Î²-globin gene cluster and five olfactory receptor genes: Genetic interactions, | Singha K., Fucharoen G., Hama A., Fucharoen S. | 10.1016/j.clinbiochem.2015.03.023 |

| | | |
|---|---|---|
| hematological phenotypes and molecular characterization | | |
| Molecular spectrum of Î±-globin gene mutations in the Aegean region of Turkey: first observation of three Î±-globin gene mutations in the Turkish population | Onay H., Aykut A., Karaca E., Durmaz A., Solmaz A.E., Ã‡oÄŸulu Ã–., AydÄ±nok Y., Vergin C., Ã–zkÄ±nay F. | 10.1007/s12185-015-1796-y |
| Genetic heterogeneity of the Î²-globin gene in various geographic populations of Yunnan in southwestern China | Zhang J., He J., Zeng X.-H., Ge S.-J., Huang Y., Su J., Ding X.-M., Yang J.-Q., Cao Y.-J., Chen H., Zhang Y.-H., Zhu B.-S. | 10.1371/journal.pone.0122956 |
| Genetic determinants of Î²-thalassemia intermedia in Pakistan | Khan J., Ahmad N., Siraj S., Hoti N. | 10.3109/03630269.2014.1002136 |
| Discrimination of various thalassemia syndromes and iron deficiency and utilization of reticulocyte measurements in monitoring response to iron therapy | Winichagoon P., Kumbunlue R., Sirankapracha P., Boonmongkol P., Fucharoen S. | 10.1016/j.bcmd.2015.01.010 |
| Sequence and analysis of a whole genome from Kuwaiti population subgroup of Persian ancestry | Thareja G., John S.E., Hebbar P., Behbehani K., Thanaraj T.A., Alsmadi O. | 10.1186/s12864-015-1233-x |
| Retrospective analysis of 14 cases of disseminated infection with osteolytic lesions | Qiu Y., Zhang J., Liu G., Zhong X., Deng J., He Z., Jing B. | 10.1186/s12879-015-0782-6 |
| The prevalence and spectrum of Î±-Thalassemia in Guizhou Province of South China | Huang S.-W., Xu Y., Liu X.-M., Zhou M., Li G.-F., An B.-Q., Su L., Wu X., Lin J. | 10.3109/03630269.2015.1041037 |
| The spectrum of Î±-thalassemia mutations in Kermanshah Province, West Iran | Alibakhshi R., Mehrabi M., Omidniakan L., Shafieenia S. | 10.3109/03630269.2015.1070732 |
| A large cohort of Î²+-thalassemia in thailand: Molecular, hematological and diagnostic considerations | Yamsri S., Singha K., Prajantasen T., Taweenan W., Fucharoen G., Sanchaisuriya K., Fucharoen S. | 10.1016/j.bcmd.2014.11.008 |
| Detection of Hb Constant Spring (HBA2: C.427T>C) Heterozygotes in Combination with Î²-Thalassemia or Hb e Trait by Capillary Electrophoresis | Pornprasert S., Saoboontan S., Punyamung M. | 10.3109/03630269.2015.1027827 |
| Clinical and Molecular Characteristics of Non-Transfusion-Dependent Thalassemia in Kuwait | Adekile A.D., Azab A.F., Al-Sharida S.I., Al-Nafisi B.A., Akbulut N., Marouf R.A., Mustafa N.Y. | 10.3109/03630269.2015.1053489 |
| Effect of iron chelator desferrioxamine on serum zinc levels in patients with beta thalassemia major | Sultan S., Irfan S.M., Kakar J., Zeeshan R. | |
| Known and new hemoglobin A2 variants in Thailand and implication for Î²-thalassemia screening | Panyasai S., Fucharoen G., Fucharoen S. | 10.1016/j.cca.2014.09.003 |
| Prevalence of Alpha thalassemia in microcytic anemia: A tertiary care experience from north India | Sharma M., Pandey S., Ranjan R., Seth T., Saxena R. | 10.4084/MJHID.2015.004 |
| Population screening and prevention strategies for thalassemias and other | Chatterjee T., Chakravarty A., Chakravarty S. | 10.3109/03630269.2015.1068799 |

| | | |
|---|---|---|
| hemoglobinopathies of Eastern India: Experience of 18,166 cases | | |
| Interaction of Hb Grey Lynn (Vientiane) [Î±91(FG3)Leu>Phe (Î±1)] with Hb E [Î²26(B8) Glu>Lys] and Î±+-thalassemia: Molecular and hematological analysis | Singha K., Fucharoen G., Fucharoen S. | 10.7754/Clin.Lab.2014.141112 |
| Mutation spectrum of Î²-Thalassemia and other hemoglobinopathies in Chittagong, Southeast Bangladesh | Chatterjee T., Chakravarty A., Chakravarty S., Chowdhury M.A., Sultana R. | 10.3109/03630269.2015.1078810 |
| Craniofacial manifestations of Î²-thalassemia major | Javid B., Said-Al-Naief N. | 10.1016/j.oooo.2014.08.020 |
| Validation of the immunochromatographic strip for Î±-thalassemia screening: A multicenter study | Winichagoon P., Kumpan P., Holmes P., Finlayson J., Newbound C., Kabral A., Li B., Nuinoon M., Fawcett T., Tayapiwatana C., Kasinrerk W., Fucharoen S. | 10.1016/j.trsl.2014.10.013 |
| Community participation for thalassemia prevention initiated by village health volunteers in Northeastern Thailand | Jopang Y., Petchmark S., Jetsrisuparb A., Sanchaisuriya K., Sanchaisuriya P., Schelp F.P. | 10.1177/1010539511430520 |
| Î²-Globin genes: Mutation hot-spots in the global thalassemia belt | Kumar R., Sagar C., Sharma D., Kishor P. | 10.3109/03630269.2014.985831 |
| Coexistence of malaria and thalassemia in malaria endemic areas of Thailand | Kuesap J., Chaijaroenkul W., Rungsihirunrat K., Pongjantharasatien K., Na-Bangchang K. | 10.3347/kjp.2015.53.3.265 |
| Treatment of Î² -thalassemia/hemoglobin e with antioxidant cocktails results in decreased oxidative stress, increased hemoglobin concentration, and improvement of the hypercoagulable state | Yanpanitch O.-U., Hatairaktham S., Charoensakdi R., Panichkul N., Fucharoen S., Srichairatanakool S., Siritanaratkul N., Kalpravidh R.W. | 10.1155/2015/537954 |
| The prevalence of hemoglobinopathies in young adolescents in the province of MuÊ‡la in Turkey: Results of a screening program | Topal Y., Topal H., Ceyhan M.N., Azik F., Ã‡apanoÊ‡lu M., KocabaÅŸ C.N. | 10.3109/03630269.2015.1046185 |
| Mutation screening for thalassaemia in the Jino ethnic minority population of Yunnan Province, Southwest China | Wang S., Zhang R., Xiang G., Li Y., Hou X., Jiang F., Jiang F., Hu C., Jia W. | 10.1136/bmjopen-2015-010047 |
| Increasing prevalence of thalassemia in America: Implications for primary care | Sayani F.A., Kwiatkowski J.L. | 10.3109/07853890.2015.1091942 |
| The effect of thalassemia on erythrocyte reference intervals in a representative han Chinese adult population | Xu J.-H., Hao X.-K., Mu R.-Q., Pan B.-S., Zhang J., Peng M.-T., Wang L.-L., Huang X.-Z., Ma Y.-Y., Zhao M., Guo W., Qiao R., Chen W.-X., Jiang H., Shang H. | 10.7754/Clin.Lab.2014.140905 |
| Detection of deletion Î±+-thalassemia mutation [-Î± (3.7), -Î± (4.2)] by quantitative PCR assay | Seeratanachot T., Shimbhu D., Charoenkwan P., Sanguansermsri T. | |
| Sonographic markers of fetal Î±-thalassemia major | Li X., Zhou Q., Zhang M., Tian X., Zhao Y. | 10.7863/ultra.34.2.197 |

| | | |
|---|---|---|
| Diagnostic pitfalls of less well recognized HbH disease | Farashi S., Najmabadi H. | 10.1016/j.bcmd.2015.08.003 |
| Medium-based noninvasive preimplantation genetic diagnosis for human Î±-thalassemias-SEA | Wu H., Ding C., Shen X., Wang J., Li R., Cai B., Xu Y., Zhong Y., Zhou C. | 10.1097/MD.0000000000000669 |
| Identification of Hb Constant Spring (HBA2: C.427T > C) by an Automated High Performance Liquid Chromatography Method | Wisedpanichkij R., Jindadamrongwech S., Butthep P. | 10.3109/03630269.2015.1027828 |
| The homozygous hemoglobin EE genotype and chronic inflammation are associated with high serum ferritin and soluble transferrin receptor concentrations among women in rural Cambodia | Karakochuk C.D., Whitfield K.C., Rappaport A.I., Barr S.I., Vercauteren S.M., McLean J., Prak S., Hou K., Talukder A., Devenish R., Green T.J. | 10.3945/jn.115.218636 |
| Genetic hemoglobin disorders rather than iron deficiency are a major predictor of hemoglobin concentration in women of reproductive age in rural prey veng, Cambodia | Karakochuk C.D., Whitfield K.C., Barr S.I., Lamers Y., Devlin A.M., Vercauteren S.M., Kroeun H., Talukder A., McLean J., Green T.J. | 10.3945/jn.114.198945 |
| Melioidosis in the non-endemic setting: Not only in diabetic travelers returning from Southeast Asia | Bottieau E., Vlieghe E. | 10.1016/j.tmaid.2015.09.002 |
| Epidemiology of hepatitis C virus in Iran | Taherkhani R., Farshadpour F. | 10.3748/wjg.v21.i38.10790 |
| Hemoglobin e and glucose-6-phosphate dehydrogenase deficiency and plasmodium falciparum malaria in the chittagong hill districts of Bangladesh | Shannon K.L., Ahmed S., Rahman H., Prue C.S., Khyang J., Ram M., Haq M.Z., Chowdhury A., Akter J., Glass G.E., Shields T., Nyunt M.M., Khan W.A., Sack D.A., Sullivan D.J. | 10.4269/ajtmh.14-0623 |
| Risk factors for malaria and adverse birth outcomes in a prospective cohort of pregnant women resident in a high malaria transmission area of Papua New Guinea | Stanisic D.I., Moore K.A., Baiwog F., Ura A., Clapham C., King C.L., Siba P.M., Beeson J.G., Mueller I., Fowkes F.J., Rogerson S.J. | 10.1093/trstmh/trv019 |
| Dengue virus infection of erythroid precursor cells is modulated by both thalassemia trait status and virus adaptation | Sornjai W., Khungwanmaythawee K., Svasti S., Fucharoen S., Wintachai P., Yoksan S., Ubol S., Wikan N., Smith D.R. | 10.1016/j.virol.2014.10.004 |
| Genotyping of Î±-thalassemias by the colorimetric nanogold probes | Chomean S., Wangmaung N., Sritongkham P., Promptmas C., Ittarat W. | 10.1016/j.cca.2014.07.033 |
| A large cohort of hemoglobin variants in thailand: Molecular epidemiological study and diagnostic consideration | Srivorakun H., Singha K., Fucharoen G., Sanchaisuriya K., Fucharoen S. | 10.1371/journal.pone.0108365 |
| Molecular epidemiological characterization and health burden of thalassemia in Jiangxi Province, P. R. China | Lin M., Zhong T.-Y., Chen Y.-G., Wang J.-Z., Wu J.-R., Lin F., Tong X., Yang H.-T., Hu X.-M., Hu R., Zhan X.-F., Yang H., Luo Z.-Y., Li W.-Y., Yang L.-Y. | 10.1371/journal.pone.0101505 |
| Running exercise alleviates trabecular bone loss and osteopenia in hemizygous Î²-globin knockout thalassemic mice | Thongchote K., Svasti S., Teerapornpuntakit J., Krishnamra N., Charoenphandhu N. | 10.1152/ajpendo.00111.2014 |

| | | |
|---|---|---|
| Molecular characterization of Î±- and Î²-thalassaemia among Malay patients | Mohd Yatim N.F., Abd. Rahim M., Menon K., Al-Hassan F.M., Ahmad R., Manocha A.B., Saleem M., Yahaya B.H. | 10.3390/ijms15058835 |
| A simple and highly sensitive elisa for screening of the Î±-thalassemia-1 southeast asian-type deletion | Pata S., Khummuang S., Pornprasert S., Tatu T., Kasinrerk W. | 10.1080/15321819.2013.838963 |
| The prevalence and molecular spectrum of Î±- and Î²-globin gene mutations in 14,332 families of Guangdong Province, China | Yin A., Li B., Luo M., Xu L., Wu L., Zhang L., Ma Y., Chen T., Gao S., Liang J., Guo H., Qin D., Wang J., Yuan T., Wang Y., Huang W.-W., He W.-F., Zhang Y., Liu C., Xia S., Chen Q., Zhao Q., Zhang X. | 10.1371/journal.pone.0089855 |
| Interaction of hemoglobin Grey Lynn (Vientiane) with a non-deletional Î±+-thalassemia in an adult Thai proband | Singha K., Fucharoen G., Fucharoen S. | 10.11613/bm.2014.019 |
| Genetic compound heterozygosity for Southeast Asian ovalocytosis and thalassemia in Thailand: Prevalence and phenotypic analysis | Ngouprommin L., Sae-ung N., Fucharoen S., Fucharoen G., Sanchaisuriya K., Jetsrisuparb A. | 10.1111/cge.12128 |
| HLA-A, HLA-B, HLA-DRB1 allele and haplotype frequencies in 6384 umbilical cord blood units and transplantation matching and engraftment statistics in the Zhejiang cord blood bank of China | Wang F., He J., Chen S., Qin F., Dai B., Zhang W., Zhu F.M., Lv H.J. | 10.1111/iji.12064 |
| Measurement of HbA2 by capillary electrophoresis for diagnosing Î²-thalassemia/HbE disease in patients with low hbf | Prasing W., Pornprasert S. | 10.1309/LMGD96HES3DZRBZM |
| The correlation of Î±-globin gene mutations and the XmnI polymorphism with clinical severity of Hb E/Î²-thalassemia | Charoenkwan P., Teerachaimahit P., Sanguansermsri T. | 10.3109/03630269.2014.952744 |
| A newly modified hemoglobin h inclusion test as a secondary screening for Î±0-thalassemia in southeast asian populations | Fucharoen G., Yooyen K., Chaibunruang A., Fucharoen S. | 10.1159/000355187 |
| Phenotypic expression of Hb F in common high Hb F determinants in Thailand: Roles of Î±-thalassemia, 5' Î´-globin BCL11A binding region and 3' Î²-globin enhancer | Prakobkaew N., Fucharoen S., Fuchareon G., Siriratmanawong N. | 10.1111/ejh.12201 |
| Development of bead-based suspension array technology for the diagnosis of thalassemia | Yin A., Zhang L., Luo M., He T., Zhang Y., Liu C., Du L., Qin D., Liang J., Li B., Wu L., Ma Y., Guo H., Wang J., Yuan T., Wang Y., Zhang Y., Zhang X. | 10.1002/ajh.23830 |
| Thalassemia and hemoglobin e in southern Thai blood donors | Nuinoon M., Kruachan K., Sengking W., Horpet D., Sungyuan U. | 10.1155/2014/932306 |
| ARKRAY ADAMS A1c HA-8180T analyzer for diagnosis of thalassemia and hemoglobinopathies common in southeast Asia | Kunwandee J., Srivorakun H., Fucharoen G., Sanchaisuriya K., Fucharoen S. | 10.1309/LMmh649poetqrexl |
| Diagnostic utility of isoelectric focusing and high performance liquid chromatography in neonatal cord blood screening for thalassemia and non-sickling hemoglobinopathies | Uaprasert N., Settapiboon R., Amornsiriwat S., Sarnthammakul P., Thanapat T., Rojnuckarin P., Sutcharitchan P. | 10.1016/j.cca.2013.09.041 |

| | | |
|---|---|---|
| Oral manifestations and blood profile in thalassemia trait | Joob B., Wiwanitkit V. | 10.1016/j.jfma.2013.11.009 |
| Molecular basis of transfusion dependent beta-thalassemia major patients in Sabah | Teh L.K., George E., Lai M.I., Tan J.A.M.A., Wong L., Ismail P. | 10.1038/jhg.2013.131 |
| Blood transfusion from a Hb e trait donor can affect Î²-thalassemia diagnosis | Pornprasert S., Jaiping K. | 10.3109/03630269.2014.926913 |
| Genetic heterogeneity of hemoglobin AEBart's disease: A large cohort data from a single referral center in northeast Thailand | Chaibunruang A., Karnpean R., Fucharoen G., Fucharoen S. | 10.1016/j.bcmd.2013.11.006 |
| Multiplex amplification refractory mutation system (MARMA) for the detection of Î²-globin gene mutations among the transfusion-dependent Î²-thalassemia Malay patients in Kelantan, Northeast of Peninsular Malaysia | Hanafi S., Hassan R., Bahar R., Abdullah W.Z., Johan M.F., Rashid N.D., Azman N.F., Nasir A., Hassan S., Ahmad R., Othman A., Ibrahim M.I., Sukeri S., Sulong S., Yusoff S., Mohamad N.S., Hussein A., Hassan R., Yusoff N., Yahaya B.H., Ismail E., Yussof N.K.N., Salleh S., Zilfalil B.A. | |
| Hemoglobin Constant Spring is markedly high in women of an ethnic minority group in Vietnam: A community-based survey and hematologic features | Nguyen V.H., Sanchaisuriya K., Wongprachum K., Nguyen M.D., Phan T.T.H., Vo V.T., Sanchaisuriya P., Fucharoen S., Schelp F.P. | 10.1016/j.bcmd.2013.12.002 |
| Beta thalassemia in india: Current status and the challenges ahead | Grow K., Vashist M., Abrol P., Sharma S., Yadav R. | |
| New mathematical formula for differentiating thalassemia trait and iron deficiency anemia in thalassemia prevalent area: A study in healthy school-age children | Sirachainan N., Iamsirirak P., Charoenkwan P., Kadegasem P., Wongwerawattanakoon P., Sasanakul W., Chansatitporn N., Chuansumrit A. | |
| Routine screening for a-thalassaemia using an immunochromatographic strip assay for haemoglobin bartâ€™s | Prayalaw P., Fucharoen G., Fucharoen S. | 10.1177/0969141314538611 |
| Clinical Features and Molecular Analysis of Hb H Disease in Taiwan | Chao Y.-H., Wu K.-H., Wu H.-P., Liu S.-C., Peng C.-T., Lee M.-S. | 10.1155/2014/271070 |
| State of the art and new developments in molecular diagnostics for hemoglobinopathies in multiethnic societies | Harteveld C.L. | 10.1111/ijlh.12108 |
| Variability of hemoglobin F expression in hemoglobin EE disease: Hematological and molecular analysis | Pakdee N., Yamsri S., Fucharoen G., Sanchaisuriya K., Pissard S., Fucharoen S. | 10.1016/j.bcmd.2014.02.005 |
| Alpha thalassaemia in tribal communities of coastal maharashtra, India | Deo M.G., Pawar P.V. | |
| Pattern of hemoglobinopathies and thalassemias in upper Assam region of North Eastern India: High performance liquid chromatography studies in 9000 patients | Baruah M.K., Saikia M., Baruah A. | 10.4103/0377-4929.134680 |
| Five hemoglobin variants in a double heterozygote for Î±- and Î²-globin chain defects | Singha K., Fucharoen G., Fucharoen S. | 10.1159/000353123 |

| | | |
|---|---|---|
| Prevention and control of Hb Bart's Disease in Guangxi Zhuang Autonomous Region, China | He S., Zhang Q., Li D., Chen S., Tang Y., Chen Q., Zheng C. | 10.1016/j.ejogrb.2014.03.034 |
| Hb Cibeles [Î±2 CD25(B6) (GlyÂ â†’Â Asp)]: a novel alpha chain variant causing alpha-thalassemia | de la Fuente-Gonzalo F., Nieto J.M., Vinuesa L., Sevilla J., DÃaz-Mediavilla J., Villegas A., GonzÃ¡lez F.A., Ropero P. | 10.1007/s12185-014-1663-2 |
| The Î±-Thalassemias | Piel F.B., Weatherall D.J. | 10.1056/NEJMra1404415 |
| Incidence of ototoxicity in pediatric patients with transfusion-dependent thalassemia who are less well-chelated by mono- and combined therapy of iron chelating agents | Tanphaichitr A., Kusuwan T., Limviriyakul S., Atipas S., Pooliam J., Sangpraypan T., Tanphaichitr V.S., Viprakasit V. | 10.3109/03630269.2014.940462 |
| Novel approach to reactive oxygen species in nontransfusion-dependent thalassemia | Tyan P.I., Radwan A.H., Eid A., Haddad A.G., Wehbe D., Taher A.T. | 10.1155/2014/350432 |
| Advances in technologies for screening and diagnosis of hemoglobinopathies | Traeger-Synodinos J., Harteveld C.L. | 10.2217/bmm.13.103 |
| Molecular characteristic of alpha thalassaemia among patients diagnosed in UKM medical centre | Azma R.Z., Ainoon O., Hafiza A., Azlin I., Noor Farisah A.R., Nor Hidayati S., Noor Hamidah H. | |
| Incidence and prevalence of chronic iron poisoning and it's management: A review | Santra S., Agrawal D., Kumar S., Mishra S.S. | |
| Effect of Swiss-type heterocellular HPFH from XmnI-GÎ³ and HBBP1 polymorphisms on HbF, HbE, MCV and MCH levels in Thai HbE carriers | Kerdpoo S., Limweeraprajak E., Tatu T. | 10.1007/s12185-014-1516-z |
| Transfusion transmitted infections in thalassaemics: Need for reappraisal of blood screening strategy in india | Shyamala V. | 10.1111/tme.12110 |
| Haemoglobinopathies in nonendemic areas in recent years | Frezzotti A., Galeazzi M., Paladini C., Tocchini M. | 10.1515/cclm-2013-0712 |
| An evolutionary perspective of how infection drives human genome diversity: The case of malaria | Mangano V.D., Modiano D. | 10.1016/j.coi.2014.06.004 |
| Immunogenicity of a live-attenuated Japanese encephalitis vaccine in children and adolescents after hematopoietic stem cell transplantation | Pakakasama S., Wattanatitan S., Techasaensiri C., Yoksan S., Sirireung S., Hongeng S. | 10.1038/bmt.2014.149 |
| Spectrum of Î²-thalassemia mutations in Guizhou Province, PR China, including first observation of codon 121 (GAA>TAA) in Chinese population | Huang S.-W., Liu X.-M., Li G.-F., Su L., Wu X., Wang R.-L. | 10.1016/j.clinbiochem.2013.09.014 |
| Phenotypic comparison of four thalassemia model mice reconstructed from cryo-banked embryos | Boonkusol D., DinnyÃ©s A., Sa-Ardrit M., Svasti S., Faisaikarm T., Vadolas J., Fucharoen S., Kitiyanant Y. | 10.1556/ABiol.64.2013.4.5 |
| Clinical and haematological presentations of haemoglobin e disorders from Northern Pakistan | Zarin Khattak A., Sohail Taj A., Ali Shah S.M., Farooq Khattak M. | |

| | | |
|---|---|---|
| Isothermal strand-displacement polymerase reaction for visual detection of the southeast Asian-type deletion of Î±-thalassemia | Yu L., Wu W., Lie P., Liu Y., Zeng L. | 10.1016/j.jmoldx.2013.06.003 |
| Rapid prenatal diagnosis of common beta-thalassemia mutations in Southeast Asia using pyrosequencing | Ho S.S.Y., Huan P.T., Leow G.H., Ching L.K., Chiu L., Law H.Y., Koay E.S.C. | 10.1002/pd.4183 |
| Diagnosis and prevention of thalassemia | Ip H.-W., So C.-C. | 10.3109/10408363.2013.847236 |
| Burden of anemia in relation to thalassemia and iron deficiency among vietnamese pregnant women | Siridamrongvattana S., Van Hoa N., Sanchaisuriya K., Dung N., Hoa P.T.T., Sanchaisuriya P., Fucharoen G., Fucharoen S., Schelp F.P. | 10.1159/000351168 |
| Hematological characterization of compound heterozygous hemoglobin Hope/E patients with and without Î±-Thalassemia-1 SEA type deletion | Pornprasert S. | 10.7754/Clin.Lab.2013.130139 |
| Distribution of thalassemias and associated hemoglobinopathies identified by prenatal diagnosis in Taiwan | Peng C.-T., Liu S.-C., Peng Y.-C., Lin T.-H., Wang S.-J., Le C.-Y., Shih M.-C., Tien N., Lu J.-J., Lin C.-Y. | 10.1016/j.bcmd.2013.04.007 |
| Fetal red blood cell parameters in thalassemia and hemoglobinopathies | Karnpean R., Fucharoen G., Fucharoen S., Ratanasiri T. | 10.1159/000354343 |
| Strategies for basic laboratory diagnostics of the hemoglobinopathies in multi-ethnic societies: Interpretation of results and pitfalls | Giordano P.C. | 10.1111/ijlh.12037 |
| Distribution of alpha thalassaemia gene variants in diverse ethnic populations in Malaysia: Data from the institute for medical Research | Ahmad R., Saleem M., Aloysious N.S., Yelumalai P., Mohamed N., Hassan S. | 10.3390/ijms140918599 |
| The 12.6 kb-deletion in the Î²-globin gene cluster is the known Thai/Vietnamese (Î´Î²)0-thalassemia commonly found in Southeast Asia | Chalaow N., Thein S.L., Viprakasit V. | 10.3324/haematol.2013.090613 |
| Low fetal hemoglobin rates in patients carrying Thai hypothesis that the 5'd BCL11A binding site plays a major role in its fetal hemoglobin inhibitory regulation. Response to "The 12.6 kb-deletion in the Î²-globin gene cluster is the known in Southeast Asia" | Ghedira E.S., Pissard S. | 10.3324/haematol.2013.093716 |
| Discussion | Agarwal A.M., Nussenzveig R.H., Hoke C., Lorey T.S., Greene D.N. | 10.1309/AJCPF4UIJKH3EOBY |
| Analysis of distribution of thalassemic genotype and clinical phenotype | LU L.-H., YU J., ZHANG Y.-H., WU X.-F., LIU X.-Y. | 10.7669/j.issn.1001-7844.2013.03.0143 |
| Prenatal control of nondeletional Î±-thalassemia: First experience in mainland china | Li J., Li R., Zhou J.-Y., Xie X.-M., Liao C., Li D.-Z. | 10.1002/pd.4149 |

| | | |
|---|---|---|
| Molecular and hematological studies in a large cohort of Î±0-thalassemia in northeast Thailand: Data from a single referral center | Chaibunruang A., Prommetta S., Yamsri S., Fucharoen G., Sae-Ung N., Sanchaisuriya K., Fucharoen S. | 10.1016/j.bcmd.2013.04.003 |
| Problems in determining thalassemia carrier status in a program for prevention and control of severe thalassemia syndromes: A lesson from Thailand | Viprakasit V., Limwongse C., Sukpanichnant S., Ruangvutilert P., Kanjanakorn C., Glomglao W., Sirikong M., Utto W., Tanphaichitr V.S. | 10.1515/cclm-2013-0098 |
| Cost-utility analysis of oral deferasirox versus infusional deferoxamine in transfusion-dependent Î²-thalassemia patients | Keshtkaran A., Javanbakht M., Salavati S., Mashayekhi A., Karimi M., Nuri B. | 10.1111/trf.12024 |
| A two-layered classifier based on the radial basis function for the screening of thalassaemia | Masala G.L., Golosio B., Cutzu R., Pola R. | 10.1016/j.compbiomed.2013.08.020 |
| Thalassemia and Hemoglobinopathies in Thua Thien Hue Province, Central Vietnam | Nguyen H.V., Sanchaisuriya K., Nguyen D., Phan H.T.T., Siridamrongvattana S., Sanchaisuriya P., Fucharoen S., Fucharoen G., Schelp F.P. | 10.3109/03630269.2013.790829 |
| Evaluation and comparison of soluble transferrin receptor in thalassemia carriers and iron deficient patients | Khatami S., Dehnabeh S.R., Mostafavi E., Kamalzadeh N., Yaghmaei P., Saeedi P., Shariat F., Bagheriyan H., Zeinali S., Akbari M.T. | 10.3109/03630269.2013.780248 |
| Analysis of Î²/Î± globin ratio by using relative qRT-PCR for diagnosis of beta-thalassemia carriers | Ranjbaran R., Okhovat M.A., Mobarhanfard A., Aboualizadeh F., Abbasi M., Moezzi L., Golafshan H.A., Behzad-Behbahani A., Bagheri M., Sharifzadeh S. | 10.1002/jcla.21594 |
| No evidence for role of common anion exchanger 1 mutations on the severity difference in HB E-Î²-Thalassemia disease in Northeast Thailand | Ngouprommin L., Sae-Ung N., Fucharoen S., Fucharoen G., Sanchaisuriya K., Jetsrisuparb A. | |
| Diagnostic testing for Î±-globin gene disorders in a heterogeneous North American population | Waye J.S., Eng B. | 10.1111/ijlh.12066 |
| A proficiency testing program of hemoglobin analysis in prevention and control of severe hemoglobinopathies in Thailand | Karnpean R., Fucharoen G., Pansuwan A., Changtrakul D., Fucharoen S. | 10.1515/cclm-2012-0588 |
| Low cost biosensor-based molecular differential diagnosis of Î±-thalassemia (Southeast Asia deletion) | Wangmaung N., Promptmas C., Chomean S., Sanchomphu C., Ittarat W. | 10.1515/cclm-2012-0732 |
| Prenatal and newborn screening for hemoglobinopathies | Hoppe C.C. | 10.1111/ijlh.12076 |
| Effectiveness of the model for prenatal control of severe thalassemia | Tongsong T., Charoenkwan P., Sirivatanapa P., Wanapirak C., Piyamongkol W., Sirichotiyakul S., Srisupundit K., Tongprasert F., Luewan S., Ratanasiri T., Komwilaisak R., Saksiriwuttho P., Vuthiwong C., Punpuckdeekoon P., Panichkul P., Rueangchainikhom W., Choowong J., Orungrote N., Sarapak S., Kovavisarach E., Jaruyawongs P., Tansathit T., Phadungkiatwattana P., Rujiwetpongstorn J., Kor-Anantakul O., Suwanrath C., Hanprasertpong T., Pranpanus S. | 10.1002/pd.4095 |

| | | |
|---|---|---|
| Treating iron overload in patients with non-transfusion-dependent thalassemia | Taher A.T., Viprakasit V., Musallam K.M., Cappellini M.D. | 10.1002/ajh.23405 |
| Prenatal and post-natal screening of Î²-thalassemia and hemoglobin e genes in Thailand using denaturing high performance liquid chromatography | Prajantasen T., Fucharoen S., Fucharoen G., Siriratmanawong N., Pinmuang-Ngam C. | 10.1007/s11033-012-2391-4 |
| Factors affecting nutritional status among pediatric patients with transfusion-dependent beta thalassemia | Mirhosseini N.Z., Shahar S., Ghayour-Mobarhan M., Kamaruddin N.A., Banihashem A., Yusoff N.A.M., Esmaili H.A., Tavallaei S. | 10.1007/s12349-012-0112-0 |
| Detection of coinherited Hb H-constant spring/PaksÃ© disease and Hb e by capillary electrophoresis and high performance liquid chromatography | Pornprasert S., Waneesorn J. | 10.3109/03630269.2012.752744 |
| Evidence of recent natural selection on the Southeast Asian deletion ( - SEA) causing Î±-thalassemia in South China | Qiu Q.-W., Wu D.-D., Yu L.-H., Yan T.-Z., Zhang W., Li Z.-T., Liu Y.-H., Zhang Y.-P., Xu X.-M. | 10.1186/1471-2148-13-63 |
| Diagnosis of Î±-thalassemia-1 southeast Asian type deletion and fetal gender by single-tube multiplex real-time PCR | Srithep S., Kongthai K., Pornprasert S. | 10.7754/Clin.Lab.2012.120217 |
| Alpha-thalassemia mental retardation syndrome: A case report of two affected siblings | Latiff Z.A., Omar S.A.S., Lau D., Wong S.W., Ong L.C., Aziz D.A., Akmal S.N. | 10.3233/JPN-120598 |
| The prevention of thalassemia | Cao A., Kan Y.W. | 10.1101/cshperspect.a011775 |
| Protection against Oxidative Stress in Beta Thalassemia/Hemoglobin E Erythrocytes by Inhibitors of Glutathione Efflux Transporters | Muanprasat C., Wongborisuth C., Pathomthongtaweechai N., Satitsri S., Hongeng S. | 10.1371/journal.pone.0055685 |
| Association of Hb thailand [Î±56(E5)Lysâ†'Thr] and Hb phnom penh [Î±117(GH5)-Ile-Î±118(H1)] with Î±;0-Thalassemia: Molecular and hematological features and differential diagnosis | Singha K., Srivorakun H., Fucharoen G., Changtrakul Y., Komwilaisak P., Jetsrisuparb A., Puangplruk R., Fucharoen S. | 10.3109/03630269.2012.747964 |
| Identification of hemoglobin variants in samples received for glycated hemoglobin testing | Saw S., Loh T.P., Yin C., Sethi S.K. | 10.1016/j.cca.2012.10.043 |
| Phenotypic heterogeneity of asian indian inversion deletions GÎ³(AÎ³Î´Î²)0 breakpoint A and breakpoint B | Pandey S., Pandey S., Ranjan R., Mishra R., Sharma M., Saxena R. | 10.1007/s12291-012-0232-9 |
| Genotyping of beta thalassemia trait by high-resolution DNA melting analysis | Saetung R., Ongchai S., Charoenkwan P., Sanguansermsri T. | |
| To study the haemoglobinopathies and ratio of copper and zinc in Sindhi Community of Bhopal | Kaur M., Dangi C.B.S., Singh H. | |
| Detection of Î²-globin gene mutations among Î²-thalassaemia carriers and patients in malaysia: Application of multiplex amplification refractory mutation system-polymerase chain reaction | Hassan S., Ahmad R., Zakaria Z., Zulkafli Z., Abdullah W.Z. | |

| | | |
|---|---|---|
| Occult hepatitis B virus (HBV) infection: A global challenge for medicine | Assar S., Arababadi M.K., Ahmadabadi B.N., Salehi M., Kennedy D. | 10.7754/Clin.Lab.2012.120119 |
| A single-tube multiplex gap-polymerase chain reaction for the detection of eight Î²-globin gene cluster deletions common in Southeast Asia | Tritipsombut J., Phylipsen M., Viprakasit V., Chalaow N., Sanchaisuriya K., Giordano P.C., Fucharoen S., Harteveld C.L. | 10.3109/03630269.2012.747441 |
| Distribution of alpha thalassaemia in 16 year old Malaysian students in Penang, Melaka and Sabah | Ahmad R., Sabrina N., Bahrin S., Hassan R., Yelumalai P., Hidayat N., Hassan S., Zakaria Z. | |
| Prevalence of anemia and underlying iron status in naive antiretroviral therapy HIV-infected children with moderate immune suppression | Kosalaraksa P., Bunupuradah T., Vonthanak S., Wiangnon S., Hansudewechakul R., Vibol U., Kanjanavanit S., Ngampiyaskul C., Wongsawat J., Luesomboon W., Lumbiganon P., Sopa B., Apornpong T., Chuenyam T., Cooper D.A., Ruxrungtham K., Ananworanich J., Puthanakit T. | 10.1089/aid.2011.0373 |
| Plasma membrane CA2+-atpase sulfhydryl modifications: Implication for oxidized red cell | Pengpanichpakdee N., Thadtapong T., Auparakkitanon S., Wilairat P. | |
| Platelet activation and platelet-leukocyte interaction in Î²-thalassemia/hemoglobin e patients with marked nucleated erythrocytosis | Keawvichit R., Khowawisetsut L., Chaichompoo P., Polsrila K., Sukklad S., Sukapirom K., Khuhapinant A., Fucharoen S., Pattanapanyasat K. | 10.1007/s00277-012-1522-2 |
| Artemisinin-resistant Plasmodium falciparum in Pursat province, western Cambodia: A parasite clearance rate study | Amaratunga C., Sreng S., Suon S., Phelps E.S., Stepniewska K., Lim P., Zhou C., Mao S., Anderson J.M., Lindegardh N., Jiang H., Song J., Su X.-Z., White N.J., Dondorp A.M., Anderson T.J.C., Fay M.P., Mu J., Duong S., Fairhurst R.M. | 10.1016/S1473-3099(12)70181-0 |
| Resistance to malaria in humans: The impact of strong, recent selection | Hedrick P.W. | 10.1186/1475-2875-11-349 |
| Prevalence of iron deficiency anaemia and thalassaemia trait among undergraduate medical students | Azma R.Z., Ainoon O., Azlin I., Hamenuddin H., Hadi N.A., Tatt W.K., Syazana I.N., Asmaliza A.M., Das S., Hamidah N.H. | |
| Development of a fluorescence immunochromatographic assay for the detection of zeta globin in the blood of (--SEA) Î±-thalassemia carriers | Wen L., Zhu P., Liu Y., Pan Q., Qu Y., Xu X., Li X., Fu N. | 10.1016/j.bcmd.2012.05.011 |
| Prevalence of Î²-thalassemia and hemoglobin e in two migrant populations of Manipur, North East India | Achoubi N., Asghar M., Saraswathy K.N., Murry B. | 10.1089/gtmb.2011.0373 |
| Molecular characterization of hemoglobin D Punjab traits and clinical-hematological profile of the patients [CaracterizaÃ§Ã£o molecular dos traÃ§os de hemoglobina D Punjab e perfil clÃnico-hematolÃ³gico dos pacientes] | Pandey S., Mishra R.M., Pandey S., Shah V., Saxena R. | 10.1590/S1516-31802012000400008 |
| Comparison of capillary electrophoregram among heterozygous Hb Hope, Hb Hope/Î±- | Pornprasert S., Panyasai S., Kongthai K. | 10.1515/cclm-2012-0016 |

| | | |
|---|---|---|
| thalassemia-1 SEA type deletion and Hb Hope/Î²0- thalassemia | | |
| Beta globin frameworks in thalassemia major patients from North Iran | Akhavan-Niaki H., Banihashemi A., Azizi M. | |
| Development of a fibrous DNA chip for cost-effective Î²-thalassemia genotyping | Suzuki W., Osaka T., Sekizawa A., Kitagawa M., Honma I. | 10.1007/s12185-012-1153-3 |
| A spurious haemoglobin A1c result associated with double heterozygote for haemoglobin Raleigh (Î²1[NA1]Val â†' Ala) and Î±+-thalassaemia | Singha K., Fucharoen G., Chaibunruang A., Netnee P., Fucharoen S. | 10.1258/acb.2012.011234 |
| The contribution of extramedullary hematopoiesis to hepatomegaly in anemic hydrops fetalis: A study in alpha-thalassemia hydrops fetalis | Taweevisit M., Thorner P.S. | 10.2350/11-12-1126-OA.1 |
| The spectrum of Î±-and Î²-thalassemia mutations in Yunnan Province of southwestern China | Zhang J., Zhu B.-S., He J., Zeng X.-H., Su J., Xu X.-H., Li S.-Y., Chen H., Zhang Y.-H. | 10.3109/03630269.2012.717327 |
| Unmasking Hb PaksÃ© (codon 142, TAA>TAT, Î±2) and its combinations in patients also carrying Hb constant spring (codon 142, TAA>CAA, Î±2) in Northern Thailand | Pornprasert S., Panyasai S., Treesuwan K. | 10.3109/03630269.2012.709896 |
| Interaction of -Î± 3.7, Î² thalassemia mutation IVS 1-5 and HbD Punjab in a family: A case report | Pandey S., Ranjan R., Mishra R.M., Pandey S.W., Saxena R. | 10.1007/s12291-012-0189-8 |
| Health related quality of life in Middle Eastern children with beta-thalassemia | Caocci G., Efficace F., Ciotti F., Roncarolo M.G., Vacca A., Piras E., Littera R., Markous R.S.D., Collins G.S., Ciceri F., Mandelli F., Marktel S., La Nasa G. | 10.1186/1471-2326-12-6 |
| Coinheritance of Hb S [Î²6(A3)Gluâ†'Val, GAG>GTG] with Î²0-Thalassemia codon 17 (A>T) in a thai patient | Pornprasert S., Panyasai S., Kongthai K., Treesuwan K. | 10.3109/03630269.2012.669358 |
| Frequency of seropositivity of hepatits C in thalasemia major patients | Siyal A.A., Dahri B.N., Balouch T.A., Siyal M.S. | |
| Microsatellite markers within the Î±-globin gene cluster for robust preimplantation genetic diagnosis of severe Î±-Thalassemia syndromes in mediterranean populations | Destouni A., Christopoulos G., Vrettou C., Kakourou G., Kleanthous M., Traeger-Synodinos J., Kanavakis E. | 10.3109/03630269.2012.666512 |
| Melioidosis: A review article | Besari A.M., Deris Z.Z., Hashim C.W.A. | |
| Clinical severity of Î²-thalassaemia/Hb e disease is associated with differential activities of the calpain-calpastatin proteolytic system | Sukati S., Svasti S., Stifanese R., Averna M., Panutdaporn N., Penglong T., Melloni E., Fucharoen S., Katzenmeier G. | 10.1371/journal.pone.0037133 |
| Prevalence of HBV and HCV infection among multi-transfused Egyptian thalassemic patients | Mansour A.K., Aly R.M., Abdelrazek S.Y., Elghannam D.M., Abdelaziz S.M., Shahine D.A., Elmenshawy N.M., Darwish A.M. | 10.5144/1658-3876.2012.54 |
| Screening for co-existence of Î±-thalassemia in Î²-thalassemia and in HbE heterozygotes | Tatu T., Khuntarak S., Suwannasin S., Kiewkarnkha T., Khamrin S., Kasinrerk W. | 10.1007/s12185-012-1039-4 |

| | | |
|---|---|---|
| via an enzyme-linked immunosorbent assay for Hb Bart's and embryonic Î¶-globin chain | | |
| Quantification of hemoglobin Constant Spring in heterozygote and homozygote by a capillary electrophoresis method | Pornprasert S., Panyasai S., Waneesorn J., Kongthai K., Singboottra P. | 10.1111/j.1751-553X.2011.01371.x |
| The definition and epidemiology of non-transfusion-dependent thalassemia | Weatherall D.J. | 10.1016/S0268-960X(12)70003-6 |
| Genetic hemoglobin disorders, infection, and deficiencies of iron and vitamin A determine anemia in young Cambodian children | George J., Yiannakis M., Main B., Devenish R., Anderson C., An U.S., Williams S.M., Gibson R.S. | 10.3945/jn.111.148189 |
| APASL consensus statements and management algorithms for hepatitis C virus infection | Omata M., Kanda T., Yu M.-L., Yokosuka O., Lim S.-G., Jafri W., Tateishi R., Hamid S.S., Chuang W.-L., Chutaputti A., Wei L., Sollano J., Sarin S.K., Kao J.-H., McCaughan G.W. | 10.1007/s12072-012-9342-y |
| Hemoglobin E syndromes in Pakistani population | Moiz B., Hashmi M.R., Nasir A., Rashid A., Moatter T. | 10.1186/1471-2326-12-3 |
| Classification of complete blood count and haemoglobin typing data by a C4.5 decision tree, a naÃ¯ve Bayes classifier and a multilayer perceptron for thalassaemia screening | Setsirichok D., Piroonratana T., Wongseree W., Usavanarong T., Paulkhaolarn N., Kanjanakorn C., Sirikong M., Limwongse C., Chaiyaratana N. | 10.1016/j.bspc.2011.03.007 |
| A transgenic mouse model expressing exclusively human hemoglobin E: Indications of a mild oxidative stress | Chen Q., Fabry M.E., Rybicki A.C., Suzuka S.M., Balazs T.C., Etzion Z., de Jong K., Akoto E.K., Canterino J.E., Kaul D.K., Kuypers F.A., Lefer D., Bouhassira E.E., Hirsch R.E. | 10.1016/j.bcmd.2011.12.002 |
| Limit of sensitivity for melting curve screening for Î±-thalassemia | Sriiam S., Leecharoenkiat A., Lithanatudom P., Munkongdee T., Svasti S., Smith D.R. | 10.5372/1905-7415.0601.134 |
| Development of control material for hemoglobin analysis | Pornprasert S., Kongthai K., Waneesorn J., Jaiping K., Treesuwan K. | 10.1515/cclm.2011.777 |
| Micromapping of thalassemia and hemoglobinopathies in diferent regions of Northeast Thailand and Vientaine, Laos People's Democratic Republic | Tritipsombut J., Sanchaisuriya K., Phollarp P., Bouakhasith D., Sanchaisuriya P., Fucharoen G., Fucharoen S., Schelp F.P. | 10.3109/03630269.2011.637149 |
| A novel test tube method of screening for hemoglobin E | Tatu T., Kasinrerk W. | 10.1111/j.1751-553X.2011.01357.x |
| Phenotypic expression of hemoglobins A2, E and F in various hemoglobin E related disorders | Sae-ung N., Srivorakun H., Fucharoen G., Yamsri S., Sanchaisuriya K., Fucharoen S. | 10.1016/j.bcmd.2011.09.008 |
| Erythroblast cell expansion as a marker for disease severity in Î² 0-thalassemia/Hb E disease | Leecharoenkiat A., Wannatung T., Smith D.R. | 10.5897/AJB11.1205 |

| | | |
|---|---|---|
| The prevalence of Î± thalassemia in South Western Maharashtra | Momin A.A., Bankar M.P., Bhoite G.M. | |
| New updating into hemoglobinopathies | Fucharoen S., Winichagoon P. | 10.1111/j.1751-553X.2012.01446.x |
| The importance of studying inherited hematological disorders in ancient Anatolian populations [Anadoludaki antik topluluklarda kali{dotless}tsal hematolojik bozukluklari{dotless}n Ã§ali{dotless}ÅŸi{dotless}lmasi{dotless}ni{dotless}n Ã¶nemi] | AlakoÃ§ Y.D., Akar N. | 10.5152/tjh.2011.43 |
| Alpha thalassemia major - A hematological prelude to an oncological problem | Pant S. | 10.1016/j.mehy.2011.07.037 |
| Hemoglobin Lepore EF Bart's disease: A molecular, hematological, and diagnostic aspects | Chaibunruang A., Fucharoen G., Jetsrisuparb A., Fucharoen S. | 10.1007/s00277-011-1173-8 |
| Sickle cell disease in Middle East Arab countries | El-Hazmi M.A.F., Al-Hazmi A.M., Warsy A.S. | |
| In silico analysis of single nucleotide polymorphism (snps) in human Î²-globin gene | Alanazi M., Abduljaleel Z., Khan W., Warsy A.S., Elrobh M., Khan Z., Amri A.A., Bazzi M.D. | 10.1371/journal.pone.0025876 |
| Increased oxidative metabolism is associated with erythroid precursor expansion in Î² 0-thalassaemia/Hb E disease | Leecharoenkiat A., Wannatung T., Lithanatudom P., Svasti S., Fucharoen S., Chokchaichamnankit D., Srisomsap C., Smith D.R. | 10.1016/j.bcmd.2011.06.005 |
| A rapid detection for Î±-thalassemia by PCR combined with dissociation curve analysis | Jia X., Liu J., Wang L., Yao L., Tang N., Cai R., Xiao B. | 10.1016/j.yexmp.2011.06.013 |
| Haemoglobinopathies in Southeast Asia | Fucharoen S., Winichagoon P. | |
| Hb E/beta-thalassaemia: A common & clinically diverse disorder | Olivieri N.F., Pakbaz Z., Vichinsky E. | |
| Î±-Hemoglobin stabilizing protein: A modulating factor in thalassemias? | Wajcman H., Vasseur C., Pissard S., Baudin-Creuza V. | 10.3109/03630269.2011.576354 |
| How I treat thalassemia | Rachmilewitz E.A., Giardina P.J. | 10.1182/blood-2010-08-300335 |
| Molecular analysis of globin gene expression in different thalassaemia disorders: Individual variation of Î²E pre-mRNA splicing determine disease severity | Tubsuwan A., Munkongdee T., Jearawiriyapaisarn N., Boonchoy C., Winichagoon P., Fucharoen S., Svasti S. | 10.1111/j.1365-2141.2011.08770.x |
| Preimplantation genetic diagnosis for Î±-and Î²-double thalassemia | Shen X., Xu Y., Zhong Y., Zhou C., Zeng Y., Zhuang G., Ding C., Li T. | 10.1007/s10815-011-9598-5 |
| Multicenter validation of fully automated capillary electrophoresis method for diagnosis of thalassemias and hemoglobinopathies in Thailand | Sangkitporn S., Sangkitporn S.K., Tanjatham S., Suwannakan B., Rithapirom S., Yodtup C., Yowang A., Duangruang S. | |

| | | |
|---|---|---|
| Genotype and phenotype characterizations in a large cohort of Î²-thalassemia heterozygote with different forms of Î²-thalassemia in northeast Thailand | Yamsri S., Sanchaisuriya K., Fucharoen G., Sae-ung N., Fucharoen S. | 10.1016/j.bcmd.2011.05.003 |
| Comparison between capillary electrophoresis and high performance liquid chromatography for detection and quantification of Hb constant spring [Hb CS; Î±142, Termâ†'Gln (TAA>CAA in Î±2)] | Waneesorn J., Panyasai S., Kongthai K., Singboottra P., Pornprasert S. | 10.3109/03630269.2011.588140 |
| Presumptive diagnosis of common haemoglobinopathies in Southeast Asia using a capillary electrophoresis system | Fucharoen G., Srivorakun H., Singsanan S., Fucharoen S. | 10.1111/j.1751-553X.2011.01301.x |
| Feasibility of preconception screening for thalassaemia in Indonesia: Exploring the opinion of Javanese mothers | Widayanti C.G., Ediati A., Tamam M., Faradz S.M.H., Sistermans E.A., Plass A.M.C. | 10.1080/13557858.2011.564607 |
| Accuracy of immunochromatographic strip test in diagnosis of alpha-thalassemia-1 carrier | Wanapirak C., Piyamongkol W., Sirichotiyakul S., Tayapiwatana C., Kasinrerk W., Tongsong T. | |
| Detection of Î±-thalassemia-1 Southeast Asian and Thai type deletions and Î²-thalassemia 3.5-kb deletion by single-tube multiplex real-time PCR with SYBR green 1 and high-resolution melting analysis | Pornprasert S., Wiengkum T., Srithep S., Chainoi I., Singboottra P., Wongwiwatthananukit S. | 10.3343/kjlm.2011.31.3.138 |
| Evaluation of the URIT-2900 Automated Hematology Analyzer for screening of thalassemia and hemoglobinopathies in Southeast Asian populations | Karnpean R., Pansuwan A., Fucharoen G., Fucharoen S. | 10.1016/j.clinbiochem.2011.04.009 |
| Interference of hemoglobin hope on Î²-thalassemia diagnosis by the capillary electrophoresis method | Panyasai S., Sukunthamala K., Jaiping K., Wongwiwatthananukit S., Singboottra P., Pornprasert S. | 10.1309/AJCP1BT2MGATKFHL |
| Hematological characteristics and effective screening for compound heterozygosity for Hb constant spring and deletional Î±+-thalassemia | Uaprasert N., Rojnuckarin P., Settapiboon R., Amornsiriwat S., Sutcharitchan P. | 10.1002/ajh.22033 |
| Complex interaction of hemoglobin (Hb) Nakhon Ratchasima [Î±63(E12)Alaâ†'Val], a novel Î±2-globin chain variant with Hb E [Î²26(B8)Gluâ†'Lys] and a deletional Î± +-thalassemia | Srivorakun H., Fucharoen G., Puangplruk R., Kheawon N., Fucharoen S. | 10.1111/j.1600-0609.2011.01616.x |
| Quantitative analysis of Hb Bart's in cord blood by capillary electrophoresis system | Munkongdee T., Pichanun D., Butthep P., Klamchuen S., Chalermpolprapa V., Winichagoon P., Svasti S., Fucharoen S. | 10.1007/s00277-010-1137-4 |
| A melting curve analysis-based PCR assay for one-step genotyping of Î²-thalassemia mutations: A multicenter validation | Xiong F., Huang Q., Chen X., Zhou Y., Zhang X., Cai R., Chen Y., Xie J., Feng S., Wei X., Xiao Q., Zhang T., Luo S., Yang X., Hao Y., Qu Y., Li Q., Xu X. | 10.1016/j.jmoldx.2011.03.005 |
| Phosphoproteomic analysis of apoptotic hematopoietic stem cells from hemoglobin E/Î²-thalassemia | Ponnikorn S., Panichakul T., Sresanga K., Wongborisuth C., Roytrakul S., Hongeng S., Tungpradabkul S. | 10.1186/1479-5876-9-96 |

| | | |
|---|---|---|
| Molecular lesion frequency of hemoglobin gene disorders in Taiwan | Liu S.-C., Peng C.-T., Lin T.-H., Wang S.-J., Shih M.-C., Tien N., Chang C.-C., Lu J.-J., Lin C.-Y. | 10.3109/03630269.2011.572524 |
| The impact of thalassemia on Southeast Asian and Asian Indian families in the United States: A qualitative study | Liem R.I., Gilgour B., Pelligra S.A., Mason M., Thompson A.A. | |
| Biologic characteristics of bone marrow-derived mesenchymal stem cells from a patient with thalassemia syndrome | Yoon J.S., Kim E.S., Hwang D.W., Choi J.Y., Kim B.K., Park B.B., Choi J.H., Lee Y.Y. | 10.1111/j.1751-553X.2010.01285.x |
| Anemia, iron deficiency and thalassemia among adolescents in Northeast Thailand: Results from two independent surveys | Pansuwan A., Fucharoen G., Fucharoen S., Himakhun B., Dangwiboon S. | 10.1159/000322666 |
| Thalassemia and hemoglobinopathies in Southeast Asian newborns: Diagnostic assessment using capillary electrophoresis system | Srivorakun H., Fucharoen G., Changtrakul Y., Komwilaisak P., Fucharoen S. | 10.1016/j.clinbiochem.2011.01.006 |
| Hb phimai [Î²72(E16)Serâ†’Thr]: A novel Î²-globin structural variant found in association with Hb constant spring in pregnancy | Singsanan S., Srivorakun H., Fucharoen G., Puangplruk R., Fucharoen S. | 10.3109/03630269.2011.557171 |
| Elevated hemoglobin A2 as a marker for Î²-thalassemia trait in pregnant women | Ou Z., Li Q., Liu W., Sun X. | 10.1620/tjem.223.223 |
| Molecular spectrum of Î±- and Î²-globin gene mutations detected in the population of Guangxi Zhuang Autonomous Region, People's Republic of China | Zheng C.-G., Liu M., Du J., Chen K., Yang Y., Yang Z. | 10.3109/03630269.2010.547429 |
| High prevalence of rare mutations in the beta Globin gene in an ethnic group in Iran | Galehdari H., Salehi B., Pedram M., Oraki Kohshour M. | |
| Molecular and hematological characterization of hemoglobin H disease in the Bengali population of Kolkata, India | Dastidar R., Gajra B., De M. | 10.1089/gtmb.2010.0062 |
| Adaptation to anemia in hemoglobin E-Î² thalassemia | Allen A., Fisher C., Premawardhena A., Peto T., Allen S., Arambepola M., Thayalsutha V., Olivieri N., Weatherall D. | 10.1182/blood-2010-06-289488 |
| Correlation of beta-thalassemia mutations with alpha-thalassemia: An experience of the southwestern region of Iran | Rahim F. | 10.1179/102453310X12719010991821 |
| HbE/Î²-thalassemia: Basis of marked clinical diversity | Olivieri N.F., Pakbaz Z., Vichinsky E. | 10.1016/j.hoc.2010.08.008 |
| Rapid identification of common Î²-thalassemia mutations in the Chinese population using duplex or triplex amplicon genotyping by high-resolution melting analysis | He X., Sheng M., Xu M., Xiong C., Ren Z. | 10.1089/gtmb.2010.0048 |
| Study of beta-thalassemia biomarkers and their relationship to cognition among children | Shehata G.A., Elsayh K.I., Rafet N.H., Mohamed A.O., Rageh T.A. | 10.1177/0883073810368996 |

| | | |
|---|---|---|
| Molecular screening of the Hbs Constant Spring (codon 142, TAA>CAA, Î±2) and PaksÃ© (codon 142, TAA>TAT, Î±2) mutations in Thailand | Pichanun D., Munkongdee T., Klamchuen S., Butthep P., Winichagoon P., Fucharoen S., Svasti S. | 10.3109/03630269.2010.526914 |
| ThalassoChip, an array mutation and single nucleotide polymorphism detection tool for the diagnosis of Î²-thalassaemia | Shammas C., Papasavva T., Felekis X., Christophorou C., Roomere H., Synodinos J.T., Kanavakis E., El-Khateeb M., Hamamy H., Mahmoud T., Shboul M., Beshlawy A.E., Filon D., Hussein I.R., Galanello R., Romeo G., Kleanthous M. | 10.1515/CCLM.2010.331 |
| Multiplexed bead-based mesofluidic system for gene diagnosis and genotyping | Jin S.-Q., Ye B.-C., Huo H., Zeng A.-J., Xie C.-K., Ren B.-Q., Huang H.-J. | 10.1021/ac1024792 |
| SYTO9 and SYBR GREEN1 with a high-resolution melting analysis for prenatal diagnosis of Î²0-thalassemia/hemoglobin-E | Pornprasert S., Sukunthamala K. | 10.1111/j.1600-0609.2010.01512.x |
| Thalassemia and iron deficiency in a group of northeast Thai school children: Relationship to the occurrence of anemia | Panomai N., Sanchaisuriya K., Yamsri S., Sanchaisuriya P., Fucharoen G., Fucharoen S., Schelp F.P. | 10.1007/s00431-010-1218-3 |
| Prevalence of fractures among Thais with Thalassaemia syndromes | Sutipornpalangkul W., Janechetsadatham Y., siritanaratkul N., Harnroongroj T. | |
| Diagnosis of thalassemia on dried blood spot samples by high performance liquid chromatography | Pornprasert S., Kasemrad C., Sukunthamala K. | 10.3109/03630269.2010.513294 |
| Hemoglobin Q-Thailand related disorders: Origin, molecular, hematological and diagnostic aspects | Singsanan S., Karnpean R., Fucharoen G., Sanchaisuriya K., Sae-ung N., Fucharoen S. | 10.1016/j.bcmd.2010.06.001 |
| Hemoglobin H disease in Guangxi province, Southern China: Clinical review of 357 patients | Yin X.-L., Zhang X.-H., Zhou T.-H., Zhang T.-L., Luo R.-G., Wang L., Zhou Y.-L., Chen Y.-S., Kong X.-J., Liang B., He Y.-Y., Peng L., Lu L.-B., Fang S.-P., Wu Z.-K. | 10.1159/000314058 |
| Hemoglobin H disease due to a de novo mutation at the Î±2-globin gene and an inherited common Î±-thalassemia deletion found in a Chinese boy | Zhu C., Yu W., Xie J., Chen L., Ding H., Shang X., Xu X. | 10.1016/j.bcmd.2010.07.005 |
| Complex interaction of Hb Q-Thailand and Hb e with Î±0- thalassemia and hereditary persistence of fetal hemoglobin in a Chinese family | Zheng W., Liu Y., Chen D., Rong K., Ge Y., Gong C., Chen H. | 10.1007/s00277-010-0935-z |
| A descriptive profile of Î²-thalassaemia mutations in India, Pakistan and Sri Lanka | Black M.L., Sinha S., Agarwal S., Colah R., Das R., Bellgard M., Bittles A.H. | 10.1007/s12687-010-0026-9 |
| High-throughput Î²-thalassemia carrier screening by allele-specific Q-primer real-time polymerase chain reaction | Liu X., Law H.Y., Tan Y.M., Hong Y. | 10.1016/j.ab.2010.04.025 |
| Hydrops fetalis in the stillborn: A series from the central region of Thailand | Taweevisit M., Thorner P.S. | 10.2350/09-12-0771-OA.1 |
| Application of an expanded multiplex genotyping assay for the simultaneous detection of Hemoglobin Constant Spring | Kidd J.L., Azimi M., Lubin B., Vichinsky E., Hoppe C. | 10.1111/j.1751-553X.2009.01197.x |

| | | |
|---|---|---|
| and common deletional Î±-thalassemia mutations | | |
| Hb Koya Dora [Î±142, Termâ†'Ser (TAA>TCA in Î±2)]: A rare mutation of the Î±2 gene stop codon associated with Î±-Thalassemia | Brennan S.O., Ryken S., Chan T. | 10.3109/03630269.2010.486344 |
| Large scale screening for haemoglobin disorders in southern Vietnam: Implications for avoidance and management | O'Riordan S., Hien T.T., Miles K., Allen A., Quyen N.N., Hung N.Q., Anh D.Q., Tuyen L.N., Khoa D.B., Thai C.Q., Triet D.M., Phu N.H., Dunstan S., Peto T., Clegg J., Farrar J., Weatherall D. | 10.1111/j.1365-2141.2010.08237.x |
| The inherited diseases of hemoglobin are an emerging global health burden | Weatherall D.J. | 10.1182/blood-2010-01-251348 |
| Sensitivity and specificity of simple erythrocyte osmotic fragility test for screening of alpha-thalassemia-1 and beta-thalassemia trait in pregnant women | Tongprasert F., Sirichotiyakul S., Piyamongkol W., Tongsong T. | 10.1159/000271779 |
| Molecular confirmatory testing of hemoglobin Constant Spring by real-time polymerase chain reaction SYBR Green1 with high-resolution melting analysis | Panyasai S., Sukunthamala K., Pornprasert S. | 10.1111/j.1600-0609.2010.01437.x |
| Prevention of severe thalassemia in northeast Thailand: 16 Years of experience at a single university center | Yamsri S., Sanchaisuriya K., Fucharoen G., Sae-ung N., Ratanasiri T., Fucharoen S. | 10.1002/pd.2514 |
| Fermented papaya preparation for Î'-thalassemia? | Olalla-Saad S.T. | 10.1586/ehm.10.23 |
| The profile of free amino acids in latent fingerprint of healthy and beta-thalassemic volunteers | Khedr A. | 10.1016/j.jchromb.2010.04.017 |
| Emergence of pediatric melioidosis in Siem Reap, Cambodia | Pagnarith Y., Kumar V., Thaipadungpanit J., Wuthiekanun V., Amornchai P., Sin L., Day N.P., Peacock S.J. | 10.4269/ajtmh.2010.10-0030 |
| Î±-thalassaemia | Harteveld C.L., Higgs D.R. | 10.1186/1750-1172-5-13 |
| Antenatal screening for haemoglobinopathies in primary care: A cohort study and cluster randomised trial to inform a simulation model. The screening for haemoglobinopathies in first trimester (SHIFT) trial | Dormandy E., Bryan S., Gulliford M.C., Roberts T.E., Ades A.E., Calnan M., Atkin K., Karnon J., Barton P.M., Logan J., Kavalier F., Harris H.J., Johnston T.A., Anionwu E.N., Davis V., Brown K., Juarez-Garcia A., Tsianakas V., Marteau T.M. | 10.3310/hta14200 |
| Improvement of Î±o-thalassemia screening using combined osmotic fragility, dichlorophenolindophenol and Hb H inclusion tests | Chaibunruang A., Pornphannukool S., Sae-Ung N., Fucharoen G., Sanchaisuriya K., Fucharoen S. | |
| A comprehensive, simple molecular assay of common deletions and mutations causing a-thalassemia in Southeast Asia and southern China | Liu J., Jia X., Tang N., Wang L., Han H., Cai R., Wang Q., Xiao B. | 10.1002/ajh.21671 |

| | | |
|---|---|---|
| Rapid diagnosis of Î±-thalassemia by melting curve analysis | Munkongdee T., Vattanaviboon P., Thummarati P., Sewamart P., Winichagoon P., Fucharoen S., Svasti S. | 10.2353/jmoldx.2010.090136 |
| Î²-thalassemia cardiomyopathy: History, present considerations, and future perspectives | Kremastinos D.T., Farmakis D., Aessopos A., Hahalis G., Hamodraka E., Tsiapras D., Keren A. | 10.1161/CIRCHEARTFAILURE.109.913863 |
| A mechanism of ineffective erythropoiesis in Î²-thalassemia/Hb E disease | Lithanatudom P., Leecharoenkiat A., Wannatung T., Svasti S., Fucharoen S., Smith D.R. | 10.3324/haematol.2009.015701 |
| Detection of paternal alleles in maternal plasma for non-invasive prenatal diagnosis of Î²-thalassemia: A feasibility study in southern Chinese | Chan K., Yam I., Leung K.Y., Tang M., Chan T.K., Chan V. | 10.1016/j.ejogrb.2010.02.016 |
| High resolution DNA melting analysis: An application for prenatal control of Î±-thalassemia | Sirichotiyakul S., Wanapirak C., Saetung R., Sanguansermsri T. | 10.1002/pd.2480 |
| Compound Heterozygous Hb TakHb e Causes Secondary Erythrocytosis in a Thai Family | Teawtrakul N., Sirijirachai C., Chansung G., Fucharoen G. | 10.3109/03630261003680498 |
| Prenatal diagnosis of hemoglobin Bart's disease: What is the noninvasive approach? | Li D.-Z. | 10.1002/pd.2477 |
| Interactions of hemoglobin Lepore (Î´Î² hybrid hemoglobin) with various hemoglobinopathies: A molecular and hematological characteristics and differential diagnosis | Chaibunruang A., Srivorakun H., Fucharoen S., Fucharoen G., Sae-ung N., Sanchaisuriya K. | 10.1016/j.bcmd.2009.11.008 |
| Global burden, distribution and prevention of Î²-thalassemias and hemoglobin e disorders | Colah R., Gorakshakar A., Nadkarni A. | 10.1586/ehm.09.74 |
| Molecular diversity of hemoglobin H disease in India | Nadkarni A.H., Nair S.B., Italia K.Y., Warang P., Dalvi M., Ghosh K., Colah R.B. | 10.1309/AJCP70ORBRURVSJM |
| Sandwich ELISA for hemoglobin A2 quantification and identification of Î²-thalassemia carriers | Kuntaruk S., Tatu T., Keowkarnkah T., Kasinrerk W. | 10.1007/s12185-009-0490-3 |
| Hematological features and molecular lesions of hemoglobin gene disorders in Taiwanese patients | Lin H.-J., Shih M.-C., Peng C.-T., Liu T.-C., Chen K.-W., Shih H.-C., Chang J.-G. | 10.1111/j.1751-553X.2008.01095.x |
| Hemoglobinopathies in North Africa: A review | Khelil A.H., Denden S., Leban N., Daimi H., Lakhdhar R., Lefranc G., Ben Chibani J., Perrin P. | 10.3109/03630260903571286 |
| Whole blood and component use in resource poor settings | Marwaha N. | 10.1016/j.biologicals.2009.10.020 |
| Non-haem iron-mediated oxidative stress in Haemoglobin E beta-thalassaemia | Chakraborty I., Mitra S., Gachhui R., Kar M. | |

| | | |
|---|---|---|
| Classification of haemoglobin typing chromatograms by neural networks and decision trees for thalassaemia screening | Piroonratana T., Wongseree W., Assawamakin A., Paulkhaolarn N., Kanjanakorn C., Sirikong M., Thongnoppakhun W., Limwongse C., Chaiyaratana N. | 10.1016/j.chemolab.2009.07.014 |
| Incidence of haemoglobinopathies in various populations - The impact of immigration | Henderson S., Timbs A., McCarthy J., Gallienne A., Van Mourik M., Masters G., May A., Khalil M.S.M., Schuh A., Old J. | 10.1016/j.clinbiochem.2009.05.012 |
| SYTO9 and SYBR green1 with high resolution melting analysis for molecular confirmatory testing of the common Southeast Asian Î²0- thalassemia mutations | Chamras U., Sukunthamala K., Pornprasert S. | 10.3109/03630260903336560 |
| Beta globin gene cluster haplotypes of the beta thalassemia mutations observed in the Denizli province of Turkey | Bahadir A., Ã–ztÃ¼rk O., Atalay A., Atalay E.Ã–. | |
| Rapid diagnosis of the Î±-thalassemia-1 Southeast Asian type deletion using a single tube real-time SYBR-polymerase chain reaction combined with dissociation curve analysis | Liu J.-Z., Xiao B., Wang Q.-T., Wang L.-R. | 10.3109/03630260903337055 |
| Drug metabolizing enzyme CYP1A2 status in pediatric patients with hemoglobin E-Î² thalassemia | Senggunprai L., Kukongviriyapan U., Jetsrisuparb A., Kukongviriyapan V. | |
| Complex interaction of Hb e [Î²26(B8)Gluâ†'Lys], Hb Korle-Bu [Î²73(E17)Aspâ†'Asn] and a deletional Î±-thalassemia-1 in pregnancy | Siriratmanawong N., Chansri W., Singsanan S., Fucharoen G., Fucharoen S. | 10.3109/03630260903343780 |
| Hb Owari associated with Î±-thalassaemia-1 in a Taiwanese subject | Wang L.-H., Chang C.-S., Hsieh L.-L., Er T.-K., Chang J.-G., Liu T.-C. | |
| The significance of the hemoglobin A2 value in screening for hemoglobinopathies | Giambona A., Passarello C., Renda D., Maggio A. | 10.1016/j.clinbiochem.2009.06.026 |
| Prospective and retrospective primary prevention of Hemoglobinopathies in multiethnic societies | Giordano P.C. | 10.1016/j.clinbiochem.2009.06.027 |
| Genetic recombination as a major cause of mutagenesis in the human globin gene clusters | Borg J., Georgitsi M., Aleporou-Marinou V., Kollia P., Patrinos G.P. | 10.1016/j.clinbiochem.2009.07.014 |
| Hemoglobin H-constant spring in North America: An alpha thalassemia with frequent complications | Singer S.T., Kim H.-Y., Olivieri N.F., Kwiatkowski J.L., Coates T.D., Carson S., Neufeld E., Cunningham M.J., Giardina P.J., Mueller B.U., Quinn C.T., Fung E., Vichinsky E. | 10.1002/ajh.21523 |
| Iron chelation therapy in the management of thalassemia: The Asian perspectives | Viprakasit V., Lee-Lee C., Chong Q.T., Lin K.-H., Khuhapinant A. | 10.1007/s12185-009-0432-0 |
| Alpha globin gene numbers: An important modifier of HbE/Î² thalassemia | Sharma V., Kumar B., Kumar G., Saxena R. | 10.1179/102453309X446126 |

| | | |
|---|---|---|
| A comparison of the accuracy of the corpuscular fragility and mean corpuscular volume tests for the alpha-thalassemia 1 and beta-thalassemia traits | Sirichotiyakul S., Wanapirak C., Srisupundit K., Luewan S., Tongsong T. | 10.1016/j.ijgo.2009.05.012 |
| Letter to the editor | Li D.-Z. | 10.1111/j.1751-553X.2008.01074.x |
| Improvement in the detection of Î±0- and deletional Î±-thalassemia by real-time pcr combined with dissociation curve analysis | Liu J., Tang N., Liu Q., Wang L., Han H., Cai R., Wu X., Xiao B. | 10.1159/000232578 |
| Comparison of the accuracy of dichlorophenolindophenol (DCIP), modified DCIP, and hemoglobin E tests to screen for the HbE trait in pregnant women | Wanapirak C., Sirichotiyakul S., Luewan S., Srisupundit K., Tongsong T. | 10.1016/j.ijgo.2009.04.020 |
| Pharmaco/ferrokinetic-related pro-oxidant activity of deferiprone in Î²-thalassemia | Jirasomprasert T., Morales N.P., Limenta L.M.G., Sirijaroonwong S., Yamanont P., Wilairat P., Fucharoen S., Chantharaksri U. | 10.1080/10715760902870611 |
| Variable clinical phenotypes of Î±-thalassemia syndromes | Singer S.T. | 10.1100/tsw.2009.69 |
| Increased Hb A2 values in an HIV-1-infected patient receiving antiretroviral drugs: A pitfall for thalassemia antenatal diagnosis | Pornprasert S., Sukunthamala K., Leechanachai P., Sanguansermsri T. | 10.1080/03630260902813486 |
| Analysis of fetal blood using capillary electrophoresis system: A simple method for prenatal diagnosis of severe thalassemia diseases | Srivorakun H., Fucharoen G., Sae-Ung N., Sanchaisuriya K., Ratanasiri T., Fucharoen S. | 10.1111/j.1600-0609.2009.01245.x |
| Accurate prenatal diagnosis of Hb Bart's hydrops fetalis in daily practice with a double-check PCR system | Karnpean R., Fucharoen G., Fucharoen S., Sae-Ung N., Sanchaisuriya K., Ratanasiri T. | 10.1159/000225930 |
| Simple, efficient, and cost-effective multiplex genotyping with matrix assisted laser desorption/ionization time-of-flight mass spectrometry of hemoglobin beta gene mutations | Thongnoppakhun W., Jiemsup S., Yongkiettrakul S., Kanjanakorn C., Limwongse C., Wilairat P., Vanasant A., Rungroj N., Yenchitsomanus P.-T. | 10.2353/jmoldx.2009.080151 |
| Use of HbA estimation by CE-HPLC for prenatal diagnosis of Î²-thalassemia; experience from a tertiary care centre in north India: A brief report | Rao S., Saxena R., Deka D., Kabra M. | 10.1179/102453309X385269 |
| Thalassemia intermedia in HbH-CS disease with compound heterozygosity for Î²-thalassemia: Challenges in hemoglobin analysis and clinical diagnosis | Tan J.A.M.A., Juan L.K., Kim L.T., Yong C.W., George E. | 10.1266/ggs.84.67 |
| Simple method for screening of Î±-thalassaemia 1 carriers | Tayapiwatana C., Kuntaruk S., Tatu T., Chiampanichayakul S., Munkongdee T., Winichagoon P., Fuchareon S., Kasinrerk W. | 10.1007/s12185-009-0331-4 |
| False positive rates of thalassemia screening in rural clinical setting: 10-year experience in Thailand | Jopang Y., Thinkhamrop B., Puangpruk R., Netnee P. | |

| | | |
|---|---|---|
| Elevated serum transferrin receptor levels in common types of thalassemia heterozygotes in Southeast Asia: A correlation with genotypes and red cell indices | Uaprasert N., Rojnuckarin P., Bhokaisawan N., Settapiboon R., Wacharaprechanont T., Amornsiriwat S., Sutcharitchan P. | 10.1016/j.cca.2009.01.031 |
| Clinical features and molecular analysis in Thai patients with HbH disease | Laosombat V., Viprakasit V., Chotsampancharoen T., Wongchanchailert M., Khodchawan S., Chinchang W., Sattayasevana B. | 10.1007/s00277-009-0743-5 |
| Modification of platelet shape change parameter by oxidized lipoprotein from Î²-thalassemia/hemoglobin E | Sutipornpalangkul W., Unchern S., Sanvarinda Y., Chantharaksri U., Fucharoen S. | |
| Newborn screening for hemoglobinopathies in california | Michlitsch J., Azimi M., Hoppe C., Walters M.C., Lubin B., Lorey F., Vichinsky E. | 10.1002/pbc.21883 |
| Perinatal zidovudine prophylaxis in HIV type-1-infected pregnant women with thalassaemia carriage in Thailand | Briand N., Pornprasert S., Ngo-Giang-Huong N., GalactÃ©ros F., Pissara S., Tatu T., Sanguansermsri T., Jourdoin G., Lallemant M., Le Coeur S. | |
| Effect of Î±-gene numbers on phenotype of HbE/Î² thalassemia patients | Sharma V., Saxena R. | 10.1007/s00277-009-0723-9 |
| Clinical and hematological phenotype of homozygous hemoglobin E: revisit of a benign condition with hidden reproductive risk | Tachavanich K., Viprakasit V., Chinchang W., Glomglao W., Pung-Amritt P., Tanphaichitr V.S. | |
| Detection and identification of Î²-thalassemia 3.5 kb deletion by SYBR Green1 and high resolution melting analysis | Prathomtanapong P., Pornprasert S., Phusua A., Suanta S., Saetung R., Sanguansermsri T. | 10.1111/j.1600-0609.2008.01173.x |
| Detection of Hb e mutation (Î²26, GAG-AAG, Glu-Lys) using allelic discrimination analysis | Sangkitporn S., Sangkitporn S.K., Sangnoi A., Duangruang S. | 10.1111/j.1751-553X.2007.01006.x |
| Prenatal diagnosis of Î²-thalassemia/Hb E by hemoglobin typing compared to DNA analysis | Sirichotiyakul S., Saetung R., Sanguansermsri T. | 10.1080/03630260802626046 |
| Development and validation of a Î¶-globin-specific ELISA for carrier screening of the (--SEA) Î± thalassaemia deletion | Tang L., Zhu P., Zhou W.J., Zheng J., Zhou Y.Q., Fu N., Xu X.M. | 10.1136/jcp.2008.059477 |
| RNA repair restores hemoglobin expression in IVS2-654 thalassemic mice | Svasti S., Suwanmanee T., Fucharoen S., Moulton H.M., Nelson M.H., Maeda N., Smithies O., Kole R. | 10.1073/pnas.0812436106 |
| Sensitivity and specificity of mean corpuscular hemoglobin (MCH): For screening alpha-thalassemia-1 trait and beta-thalassemia trait | Pranpanus S., Sirichotiyakul S., Srisupundit K., Tongsong T. | |
| Molecular, hematological and clinical aspects of thalassemia major and thalassemia intermedia associated with Hb E-Î²-thalassemia in Northeast Thailand | Nuntakarn L., Fucharoen S., Fucharoen G., Sanchaisuriya K., Jetsrisuparb A., Wiangnon S. | 10.1016/j.bcmd.2008.09.002 |
| Renal tubule function in beta-thalassemia after hematopoietic stem cell transplantation | Sumboonnanonda A., Sanpakit K., Piyaphanee N. | 10.1007/s00467-008-0949-0 |

| | | |
|---|---|---|
| Evolution of hematological parameters in hiv-1-infected patients with and without thalassemia carriages during highly active antiretroviral therapy | Pornprasert S., Sonboon P., Kiatwattanacharoen S., Klinbuayaem V., Leenasirimakul P., Promping C., Inta P., Ajhan S., Leechanachai P. | 10.1310/hct1002-88 |
| Hb G-Waimanalo: occurrence in combination with alpha-thalassemia-1 Southeast Asian deletion | Lin M., Wu J.-R., Yang L.-Y., Chen H., Wang P.-P., Wang Q., Zheng L. | 10.1016/j.bcmd.2008.09.005 |
| Detection of beta-globin gene mutations among Kelantan Malay thalassaemia patients by polymerase chain reaction restriction fragment length polymorphism | Rozitah R., Nizam M.Z., Nur Shafawati A.R., Nor Atifah M.A., Dewi M., Kannan T.P., Ariffin N., Norsarwany M., Setianingsih I., Harahap A., Zilfalil B.A. | |
| Genetic modifiers in hemoglobinopathies | Rund D., Fucharoen S. | 10.2174/156652408786241410 |
| Accuracy of serum transferrin receptor levels in the diagnosis of iron deficiency among hospital patients in a population with a high prevalence of thalassaemia trait | Ong K.H., Tan H.L., Tam L.P., Hawkins R.C.W., Kuperan P. | 10.1111/j.1751-553X.2007.00982.x |
| Hemoglobin profiles and hematologic features of thalassemic newborns: Application to screening of Î±-thalassemia 1 and hemoglobin E | Tritipsombut J., Sanchaisuriya K., Fucharoen S., Fucharoen G., Siriratmanawong N., Pinmuang-ngam C., Sanchaisuriya P. | 10.1043%2F1543-2165-132.11.1739 |
| Comparison of real-time polymerase chain reaction SYBR Green1 with high resolution melting analysis and TaqMan MGB probes for detection of Î±-thalassemia-1 South-East Asian type on dried blood spots | Pornprasert S., Kaewbundit A., Phusua A., Suanta S., Saetung R., Sanguansermsri T. | 10.1111/j.1600-0609.2008.01137.x |
| Low cost combination of DCIP and MCV was better than that of DCIP and OF in the screening for hemoglobin E | Prayongratana K., Polprasert C., Raungrongmorakot K., Tatone K., Santiwatanakul S. | |
| Rapid diagnosis of thalassemias and other hemoglobinopathies by capillary electrophoresis system | Winichagoon P., Svasti S., Munkongdee T., Chaiya W., Boonmongkol P., Chantrakul N., Fucharoen S. | 10.1016/j.trsl.2008.08.004 |
| Separation of haemoglobin HbE and HbA2 by the fully automated, high-pressure liquid chromatography Tosoh HLC-723 G7 analyzer | Lippi G., Carta M.R., Salvagno G.L., Bellorio F., Montagnana M., Soffiati G., Guidi G.C. | 10.1111/j.1751-553X.2007.00988.x |
| Hematopoietic stem cell transplantation in Thailand | Issaragrisil S. | 10.1038/bmt.2008.142 |
| Hb constant spring [Î±142, termâ†’Gln (TAA>CAA in Î±2)] in the Î±-thalassemia of anemic patients in Myanmar | Ne-Win, Harano K., Kyaw-Shwe, Aye-Aye-Myint, Khin-Thander-Aye, Okada S., Harano T. | 10.1080/03630260802341588 |
| First report of a nonsense mutation at codon 15(TGG â†’ TAG) in exon 1 of the Î² globin gene in a Î² thalassemia trait in State of Orissa, India | Nishank S.S., Ranjit M., Chhotray G.P. | 10.1179/102453308X315852 |

| | | |
|---|---|---|
| Co-inheritance of Î±-and Î²-thalassemia in Khuzestan Province, Iran | Rahim F., Kaikhaei B., Zandian K., Hoseini A. | 10.1179/102453308X315843 |
| Comparison of the mismatch-specific endonuclease method and denaturing high-performance liquid chromatography for the identification of HBB gene mutations | Hung C.-C., Su Y.-N., Lin C.-Y., Chang Y.-F., Chang C.-H., Cheng W.-F., Chen C.-A., Lee C.-N., Lin W.-L. | 10.1186/1472-6750-8-62 |
| Thalassemia and hemoglobinopathies in pregnant Lao women: Carrier screening, prevalence and molecular basis | Savongsy O., Fucharoen S., Fucharoen G., Sanchaisuriya K., Sae-ung N. | 10.1007/s00277-008-0490-z |
| Molecular mechanism of Î²-thalassaemia caused by 22-bp duplication | Svasti S., Boonchoy C., Vanichsetakul P., Winichagoon P., Fucharoen S. | 10.1007/s00277-008-0479-7 |
| Alpha-thalassaemia | Leung W.C., Leung K.Y., Lau E.T., Tang M.H.Y., Chan V. | 10.1016/j.siny.2008.02.006 |
| Detection of Î²-thalassemia mutations using a multiplex amplification refractory mutation system assay | Mirasena S., Shimbhu D., Sanguansermsri M., Sanguansermsri T. | 10.1080/03630260701798391 |
| Effective screening for double heterozygosity of Hb E/Î±0 -thalassemia | Sanchaisuriya K., Chirakul S., Srivorakun H., Fucharoen G., Fucharoen S., Changtrakul Y., Sanchaisuriya P. | 10.1007/s00277-008-0520-x |
| A scoring system for the classification of Î²-thalassemia/Hb E disease severity | Sripichai O., Makarasara W., Munkongdee T., Kumkhaek C., Nuchprayoon I., Chuansumrit A., Chuncharunee S., Chantrakoon N., Boonmongkol P., Winichagoon P., Fucharoen S. | 10.1002/ajh.21130 |
| Detection of alpha-thalassemia-1 Southeast Asian type using real-time gap-PCR with SYBR Green1 and high resolution melting analysis | Pornprasert S., Phusua A., Suanta S., Saetung R., Sanguansermsri T. | 10.1111/j.1600-0609.2008.01055.x |
| Hematological abnormalities in patients with distal renal tubular acidosis and hemoglobinopathies | Khositseth S., Sirikanaerat A., Khoprasert S., Opastirakul S., Kingwatanakul P., Thongnoppakhun W., Yenchitsomanus P.-T. | 10.1002/ajh.21151 |
| Genetic disorders and malaria in Indo-China region | Wiwanitkit V. | |
| Increased urinary 1,N6-ethenodeoxyadenosine and 3,N4-ethenodeoxycytidine excretion in thalassemia patients: Markers for lipid peroxidation-induced DNA damage | Meerang M., Nair J., Sirankapracha P., Thephinlap C., Srichairatanakool S., Fucharoen S., Bartsch H. | 10.1016/j.freeradbiomed.2008.02.009 |
| Detection and haplotype differentiation of Southeast Asian Î±-thalassemia using polymerase chain reaction and a piezoelectric biosensor immobilized with a single oligonucleotide probe | Vattanaviboon P., Sangseekhiow K., Winichagoon P., Promptmas C. | 10.1016/j.trsl.2007.12.009 |
| Mechanisms of and obstacles to iron cardiomyopathy in thalassemia | Peng C.-T., Chang J.-S., Wu K.-H., Tsai C.-H., Lin H.-S. | 10.2741/3130 |
| Evidence of gene conversion in the evolutionary process of the codon 41/42 (- | Zhang W., Cai W.-W., Zhou W.-P., Li H.-P., Li L., Yan W., Deng Q.-K., Zhang Y.-P., Fu Y.-X., Xu X.-M. | 10.1007/s00239-008-9096-2 |

| | | |
|---|---|---|
| CTTT) mutation causing Î²-thalassemia in southern China | | |
| Studies in haemoglobin E beta-thalassaemia | Olivieri N.F., Muraca G.M., O'Donnell A., Premawardhena A., Fisher C., Weatherall D.J. | 10.1111/j.1365-2141.2008.07126.x |
| Spectrum of Î²-thalassemia mutations in various ethnic regions of Iran | Rahim F., Abromand M. | |
| Alpha-thalassaemia in association with beta-thalassaemia patients in Malaysia: A study on the co-inheritance of both disorders | Wee Y.C., Tan K.L., Kuldip K., Tai K.S., George E., Tan P.C., Chia P., Subramaniam R., Yap S.F., Tan J.A.M.A. | 10.1159/000113874 |
| Thalassemia and its relevance to personalized medicine | Kleanthous M., Phylactides M. | 10.2217/17410541.5.2.141 |
| Molecular evidences of single mutational events followed by recurrent crossing-overs in the common Î´-globin alleles in the Mediterranean area | Lacerra G., Musollino G., Scarano C., Lagona L.F., Caruso D.G., Testa R., Prezioso R., Di Noce F., Medulla E., Friscia M.G., Mastrullo L., Caldora M., Nota L., Gaudiano C., Magnano C., Ciaccio C., Romeo M.A., Carestia C. | 10.1016/j.gene.2007.12.004 |
| Host erythrocyte polymorphisms and exposure to Plasmodium falciparum in Papua New Guinea | Fowkes F.J.I., Michon P., Pilling L., Ripley R.M., Tavul L., Imrie H.J., Woods C.M., Mgone C.S., Luty A.J.F., Day K.P. | 10.1186/1475-2875-7-1 |
| A multicenter trial of the effectiveness of Î¶-globin enzyme-linked immunosorbent assay and hemoglobin h inclusion body screening for the detection of Î±0-thalassemia trait | Lafferty J.D., Barth D.S., Sheridan B.L., McFarlane A.G., Halchuk L.M., Raby A., Crowther M.A. | 10.1309/MNPF3XXXVAX5NM9H |
| Î²-Thalassemia major births after national screening program in Taiwan | Chern J.P.S., Lin K.-H., Lu M.-Y., Lin D.-T., Jou S.-T., Yang Y.-L., Chang H.-H., Su S., Lin K.-S. | 10.1002/pbc.21185 |
| Endothelial dysfunction and oxidant status in pediatric patients with hemoglobin E-Î² thalassemia | Kukongviriyapan V., Somparn N., Senggunprai L., Prawan A., Kukongviriyapan U., Jetsrisuparb A. | 10.1007/s00246-007-9107-x |
| Î±/Î²-Globin mRNA ratio determination by multiplex quantitative real-time reverse transcription-polymerase chain reaction as an indicator of globin gene function | Chaisue C., Kitcharoen S., Wilairat P., Jetsrisuparb A., Fucharoen G., Fucharoen S. | 10.1016/j.clinbiochem.2007.08.005 |
| Application of maternal plasma DNA analysis for noninvasive prenatal diagnosis of Hb E-Î²-thalassemia | Tungwiwat W., Fucharoen G., Fucharoen S., Ratanasiri T., Sanchaisuriya K., Sae-Ung N. | 10.1016/j.trsl.2007.06.006 |
| Iron deficiency among children of asylum seekers in The Netherlands | Stellinga-Boelen A.A.M., Storm H., Wiegersma P.A., Bijleveld C.M.A., Verkade H.J. | 10.1097/MPG.0b013e31810e76a5 |
| Multicenter study of the molecular basis of thalassemia intermedia in different ethnic populations | Verma I.C., Kleanthous M., Saxena R., Fucharoen S., Winichagoon P., Raizuddin S., Khan S.N., Akbari M.T., Izadyar M., Kotea N., Old J.M., Ioannou P.A., Khan B. | 10.1080/03630260701641245 |
| Prenatal control of Hb Bart's disease in southern China | Liao C., Li Q., Wei J., Feng Q., Li J., Huang Y., Li D. | 10.1080/03630260701634463 |
| Molecular characterization and origins of Hb constant spring and Hb PaksÃ© in Southeast Asian populations | Singsanan S., Fucharoen G., Savongsy O., Sanchaisuriya K., Fucharoen S. | 10.1007/s00277-007-0310-x |

| | | |
|---|---|---|
| Hemoglobin H disease induced by the common SEA deletion and the rare hemoglobin Quong Sze in a Thai female: Longitudinal clinical course, molecular characterization, and development of a PCR/RFLP-based detection method | Sura T., Trachoo O., Viprakasit V., Vathesatogkit P., Tunteeratum A., Busabaratana M., Wisedpanichkij R., Isarangkura P. | 10.1007/s00277-007-0303-9 |
| H63D mutation of the hemochromatosis gene and serum ferritin levels in Thai thalassemia carriers | Yamsri S., Sanchaisuriya K., Fucharoen S., Fucharoen G., Jetsrisuparb A., Wiangnon S., Changtrakul Y., Sanchaisuriya P. | 10.1159/000105677 |
| G6PD deficiency: the genotype-phenotype association | Mason P.J., Bautista J.M., Gilsanz F. | 10.1016/j.blre.2007.05.002 |
| Hemoglobin E disease in North Indian population: A report of 11 cases | Kishore B., Khare P., Gupta R.J., Bisht S., Majumdar K. | 10.1080/10245330701255247 |
| Sickle cell-haemoglobin E (HbSE) compound heterozygosity: A clinical and haematological study | Knox-Macaulay H.H.M., Ahmed M.M., Gravell D., Al-Kindi S., Ganesh A. | 10.1111/j.1365-2257.2006.00886.x |
| Molecular assay of -Î±3.7 and -Î±4.2 deletions causing Î±-thalassemia by denaturing high-performance liquid chromatography | Hung C.-C., Lee C.-N., Chen C.-P., Jong Y.-J., Hsieh W.-S., Lin W.-L., Su Y.-N., Hsu S.-M. | 10.1016/j.clinbiochem.2007.03.018 |
| Severe Î²0 thalassemia/hemoglobin E disease caused by de novo 22-base pair duplication in the paternal allele of Î² globin gene | Rojnuckarin P., Settapiboon R., Vanichsetakul P., Sueblinvong T., Sutcharitchan P. | 10.1002/ajh.20816 |
| Hemoglobin SE disease - A concise review | Masiello D., Heeney M.M., Adewoye A.H., Eung S.H., Luo H.-Y., Steinberg M.H., Chui D.H.K. | 10.1002/ajh.20847 |
| Prenatal diagnosis of Hb Bart's hydrops fetalis caused by a genetic compound heterozygosity for two different Î±o-thalassemia determinants | Siriratmanawong N., Pinmuang-Ngam C., Fucharoen G., Fucharoen S. | 10.1159/000100787 |
| Age-related changes in adaptation to severe anemia in childhood in developing countries | O'Donnell A., Premawardhena A., Arambepola M., Allen S.J., Peto T.E.A., Fisher C.A., Rees D.C., Olivieri N.F., Weatherall D.J. | 10.1073/pnas.0703424104 |
| Expression of Î²E and Î³-globin genes in infants heterozygous for hemoglobin E and double heterozygous for hemoglobin E and Î±-thalassemia | Winichagoon P., Svasti S., Winichagoon P., Chitchumroonchokchai C., Fucharoen S. | 10.3324/haematol.10930 |
| Validation of a reverse-hybridization StripAssay for the simultaneous analysis of common Î±-thalassemia point mutations and deletions | Puehringer H., Najmabadi H., Law H.-Y., Krugluger W., Viprakasit V., Pissard S., Baysal E., Taher A., Farra C., Al-Ali A., Al-Ateeq S., Oberkanins C. | 10.1515/CCLM.2007.125 |
| Coexistence of Southeast Asian ovalocytosis and Î²-thalassemia: A molecular and hematological analysis | Fucharoen G., Fucharoen S., Singsanan S., Sanchaisuriya K. | 10.1002/ajh.20818 |
| Identification of hemoglobin AC heterozygote status in a malay family: A decision between hemoglobin | Rosline H., Roshan T.M., Ahmed S.A., Ilunihayati I. | |

| | | |
|---|---|---|
| electrophoresis and high performance liquid chromotography | | |
| Genetic benefits of consanguinity through selection of genotypes protective against malaria | Denic S., Nicholls M.G. | |
| Effects of combined UDP-glucuronosyltransferase (UGT) 1A1*28 and 1A6*2 on paracetamol pharmacokinetics in Î²-thalassemia/HbE | Tankanitlert J., Morales N.P., Howard T.A., Fucharoen P., Ware R.E., Fucharoen S., Chantharaksri U. | 10.1159/000097908 |
| Genotypes of hepatitis C virus (HCV) among positive Lebanese patients: Comparison of data with that from other Middle Eastern countries | Sharara A.I., Ramia S., Ramlawi F., Fares J.E., Klayme S., Naman R. | 10.1017/S0950268806006911 |
| Molecular analysis of Î±/Î²-thalassemia in a Southern Chinese population | Ye B.-C., Zhang Z., Lei Z. | 10.1089/gte.2006.0502 |
| Prevalence of Î²-thalassaemia in subcastes of Indian Sindhis: Results from a two-phase survey | Jawahirani A., Mamtani M., Das K., Rughwani V., Kulkarni H. | 10.1016/j.puhe.2006.10.017 |
| Rapid carrier screening for Î²-thalassemia by single-step allele-specific PCR and detection | Quek D.L., Ng Y.-Y., Wang W., Tan A.S.C., Tang-Lim G.-I., Ng I.S.L., Law H.-Y., Chong S.S. | 10.1016/j.clinbiochem.2007.01.003 |
| The first compound heterozygosity for HKÎ±Î± allele and Southeast Asian deletion allele | Li Z., Cai S., Rong K., Song G., Li Y., Guo R. | 10.1016/j.clinbiochem.2006.10.030 |
| The thalassaemia syndromes | Birgens H., Ljung R. | 10.1080/00365510601046417 |
| Î±0-Thalassemia and related disorders in northeast Thailand: A molecular and hematological characterization | Sae-Ung N., Fucharoen G., Sanchaisuriya K., Fucharoen S. | 10.1159/000096857 |
| Effect of the maternal Î²E-globin gene on hematologic responses to iron supplementation during pregnancy | Sanchaisuriya K., Fucharoen S., Ratanasiri T., Sanchaisuriya P., Fucharoen G., Dietz E., Schelp F.P. | |
| The prevalence and molecular basis of hemoglobinopathies in Cambodia | Carnley B.P., Prior J.F., Gilbert A., Lim E., Devenish R., Sing H., Sarin E., Guhadasan R., Sullivan S.G., Wise C.A., Bittles A.H., Chan K., Wong M.-S., Chan V., Erber W.N. | 10.1080/03630260600868071 |
| Thalassemia among blood donors at the Hospital Universiti Sains Malaysia | Rosline H., Ahmed S.A., Al-Joudi F.S., Rapiaah M., Naing N.N., Adam N.A.M. | |
| Successful preimplantation genetic diagnosis for alpha- and beta-thalassemia in China | Deng J., Peng W.-L., Li J., Fang C., Liang X.-Y., Zeng Y.-H., Sun H.-Y., Zhou C.-Q., Zhuang G.-L. | 10.1002/pd.1549 |
| Sensitivity and specificity of dichlorophenol-indophenol precipitation test to screen for the hemoglobin E trait in pregnant women | Tongsong T., Sirichotiyakul S., Chaisen R., Wanapirak C. | 10.1016/j.ijgo.2006.05.013 |

| | | |
|---|---|---|
| Result of video-education on "genetic transmission in thalassemia" to thalassemic patients and their parents | Paholpak S., Jetsrisuparb A., Wiangnon S., Sangsahachat D., Padtawaro L. | |
| Experience in preimplantation genetic diagnosis for exclusion of homozygous Î±Â° thalassemia | Chan V., Ng E.H.Y., Yam I., Yeung W.S.B., Ho P.C., Chan T.K. | 10.1002/pd.1550 |
| Value of mean corpuscular volume and mean corpuscular haemoglobin in screening for Î²-thalassaemia trait | Mamtani M., Jawahirani A., Rughwani V., Das K., Kulkarni H. | 10.1159/000094688 |
| Red blood cell defects and malaria | Williams T.N. | 10.1016/j.molbiopara.2006.05.007 |
| Common Î±-thalassemia deletions in transfusion-dependent thalassemia patients in the Southeast Asia Region of Myanmar | Ne-Win, Harano K., Harano T., Thein-Thein-Myint, Rai-Mra, Aye-Aye-Myint, Shimono K., Okada S. | 10.1532/LH96.06017 |
| Molecular characterization of thalassemia intermedia in Indians | Panigrahi I., Agarwal S., Pradhan M., Choudhry D.R., Choudhry V.P., Saxena R. | |
| Enhanced oxidative cross-linking of hemoglobin E with spectrin and loss of erythrocyte membrane asymmetry in hemoglobin EÎ²-thalassemia | Datta P., Basu S., Chakravarty S.B., Chakravarty A., Banerjee D., Chandra S., Chakrabarti A. | 10.1016/j.bcmd.2006.06.001 |
| Bias-corrected diagnostic performance of the naked eye single tube red cell osmotic fragility test (NESTROFT): An effective screening tool for Î²-thalassemia | Mamtani M., Jawahirani A., Das K., Rughwani V., Kulkarni H. | 10.1080/10245330600915875 |
| Human red blood cell polymorphisms and malaria | Williams T.N. | 10.1016/j.mib.2006.06.009 |
| A successful strategy for Preimplantation Genetic Diagnosis of beta-thalassemia and simultaneous detection of Down's syndrome using multiplex fluorescent PCR | Piyamongkol W., Vutyavanich T., Piyamongkol S., Wells D., Kunaviktikul C., Tongsong T., Chaovisitsaree S., Saetung R., Sanguansermsri T. | |
| Molecular and hematological profiles of hemoglobin EE disease with different forms of Î±-thalassemia | Fucharoen G., Trithipsombat J., Sirithawee S., Yamsri S., Changtrakul Y., Sanchaisuriya K., Fucharoen S. | 10.1007/s00277-006-0093-5 |
| Three new Î±-thalassemia point mutations ascertained through newborn screening | Eng B., Patterson M., Walker L., Hoppe C., Azimi M., Lee H., Giordano P., Waye J. | 10.1080/03630260600642021 |
| Molecular characterization of the - SEA alpha thalassemia allele in Mexican patients with HbH disease | Nava M.P., Trejo J.M., Aguilar-Luna C., Barros-NÃºÃ±ez P., de la Luz ChÃ¡vez Ma., MagaÃ±a M.T., Perea J., Ibarra B. | |
| Thalassemia and hemoglobinopathies rather than iron deficiency are major causes of pregnancy-related anemia in northeast Thailand | Sanchaisuriya K., Fucharoen S., Ratanasiri T., Sanchaisuriya P., Fucharoen G., Dietz E., Schelp F.P. | 10.1016/j.bcmd.2006.04.006 |
| Gateways to clinical trials: July/August 2006 | BayÃ©s M., Rabasseda X., Prous J.R. | |
| Screening of concurrent Î±-thalassaemia 1 in Î²-thalassaemia carriers | Chong Y.M., Tan J.A.M.A., Zubaidah Z., Rahimah A., Kuldip K., George E. | |

| | | |
|---|---|---|
| Chicken egg yolk antibodies specific for the Î³ chain of human hemoglobin for diagnosis of thalassemia | Jintaridth P., Srisomsap C., Vichittumaros K., Kalpravidh R.W., Winichagoon P., Fucharoen S., Svasti M.R.J., Kasinrerk W. | 10.1532/IJH97.A20515 |
| An increase of the cardiothoracic ratio leads to a diagnosis of Bart's hydrops | Phupong V. | |
| Development of severe anemia during fever episodes in patients with hemoglobin E trait and hemoglobin H disease combinations | Jetsrisuparb A., Sanchaisuriya K., Fucharoen G., Fucharoen S., Wiangnon S., Jetsrisuparb C., Sirijirachai J., Chansoong K. | 10.1097/01.mph.0000212910.99394.e0 |
| Risk of a couple having a child with severe thalassemia syndrome, prevalence in lower northern Thailand | Wong P., Thanormrat P., Srithipayawan S., Jermnim N., Niyomthom S., Nimnuch N., Sanguansermsri T. | |
| Impaired interaction of Î±-haemoglobin-stabilising protein with Î±-globin termination mutant in a yeast two-hybrid system | Turbpaiboon C., Limjindaporn T., Wongwiwat W., U-Pratya Y., Siritanaratkul N., Yenchitsomanus P.-T., Jitrapakdee S., Wilairat P. | 10.1111/j.1365-2141.2005.05865.x |
| Hb Woodville, a rare Î±-globin variant, caused by codon 6 mutation of the Î±1 gene | Viprakasit V., Chinchang W., Chotimarat P. | 10.1111/j.0902-4441.2005.t01-1-EJH2334.x |
| Reassessment of a simple chemical method using DCIP for screening for haemoglobin E | Chapple L., Harris A., Phelan L., Bain B.J. | 10.1136/jcp.2005.027961 |
| Rapid detection of deletional Î±-thalassemia by an oligonucleotide microarray | Zesong L., Ruijun G., Wen Z. | 10.1002/ajh.20369 |
| Alpha-thalassemia major presenting in a term neonate without hydrops | Monaco S.E., Davis M., Huang A.-C., Bhagat G., Baergen R.N., Lorenz J.M., Chung W.K., Thaker H.M. | 10.1007/s10024-005-0063-2 |
| Heterogeneity in Î±-thalassemia interactions in Malays, Chinese and Indians in Malaysia | Wee Y.-C., Tan K.-L., Chow T.W.-P., Yap S.-F., Tan J.-A.M.A. | 10.1111/j.1447-0756.2005.00333.x |
| Common origin of a rare Î²-globin initiation codon mutation (ATG â†' AGG) in Asians | Viprakasit V., Chinchang W., Suwanthol L., Tanphaichitr V.S. | 10.1111/j.1365-2257.2005.00734.x |
| Molecular analysis of unknown Î²-globin gene mutations using polymerase chain reaction-single strand conformation polymorphism (PCR-SSCP) technique and its application in Thai families with Î²-thalassemias and Î²-globin variants | Chinchang W., Viprakasit V., Pung-Amritt P., Tanphaichitr V.S., Yenchitsomanus P.-T. | 10.1016/j.clinbiochem.2005.07.013 |
| Validation of osmotic fragility test and dichlorophenol indophenol precipitation test for screening of thalassemia and Hb E | Sangkitporn S., Sangkitporn S., Sangnoi A., Supangwiput O., Tanphaichitr V.S. | |
| Fetal haemoglobin augmentation in E/Î²0 thalassaemia: Clinical and haematological outcome | Singer S.T., Kuypers F.A., Olivieri N.F., Weatherall D.J., Mignacca R., Coates T.D., Davies S., Sweeters N., Vichinsky E.P. | 10.1111/j.1365-2141.2005.05768.x |

| | | |
|---|---|---|
| Molecular diagnosis of inherited disorders: Lessons from hemoglobinopathies | Patrinos G.P., Kollia P., Papadakis M.N. | 10.1002/humu.20225 |
| Haemoglobin E Î² thalassaemia in Sri Lanka | Premawardhena A., Fisher C.A., Olivieri N.F., De Silva S., Arambepola M., Perera W., O'Donnell A., Peto T.E.A., Viprakasit V., Merson L., Muraca G., Weatherall D.J. | 10.1016/S0140-6736(05)67396-5 |
| Compound heterozygote state for GÎ³ AÎ³(Î´Î²)Â°-thalassemia and hereditary persistence of fetal hemoglobin | Fucharoen S., Panyasai S., Surapot S., Fucharoen G., Sanchaisuriya K. | 10.1002/ajh.20426 |
| High frequency of Î±-thalassemia in the so ethnic group of South Laos | Sengchanh S., Sanguansermsri T., Horst D., Horst J., Flatz G. | 10.1159/000087892 |
| Neonatal anemia associated with Southeast Asian Ovalocytosis | Laosombat V., Dissaneevate S., Wongchanchailert M., Satayasevana B. | 10.1532/IJH97.A20505 |
| Heart failure in Î²-thalassemia syndromes: A decade of progress | Hahalis G., Alexopoulos D., Kremastinos D.T., Zoumbos N.C. | 10.1016/j.amjmed.2005.02.021 |
| Compound heterozygote states for Hb C/Hb Malay and Hb C/Hb E in pregnancy: A molecular and hematological analysis | Fucharoen S., Fucharoen G., Sanchaisuriya K., Surapot S. | 10.1016/j.bcmd.2005.05.004 |
| A rare association of Î±0-thalassemia (--SEA) and an initiation codon mutation (ATGâ†'A-G) of the Î±2 gene causes Hb H disease in Thailand | Viprakasit V., Chinchang W., Glomglao W., Tanphaichitr V.S. | 10.1081/HEM-200066339 |
| Clinical phenotypes and molecular diagnosis in a hitherto interaction of Hb E/Î² thalassemia syndrome (Î²E/ Î² -31, Aâ†'G) | Vathana N., Viprakasit V., Sanpakit K., Chinchang W., Veerakul G., Tanphaichitr V. | |
| High incidence of Î±-thalassemia, hemoglobin E, and glucose-6-phosphate dehydrogenase deficiency in populations of malaria-endemic southern Shan State, Myanmar | Than A.M., Harano T., Harano K., Myint A.A., Ogino T., Okada S. | 10.1532/IJH97.05028 |
| Hematological parameters and red blood cell indices in healthy Thai children: A revision for 2005 | Viprakasit V., Suwanthol L., Sangpraypan T., Glomglao W., Utto W., Veerakul G. | |
| Is there aberration in the secondary structure of globin chain in haemoglobin Suan-Dok disorder? | Wiwanitkit V. | |
| Sensitivity and specificity of mean corpuscular volume testing for screening for Î±-thalassemia-1 and Î²-thalassemia traits | Sirichotiyakul S., Maneerat J., Sa-Nguansermsri T., Dhananjayanonda P., Tongsong T. | 10.1111/j.1447-0756.2005.00280.x |
| Noninvasive prenatal diagnosis for hemoglobin Bart's hydrops fetalis | Winichagoon P., Sithongdee S., Kanokpongsakdi S., Tantisirin P., Bernini L.F., Fucharoen S. | 10.1532/IJH97.A20501 |
| Molecular genetic confirmatory testing from newborn screening samples for the common African-American, Asian Indian, Southeast Asian, and Chinese Î²-thalassemia mutations | Bhardwaj U., Zhang Y.-H., Lorey F., McCabe L.L., McCabe E.R.B. | 10.1002/ajh.20269 |

| | | |
|---|---|---|
| Prevalence and haematological parameters of thalassaemia in Lower Northern Thailand | Tangvarasittichai O., Sitthiworanan C., Dechgitvigrom W., Sanguansermsri T., Jeenapongsa R. | |
| Modeling for tertiary structure of globin chain in Hemoglobin Suan-Dok disorder | Wiwanitkit V. | 10.1080/10245330500067108 |
| Thalassaemia screening in pregnancy | Leung T.N., Lau T.K., Chung T.K.H. | |
| Thalassemia intermedia associated with complex interaction of Hb Beijing [Î±16(A14)Lysâ†'Asn] and Hb E [Î²26(B8)Gluâ†'Lys] with a deletional Î±-thalassemia-1 in a Thai family | Fucharoen S., Chunpanich S., Sanchaisuriya K., Fucharoen G., Kunyanone N. | 10.1081/HEM-200047085 |
| Association of Hb Q-Thailand with homozygous Hb E and heterozygous Hb Constant Spring in pregnancy | Sanchaisuriya K., Chunpanich S., Fucharoen S., Fucharoen G., Sanchaisuriya P., Changtrakun Y. | 10.1111/j.1600-0609.2004.00381.x |
| Alpha-thalassaemia and population health in Southeast Asia | Chui D.H.K. | 10.1080/03014460500075084 |
| Do alpha deletions influence hydroxyurea response in thalassemia intermedia? | Panigrahi I., Dixit A., Arora S., Kabra M., Mahapatra M., Choudhry V.P., Saxena R. | 10.1080/10245330400020439 |
| A reliable screening protocol for thalassemia and hemoglobinopathies in pregnancy: An alternative approach to electronic blood cell counting | Sanchaisuriya K., Fucharoen S., Fucharoen G., Ratanasiri T., Sanchaisuriya P., Changtrakul Y., Ukosanakarn U., Ussawaphark W., Schelp F.P. | 10.1309/FUF9-EVGQ-24V1-PKTP |
| Antioxidant deficit and enhanced susceptibility to oxidative damage in individuals with different forms of Î±-thalassaemia | Cheng M.-L., Ho H.-Y., Tseng H.-C., Lee C.-H., Shih L.-Y., Chiu D.T.-Y. | 10.1111/j.1365-2141.2004.05257.x |
| Study on molecular epidemiology of the Î±-thalassemias in Liuzhou City, Guangxi Autonomous Region, China | Cai R., Liu J., Wang L., Liang X., Xiao B., Su L., Zhou Y., Pan L. | 10.1081/HEM-200037743 |
| A new highly unstable Î± chain variant causing Î±+- thalassemia: Hb Zurich Albisrieden [Î±59(E8)Glyâ†'Arg (Î±2)] | Dutly F., Fehr J., Goede J.S., Morf M., Troxler H., Frischknecht H. | 10.1081/HEM-200037714 |
| Prevalence of Thalassemia in pregnant women at Maharaj Nakorn Chiang Mai Hospital | Wanapirak C., Muninthorn W., Sanguansermsri T., Dhananjayanonda P., Tongsong T. | |
| Acute haemolytic crisis in a Thai patient with homozygous haemoglobin Constants Spring (Hb CS/CS): A case report | Viprakasit V., Veerakul G., Sanpakit K., Pongtanakul B., Chinchang W., Tanphaichitr V.S. | 10.1179/027249304225019145 |
| Molecular and hematological characterization of hemoglobin hope/hemoglobin E and hemoglobin hope/Î±-Thalassemia 2 in Thai patients | Chunpanich S., Fucharoen S., Sachaisuriya K., Fucharoen G., Kam-Itsara K. | 10.1532/LH96.04050 |
| Endothelial injury and altered hemodynamics in thalassemia | Butthep P., Nuchprayoon I., Futrakul N. | |

| | | |
|---|---|---|
| Hematologic problems in immigrants from Southeast Asia | Jeng M.R., Vichinsky E. | 10.1016/j.hoc.2004.06.014 |
| Generation of transgenic mice expressing human hemoglobin E | Chen Q., Bouhassira E.E., Besse A., Suzuka S.M., Fabry M.E., Nagel R.L., Hirsch R.E. | 10.1016/j.bcmd.2004.07.006 |
| Î±2 codon 30 deletion (Î"GAG) causing non-deletional hemoglobin H disease in Guangxi province | Chen P., Li S.-Q., Wu H. | |
| Thalassemia in Sri Lanka: A progress report | Premawardhena A., de Silva S., Arambepola M., Olivieri N., Merson L., Muraco J., Allen A., Fisher C., Peto T., Vichinsky E., Weatherall D. | 10.1093/hmg/ddh250 |
| Laboratory diagnosis of a compound heterozygosity for Hb Hekinan [Î±27(B8) Glu-Asp] and a deletional Î±-thalassaemia 2 in Thailand | Chunpanich S., Ayukarn K., Sanchaisuriya K., Fucharoen G., Fucharoen S. | 10.1111/j.1365-2257.2004.00627.x |
| The 'hot spot' of Hb E [Î²26(B8)Gluâ†'Lys] in Southeast Asia: Î²-globin anomalies in the Lao Theung population of southern Laos | Flatz G., Sanguansermsri T., Sengchanh S., Horst D., Horst J. | 10.1081/HEM-120040334 |
| Erythrocyte osmotic fragility test for screening of alpha-thalassemia-1 and beta-thalassemia trait in pregnancy | Sirichotiyakul S., Tantipalakorn C., Sanguansermsri T., Wanapirak C., Tongsong T. | 10.1016/j.ijgo.2004.04.037 |
| Simultaneous detection of Î±-thalassemia and Î²-thalassemia by oligonucleotide microarray | Bang-Ce Y., Hongqiong L., Zhuanfong Z., Zhengsong L., Jianling G. | |
| Homogeneity of Î² 0-Thalassemia codon 17 (A â†'T) alleles in Northern Thailand using a direct DNA sequencing method | Sanguansermsri P., Shimbhu D., Wongvilairat R., Saetung R., Sanguansermsri T. | |
| Comparison of cost effectiveness between measuring the serum erythropoietin level and reticulocyte count for monitoring thalassemic patients: A note in Thai beta thalassemia/Hb E subjects | Wiwanitkit V. | 10.1080/10245330410001714257 |
| Hemoglobin Pakse: Presence on red blood cell membrane and detection by polymerase chain reaction - Single-strand conformational polymorphism | Turbpaiboon C., Siritantikorn A., Thongnoppakhun W., Bunditworapoom D., Limwongse C., Yenchitsomanus P.-T., Siritanaratkul N., Wilairat P. | 10.1532/IJH97.A20402 |
| MS analysis of single-nucleotide differences in circulating nucleic acids: Application to noninvasive prenatal diagnosis | Ding C., Chiu R.W.K., Lau T.K., Leung T.N., Chan L.C., Chan A.Y.Y., Charoenkwan P., Ng I.S.L., Law H.-Y., Ma E.S.K., Xu X., Wanapirak C., Sanguansermsri T., Liao C., Tan Jin Ai M.A., Chui D.H.K., Cantor C.R., Lo Y.M.D. | 10.1073/pnas.0403962101 |
| Overview of molecular deletion patterns of Î± 0-thalassemia in Thailand [3] | Wiwanitkit V. | 10.1532/LH96.03048 |
| Combined osmotic fragility and dichlorophenol-indolphenol test for hemoglobin disorder screening in Thai pregnant subjects: An appraisal [1] | Wiwanitkit V. | |
| High frequency of deletional Î±-thalassemia in Î²-thalassemia trait: Implications for genetic counseling | Panigrahi I., Ahmed Rafeeq P.H., Choudhry V.P., Saxena R. | 10.1002/ajh.20083 |

| | | |
|---|---|---|
| Prevalence of HFE mutations among the Thai population and correlation with iron loading in haemoglobin E disorder | Viprakasit V., Vathesathokit P., Chinchang W., Tachavanich K., Pung-Amritt P., Wimhurst V.L.C., Yenchitsomanus P.-T., Merryweather-Clarke A.T., Tanphaichitr V.S. | 10.1111/j.1600-0609.2004.00246.x |
| Two cases of compound heterozygosity for Hb Hekinan [Î±27(B8)Gluâ†' Asp (Î±1)] and Î±-thalassemia in Thailand | Ngiwsara L., Srisomsap C., Winichagoon P., Fucharoen S., Svasti J. | 10.1081/HEM-120035913 |
| Rapid detection of common Southeast Asian Î²-thalassemia mutations by nonisotopic multiplex PCR-SSCP analysis | Yip S.P., Fung L.F., Lo S.T.H. | 10.1089/10906570417 97301 |
| Molecular diagnosis of haemoglobin disorders | Clark B.E., Thein S.L. | 10.1111/j.1365-2257.2004.00607.x |
| Evaluation of alpha hemoglobin stabilizing protein (AHSP) as a genetic modifier in patients with Î² thalassemia | Viprakasit V., Tanphaichitr V.S., Chinchang W., Sangkla P., Weiss M.J., Higgs D.R. | 10.1182/blood-2003-11-3957 |
| A simplified screening strategy for thalassaemia and haemoglobin E in rural communities in south-east Asia | Fucharoen G., Sanchaisuriya K., Sae-Ung N., Dangwibul S., Fucharoen S. | |
| The diverse molecular basis and hematological features of Hb H and AEBart's diseases in northeast Thailand | Boonsa S., Sanchaisuriya K., Fucharoen G., Wiangnon S., Jetsrisuparb A., Fucharoen S. | 10.1159/000076523 |
| Thalassemia Screening among Royal Thai Army Medical Cadets | Nathalang O., Arnutti P., Nillakupt K. | |
| Osmotic fragility test for screening for thalassaemia in Thai pregnant subjects: A re-evaluation | Wiwanitkit V. | |
| Detection of Three Common, Deletional Î±-Thalassemia Determinants in Southern China by a Single-Tube Multiplex Polymerase Chain Reaction Method | Liu J.Z., Ou C.Y., Wang L.R., Xiao B., Huang L.J., Chen L.C. | 10.1081/HEM-120028885 |
| Unusual phenotype of hemoglobin EE with hemoglobin H disease: A pitfall in clinical diagnosis and genetic counseling | Viprakasit V., Tanphaichitr V.S. | 10.1016/j.jpeds.2003.12.021 |
| Co-inheritance of Hb Pak Num Po, a Novel Î±1 Gene Mutation, and Î±0 Thalassemia Associated with Transfusion-Dependent Hb H Disease | Viprakasit V., Tanphaichitr V.S., Veerakul G., Chinchang W., Petrarat S., Pung-Amritt P., Higgs D.R. | 10.1002/ajh.10479 |
| Red blood cell vesicles in thalassemia | Lamchiagdhase P., Rattanapong C., Lerdwana S., Nitipongwanich R., Noulsri E., Pattanapanyasat K. | |
| Prevention of Thalassemia: Experiences from Samui Island | Sangkitporn S., Pathompanichratana S., Songkharm B., Pathtong W., Chongkitivitya N., Sangkitporn S.K., Watanapocha U. | |
| Prevention and Control of Thalassemia at Saraburi Regional Hospital | Chareonkul P., Kraisin J. | |
| A thalassaemia array for Southeast Asia | Chan K., Wong M.S., Chan T.K., Chan V. | 10.1046/j.1365-2141.2003.04758.x |

| | | |
|---|---|---|
| Î²+45 G â†' C: A novel silent Î²-thalassaemia mutation, the first in the Kozak sequence | De Angioletti M., Lacerra G., Sabato V., Carestia C. | 10.1046/j.1365-2141.2003.04754.x |
| Prevalence and Clinical Significance of Hepatitis C Virus Infection in Thai Patients with Thalassemia | Wanachiwanawin W., Luengrojanakul P., Sirangkapracha P., Leowattana W., Fucharoen S. | |
| Carrier testing for autosomal-recessive disorders | Vallance H., Ford J. | 10.1080/10408360390247832 |
| Interaction of hemoglobin E and several forms of Î±-thalassemia in Cambodian families | Fucharoen S., Sanchaisuriya K., Fucharoen G., Panyasai S., Devenish R., Luy L. | |
| Rapid, simultaneous genotyping of five common Southeast Asian Î²-thalassemia mutations by multiplex minisequencing and denaturing HPLC | Yip S.P., Pun S.F., Leung K.H., Lee S.Y. | 10.1373/49.10.1656 |
| A Community-Based Thalassemia Prevention and Control Model in Northern Thailand | Pansatiankul B., Saisorn S. | |
| Pulmonary arterial hypertension in previously splenectomized patients with Î²-thalassemic disorders | Atichartakarn V., Likittanasombat K., Chuncharunee S., Chandanamattha P., Worapongpaiboon S., Angchaisuksiri P., Aryurachai K. | |
| Birth of healthy children after preimplantation diagnosis of Î²-thalassemia by whole-genome amplification | Jiao Z., Zhou C., Li J., Shu Y., Liang X., Zhang M., Zhuang G. | 10.1002/pd.659 |
| Testing the "Malaria Hypothesis"for the Case of Thailand: A Genetic Appraisal | Poolsuwan S. | |
| Monitoring of erythropoietin level in thalassaemic patients by radioimmunoassay | Wiwanitkit V., Paritpokee N., Bhokaisawan N., Boonchalermvichian C., Nuchprayoon I., Preechakas P. | |
| The global distribution of length polymorphisms of the promoters of the glucuronosyltransferase 1 gene (UGT1A1): Hematologic and evolutionary implications | Premawardhena A., Fisher C.A., Liu Y.T., Verma I.C., De Silva S., Arambepola M., Clegg J.B., Weatherall D.J. | 10.1016/S1079-9796(03)00071-8 |
| The simultaneous presence of Î±- and Î²-thalassaemia alleles: A pitfall of thalassaemia screening | Law H.Y., Chee M.K.L., Tan G.P., Ng I.S.L. | 10.1159/000069539 |
| Red cell indices and therapeutic trial of iron in diagnostic work-up for anemic Thai females | Nuchprayoon I., Sukthawee B., Nuchprayoon T. | |
| Analysis of Î²-thalassemia mutations in northern Thailand using an automated fluorescence DNA sequencing technique | Sirichotiyakul S., Saetung R., Sanguansermsri T. | 10.1081/HEM-120021541 |
| Molecular characterization of hereditary persistence of fetal hemoglobin in the Karen People of Thailand | Trachoo O., Sura T., Sakuntabhai A., Singhasivanon P., Krudsood S., Phimpraphi W., Krasaesub S., Chanjarunee S., Looareesuwan S. | 10.1081/HEM-120021542 |
| Effect of nutrition support on immunity in paediatric patients with beta-thalassaemia major | Tienboon P. | |

| | | |
|---|---|---|
| Complex interaction of Hb Hekinan [Î±27(B8) Glu-Asp] and Hb E [Î²26(B8) Glu-Lys] with a deletional Î±-thalassemia 1 in a Thai family | Fucharoen S., Changtrakun Y., Ratanasiri T., Fucharoen G., Sanchaisuriya K. | 10.1034/j.1600-0609.2003.00049.x |
| Multiple minisequencing screen for seven Southeast Asian nondeletional Î±-thalassemia mutations | Wang W., Ma E.S.K., Chan A.Y.Y., Chui D.H.K., Chong S.S. | 10.1373/49.5.800 |
| Prenatal detection of fetal hemoglobin E gene from maternal plasma | Fucharoen G., Tungwiwat W., Ratanasiri T., Sanchaisuriya K., Fucharoen S. | 10.1002/pd.607 |
| Insertion of common mutations into the human Î²-globin locus using GET Recombination and an EcoRI endonuclease counterselection cassette | Jamsai D., Nefedov M., Narayanan K., Orford M., Fucharoen S., Williamson R., Ioannou P.A. | 10.1016/S0168-1656(02)00287-0 |
| Hemin: A possible cause of oxidative stress in blood circulation of Î²-thalassemia/hemoglobin E disease | Phumala N., Porasuphatana S., Unchern S., Pootrakul P., Fucharoen S., Chantharaksri U. | 10.1080/1071576031000060607 |
| Multiplex minisequencing screen for common Southeast Asian and Indian Î²-thalassemia mutations | Wang W., Kham S.K.Y., Yeo G.-H., Quah T.-C., Chong S.S. | 10.1373/49.2.209 |
| Hemoglobin H disease: Not necessarily a benign disorder | Chui D.H.K., Fucharoen S., Chan V. | 10.1182/blood-2002-07-1975 |
| Molecular characterization of deletional forms of Î²-thalassemia in Taiwan | Peng C.-T., Liu S.-C., Chiou S.-S., Kuo P.-L., Shih M.-C., Chang J.-Y., Chang J.-G. | |
| Prenatal diagnosis of Alpha-thalassemia of Southeast Asian deletion with non-radioactive southern hybridization | Cheng P.-J., Chu D.-C., Lee C.-H., Chiueh H.-Y., Lin Y.-T., Soong Y.-K. | |
| Serum erythropoietin levels in pediatric patients with Î²-thalassemia/hemoglobin E | Paritpokee N., Wiwanitkit V., Bhokaisawan N., Boonchalermvichian C., Preechakas P. | |
| Use of multiplex PCR assay and hemoglobin H preparation in the diagnosis of Î±-thalassemia trait | Griswold D., Bergstrome Jones A., McNeely M., Poon A. | |
| Screening for the carriers of thalassemias and abnormal hemoglobins at the community level | Winichagoon P., Thitivichianlert A., Lebnak T., Piankijagum A., Fucharoen S. | |
| Hemoglobinopathies among five major ethnic groups in Karachi, Pakistan | Ghani R., Manji M.A., Ahmed N. | |
| Compound heterozygosity for Hb Korle-Bu (Î²73; Asp-Asn) and Hb E (Î²26; Glu-Lys) with a 3.7-kb deletional Î±-thalassemia in Thai patients | Changtrakun Y., Fucharoen S., Ayukarn K., Siriratmanawong N., Fucharoen G., Sanchaisuriya K. | 10.1007/s00277-002-0485-0 |
| Molecular characterization of thalassemia intermedia associated with HPFH-6/Î²-thalassemia and HPFH-6/Hb E in Thai patients | Fucharoen S., Fucharoen G., Sanchaisuriya K., Surapot S. | 10.1159/000064707 |
| Hb G-Makassar [Î²6(A3)Gluâ†'Ala; codon 6 (GAGâ†'GCG)]: Molecular characterization, clinical, and hematological effects | Viprakasit V., Wiriyasateinkul A., Sattayasevana B., Miles K.L., Laosombat V. | 10.1081/HEM-120015028 |

| | | |
|---|---|---|
| Molecular characterization of Hb D-Punjab [Î²121(GH4)Glu â†’ Gln] in Thailand | Fucharoen S., Changtrakun Y., Surapot S., Fucharoen G., Sanchaisuriya K. | 10.1081/HEM-120015030 |
| Hb PaksÃ© [(Î±2) codon 142 (TAAâ†’TAT or Termâ†’Tyr)] in Thai patients with EABart's disease and Hb H disease | Sanchaisuriya K., Fucharoen G., Fucharoen S. | 10.1081/HEM-120015026 |
| Molecular analysis of Î²-thalassemia in South Vietnam | Saovaros Svasti M.L., Hieu T.M., Munkongdee T., Winichagoon P., Van Be T., Van Binh T., Fucharoen S. | 10.1002/ajh.10193 |
| Molecular and hematological characterization of HPFH-6/indian deletion-inversion GÎ³(AÎ³Î´Î²)O -Thalassemia and GÎ³(AÎ³Î´Î²)O -thalassemia/HbE in Thai patients | Fucharoen S., Pengjam Y., Surapot S., Fucharoen G., Sanchaisuriya K. | 10.1002/ajh.10202 |
| Prenatal diagnosis of Î±-thalassemia-1 (SEA type) by chorionic villus sampling | Chanprapaph P., Wanapirak C., Sanguansermsri T., Tongsong T., Sirichotiyakul S. | |
| Evolutionary and historical aspects of the burden of malaria | Carter R., Mendis K.N. | 10.1128/CMR.15.4.564-594.2002 |
| Relationship between hypercoagulable state and erythrocyte phosphatidylserine exposure in splenectomized haemoglobin E/Î²-thalassaemic patients | Atichartakarn V., Angchaisuksiri P., Aryurachai K., Onpun S., Chuncharunee S., Thakkinstian A., Atamasirikul K. | 10.1046/j.1365-2141.2002.03711.x |
| A simplified screening for Î±-thalassemia 1 (SEA type) using a combination of a modified osmotic fragility test and a direct PCR on whole blood cell lysates | Panyasai S., Sringam P., Fucharoen G., Sanchaisuriya K., Fucharoen S. | 10.1159/000064746 |
| Routine screening of (--SEA) Î±-thalassemia deletion by an enzyme-linked immunosorbent assay for embryonic Î¶-globin chains | Ma S.K., Ma V., Chan A.Y.Y., Chan L.C., Chui D.H.K. | 10.1159/000063060 |
| Rapid confirmation of Southeast Asian and Filipino Î±-thalassemia genotypes from newborn screening specimens | Bhardwaj U., Zhang Y.-H., Blackburn W., McCabe L.L., McCabe E.R.B. | 10.1002/ajh.10182 |
| Thalassemia mutations and their clinical aspects in Japan. | Hattori Y. | |
| Psychosocial problems in children with thalassemia and their siblings | Louthrenoo O., Sittipreechacharn S., Thanarattanakorn P., Sanguansermsri T. | |
| Hepatitis G virus genotypes in a tertiary care centre in southern India | Abraham P., Hallett R., Radhakrishnan S., Raghuraman S., Sridharan G., Teo C.G. | 10.1016/S1386-6532(02)00008-2 |
| Î²-thalassemia in the Korean population | Park S.S., Lee Y.J., Kim J.Y., Joo S.I., Hattori Y., Ohba Y., Cho H.-I. | 10.1081/HEM-120005451 |
| Double heterozygosity for Hb Pyrgos [Î²83(EF7)Glyâ†’Asp] and Hb E [Î²26(B8)Gluâ†’Lys] found in association with Î±-thalassemia | Sawangareetrakul P., Svasti S., Yodsowon B., Winichagoon P., Srisomsap C., Svasti J., Fucharoen S. | 10.1081/HEM-120005459 |

| | | |
|---|---|---|
| Compound heterozygosity for Î±0-thalassemia (--THAI) and Hb constant spring causes severe Hb H disease | Viprakasit V., Tanphaichitr V.S. | 10.1081/HEM-120005453 |
| Serum concentrations of soluble transferrin receptor among paediatric patients with transfusion-dependant Î²-thalassaemia/haemoglobin E | Bhokaisawan N., Paritpokee N., Wiwanitkit V., Boonchalermvichian C., Nuchprayoon I. | 10.1179/000349802125001041 |
| Evaluation of a single-tube multiplex polymerase chain reaction screen for detection of common alpha-thalassemia genotypes in a clinical laboratory | Jones A.K.B., Poon A. | 10.1309/3VK2-UCJ1-5GBJ-QV8Q |
| Increased circulating activated endothelial cells, vascular endothelial growth factor, and tumor necrosis factor in thalassemia | Butthep P., Rummavas S., Wisedpanichkij R., Jindadamrongwech S., Fucharoen S., Bunyaratvej A. | 10.1002/ajh.10101 |
| Complex interactions of Î´Î² hybrid haemoglobin (Hb Lepore-Hollandia) Hb E (Î²26 gâ†'a) and Î±+ thalassaemia in a Thai family | Viprakasit V., Pung-Amritt P., Suwanthon L., Clark K., Tanphaichitr V.S. | 10.1034/j.1600-0609.2002.01637.x |
| PCR-based analysis of Î±-thalassemia in Southern Taiwan | Chen T.-P., Liu T.-C., Chang C.-S., Chang J.-G., Tsai H.-J., Lin S.-F. | 10.1007/BF02982041 |
| Association of homozygous Î±-thalassaemia of the Southeast Asian type with hypospadias: Still an intriguing enigma | Utsch B., Hansmann M., Albers N., Lentze M.J., Bidlingmaier F., Ludwig M. | 10.1159/000048023 |
| Clinical phenotypes and molecular characterization of Hb H-PaksÃ© disease | Viprakasit V., Tanphaichitr V.S., Pung-Amritt P., Petrarat S., Suwantol L., Fisher C., Higgs D.R. | |
| Application of automated HPLC in prenatal diagnosis of Thalassemia | Winichagoon P., Sriphanich R., Sae-Ngow B., Chowthaworn J., Tantisirin P., Kanokpongsakdi S., Fucharoen S., Wasi P. | |
| Circulating hematopoietic progenitor cells in a fetus with alpha thalassemia: Comparison with the cells circulating in normal and non-thalassemic anemia fetuses and implications for in utero transplantations | Migliaccio A.R., Migliaccio G., Di Baldassarre A., Eddleman K. | 10.1038/sj.bmt.1703599 |
| Red cell genetic abnormalities, Î²-globin gene haplotypes, and APOB polymorphism in the Great Andamanese, a primitive Negrito tribe of Andaman and Nicobar Islands, India | Murhekar K.M., Murhekar M.V., Mukherjee M.B., Gorakshakar A.C., Surve R., Wadia M., Phanasgaonkar S., Shridevi S., Colah Roshan B., Mohanty D. | |
| Quantitative polmerase chain reaction for the rapid prenatal diagnosis of homozygous Î±-thalassaemia (HB Barts hydrops fetalis) | Chan V., Yip B., Lam Y.H., Tse H.Y., Wong H.S., Chan T.K. | 10.1046/j.1365-2141.2001.03112.x |
| A multi-center study in order to further define the molecular basis of Î²-thalassemia in Thailand, Pakistan, Sri Lanka, Mauritius, Syria, and India, and to develop a simple molecular diagnostic strategy by amplification refractory mutation system-polymerase chain reaction | Old J.M., Khan S.N., Verma I., Fucharoen S., Kleanthous M., Ioannou P., Kotea N., Fisher C., Riazuddin S., Saxena R., Winichagoon P., Kyriacou K., Al-Quobaili F., Khan B. | 10.1081/HEM-100107877 |

| | | |
|---|---|---|
| Real-time quantitative PCR analysis for Î±-thalassemia-1 of Southeast Asian type deletion in Taiwan | Sun C.-F., Lee C.-H., Cheng S.-W., Lin M.-H., Wu T.-L., Tsao K.-C., Chiu D.T.Y., Liou J.-D., Chu D.-C. | 10.1034/j.1399-0004.2001.600409.x |
| Clinical and hematological features of Î²+-thalassemia (IVS-1 nt 5, G-C mutation) in Thai patients | Laosombat V., Wongchanchailert M., Sattayasevana B., Wiriyasateinkul A., Fucharoen S. | 10.1034/j.1600-0609.2001.t01-1-00431.x |
| Haemoglobin H disease due to (--SEA) Î±-globin gene deletion and Î±2-codon 30 (Î”GAG) mutation: A family study | Ma S.K., Chan A.Y.Y., Chiu E.K.W., Chan L.C. | |
| Molecular analysis of Î²-thalassemia in south vietnam | Hao L.T., Pissard S., Van P.H., Lacombe C., Hanh T.D., Goossens M., Kiet T.D. | 10.1081/HEM-100105223 |
| The hemoglobin O mutation in Indonesia: Distribution and phenotypic expression | Daud D., Harahap A., Setianingsih I., Nainggolan I., Tranggana S., Pakasi R., Marzuki S. | 10.1007/s100380170030 |
| Oligomerization and ligand binding in a homotetrameric hemoglobin: Two high-resolution crystal structures of hemoglobin Bart's (Î³4), a marker for Î±-thalassemia | Kidd R.D., Baker H.M., Mathews A.J., Brittain T., Baker E.N. | 10.1110/ps.11701 |
| Universal newborn screening for Hb H disease in California | Lorey F., Cunningham G., Vichinsky E.P., Lubin B.H., Witkowska H.E., Matsunaga A., Azimi M., Sherwin J., Eastman J., Farina F., Waye J.S., Chui D.H.K. | |
| Simultaneous PCR detection of Î²-thalassemia and Î±-thalassemia 1 (SEA type) in prenatal diagnosis of complex thalassemia syndrome | Siriratmanawong N., Fucharoen G., Sanchaisuriya K., Ratanasiri T., Fucharoen S. | 10.1016/S0009-9120(01)00250-8 |
| Atypical hemoglobin H disease in a Thai patient resulting from a combination of Î±-thalassemia 1 and hemoglobin constant spring with hemoglobin J Bangkok heterozygosity | Fucharoen S., Ayukarn K., Sanchaisuriya K., Fucharoen G. | 10.1034/j.1600-0609.2001.066005312.x |
| Linear growth in homozygous Î²-thalassemia and Î²-thalassemia/hemoglobin E patients under different treatment regimens | Viprakasit V., Mahasandana C., Suwantol L., Kankirawatana S., Suvatte V., Tanphaichitr V.S., Assteerawatt A., Veerakul G., Pung-Amritt P. | |
| Homozygous Î±-thalassemia associated with hypospadias: Sea-type deletion does not affect expression of the -14 gene and loss of the Î¸1-globin gene on 16p13.3 is compensated by its duplicate Î¸2 on chromosome 10 [3] | Utsch B., Albers N., Dame C., Bartmann P., Lentze M.J., Ludwig M. | 10.1002/ajmg.1344 |
| Genetic diseases of hemoglobin: Diagnostic methods for elucidating Î²-thalassemia mutations | Tuzmen S., Schechter A.N. | 10.1054/blre.2001.0147 |
| Genetic counseling for thalassemia in Thailand : Problems and solution | Dhamcharee V., Romyanan O., Ninlagarn T. | |

| | | |
|---|---|---|
| Clinical and hematologic features of Î²0-thalassemia (frameshift 41/42 mutation) in Thai patients | Laosombat V., Wongchanchailert M., Sattayasevana B., Wiriyasateinkul A., Fucharoen S. | |
| Effect of antioxidant flavonoids and a food mutagen on lymphocytes of a thalassemia patient without chelation therapy in the comet assay | Anderson D., Dhawan A., Yardley-Jones A., Ioannides C., Webb J. | 10.1002/1520-6866(2001)21:2<165::AID-TCM5>3.0.CO;2-Z |
| Clinical and hematological features of codon 17, A-T mutation of Î²-thalassemia in Thai patients | Laosombat V., Wongchanchailert M., Sattayasevana B., Wiriyasateinkul A., Fucharoen S. | 10.1034/j.1600-0609.2001.00305.x |
| Severe terminal transverse limb reduction defects in homozygous Southeast-Asian Î±-thalassaemia-1 | Chen C.-P. | |
| Effect of Î±-globin genotype on the pathophysiology of sickle cell disease | Ballas S.K. | 10.1080/15227950151073138 |
| Major hematologic diseases in the developing world- new aspects of diagnosis and management of thalassemia, malarial anemia, and acute leukemia. | Greenberg P.L., Gordeuk V., Issaragrisil S., Siritanaratkul N., Fucharoen S., Ribeiro R.C. | |
| A review on the origin and spread of deleterious mutants of the Î²-globin gene in Indian populations | Das S.K., Talukder G. | |
| Non-radioactive Southern hybridization for early diagnosis of Î±-thalassemia with Southeast Asian-type deletion in Taiwan | Chu D.-C., Lee C.-H., Lo M.-D., Cheng S.-W., Chen D.-P., Wu T.-L., Tsao K.-C., Chiu D.T.Y., Sun C.-F. | 10.1002/1096-8628(20001211)95:4<332::AID-AJMG7>3.0.CO;2-O |
| A reliable screening test to identify adult carriers of the (--SEA) alpha0-thalassemia deletion: Detection of embryonic zeta-globin chains by enzyme-linked immunosorbent assay | Lafferty J.D., Crowther M.A., Waye J.S., Chui D.H.K. | 10.1309/26G7-BQH4-93BV-UR0Q |
| Correlation between some discrimination functions and hemoglobin E | Ittarat W., Ongcharoenjai S., Rayatong O., Pirat N. | |
| Up-regulation of cell growth associated with an extra Y chromosome in a child with Î²-thalassemia major having undergone hematopoietic stem cell transplant | Tsang K.S., Li C.K., Chik K.W., Wong A.P.Y., Lau T.T., Li K., Pong H.N.H., Shing M.M.K., Yuen P.M.P. | 10.1097/00043426-200003000-00010 |
| Combined oral and parenteral iron chelation in beta thalassaemia major | Balveer K., Pyar K., Wonke B. | |
| Clinical manifestation of Î²-thalassemia/hemoglobin E disease | Fucharoen S., Ketvichit P., Pootrakul P., Siritanaratkul N., Piankijagum A., Wasi P. | |

| | | |
|---|---|---|
| Asian immigration and public health in California: Thalassemia in newborns in California | Lorey F. | |
| Infections in E-Î² thalassemia | Wanachiwanawin W. | |
| Outreach strategies for Southeast Asian communities: Experience, practice, and suggestions for approaching Southeast Asian immigrant and refugee communities to provide thalassemia education and trait testing | Choy J., Foote D., Bojanowski J., Yamashita R., Vichinsky E. | |
| Molecular analysis of Î²-thalassemia in Vietnam | Filon D., Oppenheim A., Rachmilewitz E.A., Kot R., Ba Truc D. | |
| Molecular analysis of Î±-thalassemia in Nepal: Correlation with malaria endemicity | Sakai Y., Kobayashi S., Shibata H., Furuumi H., Endo T., Fucharoen S., Hamano S., Acharya G.P., Kawasaki T., Fukumaki Y. | |
| Ten years' experience of antenatal mean corpuscular volume screening and prenatal diagnosis for thalassaemias in Hong Kong | Sin S.Y., Ghosh A., Tang L.C.H., Chan V. | |
| Differential diagnosis of Hb EE and Hb E-Î²(o)-thalassemia by protein and DNA analyses | Wong S.C., Aw T.C., Suri R., Wong C.K., Plaseska D., Efremov G.D. | |
| Spinal cord compression: A rareness in pregnant thalassemic woman | Phupong V., Uerpairojkij B., Limpongsanurak S. | |
| Infrared spectroscopic studies of nanoscale iron oxide deposits isolated from human thalassemic tissues | Chua-Anusorn W., Webb J. | 10.1016/S0162-0134(99)00233-0 |
| Prenatal control of severe thalassaemia: Chiang Mai Strategy | Tongsong T., Wanapirak C., Sirivatanapa P., Sanguansermsri T., Sirichotiyakul S., Piyamongkol W., Chanprapaph P. | 10.1002/(SICI)1097-0223(200003)20:3<229::AID-PD790>3.0.CO;2-3 |
| Clinical and hematologic aspects of hemoglobin E Î²-thalassemia | Fucharoen S., Winichagoon P. | 10.1097/00062752-200003000-00006 |
| Lymphocyte subsets and specific T-cell immune response in thalassemia | Pattanapanyasat K., Thepthai C., Lamchiagdhase P., Lerdwana S., Tachavanich K., Thanomsuk P., Wanachiwanawin W., Fucharoen S., Darden J.M. | 10.1002/(SICI)1097-0320(20000215)42:1<11::AID-CYTO3>3.0.CO;2-1 |
| PCR-based diagnosis of the Filipino (--(FIL)) and Thai (--(THAI)) Î±- thalassemia-1 deletions | Eng B., Patterson M., Borys S., Chui D.H.K., Waye J.S. | 10.1002/(SICI)1096-8652(200001)63:1<54::AID- |

| | | |
|---|---|---|
| | | AJH12>3.0.CO;2-B |
| Spectrum of Î² thalassemia mutations and their linkage to Î²-globin gene haplotypes in the Indo-Mauritians | Kotea N., Ramasawmy R., Lu C.Y., Fa N.S., Gerard N., Beesoon S., Ducrocq R., Surrun S.K., Nagel R.L., Krishnamoorthy R. | 10.1002/(SICI)1096-8652(200001)63:1<11::AID-AJH3>3.0.CO;2-D |
| Screening for (-(SEA)) Î±-globin gene deletion in Î²-thalassemia carriers and prevention of hydrops fetalis | Ma E.S.K., Chan A.Y.Y., Shau Yin Ha, Chan G.C.F., Yu Lung Lau, Li Chong Chan | |
| Thalassemia syndromes in Saudi Arabia: Meta-analysis of local studies | Al-Awamy B.H. | |
| Single-tube multiplex-PCR screen for common deletional determinants of Î±-thalassemia | Chong S.S., Boehm C.D., Higgs D.R., Cutting G.R. | |
| Simplified multiplex-PCR diagnosis of common Southeast Asian deletional determinants of Î±-Thalassemia | Chong S.S., Boehm C.D., Cutting G.R., Higgs D.R. | |
