# Supplementary material for: Prevalence of Alpha(α)-Thalassemia in Southeast Asia (2010–2020): A Meta-Analysis Involving 83,674 Subjects
Source: Int J Environ Res Public Health. 2020 Oct 9;17(20):7354. doi: 10.3390/ijerph17207354 (PMC7600098; doi:10.3390/ijerph17207354)
Supplement: Supplementary file 1 [file ijerph-17-07354-s001.zip › ijerph-909078-supplementary/Table S4. The SCOPUS literature (N = 152) included in the analysis..pdf]

Table S4: The SCOPUS literature (N = 152) included in the analysis.

Title	Authors	DOI
Molecular Epidemiology of Hemoglobinopathies in Cambodia	Munkongdee T., Tanakulmas J., Butthep P., Winichagoon P., Main B., Yiannakis M., George J., Devenish R., Fucharoen S., Svasti S.	10.3109/03630269.2016.1158723
Progress Toward the Control and Management of the Thalassemias	Fucharoen S., Weatherall D.J.	10.1016/j.hoc.2015.12.001
Hematological Practice in Hong Kong and China	Kwong Y.-L., Ha S.-Y., Chan V.	10.1016/j.hoc.2015.11.010
Prevalence of α-thalassaemia genotypes in pregnant women in northern Thailand	Pharephan S., Sirivatanapa P., Makonkawkeyoon S., Tuntiwechapikul W., Makonkawkeyoon L.	10.4103/0971-5916.182622
Thalassemia screening using different automated blood cell counters: Consideration of appropriate cutoff values	Chaitraiphop C., Sanchaisuriya K., Inthavong S., Fucharoen G., Sanchaisuriya P., Changtrakun Y., Fucharoen S.	10.7754/Clin.Lab.2015.150720
Characterization of Deletions of the HBA and HBB Loci by Array Comparative Genomic Hybridization	Sabath D.E., Bender M.A., Sankaran V.G., Vamos E., Kentsis A., Yi H.-S., Greisman H.A.	10.1016/j.jmoldx.2015.07.011
Prevalence of thalassemia carriers among the Lahu hill tribe population, Chiang Rai, Thailand	Apidechkul T.	10.5372/1905-7415.0904.423
Molecular spectrum of α-globin gene mutations in the Aegean region of Turkey: first observation of three α-globin gene mutations in the Turkish population	Onay H., Aykut A., Karaca E., Durmaz A., Solmaz A.E., Çoğulu Ö., Aydınok Y., Vergin C., Özkınay F.	10.1007/s12185-015-1796-y
Discrimination of various thalassemia syndromes and iron deficiency and utilization of reticulocyte measurements in monitoring response to iron therapy	Winichagoon P., Kumbunlue R., Sirankapracha P., Boonmongkol P., Fucharoen S.	10.1016/j.bcmd.2015.01.010
The spectrum of α-thalassemia mutations in Kermanshah Province, West Iran	Alibakhshi R., Mehrabi M., Omidniakan L., Shafieenia S.	10.3109/03630269.2015.1070732
The prevalence and spectrum of α-Thalassemia in Guizhou Province of South China	Huang S.-W., Xu Y., Liu X.-M., Zhou M., Li G.-F., An B.-Q., Su L., Wu X., Lin J.	10.3109/03630269.2015.1041037
Sonographic markers of fetal α-thalassemia major	Li X., Zhou Q., Zhang M., Tian X., Zhao Y.	10.7863/ultra.34.2.197
Interaction of Hb Grey Lynn (Vientiane) [α91(FG3)Leu>Phe (α1)] with Hb E [β26(B8) Glu>Lys] and α+-thalassemia:	Singha K., Fucharoen G., Fucharoen S.	10.7754/Clin.Lab.2014.141112

Molecular and hematological analysis		
Validation of the immunochromatographic strip for α-thalassemia screening: A multicenter study	Winichagoon P., Kumpan P., Holmes P., Finlayson J., Newbound C., Kabral A., Li B., Nuinoon M., Fawcett T., Tayapiwatana C., Kasinrerk W., Fucharoen S.	10.1016/j.trsl.2014.10.013
Detection of deletion α+-thalassemia mutation [-α (3.7), -α (4.2)] by quantitative PCR assay	Seeratanachot T., Shimbhu D., Charoenkwan P., Sanguansermsri T.	
Prevalence of Alpha thalassemia in microcytic anemia: A tertiary care experience from north India	Sharma M., Pandey S., Ranjan R., Seth T., Saxena R.	10.4084/MJHID.2015.004
Increasing prevalence of thalassemia in America: Implications for primary care	Sayani F.A., Kwiatkowski J.L.	10.3109/07853890.2015.1091942
Known and new hemoglobin A2 variants in Thailand and implication for β-thalassemia screening	Panyasai S., Fucharoen G., Fucharoen S.	10.1016/j.cca.2014.09.003
Coexistence of malaria and thalassemia in malaria endemic areas of Thailand	Kuesap J., Chaijaroenkul W., Rungsihirunrat K., Pongjantharasatien K., Na-Bangchang K.	10.3347/kjp.2015.53.3.265
The effect of thalassemia on erythrocyte reference intervals in a representative han Chinese adult population	Xu J.-H., Hao X.-K., Mu R.-Q., Pan B.-S., Zhang J., Peng M.-T., Wang L.-L., Huang X.-Z., Ma Y.-Y., Zhao M., Guo W., Qiao R., Chen W.-X., Jiang H., Shang H.	10.7754/Clin.Lab.2014.140905
Medium-based noninvasive preimplantation genetic diagnosis for human α-thalassemias-SEA	Wu H., Ding C., Shen X., Wang J., Li R., Cai B., Xu Y., Zhong Y., Zhou C.	10.1097/MD.0000000000000669
Diagnostic pitfalls of less well recognized HbH disease	Farashi S., Najmabadi H.	10.1016/j.bcmd.2015.08.003
Population screening and prevention strategies for thalassemias and other hemoglobinopathies of Eastern India: Experience of 18,166 cases	Chatterjee T., Chakravarty A., Chakravarty S.	10.3109/03630269.2015.1068799
Risk factors for malaria and adverse birth outcomes in a prospective cohort of pregnant women resident in a high malaria transmission area of Papua New Guinea	Stanisic D.I., Moore K.A., Baiwog F., Ura A., Clapham C., King C.L., Siba P.M., Beeson J.G., Mueller I., Fowkes F.J., Rogerson S.J.	10.1093/trstmh/trv019
Mutation screening for thalassaemia in the Jino ethnic minority population of Yunnan Province, Southwest China	Wang S., Zhang R., Xiang G., Li Y., Hou X., Jiang F., Jiang F., Hu C., Jia W.	10.1136/bmjopen-2015-010047

Identification of Hb Constant Spring (HBA2: C.427T > C) by an Automated High Performance Liquid Chromatography Method	Wisedpanichkij R., Jindadamrongwech S., Butthep P.	10.3109/03630269.2015.1027828
Hemoglobin e and glucose-6-phosphate dehydrogenase deficiency and plasmodium falciparum malaria in the chittagong hill districts of Bangladesh	Shannon K.L., Ahmed S., Rahman H., Prue C.S., Khyang J., Ram M., Haq M.Z., Chowdhury A., Akter J., Glass G.E., Shields T., Nyunt M.M., Khan W.A., Sack D.A., Sullivan D.J.	10.4269/ajtmh.14-0623
Genetic hemoglobin disorders rather than iron deficiency are a major predictor of hemoglobin concentration in women of reproductive age in rural prey veng, Cambodia	Karakochuk C.D., Whitfield K.C., Barr S.I., Lamers Y., Devlin A.M., Vercauteren S.M., Kroeun H., Talukder A., McLean J., Green T.J.	10.3945/jn.114.198945
Genotyping of α-thalassemias by the colorimetric nanogold probes	Chomean S., Wangmaung N., Sritongkham P., Promptmas C., Ittarat W.	10.1016/j.cca.2014.07.033
A large cohort of hemoglobin variants in thailand: Molecular epidemiological study and diagnostic consideration	Srivorakun H., Singha K., Fucharoen G., Sanchaisuriya K., Fucharoen S.	10.1371/journal.pone.0108365
Molecular epidemiological characterization and health burden of thalassemia in Jiangxi Province, P. R. China	Lin M., Zhong T.-Y., Chen Y.-G., Wang J.-Z., Wu J.-R., Lin F., Tong X., Yang H.-T., Hu X.-M., Hu R., Zhan X.-F., Yang H., Luo Z.-Y., Li W.-Y., Yang L.-Y.	10.1371/journal.pone.0101505
Molecular characterization of α- and β-thalassaemia among Malay patients	Mohd Yatim N.F., Abd. Rahim M., Menon K., Al-Hassan F.M., Ahmad R., Manocha A.B., Saleem M., Yahaya B.H.	10.3390/ijms15058835
A simple and highly sensitive elisa for screening of the α-thalassemia-1 southeast asian-type deletion	Pata S., Khummuang S., Pornprasert S., Tatu T., Kasinrerk W.	10.1080/15321819.2013.838963
The prevalence and molecular spectrum of α- and β-globin gene mutations in 14,332 families of Guangdong Province, China	Yin A., Li B., Luo M., Xu L., Wu L., Zhang L., Ma Y., Chen T., Gao S., Liang J., Guo H., Qin D., Wang J., Yuan T., Wang Y., Huang W.-W., He W.-F., Zhang Y., Liu C., Xia S., Chen Q., Zhao Q., Zhang X.	10.1371/journal.pone.0089855
Interaction of hemoglobin Grey Lynn (Vientiane) with a non-deletional α+-thalassemia in an adult Thai proband	Singha K., Fucharoen G., Fucharoen S.	10.11613/bm.2014.019
A newly modified hemoglobin h inclusion test as a secondary screening for α0-thalassemia in southeast asian populations	Fucharoen G., Yooyen K., Chaibunruang A., Fucharoen S.	10.1159/000355187
ARKRAY ADAMS A1c HA-8180T analyzer for diagnosis of	Kunwandee J., Srivorakun H., Fucharoen G., Sanchaisuriya K., Fucharoen S.	10.1309/LMmh649poetqrexl

thalassemia and hemoglobinopathies common in southeast Asia		
The correlation of α-globin gene mutations and the XmnI polymorphism with clinical severity of Hb E/β-thalassemia	Charoenkwan P., Teerachaimahit P., Sanguansermsri T.	10.3109/03630269.2014.952744
Hb Cibeles [α2 CD25(B6) (Gly → Asp)]: a novel alpha chain variant causing alpha-thalassemia	de la Fuente-Gonzalo F., Nieto J.M., Vinuesa L., Sevilla J., Díaz-Mediavilla J., Villegas A., González F.A., Ropero P.	10.1007/s12185-014-1663-2
Phenotypic expression of Hb F in common high Hb F determinants in Thailand: Roles of α-thalassemia, 5' δ-globin BCL11A binding region and 3' β-globin enhancer	Prakobkaew N., Fucharoen S., Fuchareon G., Siriratmanawong N.	10.1111/ejh.12201
Prevalence of mutations of alpha Globin gene in suspected alpha carrier couples, Babolsar, 2006-2011	Valizadeh F., Deylami A.	
Five hemoglobin variants in a double heterozygote for α- and β-globin chain defects	Singha K., Fucharoen G., Fucharoen S.	10.1159/000353123
Development of bead-based suspension array technology for the diagnosis of thalassemia	Yin A., Zhang L., Luo M., He T., Zhang Y., Liu C., Du L., Qin D., Liang J., Li B., Wu L., Ma Y., Guo H., Wang J., Yuan T., Wang Y., Zhang Y., Zhang X.	10.1002/ajh.23830
Molecular characteristic of alpha thalassaemia among patients diagnosed in UKM medical centre	Azma R.Z., Ainoon O., Hafiza A., Azlin I., Noor Farisah A.R., Nor Hidayati S., Noor Hamidah H.	
Diagnostic utility of isoelectric focusing and high performance liquid chromatography in neonatal cord blood screening for thalassemia and non-sickling hemoglobinopathies	Uaprasert N., Settapiboon R., Amornsiriwat S., Sarnthammakul P., Thanapat T., Rojnuckarin P., Sutcharitchan P.	10.1016/j.cca.2013.09.041
Measurement of HbA2 by capillary electrophoresis for diagnosing β-thalassemia/HbE disease in patients with low hbf	Prasing W., Pornprasert S.	10.1309/LMGD96HES3DZRBZM
Alpha thalassaemia in tribal communities of coastal maharashtra, India	Deo M.G., Pawar P.V.	
The α-Thalassemias	Piel F.B., Weatherall D.J.	10.1056/NEJMra1404415
Thalassemia and hemoglobin e in southern Thai blood donors	Nuinoon M., Kruachan K., Sengking W., Horpet D., Sungyuan U.	10.1155/2014/932306
State of the art and new developments in molecular	Harteveld C.L.	10.1111/ijlh.12108

diagnostics for hemoglobinopathies in multiethnic societies		
Variability of hemoglobin F expression in hemoglobin EE disease: Hematological and molecular analysis	Pakdee N., Yamsri S., Fucharoen G., Sanchaisuriya K., Pissard S., Fucharoen S.	10.1016/j.bcmd.2014.02.005
Clinical Features and Molecular Analysis of Hb H Disease in Taiwan	Chao Y.-H., Wu K.-H., Wu H.-P., Liu S.-C., Peng C.-T., Lee M.-S.	10.1155/2014/271070
Pattern of hemoglobinopathies and thalassemias in upper Assam region of North Eastern India: High performance liquid chromatography studies in 9000 patients	Baruah M.K., Saikia M., Baruah A.	10.4103/0377-4929.134680
Hemoglobin Constant Spring is markedly high in women of an ethnic minority group in Vietnam: A community-based survey and hematologic features	Nguyen V.H., Sanchaisuriya K., Wongprachum K., Nguyen M.D., Phan T.T.H., Vo V.T., Sanchaisuriya P., Fucharoen S., Schelp F.P.	10.1016/j.bcmd.2013.12.002
Routine screening for a-thalassaemia using an immunochromatographic strip assay for haemoglobin bart's	Prayalaw P., Fucharoen G., Fucharoen S.	10.1177/0969141314538611
Prevention and control of Hb Bart's Disease in Guangxi Zhuang Autonomous Region, China	He S., Zhang Q., Li D., Chen S., Tang Y., Chen Q., Zheng C.	10.1016/j.ejogrb.2014.03.034
Effect of Swiss-type heterocellular HPFH from XmnI-Gγ and HBBP1 polymorphisms on HbF, HbE, MCV and MCH levels in Thai HbE carriers	Kerdpoo S., Limweeraprajak E., Tatu T.	10.1007/s12185-014-1516-z
An evolutionary perspective of how infection drives human genome diversity: The case of malaria	Mangano V.D., Modiano D.	10.1016/j.coi.2014.06.004
Thalassemias-pathophysiology, molecular basics and diagnostics [Talasemie - patofizjologia, podstawy molekularne i diagnostyka]	Turowski P., Uhrynowska M., Brojer E.	
Isothermal strand-displacement polymerase reaction for visual detection of the southeast Asian-type deletion of α-thalassemia	Yu L., Wu W., Lie P., Liu Y., Zeng L.	10.1016/j.jmoldx.2013.06.003

Diagnosis and prevention of thalassemia	Ip H.-W., So C.-C.	10.3109/10408363.2013.847236
Burden of anemia in relation to thalassemia and iron deficiency among vietnamese pregnant women	Siridamrongvattana S., Van Hoa N., Sanchaisuriya K., Dung N., Hoa P.T.T., Sanchaisuriya P., Fucharoen G., Fucharoen S., Schelp F.P.	10.1159/000351168
Hematological characterization of compound heterozygous hemoglobin Hope/E patients with and without α-Thalassemia-1 SEA type deletion	Pornprasert S.	10.7754/Clin.Lab.2013.130139
Fetal red blood cell parameters in thalassemia and hemoglobinopathies	Karnpean R., Fucharoen G., Fucharoen S., Ratanasiri T.	10.1159/000354343
Strategies for basic laboratory diagnostics of the hemoglobinopathies in multi-ethnic societies: Interpretation of results and pitfalls	Giordano P.C.	10.1111/ijlh.12037
Distribution of alpha thalassaemia gene variants in diverse ethnic populations in Malaysia: Data from the institute for medical Research	Ahmad R., Saleem M., Aloysious N.S., Yelumalai P., Mohamed N., Hassan S.	10.3390/ijms140918599
Prenatal control of nondeletional α-thalassemia: First experience in mainland china	Li J., Li R., Zhou J.-Y., Xie X.-M., Liao C., Li D.-Z.	10.1002/pd.4149
Discussion	Agarwal A.M., Nussenzveig R.H., Hoke C., Lorey T.S., Greene D.N.	10.1309/AJCPF4UIJKH3EOBY
Problems in determining thalassemia carrier status in a program for prevention and control of severe thalassemia syndromes: A lesson from Thailand	Viprakasit V., Limwongse C., Sukpanichnant S., Ruangvutilert P., Kanjanakorn C., Glomglao W., Sirikong M., Utto W., Tanphaichitr V.S.	10.1515/cclm-2013-0098
Molecular and hematological studies in a large cohort of α0-thalassemia in northeast Thailand: Data from a single referral center	Chaibunruang A., Prommetta S., Yamsri S., Fucharoen G., Sae-Ung N., Sanchaisuriya K., Fucharoen S.	10.1016/j.bcmd.2013.04.003
Evaluation and comparison of soluble transferrin receptor in thalassemia carriers and iron deficient patients	Khatami S., Dehnabeh S.R., Mostafavi E., Kamalzadeh N., Yaghmaei P., Saeedi P., Shariat F., Bagheriyan H., Zeinali S., Akbari M.T.	10.3109/03630269.2013.780248
Low cost biosensor-based molecular differential diagnosis of α-thalassemia (Southeast Asia deletion)	Wangmaung N., Promptmas C., Chomean S., Sanchomphu C., Ittarat W.	10.1515/cclm-2012-0732

Diagnostic testing for α-globin gene disorders in a heterogeneous North American population	Waye J.S., Eng B.	10.1111/ijlh.12066
Prenatal and newborn screening for hemoglobinopathies	Hoppe C.C.	10.1111/ijlh.12076
Treating iron overload in patients with non-transfusion-dependent thalassemia	Taher A.T., Viprakasit V., Musallam K.M., Cappellini M.D.	10.1002/ajh.23405
Effectiveness of the model for prenatal control of severe thalassemia	Tongsong T., Charoenkwan P., Sirivatanapa P., Wanapirak C., Piyamongkol W., Sirichotiyakul S., Srisupundit K., Tongprasert F., Luewan S., Ratanasiri T., Komwilaisak R., Saksiriwuttho P., Vuthiwong C., Punpuckdeekoon P., Panichkul P., Rueangchainikhom W., Choowong J., Orungrote N., Sarapak S., Kovavisarach E., Jaruyawongs P., Tansathit T., Phadungkiatwattana P., Rujiwetpongstorn J., Kor-Anantakul O., Suwanrath C., Hanprasertpong T., Pranpanus S.	10.1002/pd.4095
Detection of coinherited Hb H-constant spring/Paksé disease and Hb e by capillary electrophoresis and high performance liquid chromatography	Pornprasert S., Waneesorn J.	10.3109/03630269.2012.752744
Evidence of recent natural selection on the Southeast Asian deletion (- SEA) causing α-thalassemia in South China	Qiu Q.-W., Wu D.-D., Yu L.-H., Yan T.-Z., Zhang W., Li Z.-T., Liu Y.-H., Zhang Y.-P., Xu X.-M.	10.1186/1471-2148-13-63
Diagnosis of α-thalassemia-1 southeast Asian type deletion and fetal gender by single-tube multiplex real-time PCR	Srithep S., Kongthai K., Pornprasert S.	10.7754/Clin.Lab.2012.120217
Alpha-thalassemia mental retardation syndrome: A case report of two affected siblings	Latiff Z.A., Omar S.A.S., Lau D., Wong S.W., Ong L.C., Aziz D.A., Akmal S.N.	10.3233/JPN-120598
Association of Hb thailand [α56(E5)Lys→Thr] and Hb phnom penh [α117(GH5)-Ile-α118(H1)] with α;0-Thalassemia: Molecular and hematological features and differential diagnosis	Singha K., Srivorakun H., Fucharoen G., Changtrakul Y., Komwilaisak P., Jetsrisuparb A., Puangplruk R., Fucharoen S.	10.3109/03630269.2012.747964
Identification of hemoglobin variants in samples received for glycated hemoglobin testing	Saw S., Loh T.P., Yin C., Sethi S.K.	10.1016/j.cca.2012.10.043
Automated capillary electrophoresis in the screening for hemoglobinopathies	Cotton F., Wolff F., Gulbis B.	10.1007/978-1-62703-296-4_16

Distribution of alpha thalassaemia in 16 year old Malaysian students in Penang, Melaka and Sabah	Ahmad R., Sabrina N., Bahrin S., Hassan R., Yelumalai P., Hidayat N., Hassan S., Zakaria Z.	
Artemisinin-resistant Plasmodium falciparum in Pursat province, western Cambodia: A parasite clearance rate study	Amaratunga C., Sreng S., Suon S., Phelps E.S., Stepniewska K., Lim P., Zhou C., Mao S., Anderson J.M., Lindegardh N., Jiang H., Song J., Su X.-Z., White N.J., Dondorp A.M., Anderson T.J.C., Fay M.P., Mu J., Duong S., Fairhurst R.M.	10.1016/S1473-3099(12)70181-0
Resistance to malaria in humans: The impact of strong, recent selection	Hedrick P.W.	10.1186/1475-2875-11-349
Prevalence of iron deficiency anaemia and thalassaemia trait among undergraduate medical students	Azma R.Z., Ainoon O., Azlin I., Hamenuddin H., Hadi N.A., Tatt W.K., Syazana I.N., Asmaliza A.M., Das S., Hamidah N.H.	
Development of a fluorescence immunochromatographic assay for the detection of zeta globin in the blood of (--SEA) α-thalassemia carriers	Wen L., Zhu P., Liu Y., Pan Q., Qu Y., Xu X., Li X., Fu N.	10.1016/j.bcmd.2012.05.011
A spurious haemoglobin A1c result associated with double heterozygote for haemoglobin Raleigh (β1[NA1]Val → Ala) and α+-thalassaemia	Singha K., Fucharoen G., Chaibunruang A., Netnee P., Fucharoen S.	10.1258/acb.2012.011234
The contribution of extramedullary hematopoiesis to hepatomegaly in anemic hydrops fetalis: A study in alpha-thalassemia hydrops fetalis	Taweevisit M., Thorner P.S.	10.2350/11-12-1126-OA.1
The spectrum of α-and β-thalassemia mutations in Yunnan Province of southwestern China	Zhang J., Zhu B.-S., He J., Zeng X.-H., Su J., Xu X.-H., Li S.-Y., Chen H., Zhang Y.-H.	10.3109/03630269.2012.717327
Unmasking Hb Paksé (codon 142, TAA>TAT, α2) and its combinations in patients also carrying Hb constant spring (codon 142, TAA>CAA, α2) in Northern Thailand	Pornprasert S., Panyasai S., Treesuwan K.	10.3109/03630269.2012.709896
Microsatellite markers within the α-globin gene cluster for robust preimplantation genetic diagnosis of severe α-Thalassemia syndromes in mediterranean populations	Destouni A., Christopoulos G., Vrettou C., Kakourou G., Kleanthous M., Traeger-Synodinos J., Kanavakis E.	10.3109/03630269.2012.666512
Screening for co-existence of α-thalassemia in β-thalassemia	Tatu T., Khuntarak S., Suwannasin S., Kiewkarnkha T., Khamrin S., Kasinrerk W.	10.1007/s12185-012-1039-4

and in HbE heterozygotes via an enzyme-linked immunosorbent assay for Hb Bart's and embryonic ζ-globin chain		
Quantification of hemoglobin Constant Spring in heterozygote and homozygote by a capillary electrophoresis method	Pornprasert S., Panyasai S., Waneesorn J., Kongthai K., Singboottra P.	10.1111/j.1751-553X.2011.01371.x
Genetic hemoglobin disorders, infection, and deficiencies of iron and vitamin A determine anemia in young Cambodian children	George J., Yiannakis M., Main B., Devenish R., Anderson C., An U.S., Williams S.M., Gibson R.S.	10.3945/jn.111.148189
Classification of complete blood count and haemoglobin typing data by a C4.5 decision tree, a naïve Bayes classifier and a multilayer perceptron for thalassaemia screening	Setsirichok D., Piroonratana T., Wongseree W., Usavanarong T., Paulkhaolarn N., Kanjanakorn C., Sirikong M., Limwongse C., Chaiyaratana N.	10.1016/j.bspc.2011.03.007
Limit of sensitivity for melting curve screening for α-thalassemia	Sriiam S., Leecharoenkiat A., Lithanatudom P., Munkongdee T., Svasti S., Smith D.R.	10.5372/1905-7415.0601.134
Micromapping of thalassemia and hemoglobinopathies in diferent regions of Northeast Thailand and Vientaine, Laos People's Democratic Republic	Tritipsombut J., Sanchaisuriya K., Phollarp P., Bouakhasith D., Sanchaisuriya P., Fucharoen G., Fucharoen S., Schelp F.P.	10.3109/03630269.2011.637149
Phenotypic expression of hemoglobins A2, E and F in various hemoglobin E related disorders	Sae-ung N., Srivorakun H., Fucharoen G., Yamsri S., Sanchaisuriya K., Fucharoen S.	10.1016/j.bcmd.2011.09.008
The prevalence of α thalassemia in South Western Maharashtra	Momin A.A., Bankar M.P., Bhoite G.M.	
New updating into hemoglobinopathies	Fucharoen S., Winichagoon P.	10.1111/j.1751-553X.2012.01446.x
Detection of common deletions and mutations causing α-thalassemia in Southeast Asians and Southern Chinese with denaturing high performance liquid chromatography	Jia X.-Y., Wei X.-J., Tang N., Wang L.-R., Han H., Zheng M.-L., Cai R., Xiao B., Liu J.-Z.	10.3760/cma.j.issn.1003-9406.2011.06.016
Alpha thalassemia major - A hematological prelude to an oncological problem	Pant S.	10.1016/j.mehy.2011.07.037
Haemoglobinopathies in Southeast Asia	Fucharoen S., Winichagoon P.	
A rapid detection for α-thalassemia by PCR combined with dissociation curve analysis	Jia X., Liu J., Wang L., Yao L., Tang N., Cai R., Xiao B.	10.1016/j.yexmp.2011.06.013

Hb E/beta-thalassaemia: A common & clinically diverse disorder	Olivieri N.F., Pakbaz Z., Vichinsky E.	
How I treat thalassemia	Rachmilewitz E.A., Giardina P.J.	10.1182/blood-2010-08-300335
Preimplantation genetic diagnosis for α-and β-double thalassemia	Shen X., Xu Y., Zhong Y., Zhou C., Zeng Y., Zhuang G., Ding C., Li T.	10.1007/s10815-011-9598-5
Molecular analysis of globin gene expression in different thalassaemia disorders: Individual variation of βE pre-mRNA splicing determine disease severity	Tubsuwan A., Munkongdee T., Jearawiriyapaisarn N., Boonchoy C., Winichagoon P., Fucharoen S., Svasti S.	10.1111/j.1365-2141.2011.08770.x
Genotype and phenotype characterizations in a large cohort of β-thalassemia heterozygote with different forms of β-thalassemia in northeast Thailand	Yamsri S., Sanchaisuriya K., Fucharoen G., Sae-ung N., Fucharoen S.	10.1016/j.bcmd.2011.05.003
Comparison between capillary electrophoresis and high performance liquid chromatography for detection and quantification of Hb constant spring [Hb CS; α142, Term→Gln (TAA>CAA in α2)]	Waneesorn J., Panyasai S., Kongthai K., Singboottra P., Pornprasert S.	10.3109/03630269.2011.588140
Detection of α-thalassemia-1 Southeast Asian and Thai type deletions and β-thalassemia 3.5-kb deletion by single-tube multiplex real-time PCR with SYBR green 1 and high-resolution melting analysis	Pornprasert S., Wiengkum T., Srithep S., Chainoi I., Singboottra P., Wongwiwatthananukit S.	10.3343/kjlm.2011.31.3.138
Evaluation of the URIT-2900 Automated Hematology Analyzer for screening of thalassemia and hemoglobinopathies in Southeast Asian populations	Karnpean R., Pansuwan A., Fucharoen G., Fucharoen S.	10.1016/j.clinbiochem.2011.04.009
Complex interaction of hemoglobin (Hb) Nakhon Ratchasima [α63(E12)Ala→Val], a novel α2-globin chain variant with Hb E [β26(B8)Glu→Lys] and a deletional α +-thalassemia	Srivorakun H., Fucharoen G., Puangplruk R., Kheawon N., Fucharoen S.	10.1111/j.1600-0609.2011.01616.x
Accuracy of immunochromatographic strip test in diagnosis of alpha-thalassemia-1 carrier	Wanapirak C., Piyamongkol W., Sirichotiyakul S., Tayapiwatana C., Kasinrerk W., Tongsong T.	

Hematological characteristics and effective screening for compound heterozygosity for Hb constant spring and deletional α+-thalassemia	Uaprasert N., Rojnuckarin P., Settapiboon R., Amornsiriwat S., Sutcharitchan P.	10.1002/ajh.22033
Quantitative analysis of Hb Bart's in cord blood by capillary electrophoresis system	Munkongdee T., Pichanun D., Butthep P., Klamchuen S., Chalermpolprapa V., Winichagoon P., Svasti S., Fucharoen S.	10.1007/s00277-010-1137-4
Molecular lesion frequency of hemoglobin gene disorders in Taiwan	Liu S.-C., Peng C.-T., Lin T.-H., Wang S.-J., Shih M.-C., Tien N., Chang C.-C., Lu J.-J., Lin C.-Y.	10.3109/03630269.2011.572524
Molecular characterization of two new mutations of α° thalassemia in two Spanish families (mutation - ED and - GP) [Caracterización molecular de dos nuevas mutaciones de α° talasemia en 2 familias españolas (mutación - ED y - GP)]	Villegas A., Ropero P., Anguita E., Hernández A., Polo M., González F.A.	10.1016/j.medcli.2011.01.015
Thalassemia and hemoglobinopathies in Southeast Asian newborns: Diagnostic assessment using capillary electrophoresis system	Srivorakun H., Fucharoen G., Changtrakul Y., Komwilaisak P., Fucharoen S.	10.1016/j.clinbiochem.2011.01.006
Hb phimai [β72(E16)Ser→Thr]: A novel β-globin structural variant found in association with Hb constant spring in pregnancy	Singsanan S., Srivorakun H., Fucharoen G., Puangplruk R., Fucharoen S.	10.3109/03630269.2011.557171
Molecular spectrum of α- and β-globin gene mutations detected in the population of Guangxi Zhuang Autonomous Region, People's Republic of China	Zheng C.-G., Liu M., Du J., Chen K., Yang Y., Yang Z.	10.3109/03630269.2010.547429
Molecular and hematological characterization of hemoglobin H disease in the Bengali population of Kolkata, India	Dastidar R., Gajra B., De M.	10.1089/gtmb.2010.0062
Correlation of beta-thalassemia mutations with alpha-thalassemia: An experience of the southwestern region of Iran	Rahim F.	10.1179/102453310X12719010991821
HbE/β-thalassemia: Basis of marked clinical diversity	Olivieri N.F., Pakbaz Z., Vichinsky E.	10.1016/j.hoc.2010.08.008
Molecular screening of the Hbs Constant Spring (codon 142, TAA>CAA, α2) and Paksé (codon 142, TAA>TAT, α2) mutations in Thailand	Pichanun D., Munkongdee T., Klamchuen S., Butthep P., Winichagoon P., Fucharoen S., Svasti S.	10.3109/03630269.2010.526914

Thalassemia and iron deficiency in a group of northeast Thai school children: Relationship to the occurrence of anemia	Panomai N., Sanchaisuriya K., Yamsri S., Sanchaisuriya P., Fucharoen G., Fucharoen S., Schelp F.P.	10.1007/s00431-010-1218-3
Hemoglobin H disease due to a de novo mutation at the α2-globin gene and an inherited common α-thalassemia deletion found in a Chinese boy	Zhu C., Yu W., Xie J., Chen L., Ding H., Shang X., Xu X.	10.1016/j.bcmd.2010.07.005
Hemoglobin Q-Thailand related disorders: Origin, molecular, hematological and diagnostic aspects	Singsanan S., Karnpean R., Fucharoen G., Sanchaisuriya K., Sae-ung N., Fucharoen S.	10.1016/j.bcmd.2010.06.001
Hemoglobin H disease in Guangxi province, Southern China: Clinical review of 357 patients	Yin X.-L., Zhang X.-H., Zhou T.-H., Zhang T.-L., Luo R.-G., Wang L., Zhou Y.-L., Chen Y.-S., Kong X.-J., Liang B., He Y.-Y., Peng L., Lu L.-B., Fang S.-P., Wu Z.-K.	10.1159/000314058
Prevalence of fractures among Thais with Thalassaemia syndromes	Sutipornpalangkul W., Janechetsadatham Y., siritanaratkul N., Harnroongroj T.	
Complex interaction of Hb Q-Thailand and Hb e with α0-thalassemia and hereditary persistence of fetal hemoglobin in a Chinese family	Zheng W., Liu Y., Chen D., Rong K., Ge Y., Gong C., Chen H.	10.1007/s00277-010-0935-z
Hydrops fetalis in the stillborn: A series from the central region of Thailand	Taweevisit M., Thorner P.S.	10.2350/09-12-0771-OA.1
Hb Koya Dora [α142, Term→Ser (TAA>TCA in α2)]: A rare mutation of the α2 gene stop codon associated with α-Thalassemia	Brennan S.O., Ryken S., Chan T.	10.3109/03630269.2010.486344
Application of an expanded multiplex genotyping assay for the simultaneous detection of Hemoglobin Constant Spring and common deletional α-thalassemia mutations	Kidd J.L., Azimi M., Lubin B., Vichinsky E., Hoppe C.	10.1111/j.1751-553X.2009.01197.x
Thalassemia as a global health problem: Recent progress toward its control in the developing countries	Weatherall D.J.	10.1111/j.1749-6632.2010.05546.x
Pulmonary hypertension in thalassemia	Morris C.R., Vichinsky E.P.	10.1111/j.1749-6632.2010.05580.x
Large scale screening for haemoglobin disorders in southern Vietnam: Implications for avoidance and management	O'Riordan S., Hien T.T., Miles K., Allen A., Quyen N.N., Hung N.Q., Anh D.Q., Tuyen L.N., Khoa D.B., Thai C.Q., Triet D.M., Phu N.H., Dunstan S., Peto T., Clegg J., Farrar J., Weatherall D.	10.1111/j.1365-2141.2010.08237.x

Sensitivity and specificity of simple erythrocyte osmotic fragility test for screening of alpha-thalassemia-1 and beta-thalassemia trait in pregnant women	Tongprasert F., Sirichotiyakul S., Piyamongkol W., Tongsong T.	10.1159/000271779
[Detection of deletional alpha-thalassemia from free fetal DNA in maternal plasma].	Chen Y., Ou Q.S., Zhou H.R.	
Prevention of severe thalassemia in northeast Thailand: 16 Years of experience at a single university center	Yamsri S., Sanchaisuriya K., Fucharoen G., Sae-ung N., Ratanasiri T., Fucharoen S.	10.1002/pd.2514
Molecular confirmatory testing of hemoglobin Constant Spring by real-time polymerase chain reaction SYBR Green1 with high-resolution melting analysis	Panyasai S., Sukunthamala K., Pornprasert S.	10.1111/j.1600-0609.2010.01437.x
α-thalassaemia	Harteveld C.L., Higgs D.R.	10.1186/1750-1172-5-13
Improvement of αo-thalassemia screening using combined osmotic fragility, dichlorophenolindophenol and Hb H inclusion tests	Chaibunruang A., Pornphannukool S., Sae-Ung N., Fucharoen G., Sanchaisuriya K., Fucharoen S.	
A comprehensive, simple molecular assay of common deletions and mutations causing a-thalassemia in Southeast Asia and southern China	Liu J., Jia X., Tang N., Wang L., Han H., Cai R., Wang Q., Xiao B.	10.1002/ajh.21671
Rapid diagnosis of α-thalassemia by melting curve analysis	Munkongdee T., Vattanaviboon P., Thummarati P., Sewamart P., Winichagoon P., Fucharoen S., Svasti S.	10.2353/jmoldx.2010.090136
High resolution DNA melting analysis: An application for prenatal control of α-thalassemia	Sirichotiyakul S., Wanapirak C., Saetung R., Sanguansermsri T.	10.1002/pd.2480
Prenatal diagnosis of hemoglobin Bart's disease: What is the noninvasive approach?	Li D.-Z.	10.1002/pd.2477
Interactions of hemoglobin Lepore (δβ hybrid hemoglobin) with various hemoglobinopathies: A molecular and hematological characteristics and differential diagnosis	Chaibunruang A., Srivorakun H., Fucharoen S., Fucharoen G., Sae-ung N., Sanchaisuriya K.	10.1016/j.bcmd.2009.11.008
Molecular diversity of hemoglobin H disease in India	Nadkarni A.H., Nair S.B., Italia K.Y., Warang P., Dalvi M., Ghosh K., Colah R.B.	10.1309/AJCP70ORBRURVSJM
Hematological features and molecular lesions of	Lin H.-J., Shih M.-C., Peng C.-T., Liu T.-C., Chen K.-W., Shih H.-C., Chang J.-G.	10.1111/j.1751-553X.2008.01095.x

hemoglobin gene disorders in Taiwanese patients		
