# Supplementary material for: Prevalence of Alpha(α)-Thalassemia in Southeast Asia (2010–2020): A Meta-Analysis Involving 83,674 Subjects
Source: Int J Environ Res Public Health. 2020 Oct 9;17(20):7354. doi: 10.3390/ijerph17207354 (PMC7600098; doi:10.3390/ijerph17207354)
Supplement: Supplementary file 1 [file ijerph-17-07354-s001.zip › ijerph-909078-supplementary/Table S5. The Pubmed literature (N = 126) included in the analysis..pdf]

Table S5: The Pubmed literature (*N* = 126) included in the analysis.

Title	Authors	Details
Management and Outcomes of Fetal Hydrops in a Tertiary Care Centre in Singapore.	Thong XY, Lee LY, Chia DA, Wong YC, Biswas A.	Ann Acad Med Singapore. 2017 Jan;46(1):4-10.
Diagnosis of common hemoglobinopathies among South East Asian population using capillary isoelectric focusing system.	Srivorakun H, Fucharoen G, Sanchaisuriya K, Fucharoen S.	Int J Lab Hematol. 2017 Feb;39(1):101-111. doi: 10.1111/ijlh.12585.
Molecular Understanding of Non-Transfusion-Dependent Thalassemia Associated with Hemoglobin E-Î²-Thalassemia in Northeast Thailand.	Yamsri S, Pakdee N, Fucharoen G, Sanchaisuriya K, Fucharoen S.	Acta Haematol. 2016;136(4):233-239.
HbE/Î²-Thalassemia and Oxidative Stress: The Key to Pathophysiological Mechanisms and Novel Therapeutics.	Hirsch RE, Sibmooh N, Fucharoen S, Friedman JM.	Antioxid Redox Signal. 2016 Nov 28. [Epub ahead of print]
A Number of Cases in Iran Presenting with Coinheritance of Hemoglobin-H Disease and Beta-Thalassemia Minor.	Zarei T, Dehbozorgian J, Imanifard J, Setoodegan F, Karimi M.	Hemoglobin. 2016 Sep;40(5):316-318.
[Gene Diagnosis and Analysis of Clinical Hematological Phenotype of Thailand Deleted Î±-Thalassemia 1].	Lin N, Huang HL, Wang Y, Zheng L, Fang XQ, Cai MY, Wang LS, Liu HK, Xu LP, Lin Y.	Zhongguo Shi Yan Xue Ye Xue Za Zhi. 2016 Aug;24(4):1116-20. doi: 10.7534/j.issn.1009-2137.2016.04.029. Chinese.
Occurrence of the -â€‰-(SEA), -â€‰-(THAI) and -â€‰-(FIL) Î±-Thalassemia-1 Carriers from a 7-Year Study at Ramathibodi Hospital, Bangkok, Thailand.	Pongjantharasatien K, Banyatsuppasin W, Pounsawat S, Jindadamrongwech S.	Hemoglobin. 2016 Aug;40(4):283-4. doi: 10.1080/03630269.2016.1189932.
Rapid detection of Î±-thalassaemia variants using droplet digital PCR.	Lee TY, Lai MI, Ramachandran V, Tan JA, Teh LK, Othman R, Hussein NH, George E.	Int J Lab Hematol. 2016 Aug;38(4):435-43. doi: 10.1111/ijlh.12520.
Screening of (-SEA) Î±-thalassaemia using an immunochromatographic strip assay for the Î¶-globin chain in a population with a high prevalence and heterogeneity of haemoglobinopathies.	Jomoui W, Fucharoen G, Sanchaisuriya K, Fucharoen S.	J Clin Pathol. 2017 Jan;70(1):63-68. doi: 10.1136/jclinpath-2016-203765.
Analysis of Î±1 and Î±2 globin genes among patients with hemoglobin Adana in Malaysia.	Lee TY, Lai MI, Ismail P, Ramachandran V, Tan JA, Teh LK, Othman R, Hussein NH, George E.	Genet Mol Res. 2016 Apr 7;15(2). doi: 10.4238/gmr.15027400.
Spectrum of Common Î±-Globin Deletion Mutations in the Southern Region of Vietnam.	Bui Thi Kim L, Phu Chi D, Hoang Thanh C.	Hemoglobin. 2016 Jun;40(3):206-7. doi: 10.3109/03630269.2016.1166126.
Molecular Epidemiology of Hemoglobinopathies in Cambodia.	Munkongdee T, Tanakulmas J, Butthep P, Winichagoon P, Main B, Yiannakis M, George J, Devenish R, Fucharoen S, Svasti S.	Hemoglobin. 2016 Jun;40(3):163-7. doi: 10.3109/03630269.2016.1158723.

The prevalence of alpha-thalassemia amongst Tai and Mon-Khmer ethnic groups residing in northern Thailand: A population-based study.	Lithanatudom P, Khampan P, Smith DR, Svasti S, Fucharoen S, Kangwanpong D, Kampuansai J.	Hematology. 2016 Sep;21(8):480-5. doi: 10.1080/10245332.2016. 1148374.
Hb lepore/Î²0-thalassaemia with Î±+-thalassaemia interactions, a potential diagnostic pitfall.	Alauddin H, Mohamad Nasir S, Ahadon M, Raja Sabudin RZ, Ithnin A, Hussin NH, Alias H, Loh CK, Abdul Latiff Z, Abdul Murad NA, Othman A.	Malays J Pathol. 2015 Dec;37(3):287-92.
Hemoglobin Constant Spring among Southeast Asian Populations: Haplotypic Heterogeneities and Phylogenetic Analysis.	Jomoui W, Fucharoen G, Sanchaisuriya K, Nguyen VH, Fucharoen S.	PLoS One. 2015 Dec 18;10(12):e0145230. doi: 10.1371/journal.pone.01 45230.
Hemoglobin Variants in Northern Thailand: Prevalence, Heterogeneity and Molecular Characteristics.	Panyasai S, Fucharoen G, Fucharoen S.	Genet Test Mol Biomarkers. 2016 Jan;20(1):37-43. doi: 10.1089/gtmb.2015.0182 .
DETECTION OF DELETION Î±(+)-THALASSEMIA MUTATION [-Î± (3.7), -Î± (4.2)] BY QUANTITATIVE PCR ASSAY.	Seeratanachot T, Shimbhu D, Charoenkwan P, Sanguansermsri T.	Southeast Asian J Trop Med Public Health. 2015 Jan;46(1):110-5.
The Homozygous Hemoglobin EE Genotype and Chronic Inflammation Are Associated with High Serum Ferritin and Soluble Transferrin Receptor Concentrations among Women in Rural Cambodia.	Karakochuk CD, Whitfield KC, Rappaport AI, Barr SI, Vercauteren SM, McLean J, Prak S, Hou K, Talukder A, Devenish R, Green TJ.	J Nutr. 2015 Dec;145(12):2765-73. doi: 10.3945/jn.115.218636.
Molecular Heterogeneity of Thalassemia among Pregnant Laotian Women.	Wongprachum K, Sanchaisuriya K, Dethvongphanh M, Norcharoen B, Htalongsengchan B, Vidamaly V, Sanchaisuriya P, Fucharoen S, Fucharoen G, Schelp FP.	Acta Haematol. 2016;135(2):65-9. doi: 10.1159/000438739.
Correlation of BACH1 and Hemoglobin E/Beta-Thalassemia Globin Expression.	Lee TY, Muniandy L, Teh LK, Abdullah M, George E, Sathar J, Lai MI.	Turk J Haematol. 2016 Mar 5;33(1):15-20. doi: 10.4274/tjh.2014.0197.
A novel gap-PCR with high resolution melting analysis for the detection of Î±-thalassaemia Southeast Asian and Filipino Î²Â°-thalassaemia deletion.	Kho SL, Chua KH, George E, Tan JA.	Sci Rep. 2015 Sep 14;5:13937. doi: 10.1038/srep13937.
Phenotype and Genotype in a Cohort of 312 Adult Patients with Nontransfusion-Dependent Thalassemia in Northeast Thailand.	Prayalaw P, Teawtrakul N, Jetsrisuparb A, Pongudom S, Fucharoen G, Fucharoen S.	Acta Haematol. 2016;135(1):15-20. doi: 10.1159/000435802.
The Spectrum of Î±-Thalassemia Mutations in Kermanshah Province, West Iran.	Alibakhshi R, Mehrabi M, Omidniakan L, Shafieenia S.	Hemoglobin. 2015;39(6):403-6. doi: 10.3109/03630269.2015. 1070732.
Coexistence of Malaria and Thalassemia in Malaria Endemic Areas of Thailand.	Kuesap J, Chaijaroenkul W, Rungsihirunrat K, Pongjantharasatien K, Na-Bangchang K.	Korean J Parasitol. 2015 Jun;53(3):265-70. doi: 10.3347/kjp.2015.53.3.26 5.

Interaction of Hb Grey Lynn (Vientiane) [Î±91(FG3)Leu>Phe (Î±1)] with Hb E [Î²26(B8) Glu>Lys] and Î±(+)-thalassemia: Molecular and Hematological Analysis.	Singha K, Fucharoen G, Fucharoen S.	Clin Lab. 2015;61(5-6):631-5.
Molecular spectrum of Î±-globin gene mutations in the Aegean region of Turkey: first observation of three Î±-globin gene mutations in the Turkish population.	Onay H, Aykut A, Karaca E, Durmaz A, Solmaz AE, Ã‡oÄŸulu Ã–, AydÄ±nok Y, Vergin C, Ã–zkÄ±nay F.	Int J Hematol. 2015 Jul;102(1):1-6. doi: 10.1007/s12185-015-1796-y.
Implementation of newborn screening for hemoglobin h disease in mainland china.	Xie XM, Zhou JY, Li J, Li R, Liao C, Li DZ.	Indian J Hematol Blood Transfus. 2015 Jun;31(2):242-6. doi: 10.1007/s12288-014-0432-y.
Sonographic markers of fetal Î±-thalassemia major.	Li X, Zhou Q, Zhang M, Tian X, Zhao Y.	J Ultrasound Med. 2015 Feb;34(2):197-206. doi: 10.7863/ultra.34.2.197. Review.
Genetic hemoglobin disorders rather than iron deficiency are a major predictor of hemoglobin concentration in women of reproductive age in rural prey Veng, Cambodia.	Karakochuk CD, Whitfield KC, Barr SI, Lamers Y, Devlin AM, Vercauteren SM, Kroeun H, Talukder A, McLean J, Green TJ.	J Nutr. 2015 Jan;145(1):134-42. doi: 10.3945/jn.114.198945.
Î±-thalassemia-associated hydrops fetalis: A rare cause of thyrotoxic cardiomyopathy.	Tumian NR, Wong M, Wong CL.	J Obstet Gynaecol Res. 2015 Jun;41(6):967-70. doi: 10.1111/jog.12648.
A large cohort of Î²(+)-thalassemia in Thailand: molecular, hematological and diagnostic considerations.	Yamsri S, Singha K, Prajantasen T, Taweenan W, Fucharoen G, Sanchaisuriya K, Fucharoen S.	Blood Cells Mol Dis. 2015 Feb;54(2):164-9. doi: 10.1016/j.bcmd.2014.11.008.
Prevalence of anemia, iron deficiency, thalassemia and glucose-6-phosphate dehydrogenase deficiency among hill-tribe school children in Omkoi District, Chiang Mai Province, Thailand.	Yanola J, Kongpan C, Pornprasert S.	Southeast Asian J Trop Med Public Health. 2014 Jul;45(4):920-5.
Transfusion-dependent thalassemia in Northern Sarawak: a molecular study to identify different genotypes in the multi-ethnic groups and the importance of genomic sequencing in unstudied populations.	Tan JA, Chin SS, Ong GB, Mohamed Unni MN, Soosay AE, Gudum HR, Kho SL, Chua KH, Chen JJ, George E.	Public Health Genomics. 2015;18(1):60-4. doi: 10.1159/000368342.
Immune response of thalassemia major patients in Indonesia with and without splenectomy.	Sari TT, Gatot D, Akib AA, Bardosono S, Hadinegoro SR, Harahap AR, Idjradinata PS.	Acta Med Indones. 2014 Jul;46(3):217-25.
The correlation of Î±-globin gene mutations and the XmnI polymorphism with clinical severity of Hb E/Î²-thalassemia.	Charoenkwan P, Teerachaimahit P, Sanguansermsri T.	Hemoglobin. 2014;38(5):335-8. doi: 10.3109/03630269.2014.952744.
Known and new hemoglobin A2 variants in Thailand and implication for Î²-thalassemia screening.	Panyasai S, Fucharoen G, Fucharoen S.	Clin Chim Acta. 2015 Jan 1;438:226-30. doi:

		10.1016/j.cca.2014.09.003.
ARKRAY ADAMS A1c HA-8180T Analyzer for Diagnosis of Thalassemia and Hemoglobinopathies Common in Southeast Asia.	Kunwandee J, Srivorakun H, Fucharoen G, Sanchaisuriya K, Fucharoen S.	Lab Med. 2014 Summer;45(3):e112-21. doi: 10.1309/LMMH649POETQREXL.
Hb Cibeles [Î±2 CD25(B6) (Gly â†’ Asp)]: a novel alpha chain variant causing alpha-thalassemia.	de la Fuente-Gonzalo F, Nieto JM, Vinuesa L, Sevilla J, DÃaz-Mediavilla J, Villegas A, GonzÃ¡lez FA, Ropero P.	Int J Hematol. 2014 Dec;100(6):599-601. doi: 10.1007/s12185-014-1663-2.
Hemoglobin Q-Thailand and its combinations with other forms of thalassemia or hemoglobinopathies in northern Thailand.	Panyasai S, Pornprasert S.	Clin Lab. 2014;60(7):1099-103.
The associations of SEA-alpha thalassemia 1, XmnI-Ggamma polymorphism and beta-globin gene mutations with the clinical severity of beta-thalassemia syndrome in northern Thailand.	Tatu T, Sritong W, Sa-Nguansermsri T.	J Med Assoc Thai. 2014 Mar;97(3):300-7.
New mathematical formula for differentiating thalassemia trait and iron deficiency anemia in thalassemia prevalent area: a study in healthy school-age children.	Sirachainan N, Iamsirirak P, Charoenkwan P, Kadegasem P, Wongwerawattanakoon P, Sasanakul W, Chansatitporn N, Chuansumrit A.	Southeast Asian J Trop Med Public Health. 2014 Jan;45(1):174-82.
Molecular characterization of Î±- and Î²-thalassaemia among Malay patients.	Yatim NF, Rahim MA, Menon K, Al-Hassan FM, Ahmad R, Manocha AB, Saleem M, Yahaya BH.	Int J Mol Sci. 2014 May 19;15(5):8835-45. doi: 10.3390/ijms15058835.
A case series of Î±-thalassemia intermedia due to compound heterozygosity for Hb Adana [HBA2: c179G>A (or HBA1); p.Gly60Asp] with other Î±-thalassemias in Malay families.	Alauddin H, Jaapar NA, Azma RZ, Ithnin A, Razak NF, Loh CK, Alias H, Abdul-Latiff Z, Othman A.	Hemoglobin. 2014;38(4):277-81. doi: 10.3109/03630269.2014.916720.
Molecular characteristic of alpha thalassaemia among patients diagnosed in UKM Medical Centre.	Azma RZ, Ainoon O, Hafiza A, Azlin I, Noor Farisah AR, Nor Hidayati S, Noor Hamidah H.	Malays J Pathol. 2014 Apr;36(1):27-32.
Interaction of hemoglobin Grey Lynn (Vientiane) with a non-deletional Î±(+)-thalassemia in an adult Thai proband.	Singha K, Fucharoen G, Fucharoen S.	Biochem Med (Zagreb). 2014 Feb 15;24(1):167-74. doi: 10.11613/BM.2014.019.
Criteria for detection of alpha-thalassemia-1 Thai type deletion in routine laboratory.	Pornprasert S, Punyamung M, Treesuwan K.	Clin Lab. 2013;59(11-12):1423-7.
Effect of genotype on pulmonary hypertension risk in patients with thalassemia.	Teawtrakul N, Ungprasert P, Pussadhamma B, Prayalaw P, Fucharoen S, Jetsrisuparb A, Pongudom S, Sirijerachai C, Chansung K, Wanitpongpun C, Chuncharunee S.	Eur J Haematol. 2014;92(5):429-34. doi: 10.1111/ejh.12261.
Hemoglobin Constant Spring is markedly high in women of an ethnic minority group in Vietnam: a community-based survey and hematologic features.	Nguyen VH, Sanchaisuriya K, Wongprachum K, Nguyen MD, Phan TT, Vo VT, Sanchaisuriya P, Fucharoen S, Schelp FP.	Blood Cells Mol Dis. 2014 Apr;52(4):161-5. doi: 10.1016/j.bcmd.2013.12.002.

A newly modified hemoglobin H inclusion test as a secondary screening for Î±(0)-thalassemia in Southeast Asian populations.	Fucharoen G, Yooyen K, Chaibunruang A, Fucharoen S.	Acta Haematol. 2014;132(1):10-4. doi: 10.1159/000355187.
Severe Î±-thalassemia intermedia due to a compound heterozygosity for the highly unstable Hb Adana (HBA2: c.179G>A) and a novel codon 24 (HBA2: c.75T>A) mutation.	Megawati D, Nainggolan IM, Swastika M, Susanah S, Mose JC, Harahap AR, Setianingsih I.	Hemoglobin. 2014;38(2):149-51. doi: 10.3109/03630269.2013.863206.
Genetic heterogeneity of hemoglobin AEBart's disease: a large cohort data from a single referral center in northeast Thailand.	Chaibunruang A, Karnpean R, Fucharoen G, Fucharoen S.	Blood Cells Mol Dis. 2014 Apr;52(4):176-80. doi: 10.1016/j.bcmd.2013.11.006.
A simple and highly sensitive ELISA for screening of the Î±-thalassemia-1 Southeast Asian-type deletion.	Pata S, Khummuang S, Pornprasert S, Tatu T, Kasinrerk W.	J Immunoassay Immunochem. 2014;35(2):194-206. doi: 10.1080/15321819.2013.838963.
Phenotypic expression of Hb F in common high Hb F determinants in Thailand: roles of Î±-thalassemia, 5' Î´-globin BCL11A binding region and 3' Î²-globin enhancer.	Prakobkaew N, Fucharoen S, Fuchareon G, Siriratmanawong N.	Eur J Haematol. 2014 Jan;92(1):73-9. doi: 10.1111/ejh.12201.
Diagnostic utility of isoelectric focusing and high performance liquid chromatography in neonatal cord blood screening for thalassemia and non-sickling hemoglobinopathies.	Uaprasert N, Settapiboon R, Amornsiriwat S, Sarnthammakul P, Thanapat T, Rojnuckarin P, Sutcharitchan P.	Clin Chim Acta. 2014 Jan 1;427:23-6. doi: 10.1016/j.cca.2013.09.041.
Five hemoglobin variants in a double heterozygote for Î±- and Î²-globin chain defects.	Singha K, Fucharoen G, Fucharoen S.	Acta Haematol. 2014;131(2):71-5. doi: 10.1159/000353123.
Distribution of alpha thalassaemia gene variants in diverse ethnic populations in malaysia: data from the institute for medical research.	Ahmad R, Saleem M, Aloysious NS, Yelumalai P, Mohamed N, Hassan S.	Int J Mol Sci. 2013 Sep 10;14(9):18599-614. doi: 10.3390/ijms140918599.
Fetal cardiac circumference derived by spatiotemporal image correlation as a predictor of fetal hemoglobin Bart disease at midpregnancy.	Siwawong W, Tongprasert F, Srisupundit K, Luewan S, Tongsong T.	J Ultrasound Med. 2013 Aug;32(8):1483-8. doi: 10.7863/ultra.32.8.1483.
Distribution of alpha thalassaemia in 16 year old Malaysian Students in Penang, Melaka and Sabah.	Rahimah AN, Nisha S, Safiah B, Roshida H, Punithawathy Y, Nurul H, Syahzuwan H, Zubaidah Z.	Med J Malaysia. 2012 Dec;67(6):565-70.
Molecular and hematological studies in a large cohort of Î±(0)-thalassemia in northeast Thailand: data from a single referral center.	Chaibunruang A, Prommetta S, Yamsri S, Fucharoen G, Sae-Ung N, Sanchaisuriya K, Fucharoen S.	Blood Cells Mol Dis. 2013 Aug;51(2):89-93. doi: 10.1016/j.bcmd.2013.04.003.
Thalassemia and hemoglobinopathies in Thua Thien Hue Province, Central Vietnam.	Nguyen HV, Sanchaisuriya K, Nguyen D, Phan HT, Siridamrongvattana S, Sanchaisuriya P, Fucharoen S, Fucharoen G, Schelp FP.	Hemoglobin. 2013;37(4):333-42. doi: 10.3109/03630269.2013.790829.

Problems in determining thalassemia carrier status in a program for prevention and control of severe thalassemia syndromes: a lesson from Thailand.	Viprakasit V, Limwongse C, Sukpanichnant S, Ruangvutilert P, Kanjanakorn C, Glomglao W, Sirikong M, Utto W, Tanphaichitr VS.	Clin Chem Lab Med. 2013 Aug;51(8):1605-14. doi: 10.1515/cclm-2013-0098.
Evidence of recent natural selection on the Southeast Asian deletion (--(SEA)) causing Î±-thalassemia in South China.	Qiu QW, Wu DD, Yu LH, Yan TZ, Zhang W, Li ZT, Liu YH, Zhang YP, Xu XM.	BMC Evol Biol. 2013 Mar 11;13:63. doi: 10.1186/1471-2148-13-63.
Hemoglobin Pyrgos with hemoglobin H disease: new triple heterozygosity.	Insiripong S, Jitpakdeebodin V, Jopang Y, Fucharoen S.	Southeast Asian J Trop Med Public Health. 2012 Sep;43(5):1258-61.
Low cost biosensor-based molecular differential diagnosis of Î±-thalassemia (Southeast Asia deletion).	Wangmaung N, Promptmas C, Chomean S, Sanchomphu C, Ittarat W.	Clin Chem Lab Med. 2013 Jun;51(6):1199-205. doi: 10.1515/cclm-2012-0732.
The prevention of thalassemia.	Cao A, Kan YW.	Cold Spring Harb Perspect Med. 2013 Feb 1;3(2):a011775. doi: 10.1101/cshperspect.a011775. Review.
Evidence of differential selection for the -Î±(3.7) and -Î±(4.2) single-Î±-globin gene deletions within the same population.	Yap ZM, Sun KM, Teo CR, Tan AS, Chong SS.	Eur J Haematol. 2013 Mar;90(3):210-3. doi: 10.1111/ejh.12058.
Detection of coinherited Hb H-Constant Spring/PaksÃ© disease and Hb E by capillary electrophoresis and high performance liquid chromatography.	Pornprasert S, Waneesorn J.	Hemoglobin. 2013;37(2):176-82. doi: 10.3109/03630269.2012.752744.
HbA2 levels in Î²-thalassaemia carriers with the Filipino Î²0-deletion: are the levels higher than what is found with non-deletional forms of Î²0-thalassaemia?	George E, Teh LK, Tan J, Lai MI, Wong L.	Pathology. 2013 Jan;45(1):62-5. doi: 10.1097/PAT.0b013e32835af7c1.
Association of Hb Thailand [Î±56(E5)Lysâ†'Thr] and Hb Phnom Penh [Î±117(GH5)-Ile-Î±118(H1)] with Î±(0)-thalassemia: molecular and hematological features and differential diagnosis.	Singha K, Srivorakun H, Fucharoen G, Changtrakul Y, Komwilaisak P, Jetsrisuparb A, Puangplruk R, Fucharoen S.	Hemoglobin. 2013;37(1):37-47. doi: 10.3109/03630269.2012.747964.
Identification of hemoglobin variants in samples received for glycated hemoglobin testing.	Saw S, Loh TP, Yin C, Sethi SK.	Clin Chim Acta. 2013 Jan 16;415:173-5. doi: 10.1016/j.cca.2012.10.043.
Prevalence of iron deficiency anaemia and thalassaemia trait among undergraduate medical students.	Azma RZ, Ainoon O, Azlin I, Hamenuddin H, Hadi NA, Tatt WK, Syazana IN, Asmaliza AM, Das S, Hamidah NH.	Clin Ter. 2012 Jul;163(4):287-91.
Artemisinin-resistant Plasmodium falciparum in Pursat province, western Cambodia: a parasite clearance rate study.	Amaratunga C, Sreng S, Suon S, Phelps ES, Stepniewska K, Lim P, Zhou C, Mao S, Anderson JM, Lindegardh N, Jiang H, Song J, Su XZ, White NJ, Dondorp AM, Anderson TJ, Fay MP, Mu J, Duong S, Fairhurst RM.	Lancet Infect Dis. 2012 Nov;12(11):851-8. doi: 10.1016/S1473-3099(12)70181-0.

Prevalence and hematological parameters of thalassemia in Tha Kradarn subdistrict Chachoengsao Province, Thailand.	Nillakupt K, Nathalang O, Arnutti P, Jindadamrongwech S, Boonsiri T, Panichkul S, Areekul W.	J Med Assoc Thai. 2012 May;95 Suppl 5:S124-32.
Unmasking Hb PaksÃ© (codon 142, TAA>TAT, Î±2) and its combinations in patients also carrying Hb Constant Spring (codon 142, TAA>CAA, Î±2) in northern Thailand.	Pornprasert S, Panyasai S, Treesuwan K.	Hemoglobin. 2012;36(5):491-6.
Development of a fluorescence immunochromatographic assay for the detection of zeta globin in the blood of (--(SEA)) Î±-thalassemia carriers.	Wen L, Zhu P, Liu Y, Pan Q, Qu Y, Xu X, Li X, Fu N.	Blood Cells Mol Dis. 2012 Oct 15-Dec 15;49(3-4):128-32. doi: 10.1016/j.bcmd.2012.05.011.
Genetic origin and interaction of the Filipino Î²â◎°-thalassemia with Hb E and Î±-thalassemia in a Thai family.	Yamsri S, Sanchaisuriya K, Fucharoen G, Fucharoen S.	Transl Res. 2012 Jun;159(6):473-6. doi: 10.1016/j.trsl.2011.10.008.
The definition and epidemiology of non-transfusion-dependent thalassemia.	Weatherall DJ.	Blood Rev. 2012 Apr;26 Suppl 1:S3-6. doi: 10.1016/S0268-960X(12)70003-6. Review.
Proxy indicators for identifying iron deficiency among anemic vegetarians in an area prevalent for thalassemia and hemoglobinopathies.	Wongprachum K, Sanchaisuriya K, Sanchaisuriya P, Siridamrongvattana S, Manpeun S, Schlep FP.	Acta Haematol. 2012;127(4):250-5. doi: 10.1159/000337032.
Hb A2/E levels found in co-inheritance with the Î±-thalassemia-1 - -(SEA)/type deletion and either Hb E or Î²-thalassemia.	Pornprasert S, Treesuwan K, Punyamung M, Kongthai K.	Hemoglobin. 2012;36(4):381-7. doi: 10.3109/03630269.2012.679375.
Thalassaemia screening among students in a secondary school in Ampang, Malaysia.	Jameela S, Sabirah SO, Babam J, Phan CL, Visalachy P, Chang KM, Salwana MA, Zuraidah A, Subramanian Y, Rahimah A.	Med J Malaysia. 2011 Dec;66(5):522-4.
A rare Hb H disease due to the - -(SEA) and 16.6 kb Î±-thalassemia-2 deletions.	Sroymora S, Jindadamrongwech S, Butthep P, Chuncharunee S.	Hemoglobin. 2012;36(2):200-4. doi: 10.3109/03630269.2012.655355.
Molecular study and genotype/phenotype correlation of Î² Thalassemia in Malaysia.	Sivalingam M, Looi ML, Zakaria SZ, Hamidah NH, Alias H, Latiff ZA, Ibrahim H, Jamal R.	Int J Lab Hematol. 2012 Aug;34(4):377-82. doi: 10.1111/j.1751-553X.2012.01405.x.
Hb H disease with various Î² hemoglobinopathies: molecular, hematological and diagnostic aspects.	Fucharoen S, Fucharoen G.	Hemoglobin. 2012;36(1):18-24. doi: 10.3109/03630269.2011.638406.
Micromapping of thalassemia and hemoglobinopathies in diferent regions of northeast Thailand and Vientiane, Laos People's Democratic Republic.	Tritipsombut J, Sanchaisuriya K, Phollarp P, Bouakhasith D, Sanchaisuriya P, Fucharoen G, Fucharoen S, Schelp FP.	Hemoglobin. 2012;36(1):47-56. doi: 10.3109/03630269.2011.637149.

Haemoglobinopathies in southeast Asia.	Fucharoen S, Winichagoon P.	Indian J Med Res. 2011 Oct;134:498-506. Review.
First description of a Hb A2 variant in Thailand. Identification of Hb A2-Melbourne [Î´43(CD2)Gluâ†'Lys] in Thai individuals.	Chaibunruang A, Fucharoen G, Fucharoen S.	Hemoglobin. 2012;36(1):80-4. doi: 10.3109/03630269.2011.599465.
Î±-Hemoglobin stabilizing protein: a modulating factor in thalassemias?	Wajcman H, Vasseur C, Pissard S, Baudin-Creuza V.	Hemoglobin. 2011;35(5-6):463-8. doi: 10.3109/03630269.2011.576354.
Cytokine-induced apoptosis of beta-thalassemia/hemoglobin E erythroid progenitor cells via nitric oxide-mediated process in vitro.	Kheansaard W, Panichob P, Fucharoen S, Tanyong DI.	Acta Haematol. 2011;126(4):224-30. doi: 10.1159/000329903.
Alpha thalassemia major--a hematological prelude to an oncological problem.	Pant S.	Med Hypotheses. 2011 Nov;77(5):786-7. doi: 10.1016/j.mehy.2011.07.037.
A rapid detection for Î±-thalassemia by PCR combined with dissociation curve analysis.	Jia X, Liu J, Wang L, Yao L, Tang N, Cai R, Xiao B.	Exp Mol Pathol. 2011 Oct;91(2):626-30. doi: 10.1016/j.yexmp.2011.06.013.
Detection of Î±-thalassemia-1 Southeast Asian and Thai type deletions and Î²-thalassemia 3.5-kb deletion by single-tube multiplex real-time PCR with SYBR Green1 and high-resolution melting analysis.	Pornprasert S, Wiengkum T, Srithep S, Chainoi I, Singboottra P, Wongwiwatthananukit S.	Korean J Lab Med. 2011 Jul;31(3):138-42. doi: 10.3343/kjlm.2011.31.3.138.
Interference of hemoglobin Hope on beta-thalassemia diagnosis by the capillary electrophoresis Method.	Panyasai S, Sukunthamala K, Jaiping K, Wongwiwatthananukit S, Singboottra P, Pornprasert S.	Am J Clin Pathol. 2011 Jul;136(1):14-8. doi: 10.1309/AJCP1BT2MGATKFHL.
Genotype and phenotype characterizations in a large cohort of Î²-thalassemia heterozygote with different forms of Î±-thalassemia in northeast Thailand.	Yamsri S, Sanchaisuriya K, Fucharoen G, Sae-Ung N, Fucharoen S.	Blood Cells Mol Dis. 2011 Aug 15;47(2):120-4. doi: 10.1016/j.bcmd.2011.05.003.
Evaluation of the URIT-2900 automated hematology analyzer for screening of thalassemia and hemoglobinopathies in Southeast Asian populations.	Karnpean R, Pansuwan A, Fucharoen G, Fucharoen S.	Clin Biochem. 2011 Jul;44(10-11):889-93. doi: 10.1016/j.clinbiochem.2011.04.009.
Hematological characteristics and effective screening for compound heterozygosity for Hb constant spring and deletional Î±+ -thalassemia.	Uaprasert N, Rojnuckarin P, Settapiboon R, Amornsiriwat S, Sutcharitchan P.	Am J Hematol. 2011 Jul;86(7):615-7. doi: 10.1002/ajh.22033. No abstract available.
[Molecular characterization of two new mutations of Î±Â° thalassemia in two Spanish families (mutation --(ED) and --(GP))].	Villegas A, Ropero P, Anguita E, HernÃ¡ndez A, Polo M, AtaÃºlfo GonzÃ¡lez F.	Med Clin (Barc). 2011 May 28;136(15):674-7. doi:

		10.1016/j.medcli.2011.01.015. Spanish.
Public perceptions and attitudes toward thalassaemia: Influencing factors in a multi-racial population.	Wong LP, George E, Tan JA.	BMC Public Health. 2011 Mar 30;11:193. doi: 10.1186/1471-2458-11-193.
Presumptive diagnosis of common haemoglobinopathies in Southeast Asia using a capillary electrophoresis system.	Fucharoen G, Srivorakun H, Singsanan S, Fucharoen S.	Int J Lab Hematol. 2011 Aug;33(4):424-33. doi: 10.1111/j.1751-553X.2011.01301.x.
Hemoglobin Lepore EF Bart's disease: a molecular, hematological, and diagnostic aspects.	Chaibunruang A, Fucharoen G, Jetsrisuparb A, Fucharoen S.	Ann Hematol. 2011 Nov;90(11):1337-40. doi: 10.1007/s00277-011-1173-8.
Thalassemia and hemoglobinopathies in Southeast Asian newborns: diagnostic assessment using capillary electrophoresis system.	Srivorakun H, Fucharoen G, Changtrakul Y, Komwilaisak P, Fucharoen S.	Clin Biochem. 2011 Apr;44(5-6):406-11. doi: 10.1016/j.clinbiochem.2011.01.006.
Prevalence of fractures among Thais with thalassaemia syndromes.	Sutipornpalangkul W, Janechetsadatham Y, Siritanaratkul N, Harnroongroj T.	Singapore Med J. 2010 Oct;51(10):817-21.
Molecular screening of the Hbs Constant Spring (codon 142, TAA>CAA, Î±2) and PaksÃ© (codon 142, TAA>TAT, Î±2) mutations in Thailand.	Pichanun D, Munkongdee T, Klamchuen S, Butthep P, Winichagoon P, Fucharoen S, Svasti S.	Hemoglobin. 2010;34(6):582-6. doi: 10.3109/03630269.2010.526914.
High prevalence of alpha- and beta-thalassemia in the Kadazandusuns in East Malaysia: challenges in providing effective health care for an indigenous group.	Tan JA, Lee PC, Wee YC, Tan KL, Mahali NF, George E, Chua KH.	J Biomed Biotechnol. 2010;2010. pii: 706872. doi: 10.1155/2010/706872.
The prevalence of iron deficiency anemia in pregnant women in Nakhonsawan, Thailand.	Sukrat B, Suwathanapisate P, Siritawee S, Poungthong T, Phupongpankul K.	J Med Assoc Thai. 2010 Jul;93(7):765-70.
Hb Koya Dora [alpha142, Term-->Ser (TAA>TCA in alpha2)]: a rare mutation of the alpha2 gene stop codon associated with alpha-thalassemia.	Brennan SO, Ryken S, Chan T.	Hemoglobin. 2010;34(4):402-5. doi: 10.3109/03630269.2010.486344.
[Detection of deletional alpha-thalassemia from free fetal DNA in maternal plasma].	Chen Y, Ou QS, Zhou HR.	Zhongguo Shi Yan Xue Ye Xue Za Zhi. 2010 Jun;18(3):679-82. Chinese.
Epidemiology of stillbirth and strategies for its prevention in Vietnam.	Hirst JE, Arbuckle SM, Do TM, Ha LT, Jeffery HE.	Int J Gynaecol Obstet. 2010 Aug;110(2):109-13. doi: 10.1016/j.ijgo.2010.03.028.
Prevention of severe thalassemia in northeast Thailand: 16 years of experience at a single university center.	Yamsri S, Sanchaisuriya K, Fucharoen G, Sae-Ung N, Ratanasiri T, Fucharoen S.	Prenat Diagn. 2010 Jun;30(6):540-6. doi: 10.1002/pd.2514.
Large scale screening for haemoglobin disorders in southern Vietnam: implications for avoidance and management.	O'Riordan S, Hien TT, Miles K, Allen A, Quyen NN, Hung NQ, Anh DQ, Tuyen LN, Khoa DB, Thai CQ, Triet DM, Phu NH,	Br J Haematol. 2010 Aug;150(3):359-64. doi: 10.1111/j.1365-2141.2010.08237.x.

	Dunstan S, Peto T, Clegg J, Farrar J, Weatherall D.	
[Case of deletion type beta(0)-thalassemia found in an Asian (Malaysian) family living in Japan].	Harano K, Harano T.	Rinsho Byori. 2010 Apr;58(4):325-31. Japanese.
Thalassemia and iron deficiency in a group of northeast Thai school children: relationship to the occurrence of anemia.	Panomai N, Sanchaisuriya K, Yamsri S, Sanchaisuriya P, Fucharoen G, Fucharoen S, Schelp FP.	Eur J Pediatr. 2010 Nov;169(11):1317-22. doi: 10.1007/s00431-010-1218-3.
Improvement of alpha(0)-thalassemia screening using combined osmotic fragility, dichlorophenolindophenol and Hb H inclusion tests.	Chaibunruang A, Pornphannukool S, Sae-Ung N, Fucharoen G, Sanchaisuriya K, Fucharoen S.	Clin Lab. 2010;56(3-4):111-7.
A comprehensive, simple molecular assay of common deletions and mutations causing alpha-thalassemia in Southeast Asia and southern China.	Liu J, Jia X, Tang N, Wang L, Han H, Cai R, Wang Q, Xiao B.	Am J Hematol. 2010 May;85(5):370-2. doi: 10.1002/ajh.21671. No abstract available.
Two new alpha1-globin gene point mutations: Hb Nedlands (HBA1:c.86C>T) [alpha28(B9)Ala-->Val] and Hb Queens Park (HBA1:c.98T>A) [alpha32(B13)Met-->Lys].	Phylipsen M, Prior JF, Lim E, Lingam N, Finlayson J, Arkesteijn SG, Harteveld CL, Giordano PC.	Hemoglobin. 2010 Jan;34(2):123-6. doi: 10.3109/03630261003679854.
Complex interaction of Hb Q-Thailand and Hb E with alpha(0)-thalassemia and hereditary persistence of fetal hemoglobin in a Chinese family.	Zheng W, Liu Y, Chen D, Rong K, Ge Y, Gong C, Chen H.	Ann Hematol. 2010 Sep;89(9):883-8. doi: 10.1007/s00277-010-0935-z.
Cord blood screening for alpha-thalassemia and hemoglobin variants by isoelectric focusing in northern Thai neonates: correlation with genotypes and hematologic parameters.	Charoenkwan P, Taweephol R, Sirichotiyakul S, Tantiprabha W, Sae-Tung R, Suanta S, Sakdasirisathaporn P, Sanguansermsri T.	Blood Cells Mol Dis. 2010 Jun 15;45(1):53-7. doi: 10.1016/j.bcmd.2010.02.015.
Hydrops fetalis in the stillborn: a series from the central region of Thailand.	Taweevisit M, Thorner PS.	Pediatr Dev Pathol. 2010 Sep-Oct;13(5):369-74. doi: 10.2350/09-12-0771-OA.1.
High resolution DNA melting analysis: an application for prenatal control of alpha-thalassemia.	Sirichotiyakul S, Wanapirak C, Saetung R, Sanguansermsri T.	Prenat Diagn. 2010 Apr;30(4):348-51. doi: 10.1002/pd.2480.
Novel technique for rapid detection of alpha-globin gene mutations and deletions.	Liu J, Jia X, Tang N, Zhang X, Wu X, Cai R, Wang L, Liu Q, Xiao B, Zhu J, Wang Q.	Transl Res. 2010 Mar;155(3):148-55. doi: 10.1016/j.trsl.2009.10.003.
Sensitivity and specificity of simple erythrocyte osmotic fragility test for screening of alpha-thalassemia-1 and Beta-thalassemia trait in pregnant women.	Tongprasert F, Sirichotiyakul S, Piyamongkol W, Tongsong T.	Gynecol Obstet Invest. 2010;69(4):217-20. doi: 10.1159/000271779.
Interactions of hemoglobin Lepore (deltabeta hybrid hemoglobin) with various hemoglobinopathies: A molecular and hematological characteristics and differential diagnosis.	Chaibunruang A, Srivorakun H, Fucharoen S, Fucharoen G, Sae-ung N, Sanchaisuriya K.	Blood Cells Mol Dis. 2010 Mar 15;44(3):140-5. doi: 10.1016/j.bcmd.2009.11.008.

A comprehensive ethnic-based analysis of alpha thalassaemia allelle frequency in northern Thailand	Kulaphisit M, Kampuansai J, Leecharoenkiat K, Wathikthinnakon M, Kangwanpong D, Munkongdee T, Svasti S, Fucharoen S, Smith DR, Lithanatudom P.	Sci Rep. 2017 Jul 5;7(1):4690. doi: 10.1038/s41598-017-04957-2.
Genetic origin of Î±(0)-thalassemia (SEA deletion) in Southeast Asian populations and application to accurate prenatal diagnosis of Hb Bart's hydrops fetalis syndrome	Jomoui W, Fucharoen G, Sanchaisuriya K, Charoenwijitkul P, Maneesarn J, Xu X, Fucharoen S.	J Hum Genet. 2017 Aug;62(8):747-754. doi: 10.1038/jhg.2017.41. Epub 2017 Apr 6.
Molecular characteristics of thalassemia and hemoglobin variants in prenatal diagnosis program in northern Thailand	Mankhemthong K, Phusua A, Suanta S, Srisittipoj P, Charoenkwan P, Sanguansermsri T.	Int J Hematol. 2019 Oct;110(4):474-481. doi: 10.1007/s12185-019-02694-y. Epub 2019 Jun 25.
Impact of the detection of Î¶-globin chains and hemoglobin Bart's using immunochromatographic strip tests for Î±0-thalassemia (--SEA) differential diagnosis	Pata S, Laopajon W, Pongpaiboon M, Thongkum W, Polpong N, Munkongdee T, Paiboonsukwong K, Fucharoen S, Tayapiwatana C, Kasinrerk W.	PLoS One. 2019 Oct 29;14(10):e0223996. doi: 10.1371/journal.pone.0223996. eCollection 2019.
Molecular characteristics of Î±(+)-thalassemia (3.7â€¯kb deletion) in Southeast Asia: Molecular subtypes, haplotypic heterogeneity, multiple founder effects and laboratory diagnostics	Charoenwijitkul T, Singha K, Fucharoen G, Sanchaisuriya K, Thepphitak P, Wintachai P, Karnpean R, Fucharoen S.	Clin Biochem. 2019 Sep;71:31-37. doi: 10.1016/j.clinbiochem.2019.06.005. Epub 2019 Jun 12.
